# Electrolysis-Assisted Reduction of Dimethylformamide for Unactivated Alkene Functionalizations

**DOI:** 10.1021/jacs.5c17824

**Published:** 2026-03-02

**Authors:** Yifan Xi, Sulekha Sharma, C. Oliver Kappe, Gabriele Laudadio

**Affiliations:** † Institute of Chemistry, 27267 University of Graz, NAWI Graz, Heinrichstrasse 28, 8010 Graz, Austria; ‡ Center for Continuous Flow Synthesis and Processing (CCFLOW), Research Center Pharmaceutical Engineering GmbH (RCPE), Inffeldgasse 13, 8010 Graz, Austria

## Abstract

*N,N*-Dimethylformamide is a widely used solvent, which can be employed as a reagent under certain conditions. Among the different ionic and radical activation approaches, the direct single-electron reduction of this amide appears elusive, owing to its prohibitively low reductive potential. In this work, electrolysis-assisted generation of the *N,N*-dimethylformamide distonic radical anion is presented. Its synthetic applicability was established by the functionalization of unactivated alkenes. The transformation was validated across several mono- and disubstituted olefins, generating the corresponding hydroformylated and hydroaminomethylated products. Mechanistic experiments confirmed the generation of the distonic radical, demonstrating that this intermediate was generated at the surface of the magnesium anode. This work provides a new perspective on the activation of this commonly used solvent, paving the way to other radical transformations.

## Graphical abstract

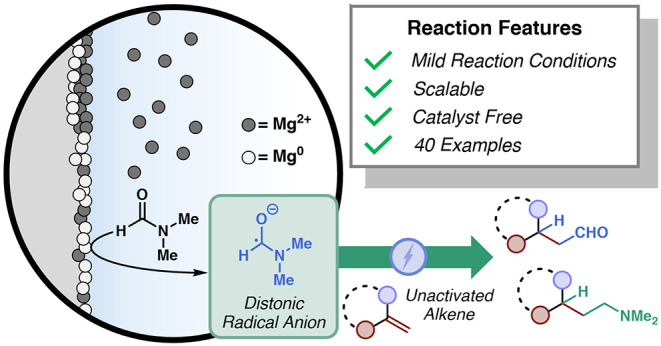

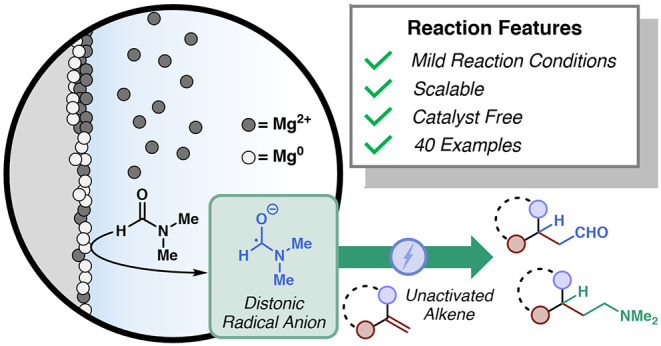

## Introduction

*N*,*N*-Dimethylformamide (DMF) is a polar aprotic solvent widely employed in organic synthesis.^1^ Owing to its high dielectric constant, it has the peculiar ability to dissolve a broad range of organic and inorganic compounds, standing out as the reaction medium of choice for many synthetic protocols.^2^ Furthermore, DMF is generally used in electrochemical reactions due to its remarkable reductive stability.^3^ Nevertheless, this versatile solvent can also turn into a useful reagent under certain conditions.^4^ Aside from the addition of Grignard reagents to the carbonyl part, typical of Bouveault aldehyde synthesis,^5^ several different direct activations of DMF have been discovered (Figure 1A). In this context, the most recognizable manipulation of this solvent is undeniably the formation of the Vilsmeier reagent, commonly obtained by subjecting the formamide to strong chlorinating reactants (e.g., POCl_3_).^6^ This aggressive electrophile has been traditionally used in the formylation of heteroarenes and alkenes.^6^ Other activation strategies revolved around the relatively low bond dissociation energy (BDE) of DMF.^7^ Several hydrogen atom transfer (HAT) processes have been established adopting thermochemical,^8–10^ photochemical,^11,12^ or electrochemical^13^ approaches. Leveraging the synthetic utility of these radicals, corresponding carbamoylation and α-methylamination reactions have been developed, depending on the nature of the HAT agent employed. This homolytic reactivity provided a different perspective on synthetic applications of this widely available chemical. To further exploit the potential of this molecule, other transformations could be envisioned by probing different activation pathways. Following this logic, single-electron transfer (SET) reduction of the carbonyl moiety would lead to a resourceful radical anion. However, achieving the generation of such a DMF intermediate appears elusive. Arguably, the inaccessibly low reductive potential prevents any mild generation of the aforementioned radical. Indeed, formal pinacol products could only be obtained employing harsh conditions, such as refluxing DMF in ethers or benzene in the presence of Li^0^, Na^0^, or K^0^ as competent reductants.^14^ Owing to this impractical redox behavior, any other synthetic application of this radical anion has been precluded. Nevertheless, sporadic appearances of this species can be found in the literature (Figure 1B). In 1990, Perichon and co-workers observed the overconsumption of the magnesium anode upon electrolysis of DMF, leading to the same dimerization product previously generated employing alkali metals.^15^ These experimental results were rationalized by the authors as an unusual reductive solvent degradation event caused by anodic magnesium. The spectroscopic evidence of this elusive radical anion was obtained in 2012 by Shkrob and Marin via EPR analysis.^16^ In their study, the longevity of this reactive radical (named radical X in their work) was justified by the stabilization originating from a solvation effect. The conclusions of these seminal results supported the hypothesis of a mild and controllable generation of the DMF radical anion via electrolysis. By carefully selecting the appropriate radical quencher, this strategy could unlock its synthetic applicability (Figure 1C). Among the possible acceptors, the use of alkenes was identified as an intuitive candidate to promote the utilization of such an intermediate.^17^ In particular, unactivated alkenes were chosen in order to circumvent competitive reductive activation of the double bond at the cathode.^18^ Employing this redox-inert alkene, the radical attack of the amide can be exploited, generating a carbon-centered radical (Figure 1D). This species undergoes a HAT quench, delivering the hemiaminal intermediate, which will lead to an aldehyde-containing product. This radical hydroformylation would offer an additional strategy to obtain this multifaceted functional handle, whose synthesis is dominated by transition-metal-catalyzed approaches.^19^

**Figure 1:**
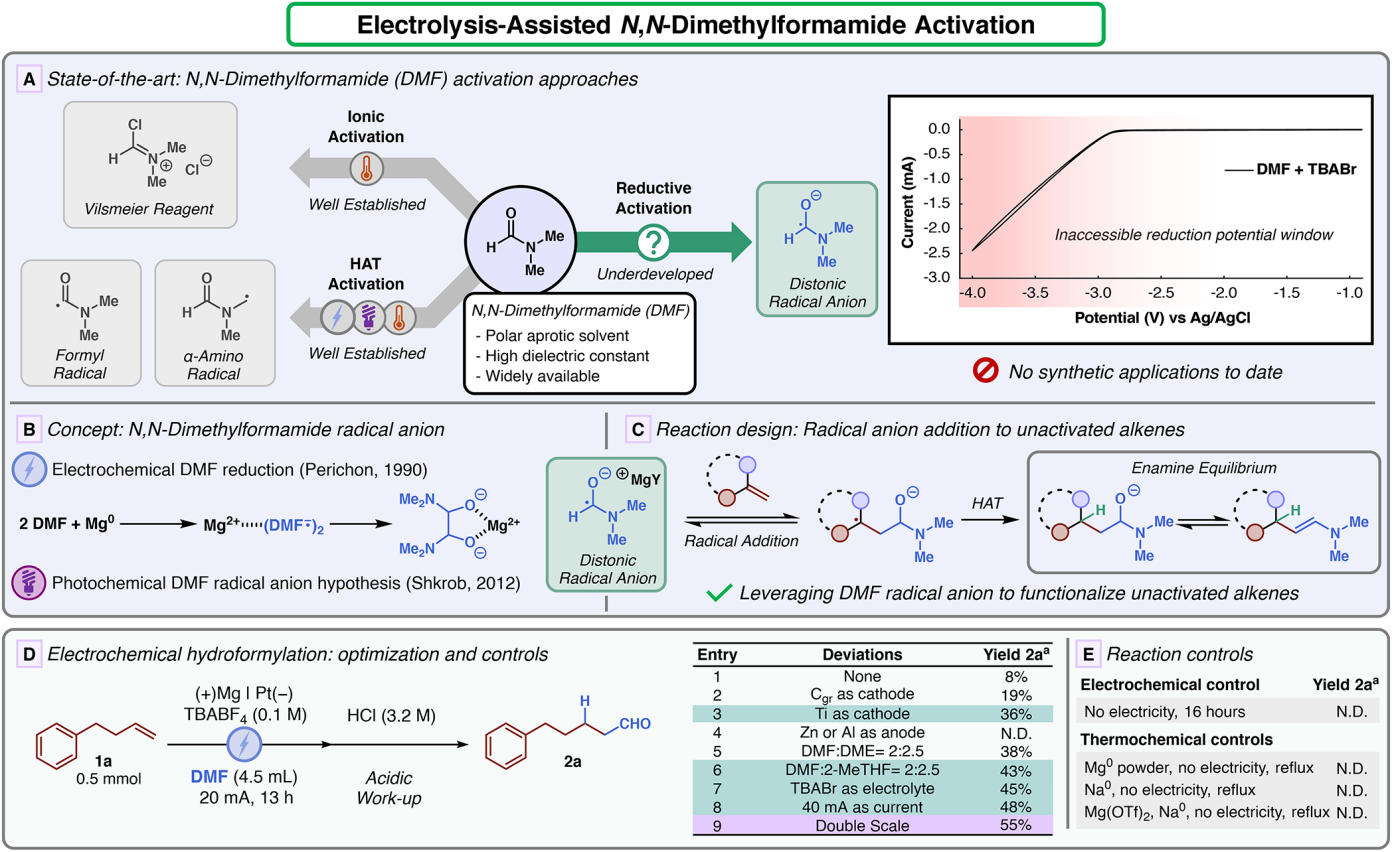
Activation of DMF. (A) State-of-the-art for DMF activation approaches. CV analysis of DMF (TBABr as the electrolyte; WE: GC; CE: Pt; RE: Ag/AgCl). (B) Concept development and relevant precedents on the DMF distonic radical. (C) Reaction design. (D) Optimization studies for the electrolysis promoted hydroformylation using the DMF radical anion. (E) Control experiments were performed for the electrochemical hydroformylation. ^a^GC-FID yield (1,3,5-trimethoxybenzene as an internal standard).

## Results and Discussion

### Reaction Optimization

Following this design, our investigation commenced by subjecting 4-phenyl-1-butene **1a** to electrolysis conditions with magnesium as the sacrificial anode and platinum as the cathode, using DMF as the solvent and tetrabutylammonium tetrafluoroborate (TBABF_4_) as the supporting electrolyte. After 13 h of electrolysis at 20 mA, the reaction mixture was extracted in acidic conditions, and 8% of the desired aldehyde was observed (Figure 1D, entry 1), with almost exclusive anti-Markovnikov selectivity (terminal/branched ∼ 20:1).^20^ Subsequently, different cathodes and anodes were screened to evaluate the impact of the electrode materials on the reaction. While several cathodes provided the desired product **2a**, titanium was identified to exhibit the best performance (Figure 1D, entry 3). Other sacrificial anodes such as aluminum and zinc were found completely ineffective (Figure 1D, entry 4). Similarly, the use of triethylamine as the sacrificial reductant in the presence of a platinum anode followed the same trend (see Supporting Information for further details). After identifying the best pair of electrodes (titanium as the cathode and magnesium as the anode), attention was focused on the nature of the electrolysis medium. At the end of the reaction, high viscosity was observed, which was attributed to the elevated concentration of magnesium salts. In order to mitigate this technical problem, it was reasoned that the addition of mildly complexating ethereal solvents would improve the solubility of the generated salts.^21^ A mixture of DMF and 1,2-dimethoxyethane (DME) provided a superior yield (Figure 1D, entry 5). The screening of different cosolvents revealed that biomass-derived 2-methyl tetrahydrofuran (2-MeTHF) provided the best performance (Figure 1D, entry 6). Next, the use of tetrabutylammonium bromide as the supporting electrolyte led to a better voltage profile (Figure 1D, entry 7).^22^ Increasing the current to 40 mA improved the reaction reproducibility with slightly better yield (48%, Figure 1D, entry 8). Finally, doubling the scale of reaction (1 mmol scale, 9 mL reaction volume) provided the best yield observed (55%, Figure 1D, entry 9). After optimization, the reaction mixture was subjected to several controls to verify the necessity of electrochemical conditions (Figure 1E). **2a** could not be generated by stirring the reaction mixture in the absence of electricity. To further exclude the formation of any organometallic species or other redox transformations, we probed exogenous chemical reductants. Refluxing the reaction mixture in the presence of magnesium powder did not lead to any formation of the product. The same outcome was observed when the reaction was carried out with metallic sodium under reflux. To finally confirm that the classical organometallic settings could not promote such a transformation, the reaction was executed in the presence of an excess amount of magnesium triflate and metallic sodium under reflux. Even under such extreme conditions, no traces of **2a** were detected. It is important to mention that under all of these controls, the starting material **1a** remained untouched.

### Reaction Scope

Once the reaction was optimized, the versatility of the transformation was explored (Figure 2). Monosubstituted alkenes were the first class of compounds tested for DMF distonic radical addition.

**Figure 2:**
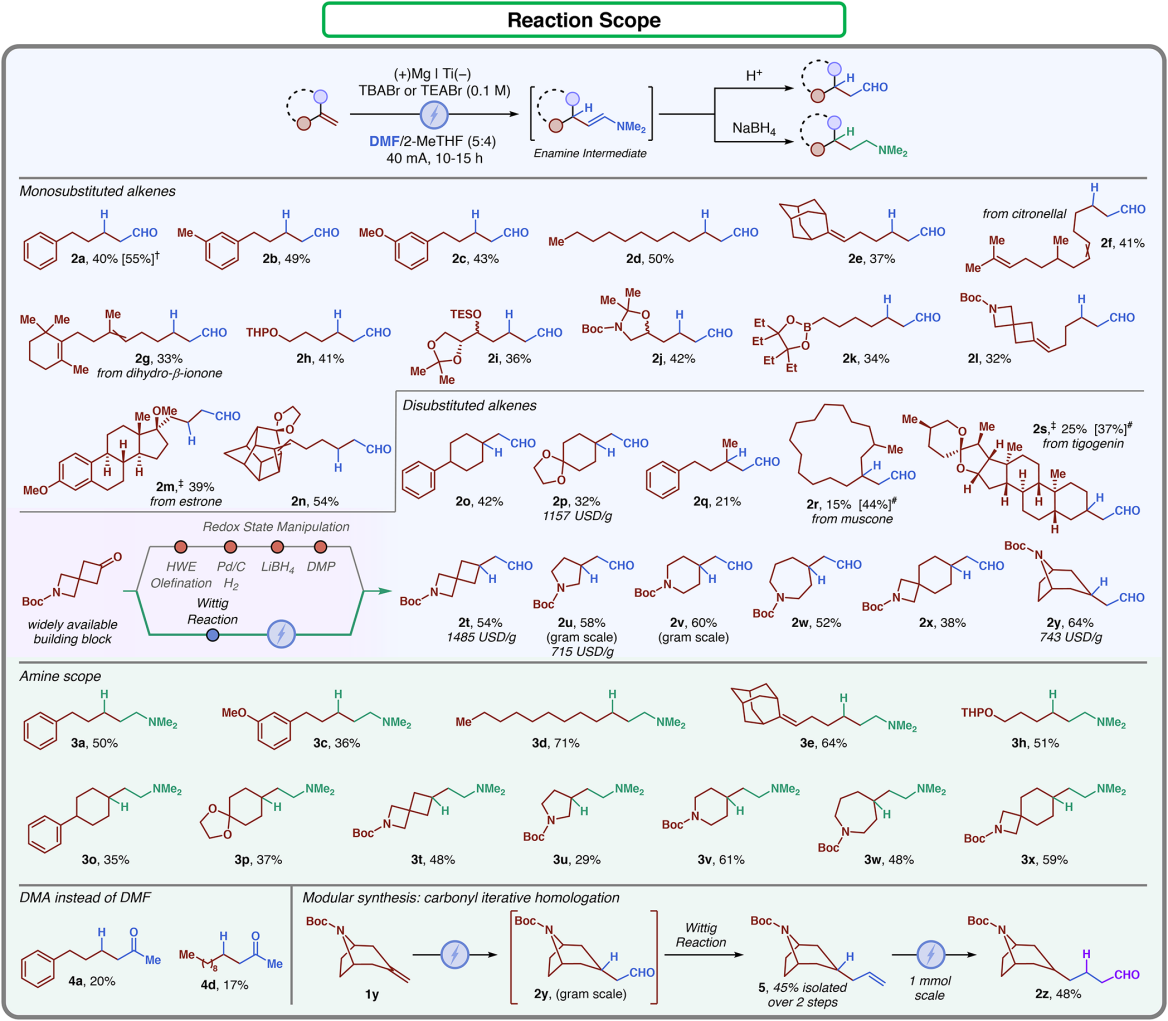
Reaction scope of electrochemical transformations. Standard conditions: alkene (1 mmol), TBABr or TEABr (0.1 M), DMF (5 mL), 2-MeTHF (4 mL), 40 mA, 10 h; Mg as the anode and Ti as the cathode. Acidic workup: 3.2 M aqueous HCl or 10% w/w citric acid. Reductive workup: after electrolysis, the addition of 10 mL of methanol and 5 equiv (5 mmol) of NaBH_4_. ^†^GC-FID yield (1,3,5-trimethoxybenzene as an internal standard). ^#^Based on recovered the starting material. ^‡^Reaction scale: 0.5 mmol. Gram scale: reaction scale conducted at 5 mmol scale. TBABr: tetrabutylammonium bromide; TEABr: tetraethylammonium bromide; THP: tetrahydropyranyl; TES: triethylsilyl; DMP: Dess–Martin periodinane.

Differently substituted 4-phenyl-1-butenes provided the desired hydroformylated products **2a**, **2b**, and **2c** with 40–50% isolated yields. 1-Undecene (**1d**) led to the formation of the desired product **2d** in 50% yield. Next, the chemoselectivity of the attack was probed by subjecting the adamantyl-substituted diene **1e**. In this case, only the selective terminal hydroformylated product **2e** was observed in 37% yield. To further confirm the exclusive selectivity toward terminal alkenes, the citronellal derivative **1f** and the dihydro-β-ionone derivative **1g** were employed. Indeed, the desired products **2f** and **2g** could be generated in 41 and 33% yield, respectively. Next, different functional groups were evaluated. Tetrahydropyranyl (THP)- and triethylsilyl (TES)-protected alcohols **1h** and **1i** were tolerated in the reaction. 1,2-Protected amino alcohols were also competent substrates for the transformation, as compound **2j** could be obtained in 42% yield. Epin boronate-containing substrate **1k** could be successfully employed as well (34% yield). N-Boc-protected spiro azetidine **1l** delivered the corresponding product in moderate yield (32%), with complete selectivity toward the terminal alkene. The protocol worked in an efficient manner even with more complex substrates, such as the estrone derivative **1m** or the polycyclic substrate **1n**, delivering the desired product in 39 and 54% yield, respectively. Subsequently, 1,1-disubstituted alkenes were investigated. Cyclohexylmethylene derivatives **1o** and **1p** delivered the corresponding aldehyde in 42 and 32% yield, respectively. Open-chain olefin **1q** and modified muscone **1r** provided the products in lower yields due to poor conversion caused by steric hindrance. Structure **1s** derived from phytosterol tigogenin could be successfully transformed into its corresponding hydroformylated product in 25% yield (37% based on the recovered starting material).

Owing to their importance in medicinal chemistry, the electrochemical hydroformylation of saturated heterocycles was tested next. The commonly used building block **2t** was obtained in 54% yield. Using this method, the synthetic route applied in the patent literature could be shortened from three tedious redox-state manipulation steps to a single electrochemical reaction.^23^ In this way, the preparation of this functional handle was accomplished in a more atom efficient and milder fashion. Pyrrolidine- (**1u**), piperidine- (**1v**), and azepane-containing alkenes (**1w**) provided the corresponding aldehydes in good yield (50–60%). Furthermore, compounds **2u** and **2v** were synthesized on a gram scale (5 mmol), demonstrating the possibility of implementing this accessible method in more complex synthetic routes. Similarly, the spirocycle **2x** was prepared in 38% yield, as well as the protected tropinone-derived compound **2y**. It is worth mentioning that many commonly used yet expensive medicinal chemistry bifunctional scaffolds such as **2p**,^24^ **2t**,^25^ **2u**,^26^ and **2y** ^27^ could be readily prepared via this approach. The assembly of the hydroformylation scope illustrates the synthetic capabilities of the DMF radical anion. However, the rationalized reaction design depicted in Figure 1C suggested that the process had more to unfold. Careful evaluation of the GC-MS analysis of the crude reaction mixture (i.e., before acidic workup) revealed the presence of the corresponding enamines.^28^ This experimental finding inspired the idea of attempting to further manipulate enamine instead of hydrolyzing it. Hence, the crude electrolysis mixture was subjected to a mild reductive workup.^29^ This two-step one-pot protocol led to the isolation of the *N,N*-dimethylamino hydromethylated product **3a** in 50% isolated yield. Notably, no traces of alcohol were observed at the end of the reduction. With this result in hand, expansion of this alternative workup was conducted. Monosubstituted alkenes **1c**, **1d**, **1e**, and **1h** all delivered the desired hydroaminated products. Furthermore, 1,1-disubstituted alkenes **1o**, **1p**, **1t**, **1u**, **1v**, **1w**, and **1x** were all successful. The implemented synthetic protocol mirrors the exquisite selectivity of the electrolysis step. After exploring the enamine reduction, we wondered whether the DMF reactivity could be expanded to other similar structures. For this reason, different amide solvents were tested. As a result, only *N*,*N*-dimethylacetamide (DMA) generated the methyl ketones **4a** and **4d** in modest yields (20 and 17% respectively), while more complex amides did not provide any desired product (see Supporting Information for a complete list). Finally, to prove the high modularity of our synthetic approach, the subsequent elongation of olefin **1y** was carried out. **2y** was obtained after gram-scale electrolysis (5 mmol) following the acidic workup. The crude product was then subjected to the Wittig reaction to obtain the elongated alkene **5**, leading to 45% isolated yield over 2 steps. This compound was then electrolyzed for a second time, providing the homologated compound **2z** in 48% yield. This synthetic route demonstrated the possibility to apply this reaction multiple times in conjunction with olefination protocols, offering an alternative robust route (22% over 3 steps).

### Reactivity Assessment

During the preparation of the reaction scope, several key correlations were identified, creating useful guidelines for the selection of competent substrates for the electrochemical process (Figure 3). By subjecting the methyl thioether-containing alkene **1aa** to electrolysis, the desired product was not obtained. Instead, reduction of the thioether moiety was observed (**1aa′**, 56%, Figure 3A). A similar outcome was observed for the cyano compound **1ab**, where the reduction of the nitrile occurred instead (**1ab**′****, 27%, Figure 3A). Intrigued by the results obtained, cyclic voltammograms of **1aa** and **1ab** were recorded to validate the redox activity. As expected, reductive peaks were observed at around −3.0 V vs the Ag/AgCl reference electrode. In comparison, benchmark alkene **1a** did not present any redox event within the operative potential window. The collected data indicated the importance of employing compounds that do not manifest reduction events within the DMF potential window. This preliminary test was confirmed by executing cyclic voltammetry (CV) analysis of different working substrates in the scope (see Supporting Information for a series of recorded analysis). Nevertheless, the redox potential is not the only parameter governing the success of the reaction. The sterically hindered olefin longifolene **1ac** did not show any reductive labile behavior according to CV analysis. However, the reaction provided only traces of desired product **2ac** even with prolonged electrolysis (Figure 3B). This experimental result underscored the impact of steric hindrance as an essential component for the success of the reaction.

**Figure 3:**
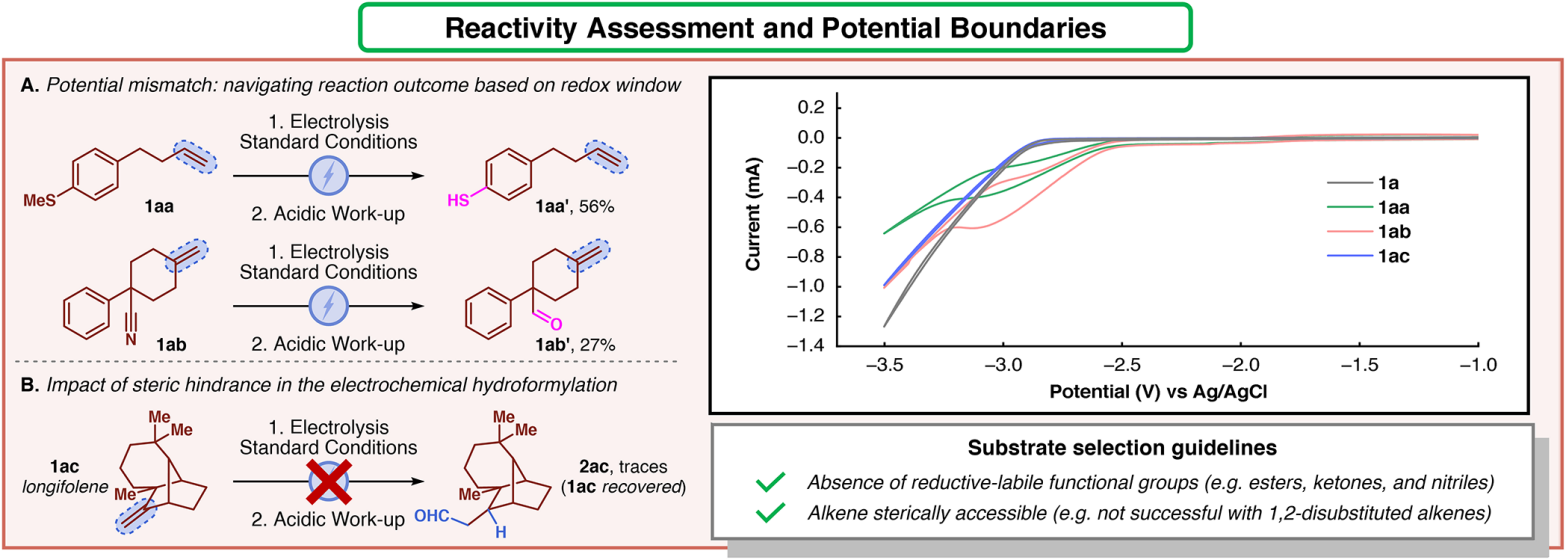
Reactivity assessment and potential boundaries: navigating and rationalizing the reaction scope. (A) Potential mismatch of substrate candidates. (B) Impact of steric hindrance on the success of the reaction. CV analysis of substrates **1a**, **1aa**, **1ab**, and **1ac** (DMF as solvent; WE: GC; CE: Pt; RE: Ag/AgCl).

### Mechanistic Investigation

Owing to the sporadic representation of this reactivity in the literature, a series of mechanistic experiments was conducted to elucidate the key steps of the reaction (Figure 4). The proposed mechanism starts with the DMF distonic radical anion generation. This intermediate is assumed to be stabilized by the surrounding solvent molecules as well as the presence of magnesium cations. This solvated ion pair is supposed to tune the nature of such a reactive species.^30^ Subsequently, reversible radical addition to the alkene occurs, generating the corresponding carbon-centered radical.^31^ Previously, Shkrob and Marin proposed that the distonic radical anion is stabilized via solvation, hindering the engagement capabilities of the activated species.^16^ This effect seems to be confirmed by the steric sensitivity observed with multisubstituted olefins. Next, the newly generated carbon-centered radical is promptly quenched by another molecule of the amide solvent. As mentioned before, the HAT event can happen at two different sites due to the small difference in the BDE, namely, at the acyl moiety (BDE = 89.7 kcal/mol) or at the dimethylamine moiety (BDE = 89.1 kcal/mol).^7^ Finally, the resulting hemiaminal anion is in equilibrium with its enamine form. Notably, both tautomers seem to be stable during electrolysis, guaranteeing the accumulation of the product. The first step of the proposed mechanism was initially investigated by executing a competitive kinetic isotopic experiment (Figure 4A). By subjecting **1a** to electrolysis in the presence of a 1:1 mixture of DMF-*h*_7_ and DMF-*d*_7_, the isolated product revealed a secondary kinetic isotopic effect (KIE = 1.14), indicating that DMF is not activated by a HAT event (see Supporting Information for further details). In addition, the electrolysis was carried out in the absence of the alkene, and 2,4-dinitrophenyl hydrazine (DNP) was then added to the crude. Two different hydrazones could be isolated following this protocol: **6a**, derived from glyoxal, and **6b**, derived from *N*,*N*-dimethylglyoxylamide. The identification of these molecules indicated the presence of the DMF radical anion dimer and the coupling between the aforementioned radical and the formyl HAT radical. These tests demonstrated that the reaction passes via the formation of the elusive DMF radical anion (**Int. A**, Figure 4), precluding an HAT activation pathway. Subsequently, a ‘radical clock’ experiment employing β-caryophyllene **1ad** was carried out to prove the radical attack on the alkene (Figure 4B). Counterintuitively, the expected β,γ-unsaturated aldehyde **2ad** was not observed. Instead, molecule **2ad**′**** was obtained in 15% yield. Compound **2ad**′**** presented two peculiar characteristics: (i) the strained cyclobutyl ring was opened and (ii) an α,β-unsaturated aldehyde was present. The unconventional product for this methodology has several mechanistic implications. First, the reaction appears to be radically promoted, as multiple papers have reported ‘radical clock’ opening of similar skeletons.^32,33^ Second, the presence of the conjugated aldehyde moiety unequivocally derived from the conjugated hydrolysis of the corresponding enamine. Notably, such an outcome was not observed when traditional rhodium-catalyzed hydroformylations were conducted with similar scaffolds.^34^ This experiment confirmed that carbon monoxide insertion mechanisms were not involved. Thus, radical addition seems to be the most plausible pathway. Next, to investigate the HAT event, a deuterium labeling test was carried out (Figure 4C). By subjecting olefin **1a** to electrolysis in the presence of pure DMF-*d*_7_, the 1,3-dideuterated compound **2a-** *d*_**2**_ was obtained in 41% yield. NMR analysis revealed a high deuteration rate at both positions (>99%), indicating not only that the HAT quenching was solely performed by DMF but also that the aldehyde carbon traces back to the acyl carbon of formamide. Experiments involving a mixture of DMF-*d*_7_ and DMF-*d*_1_ revealed that all of the C–H positions of the solvent can contribute to this step (see Supporting Information for further details). Finally, the formation of the enamine as a product of the electrochemical transformation was verified via the isolation of several amines after the NaBH_4_ reductive workup (Figure 4D). Upon defining the key steps of the transformation, the last fundamental part to elucidate was the SET event that generated the reactive radical species. Perichon and co-workers proposed that the magnesium electrode corrosion was the responsible phenomenon for the formation of the DMF distonic radical.^15^ Their observation provided the unconventional hypothesis that the SET reduction event was promoted by the freshly exposed Mg^0^ surface at the sacrificial anode. To further confirm this conjecture, several tests were performed (Figure 4E). First, the effect of DMF on the magnesium electrode was studied. CV analysis in the presence of magnesium as the working electrode showed a steady increment of current response (magnesium dissolution) when scanning from negative to positive potentials.^35^ A similar, yet attenuated, response could be observed when DMA was employed. Interestingly, repeating the analysis with other coordinating solvents such as 2-MeTHF and pyridine did not provide the same current response (Figure 4E, CV analysis). This phenomenon has been widely reported in the literature, and it indicated that the first layer at the surface of the electrode was stripped and dissolved in polar aprotic solvents.^35^ This effect exposed the reactive metallic magnesium, which triggers the chemical reduction of DMF, confirming the experimental evidence of overconsumption of magnesium previously observed.^15^ In comparison with the thermochemical control, the electrochemical process guarantees the continuous availability of reactive Mg^0^, circumventing complete inhibition of the metal surface. It is important to mention that zinc and aluminum sacrificial electrodes do not possess sufficient chemical reductive potential to accomplish the same task (Figure 4E, entry 2). To further confirm this theory, a control electrolysis using zinc as the sacrificial anode in the presence of Mg­(OTf)_2_ was carried out.^36^ The reaction did not lead to any formation of product **2a**. Therefore, even if Mg^0^ would be produced cathodically, it was not competent in promoting the hydroformylation reaction (Figure 4E, entry 3). This control, in combination with the ineffectiveness of other amides probed, indicates that no organomagnesium species was involved in the mechanism.^37^ Subsequently, a three-electrode system (Mg or Zn, Ti, and Ag/AgCl as the reference electrode) was employed to explore different controlled-potential electrolysis. By setting titanium as the working electrode at −4.0 V (vs Ag/AgCl, a sufficient potential to promote the cathodic reduction of DMF) with Zn as the counter electrode (CE), no product was observed (Figure 4E, entry 4). Conversely, by setting magnesium as the working electrode at +1.2 V (vs Ag/AgCl, a sufficient potential to promote the Mg dissolution event according to the CV analysis), product **2a** was observed in a yield similar to that in the constant current one (Figure 4E, entry 5).

**Figure 4:**
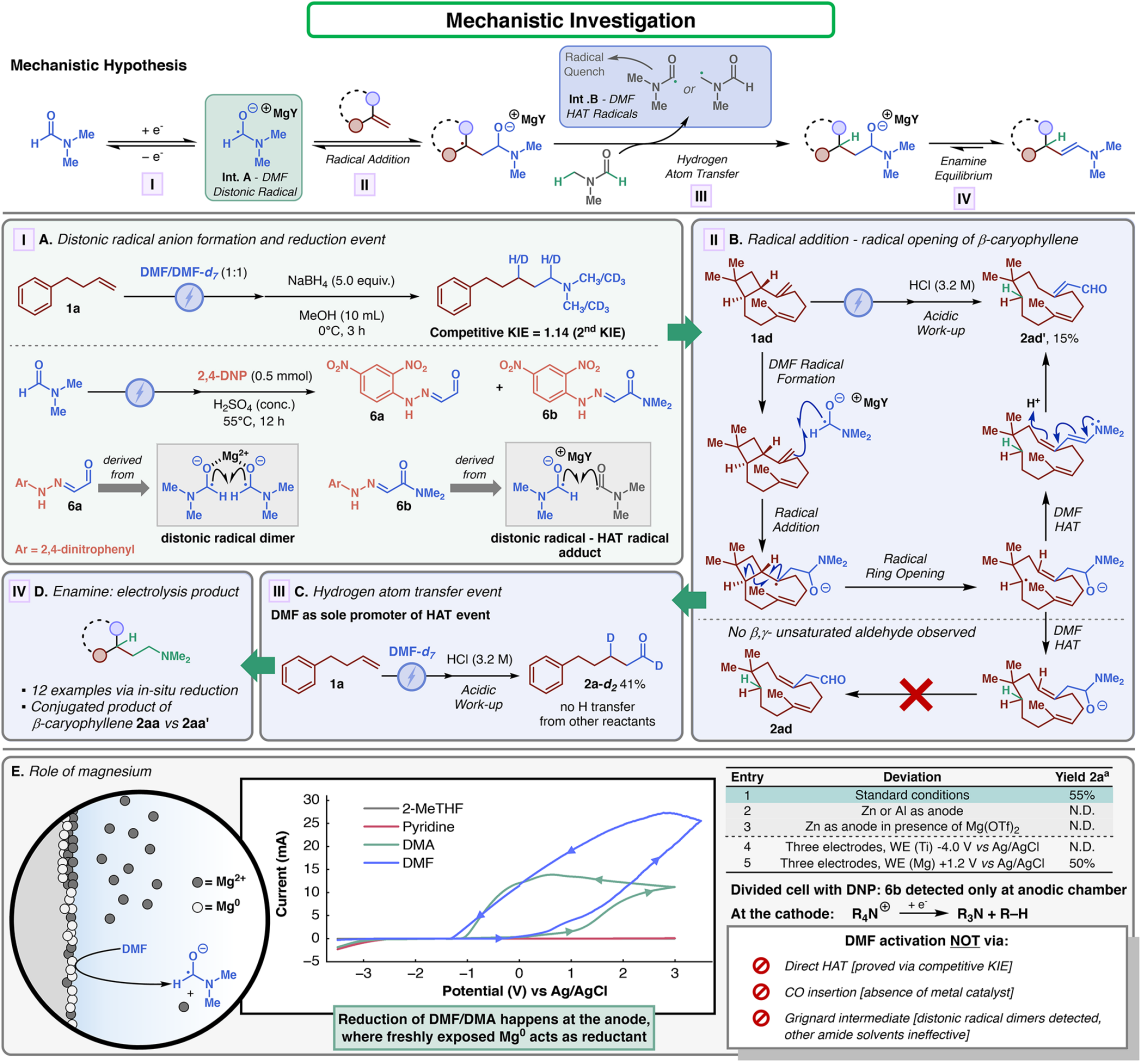
Mechanistic investigation: hypothesis and experimental observations. (A) Step I, Demonstration of distonic radical formation: Competitive kinetic isotope experiment: 2,4-dinitrophenylhydrazine (2,4-DNP) capture of generated radicals. (B) Step II, Radical addition: reaction with the β-caryophyllene radical clock (ring opening). (C) Step III, HAT: deuterated product formation. (D) Step IV, Enamine formation. (E) Role of magnesium: CV analysis of magnesium dissolution in different solvents (WE: Mg: CE: Ti; RE: Ag/AgCl). For all experimental procedures, please see Supporting Information. ^a^GC-FID yield (1,3,5-trimethoxybenzene as the internal standard).

Furthermore, divided cell experiments confirmed the presence of **6b** only at the anode, corroborating the hypothesis of an anodically generated distonic radical. Finally, the experimental observation of tributylamine or triethylamine in crude mixtures via GC and NMR analyses indicates the decomposition of the supporting electrolyte at the cathode.^17,38^

## Conclusions

In conclusion, the electrolysis-assisted generation of DMF as the radical anion is presented. This work confirms the existence of an elusive species that was theorized for 35 years, broadening the radical reactivity portfolio. This intermediate has been synthetically employed for the first time via addition to unactivated alkenes. This hydroformylation was optimized and applied to different molecules, showcasing an exquisite regio- and chemoselectivity toward terminal positions. Furthermore, *N,N*-dimethylamino hydromethylated products could be readily prepared by applying a reductive workup at the end of the electrolysis. The intriguing mechanism of this transformation was unfolded with several experiments and analyses, demonstrating the radical nature of the process. We believe that this electrolysis-assisted functionalization will provide a new perspective to the activation of this commonly used solvent, paving the way to other possible radical transformations.

## Supplementary Material



## Data Availability

The data underlying this study are available in the published article and its Supporting Information.

## Notes

Additional experimental details, materials, methods, and characterization data (PDF)
