# Supplementary material for: Electrolysis-Assisted Reduction of Dimethylformamide for Unactivated Alkene Functionalizations
Source: J Am Chem Soc. 2026 Mar 2;148(9):9367–74. doi: 10.1021/jacs.5c17824 (PMC12983302; doi:10.1021/jacs.5c17824)

Electrolysis-Assisted Reduction of Dimethylformamide for Unactivated Alkene Functionalizations

Yifan Xi[a†], Sulekha Sharma[a†], C. Oliver Kappe[a,b], Gabriele Laudadio[a,b*]

[a]Institute of Chemistry, NAWI Graz, University of Graz, Heinrichstrasse 28, 8010 Graz, Austria.
[b]Center for Continuous Flow Synthesis and Processing (CCFLOW) Research Center Pharmaceutical Engineering GmbH (RCPE), Inffeldgasse 13, 8010 Graz, Austria.

[†]Indicates equal contribution.

Correspondence to *gabriele.laudadio@uni-graz.at

Supplementary Materials

Table of contents

1. General Experimental Information 2

2. Reaction optimization 3

3. Synthetic Procedures and Characterizations 7

3.1 Starting materials synthesis 7

3.2 Graphical guide 30

3.3 Troubleshooting & FAQ 39

3.4 General procedure 1: Electrochemical hydroformylation 41

3.5 General procedure 2: Electrochemical amine synthesis 67

3.6 Electrochemical ketone synthesis using DMA as solvent. 79

3.7 Aldehyde Homologation 81

4. Mechanistic studies 83

4.1. Competitive KIE experiment 83

4.2. 2,4-Dinitrophenylhydrazine capturing experiment 85

4.3. Radical clock experiment 87

4.4. Deuteration experiment 88

4.5. HAT probe experiment 89

4.6. Control experiments 91

4.7. CV analysis 93

5. Unexpected products and limitations 101

5.1 Unexpected products 101

5.2. Limitations 103

6. References 104

7. NMR data 106

1. General Experimental Information

Solvents and chemicals were obtained from typical commercial vendors and were used as received, without any further purification.

NMR analysis was recorded on a Bruker Avance III 300 MHz instrument at room temperature, in $CDCl_3$ as a solvent, at 300 MHz and 75 MHz, respectively. Chemical shifts (δ) are reported in ppm relative to the residual solvent peak ($CDCl_3$, ^1H: 7.26 ppm, ^{13}C: 77.16 ppm; DMSO-d_6 ^1H NMR δ = 2.50 ppm, ^{13}C NMR δ = 39.52 ppm). Coupling constants are reported in Hertz. Multiplicity is reported with the usual abbreviations. Structural assignments were made with additional information from DEPT-135, COSY, HSQC, HMBC and NOESY experiments.

GC analysis was performed on a Shimadzu GC FID 230 with a flame ionization detector (FID), using an RTX-5MS Cap. column (30 m × 0.25 mm ID × 0.25 μm) and helium as carrier gas (40 cm/sec-1 linear velocity). The injector temperature was set to 280 °C. After 1 min at 50 °C, the temperature was increased by 25 °C/min to 300 °C and kept constant at 300 °C for 4 min. FID was used for detection, and the detector gases used for flame ionization were hydrogen and synthetic air (5.0 quality)

GC-MS analysis was performed using a Shimadzu GCMS-QP2010 SE, using an RTX-5MS column (30 m × 0.25 mm × 0.25 μm) and helium as carrier gas (40 cm/sec linear velocity). The injector temperature was set to 280 °C. After 1 min at 50 °C, the oven temperature was increased by 25 °C/min to 300 °C and then kept at 300 °C for 3 min. The mass detector was a quadrupole with pre-rods and electron impact ionization. The following settings were used in the detector: ion source temperature 200 °C, interface temperature 310 °C, solvent cut time 2 min 30 sec, acquisition mode scan, mass range m/z = 50 till m/z = 400.

High Resolution Mass Spectrometry were recorded in either negative or positive mode on an Agilent 6230 TOF LC/MS (G6230B) by flow injections on an Agilent 1260 Infinity Series HPLC (HiP Degasser G4225A, Binary Pump G1312B, ALS Autosampler G1329B, TCC Column thermostat G1316A, DAD Detector G4212B).

Automated flash column chromatography was performed on a Biotage Isolera system using Biotage® Sfär Silica D as packed column. Eluent was petroleum spirit 40-60 with an increasing gradient of ethyl acetate.

Analytical thin-layer chromatography (TLC) was carried out using Merck silica gel 60 GF254 plates. Compounds were visualized through UV or $KMnO_4$ or DNP. Eluent was the same as used for flash column chromatography, or it is stated otherwise.

2. Reaction optimization

After several preliminary experiments and tests for workup, we began our optimization with screening of electrodes. The reaction setup was identical to the optimized condition except a 5 mL IKA vial was used. The crude reaction mixtures were first quenched by HCl (3.2 M) and extracted with Et_2O (due to product volatility) before adding internal standard (1,3,5-Trimethoxybenzene, 0.2 mmol, 33.6 mg) and analyzed by GC-FID.

Table S1. Electrode Screening

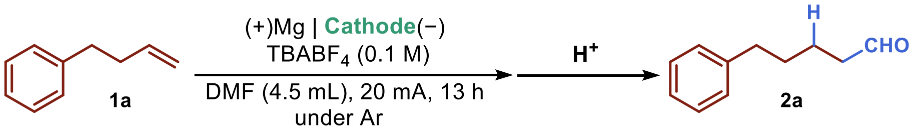

Entry	Deviation from above	Remaining S.M.	Yield 2a
1	Pt as cathode	47	8
2	C_{gr} as cathode Zn as Anode	58	N.D.
3	C_{gr} as cathode Al as Anode	72	N.D.
4	Stainless steel as cathode	47	6
5	Pb as cathode	32	7
6	Mg as cathode	30	4
6	(-) Mg|Mg (+), AC (1 Hz)	18	31
7	Ni as cathode	37	16
8	GC as cathode	29	22
9	RVC as cathode	12	26
10	Ni-foam as cathode	18	31
11	Sn as cathode	7	38
12	Ti as cathode	17	36
13	Ti as cathode Pt as Anode<br>0.1 mL Et_3N as additive	No reaction detected	

Takeaway: The reaction is only working when magnesium is used as anode. However different materials show similar reactivity when used as cathode. Alternating current (AC) electrolysis suffers from severe magnesium powder deposition.

Table S2.1. Additive Screening

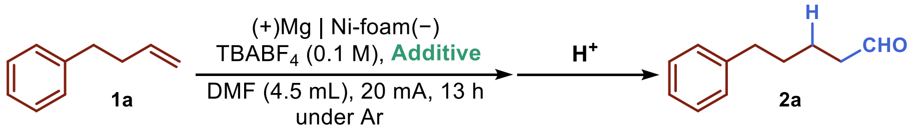

Entry	Deviation from above	Remaining S.M.	Yield 2a
1	No additive	18	31
2	TEOA (0.20 equiv.)	6	35
3	y-Terpinene (0.20 equiv.)	11	31
4	TRIPS (0.20 equiv.)	20	28
5	Ph_3SiH (0.20 equiv.)	10	32
6	Benzoquinone (0.20 equiv.)	17	25
7	Ferrocene (0.20 equiv.)	5	mixture
8	TMG (0.20 equiv.)	17	34
9	DBU (0.20 equiv.)	47	4
10	Pyridine	58	trace
11	HFIP (0.20 equiv.)	88	trace
12	Silica gel (50 mg)	58	trace

Takeaway: We found HAT donors, electron transfer mediators, bases and acids are not improving the reaction. This also indicates that the addition of sacrificial chemicals does not lead to reaction improvement, rather decreasing the efficiency of the transformation.

Table S2.2 Evaluation of sacrificial compounds on functional group sensitive substrate **1ab**.

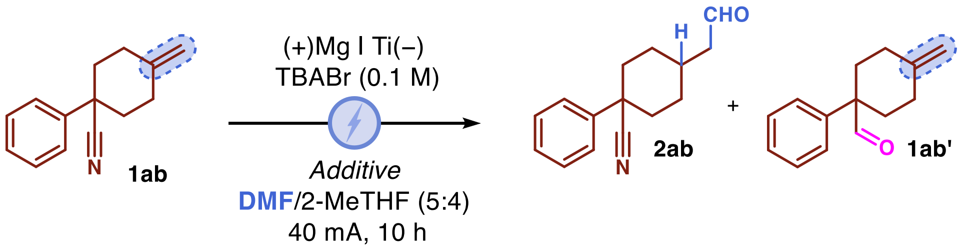

Additive	2ab	1ab'	1ab
HFIP (0.2 equiv.)	**N.D.**	N.D.	Recovered
HFIP (1.5 equiv.)	**N.D.**	33%	N.D.
Benzoquinone (0.2 equiv.)	**N.D.**	Traces	Recovered
Benzoquinone (1.5 equiv.)	**N.D.**	N.D.	54%

Takeaway: As surveyed in Table S2.1, the addition of sacrificial chemicals in substoichiometric or excess amounts did not provide the desired product, indicating that the mitigation of the cathodic reaction did not improve reaction selectivity.

Table S3. Solvent Screening

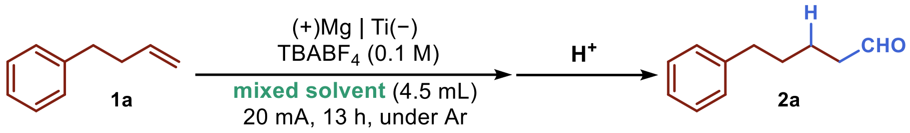

Entry	Deviation from above	Remaining S.M.	Yield 2a
1	DMF:DCM = 4:0.5	73	N.D.
2	DMF:DCM = 0.5:4	70	N.D.
3	DMF:DME = 2:2.5	15	38
4	DMF:1,4-dioxane = 2:2.5	58	36
5	DMF:THF = 2:2.5	15	38
6	DMF:MeTHF = 2:2.5	17	43
7	DMF:MeTHF = 3:1.5	47	41
8	DMF:MeTHF =1.5:3	32	23

Takeaway: The role of ether solvents could link to the solubility of magnesium salts thus help removing precipitates generated at anode.

Table S4. Supporting Electrolyte Screening

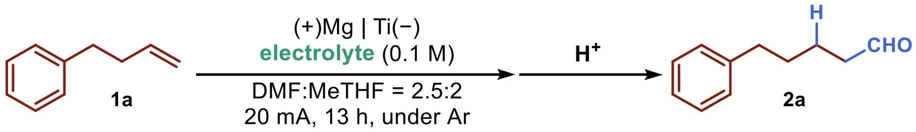

Entry	Deviation from above	Remaining S.M.	Yield 2a
1	LiBr as electrolyte	90	N.D.
2	$LiClO_4$ as electrolyte	45	26
3	$TBABF_4$ as electrolyte	19	35
4	TBACl as electrolyte	19	34
5	TBABr as electrolyte	trace	45
6	TBAI as electrolyte	trace	44

Takeaway: Tetrabutylammonium salts are suitable to the reaction due to their electrochemical decomposition to the corresponding amines which balances the cathodic reaction.

Final takeaways:

1. In the optimized condition, we choose to double the solvent volume based on the fact that the reaction is not sensitive towards concentration of starting material. Adding more solvents helps reducing the precipitation of magnesium salts during the reaction, thus stabilizing the batch voltage.
2. Another major difference is we choose to use 40 mA instead of 20 mA based on repeatability, this reaction can work even under 10 mA but increasing the current to 40 mA makes the reaction much more repeatable.

3. Synthetic Procedures and Characterizations

3.1 Starting materials synthesis

Compound 1b

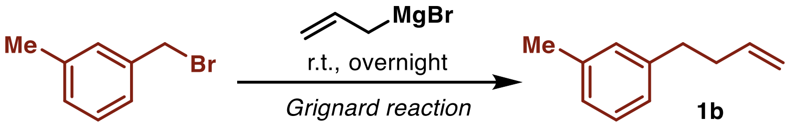

Preparation executed according to literature procedure.[1] To a stirred solution of alkyl bromide (10.0 mmol, 2.47 g) in anhydrous THF (20 mL) at 0 °C, allylmagnesium bromide (1.50 equiv., 15.0 mmol, 1.0 M in Et_2O, 15 mL) was added via syringe under argon. Upon addition the mixture was allowed to raise to room temperature and stirred overnight was then quenched with saturated NH_4Cl and extracted 3 times with Et_2O, the organic layers were combined and washed again with brine, dried over Na_2SO_4 then concentrated via rotavap and purified via silica gel column chromatography (eluent: petroleum ether, stained with $KMnO_4$) to afford the desired olefin **1b**.

Physical Appearance: colorless oil

^{1}H NMR (300 MHz, $CDCl_3$): δ 7.13 – 7.06 (m, 1H), 6.97 – 6.88 (m, 3H), 5.90 – 5.69 (m, 1H), 5.16 – 4.83 (m, 2H), 2.67 – 2.54 (m, 2H), 2.35 – 2.23 (m, 5H).

^{13}C NMR (75 MHz, $CDCl_3$): δ 142.0, 138.4, 138.0, 129.4, 128.3, 126.7, 125.5, 114.9, 35.7, 35.5, 21.6.

Characterization data for this olefin was in agreement with the literature.[1]

Compound 1c

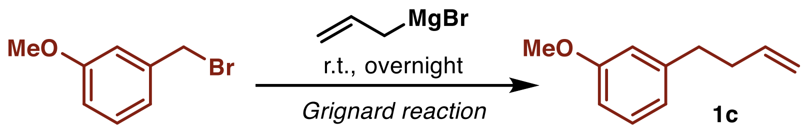

Preparation executed according to literature procedure.[1] To a stirred solution of alkyl bromide (9.95 mmol, 2.00 g) in anhydrous THF (20 mL) at 0 °C, allylmagnesium bromide (2.00 equiv., 20.00 mmol, 1.0 M in Et_2O, 20 mL) was added via syringe under argon. Upon addition the mixture was allowed to raise to room temperature and stirred overnight. The mixture was then quenched with saturated NH_4Cl and extracted 3 times with Et_2O, the organic layers were combined and washed again with brine, dried over Na_2SO_4 and concentrated via rotavap then purified via silica gel column chromatography (eluent: petroleum ether, stained with $KMnO_4$) to afford the desired olefin **1c**.

Physical Appearance: colorless oil

^{1}H NMR (300 MHz, $CDCl_3$): δ 7.16 – 7.07 (m, 1H), 6.81 – 6.54 (m, 3H), 6.01 – 5.61 (m, 1H), 5.04 – 4.86 (m, 2H), 3.71 (s, 3H), 2.66 – 2.55 (m, 2H), 2.34 – 2.23 (m, 2H).

^{13}C NMR (75 MHz, $CDCl_3$): δ 159.7, 143.6, 138.2, 129.4, 121.0, 115.0, 114.3, 111.2, 55.2, 35.5, 35.5.

Characterization data for this olefin was in agreement with the literature.[2]

Compound 1e

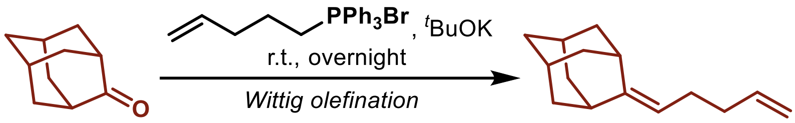

To a vigorously stirred suspension of Wittig salt (1.50 equiv., 19.97 mmol, 8.21 g) in anhydrous THF (20 mL), *t*BuOK (1.50 equiv., 19.97 mmol, 2.24 g) was added in one portion, the mixture was then allowed to stir for 15 minutes then the ketone (13.31 mmol, 2.00 g) was added in one portion and stirred overnight, The mixture was then quenched with saturated NH_4Cl and extracted 3 times with Et_2O, the organic layers were combined and washed again with brine, dried over Na_2SO_4 and concentrated via rotavap then purified via silica gel column chromatography (eluent: petroleum ether, stained with $KMnO_4$) to afford the desired olefin **1e**.

Physical Appearance: colorless oil

^{1}H NMR (300 MHz, $CDCl_3$): δ 5.92 – 5.76 (m, 1H), 5.09 – 4.90 (m, 3H), 2.83 – 2.77 (m, 1H), 2.35 – 2.29 (m, 1H), 2.18 – 1.99 (m, 4H), 1.99 – 1.60 (m, 12H).

^{13}C NMR (75 MHz, $CDCl_3$): δ 148.1, 139.0, 115.5, 114.4, 40.7, 40.0, 39.1, 37.5, 34.7, 32.2, 28.8, 26.2.

Compound 1f

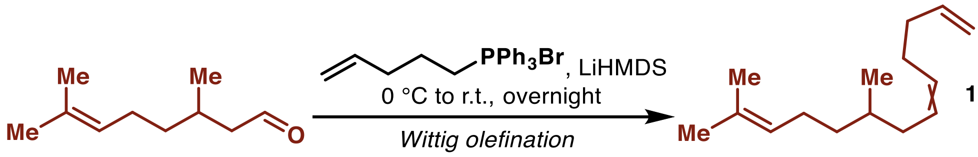

To a vigorously stirred suspension of Wittig salt (1.50 equiv., 19.45 mmol, 8.00 g) in anhydrous THF (50 mL), LiHMDS (2.00 equiv., 25.93 mmol, 1.0 M in THF, 26 mL) was added dropwise under argon at 0 °C, the mixture was then allowed to raise to room temperature and stir for 15 minutes then the ketone (12.97 mmol, 2.00 g) was added and stirred overnight, The mixture was then quenched with saturated NH_4Cl and extracted 3 times with Et_2O, the organic layers were combined and washed again with brine, dried over Na_2SO_4 and concentrated via rotavap then purified via silica gel column chromatography (eluent: petroleum ether, stained with $KMnO_4$) to afford the desired olefin **1f** as a mixture of *Z* and *E* isomers.

Physical Appearance: colorless oil

^{1}H NMR (300 MHz, CDCl$_3$): δ 5.93 – 5.73 (m, 1H), 5.47 – 5.32 (m, 2H), 5.16 – 4.91 (m, 3H), 2.20 – 2.06 (m, 4H), 2.06 – 1.80 (m, 4H), 1.69 (s, 3H), 1.61 (s, 3H), 1.57 – 1.24 (m, 2H), 1.24 – 1.03 (m, 1H), 0.88 (d, *J* = 6.5 Hz, 3H).

^{13}C NMR (75 MHz, CDCl$_3$): δ 138.7, 131.2, 129.8, 129.1, 125.0, 114.7, 36.9, 34.6, 34.0, 33.2, 26.9, 25.9, 25.8, 19.7, 17.8.z

HRMS (ESI-TOF) m/z: [M + MeOH + H]$^+$ Calcd for $C_{16}H_{31}O$ 239.2369; Found 239.2354.

Compound 1g

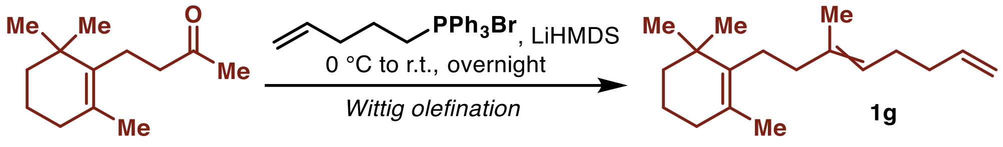

To a vigorously stirred suspension of Wittig salt (1.50 equiv., 11.58 mmol, 4.76 g) in anhydrous THF (20 mL) at 0 °C, LiHMDS (1.50 equiv., 1.0 M in Et_2O, 11.58 mmol, 12 mL) was added via syringe, the mixture was then allowed raise to room temperature and stir for 15 minutes then the ketone (7.72 mmol, 1.50 g) was added in one portion and stirred overnight, The mixture was then quenched with saturated NH_4Cl and extracted 3 times with Et_2O, the organic layers were combined and washed again with brine, dried over Na_2SO_4 and concentrated via rotavap then purified via silica gel column chromatography (eluent: petroleum ether, stained with $KMnO_4$) to afford the desired olefin **1g** as a mixture of *Z* and *E* isomers.

Physical Appearance: colorless oil

^{1}H NMR (300 MHz, $CDCl_3$): δ 5.93 – 5.75 (m, 1H), 5.21 – 4.91 (m, 3H), 2.18 – 1.96 (m, 8H), 1.96 – 1.86 (m, 2H), 1.79 – 1.72 (m, 1H), 1.71 – 1.51 (m, 7H), 1.48 – 1.38 (m, 2H), 1.10 – 0.97 (m, 6H).

^{13}C NMR (75 MHz, $CDCl_3$): δ 138.9, 138.8, 137.3, 137.3, 136.6, 136.6, 127.3, 127.1, 124.2, 123.3, 114.6, 114.5, 40.5, 40.0, 35.1, 35.1, 34.5, 34.2, 33.0, 32.9, 32.8, 28.8, 28.8, 28.1, 27.6, 27.6, 27.6, 27.5, 23.5, 20.0, 20.0, 19.7, 16.2.

Compound 1h

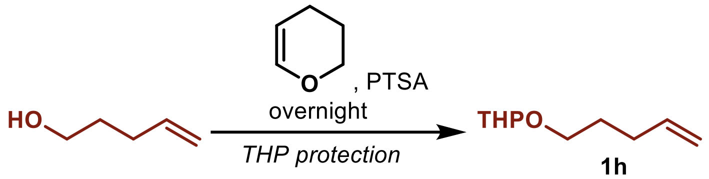

To a mixture of DHP (0.98 equiv., 22.76 mmol, 1.91 g) and PTSA (0.05 equiv., 1.16 mmol, 200 mg) in DCM (40 mL) at 0 °C, the alcohol (23.22 mmol, 2.00 g) was added, upon addition the mixture was allowed to raise to room temperature and stirred overnight and quenched with saturated $NaHCO_3$ then DCM (50 mL) was added to the mixture and the organic layer was washed with water and brine, dried over Na_2SO_4, concentrated via rotavap then purified via silica gel column chromatography (eluent: petroleum ether/ethyl acetate = 9/1, stained with $KMnO_4$) to afford the olefin **1h**.

Physical Appearance: colorless oil

^{1}H NMR (300 MHz, $CDCl_3$): δ 5.94 – 5.73 (m, 1H), 5.08 – 4.90 (m, 2H), 4.62 – 4.51 (m, 1H), 3.99 – 3.67 (m, 2H), 3.56 – 3.29 (m, 2H), 2.21 – 2.07 (m, 2H), 1.89 – 1.76 (m, 1H), 1.76 – 1.64 (m, 3H), 1.63 – 1.45 (m, 4H).

^{13}C NMR (75 MHz, $CDCl_3$): δ 138.5, 114.8, 99.0, 67.1, 62.4, 30.9, 30.5, 29.1, 25.6, 19.8.

HRMS (ESI-TOF) m/z: $[2M + Na]^+$ Calcd for $C_{20}H_{36}NNaO_4$ 363.2506; Found 363.2475.

Compound 1i

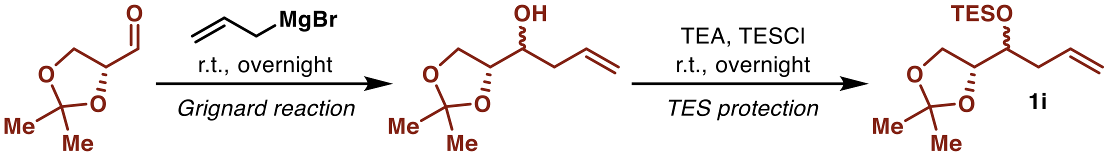

To a stirred solution of aldehyde (15.37 mmol, 2.00g) in anhydrous THF (15 mL) at 0 °C, allylmagnesium bromide (1.20 equiv., 18.44 mmol, 1.0 M in Et_2O, 18 mL) was added via syringe under argon. Upon addition the mixture was allowed to raise to room temperature and stirred overnight. The mixture was then quenched with saturated NH_4Cl and extracted 3 times with Et_2O, the organic layers were combined and washed again with brine, dried over Na_2SO_4 and concentrated via rotavap, the crude alcohol was used without further purification.

To a stirred mixture of aforementioned alcohol (13.77 mmol, 2.37 g) in DCM (120 mL), was added TEA (1.20 equiv., 16.52 mmol, 1.67 g) and TESCl (1.20 equiv., 16.52 mmol, 2.49 g) and the mixture was stirred overnight and quenched with saturated NH_4Cl, the mixture was extracted 3 times with DCM and the organic layers were combined and washed with brine, dried over Na_2SO_4, and concentrated via rotavap, then purified via silica gel column chromatography (eluent: petroleum ether/ethyl acetate = 10/1, stained with $KMnO_4$) to afford the desired olefin **1i** as a mixture of diastereomers.

Physical Appearance: colorless oil

^{1}H NMR (300 MHz, CDCl$_3$): δ 5.94 – 5.76 (m, 1H), 5.14 – 4.99 (m, 2H), 4.10 – 3.62 (m, 4H), 2.35 – 2.04 (m, 2H), 1.43 – 1.29 (m, 6H), 1.01 – 0.87 (m, 9H), 0.68 – 0.53 (m, 6H).

^{13}C NMR (75 MHz, CDCl$_3$): δ ^{13}C NMR (75 MHz, $CDCl_3$) δ 135.0, 134.3, 117.6, 117.2, 109.2, 109.0, 78.7, 78.1, 73.2, 72.5, 66.3, 65.7, 39.3, 37.9, 26.8, 26.5, 25.6, 25.4, 7.0, 7.0, 5.3, 5.2.

HRMS (ESI-TOF) m/z: $[M + Na]^+$ Calcd for $C_{15}H_{30}NaO_3Si$ 309.1856; Found 309.1863.

Compound 1j

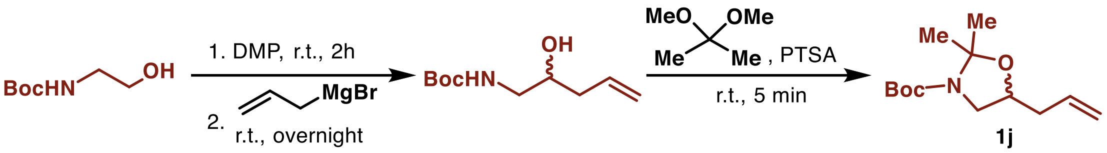

To a stirred solution of alcohol (20.00 mmol, 3.22 g) in DCM (50 mL) at 0 °C was added Dess-Martin periodinane (1.05 equiv., 21.00 mmol, 8.91 g), the mixture was allowed to stir for 2 hours at room temperature and quenched by a 1:1 mixture of saturated $Na_2S_2O_3$ and $NaHCO_3$, the solution was extracted with DCM for 3 times and the organic layers were combined and washed with brine and dried over Na_2SO_4 then concentrated via rotavap, the crude aldehyde was used without further purification.

To a stirred solution of aforementioned aldehyde (20.00 mmol, 3.18g) in anhydrous THF (50 mL) at 0 °C, allylmagnesium bromide (1.50 equiv., 30.00 mmol, 1.0 M in Et_2O, 30 mL) was added via syringe under argon. Upon addition the mixture was allowed to raise to room temperature and stirred overnight. The mixture was then quenched with saturated NH_4Cl and extracted 3 times with Et_2O, the organic layers were combined and washed again with brine, dried over Na_2SO_4 and concentrated via rotavap, then purified via silica gel column chromatography (eluent: petroleum ether/ethyl acetate = 1.5/1, stained with $KMnO_4$) to afford the alcohol.

To a stirred solution of aforementioned alcohol (3.04 mmol, 611 mg) in acetone (24 mL), was added 2,2-dimethoxypropane (6 mL) and PTSA (0.10 equiv., 0.30 mmol, 53 mg) and the mixture was stirred for 5 min and quenched with saturated $NaHCO_3$, the mixture was extracted 3 times with DCM, the organic layers were combined and washed again with brine, dried over Na_2SO_4 and concentrated via rotavap, then purified via silica gel column chromatography (eluent: petroleum ether/ethyl acetate = 9/1, stained with $KMnO_4$) to afford the desired olefin **1j** as a mixture of diastereomer.

Physical Appearance: colorless oil

^{1}H NMR (300 MHz, $CDCl_3$): δ 5.88 – 5.69 (m, 1H), 5.19 – 5.04 (m, 2H), 4.17 – 4.02 (m, 1H), 3.70 – 3.56 (m, 1H), 3.15 – 3.02 (m, 1H), 2.64 – 2.20 (m, 2H), 1.64 – 1.34 (m, 15H).

^{13}C NMR (75 MHz, $CDCl_3$): δ 152.4, 152.1, 133.4, 117.9, 93.7, 93.2, 80.2, 79.5, 73.2, 73.0, 50.6, 37.6, 28.6, 27.4, 26.3, 25.3, 24.4.

HRMS (ESI-TOF) m/z: $[M + Na]^+$ Calcd for $C_{13}H_{23}NNaO_3$ 264.1570; Found 264.1573.

Compound 1k

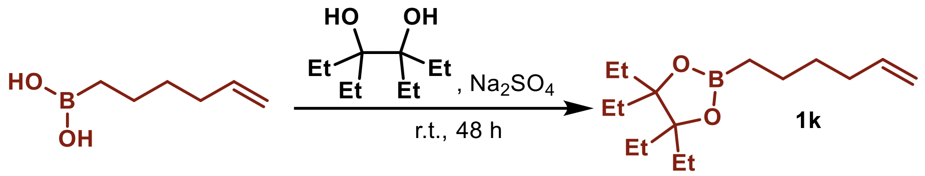

A mixture of boronic acid (0.90 equiv., 17.38 mmol, 1.98 g), 3,4-diethylhexane-3,4-diol (1.00 equiv., 19.31 mmol, 2.00 g) and Na_2SO_4 (10.00 equiv., 175.51 mmol, 25.00 g) in Et_2O (85 mL) was stirred for 48 hours under room temperature, the mixture was filtered and the filtrate was concentrated via rotavap then purified via silica gel column chromatography (eluent: petroleum ether/ethyl acetate = 5/1, stained with $KMnO_4$) to afford the desired olefin **1k**.

Physical Appearance: colorless oil

^{1}H NMR (300 MHz, CDCl$_3$):δ 5.92 – 5.73 (m, 1H), 5.05 – 4.88 (m, 2H), 2.12 – 1.99 (m, 2H), 1.79 – 1.57 (m, 8H), 1.53 – 1.32 (m, 4H), 0.92 (t, *J* = 7.5 Hz, 12H), 0.80 (t, *J* = 7.3 Hz, 2H).

^{13}C NMR (75 MHz, CDCl$_3$): δ 139.2, 114.0, 87.9, 33.6, 31.7, 26.4, 23.8, 11.3, 8.8.

Compound 1l

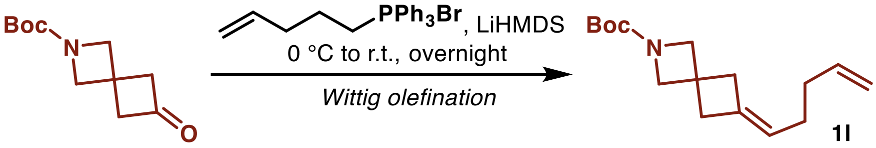

To a vigorously stirred suspension of Wittig salt (1.50 equiv., 14.20 mmol, 5.84 g) in anhydrous THF (50 mL), LiHMDS (2.00 equiv., 18.93 mmol, 1.0 M in THF, 19 mL) was added dropwise under argon at 0 °C, the mixture was then allowed to raise to room temperature and stir for 15 minutes then the ketone (9.47 mmol, 2.00 g) was added and stirred overnight, The mixture was then quenched with saturated NH_4Cl and extracted 3 times with Et_2O, the organic layers were combined and washed again with brine, dried over Na_2SO_4 and concentrated via rotavap then purified via silica gel column chromatography (eluent: petroleum ether/ethyl acetate = 9/1, stained with $KMnO_4$) to afford the desired olefin **1l**.

Physical Appearance: colorless oil

^{1}H NMR (300 MHz, $CDCl_3$): δ 5.87 – 5.68 (m, 1H), 5.21 – 5.07 (m, 1H), 5.05 – 4.89 (m, 2H), 3.90 (s, 4H), 2.78 (s, 4H), 2.12 – 1.88 (m, 4H), 1.42 (s, 9H).

^{13}C NMR (75 MHz, $CDCl_3$): δ 156.4, 138.5, 132.6, 122.0, 114.8, 79.4, 61.5, 42.1, 40.6, 33.8, 33.6, 28.5, 27.9.

HRMS (ESI-TOF) m/z: $[M + Na]^+$ Calcd for $C_{16}H_{25}NNaO_2$ 286.1777; Found 286.1778.

Compound 1m

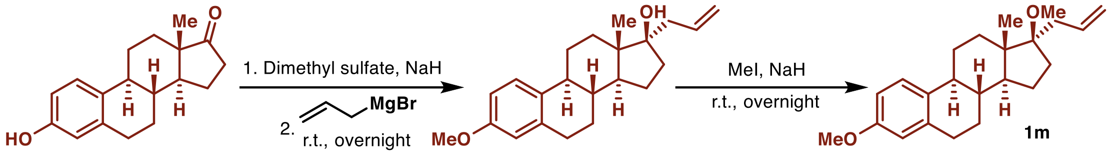

To a suspension of estrone (18.49 mmol, 5.00 g) in anhydrous THF (25 mL) was added NaH (1.50 equiv., 27.74 mmol, 60% in mineral oil, 1.11 g) and stirred at room temperature for 15 minutes then dimethyl sulfate (1.20 equiv., 22.19 mmol, 2.80 g) was added and the mixture was stirred overnight and extracted 3 times with ethyl acetate, the organic layers were combined and washed again with brine, dried over Na_2SO_4 and concentrated via rotavap then purified via silica gel column chromatography (eluent: DCM, stained with DNP) to afford ketone.

To a stirred solution of aforementioned ketone (7.03 mmol, 2.00 g) in anhydrous THF (50 mL) at 0 °C, allylmagnesium bromide (3.00 equiv., 21.10 mmol, 1.0 M in Et_2O, 21 mL) was added via syringe under argon. Upon addition the mixture was allowed to raise to room temperature and stirred overnight. The mixture was then quenched with saturated NH_4Cl and extracted 3 times with Et_2O, the organic layers were combined and washed again with brine, dried over Na_2SO_4 and concentrated via rotavap, the alcohol was used without further purification.

To a stirred solution of aforementioned alcohol (6.22 mmol, 2.03 g) in DMF (15 mL) at 0 °C, NaH (1.50 equiv., 9.33 mmol, 60% in mineral oil, 373 mg) and stirred at room temperature for 15 minutes then MeI (2.00 equiv., 12.44 mmol, 1.77 g) was added and the mixture was stirred overnight**,** The mixture was then quenched with water and extracted 3 times with Et_2O, the organic layers were combined and washed again with brine, dried over Na_2SO_4 and concentrated via rotavap then purified via silica gel column chromatography (eluent: petroleum ether/ethyl acetate = 10/1, stained with $KMnO_4$) to afford the desired olefin **1m**.

Physical Appearance: white solid

^{1}H NMR (300 MHz, CDCl$_3$): δ 7.11 (d, J = 8.6 Hz, 1H), 6.63 (dd, J = 8.6, 2.8 Hz, 1H), 6.57 – 6.51 (m, 1H), 5.92 – 5.75 (m, 1H), 5.13 – 5.02 (m, 2H), 3.69 (s, 3H), 3.19 (s, 3H), 2.89 – 2.67 (m, 3H), 2.28 – 2.14 (m, 1H), 2.13 – 1.94 (m, 2H), 1.92 – 1.71 (m, 3H), 1.70 – 1.17 (m, 8H), 0.88 (s, 3H).

^{13}C NMR (75 MHz, CDCl$_3$): δ 157.5, 138.0, 135.0, 132.6, 126.3, 117.4, 113.8, 111.5, 87.4, 55.2, 51.2, 50.9, 47.0, 43.6, 39.4, 35.4, 34.5, 33.8, 29.9, 27.4, 26.6, 23.2, 13.9.

Compound 1n

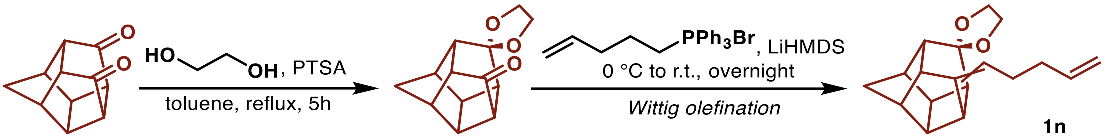

1n

The mixture of ketone (5.74 mmol, 1.00 g), glycol (1.00 equiv., 5.74 mmol, 356 mg), PTSA (0.10 equiv., 0.29 mmol, 55 mg) in toluene (12 mL) was heated to reflux for 5 hours, the mixture was quenched with saturated $NaHCO_3$, and extracted with DCM for 3 times the organic layers were combined and washed again with brine, dried over Na_2SO_4 and concentrated via rotavap then purified via silica gel column chromatography (eluent: petroleum ether/ ethyl acetate = 1.5/1, stain with DNP) to afford a white solid.

To a vigorously stirred suspension of Wittig salt (2.00 equiv., 7.16 mmol, 2.95 g) in anhydrous THF (25 mL) at 0 °C, LiHMDS (2.00 equiv., 1.0 M in Et_2O, 7.16 mmol, 7.2 mL) was added via syringe, the mixture was then allowed raise to room temperature and stir for 15 minutes then the aforementioned ketone (3.58 mmol, 897 mg) was added in one portion and stirred overnight, The mixture was then quenched with saturated solution of NH_4Cl and extracted 3 times with Et_2O, the organic layers were combined and washed again with brine, dried over Na_2SO_4 and concentrated via rotavap then purified via silica gel column chromatography (eluent: petroleum ether/ethyl acetate = 5/1, stained with $KMnO_4$) to afford the desired olefin **1n** as a mixture of *Z* and *E* isomers.

Physical Appearance: yellow oil

^{1}H NMR (300 MHz, $CDCl_3$): δ 5.95 – 5.75 (m, 1H), 5.25 – 4.81 (m, 3H), 3.99 – 3.65 (m, 4H), 3.27 – 2.52 (m, 5H), 2.52 – 2.40 (m, 1H), 2.32 – 1.94 (m, 6H), 1.75 – 1.64 (m, 1H), 1.39 – 1.27 (m, 1H).

^{13}C NMR (75 MHz, $CDCl_3$): δ 144.1, 144.0, 139.1, 116.7, 116.2, 116.2, 114.2, 114.1, 65.6, 65.5, 63.1, 63.0, 50.7, 50.3, 49.6, 47.4, 46.8, 44.9, 44.9, 43.1, 42.9, 42.2, 41.8, 41.5, 41.4, 40.0, 39.7, 36.6, 36.4, 35.1, 34.3, 34.3, 28.5, 28.4.

Compound 1o

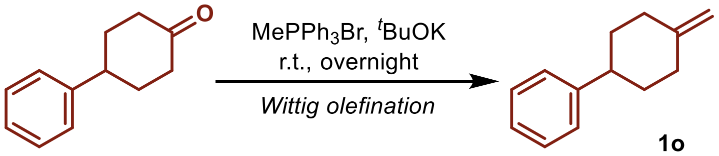

Preparation executed according to literature procedure.[3] To a vigorously stirred suspension of Wittig salt (1.50 equiv., 17.22 mmol, 6.15 g) in anhydrous THF (50 mL), ^{t}BuOK (1.50 equiv., 17.22 mmol, 1.93 g) was added in one portion, the mixture was then allowed to stir for 15 minutes then the ketone (11.48 mmol, 2.00 g) was added in one portion and stirred overnight, The mixture was then quenched with saturated NH_4Cl and extracted 3 times with Et_2O, the organic layers were combined and washed again with brine, dried over Na_2SO_4 and concentrated via rotavap then purified via silica gel column chromatography (eluent: petroleum ether, stained with $KMnO_4$) to afford the desired olefin **1o**.

Physical Appearance: colorless oil

^{1}H NMR (300 MHz, $CDCl_3$): δ 7.27 – 7.06 (m, 5H), 4.60 (t, *J* = 1.7 Hz, 2H), 2.67 – 2.52 (m, 1H), 2.44 – 2.26 (m, 2H), 2.21 – 2.02 (m, 2H), 1.96 – 1.85 (m, 2H), 1.56 – 1.38 (m, 2H).

^{13}C NMR (75 MHz, $CDCl_3$): δ 149.0, 147.0, 128.5, 127.0, 126.1, 107.5, 44.3, 35.7, 35.3.

Characterization data for this olefin was in agreement with the literature.[3]

Compound 1p

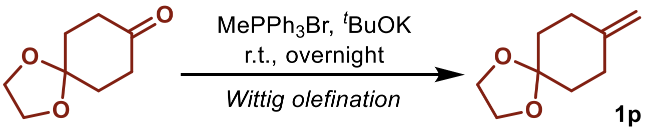

Preparation executed according to literature procedure.[4] To a vigorously stirred suspension of Wittig salt (1.20 equiv., 15.37 mmol, 5.49 g) in anhydrous THF (50 mL), ^{t}BuOK (1.20 equiv., 15.37 mmol, 1.72 g) was added in one portion, the mixture was then allowed to stir for 15 minutes then the ketone (12.81 mmol, 2.00 g) was added in one portion and stirred overnight, The mixture was then quenched with saturated NH_4Cl and extracted 3 times with Et_2O, the organic layers were combined and washed again with brine, dried over Na_2SO_4 and concentrated via rotavap then purified via silica gel column chromatography (eluent: petroleum ether/ethyl acetate = 20/1, stained with $KMnO_4$) to afford the desired olefin **1p**.

Physical Appearance: colorless oil

^{1}H NMR (300 MHz, $CDCl_3$): δ 4.66 (s, 2H), 3.96 (s, 4H), 2.33 – 2.23 (m, 4H), 1.75 – 1.64 (m, 4H).

^{13}C NMR (75 MHz, $CDCl_3$): δ 147.4, 108.6, 108.3, 64.4, 36.0, 32.1.

Characterization data for this olefin was in agreement with the literature.[4]

Compound 1q

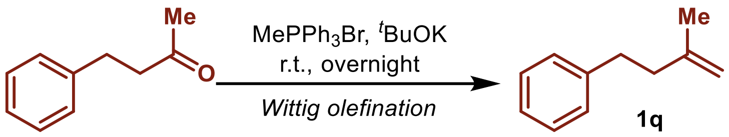

Preparation executed according to literature procedure.[5] To a vigorously stirred suspension of Wittig salt (1.50 equiv., 30.36 mmol, 10.85 g) in anhydrous THF (50 mL), ^{t}BuOK (1.50 equiv., 30.36 mmol, 3.41 g) was added in one portion, the mixture was then allowed to stir for 15 minutes then the ketone (20.24 mmol, 3.00 g) was added in one portion and stirred overnight, The mixture was then quenched with saturated NH_4Cl and extracted 3 times with Et_2O, the organic layers were combined and washed again with brine, dried over Na_2SO_4 and concentrated via rotavap then purified via silica gel column chromatography (eluent: petroleum ether, stained with $KMnO_4$) to afford the desired olefin **1q**.

Physical Appearance: colorless oil

^{1}H NMR (300 MHz, $CDCl_3$): δ 7.27 – 7.04 (m, 5H), 4.70 – 4.61 (m, 2H), 2.74 – 2.62 (m, 2H), 2.30 – 2.19 (m, 2H), 1.70 (s, 3H).

^{13}C NMR (75 MHz, $CDCl_3$): δ 145.5, 142.4, 128.5, 128.4, 125.9, 110.3, 39.7, 34.4, 22.8.

Characterization data for this olefin was in agreement with the literature.[5]

Compound 1r

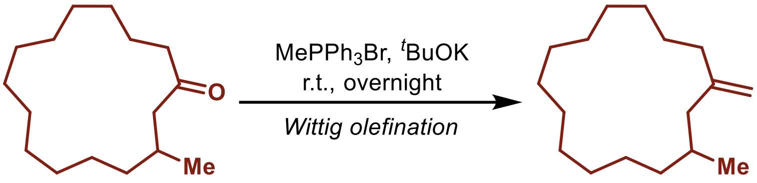

1r

To a vigorously stirred suspension of Wittig salt (1.50 equiv., 6.29 mmol, 2.25 g) in anhydrous THF (20 mL), ^{t}BuOK (1.50 equiv., 6.29 mmol, 706 mg) was added in one portion, the mixture was then allowed to stir for 15 minutes then the ketone (4.19 mmol, 1.00 g) was added in one portion and stirred overnight, The mixture was then quenched with saturated NH_4Cl and extracted 3 times with Et_2O, the organic layers were combined and washed again with brine, dried over Na_2SO_4 and concentrated via rotavap then purified via silica gel column chromatography (eluent: petroleum ether, stained with $KMnO_4$) to afford the desired olefin **1r**.

Physical Appearance: colorless oil

^{1}H NMR (300 MHz, CDCl$_3$): δ 4.78 – 4.65 (m, 2H), 2.16 – 1.88 (m, 3H), 1.81 – 1.17 (m, 24H), 0.84 (d, J = 6.5 Hz, 3H).

^{13}C NMR (75 MHz, CDCl$_3$): δ 149.4, 110.9, 44.3, 35.6, 35.2, 29.6, 27.7, 27.2, 27.0, 26.9, 26.9, 26.8, 26.8, 26.6, 25.4, 20.4.

HRMS (ESI-TOF) m/z: $[2M + H]^+$ Calcd for $C_{34}H_{65}$ 473.5081; Found 473.5089.

Compound 1s

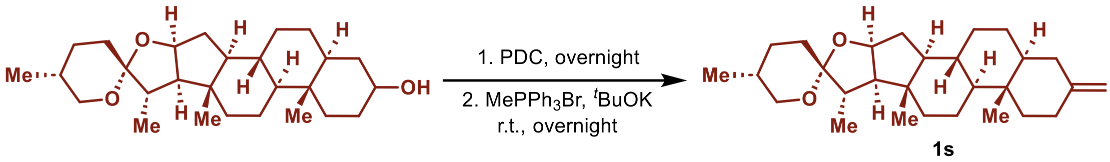

To a stirred suspension of PDC (2.11 equiv., 50.49 mmol, 15.00 g), in DCM (100 mL) was added the alcohol (23.94 mmol, 10.00 g), the mixture was then stirred vigorously overnight, then filtered and concentrated the filtrate, then purified via silica gel column chromatography (eluent: petroleum ether/ethyl acetate = 6/1, stained with DNP) to afford the ketone.

To a vigorously stirred suspension of Wittig salt (1.50 equiv., 8.25 mmol, 2.95 g) in anhydrous THF (20 mL), ^{t}BuOK (1.50 equiv., 8.25 mmol, 926 mg) was added in one portion, the mixture was then allowed to stir for 15 minutes then the ketone (5.50 mmol, 2.28 g) was added in one portion and stirred overnight, The mixture was then quenched with saturated NH_4Cl and extracted 3 times with DCM, the organic layers were combined and washed again with brine, dried over Na_2SO_4 and concentrated via rotavap then purified via silica gel column chromatography (eluent: petroleum ether/ethyl acetate = 9/1, stained with $KMnO_4$) to afford the desired olefin **1s**.

Physical Appearance: white solid

^{1}H NMR (300 MHz, $CDCl_3$): δ 4.59 – 4.49 (m, 2H), 4.45 – 4.32 (m, 1H), 3.52 – 3.30 (m, 2H), 2.25 – 2.09 (m, 2H), 2.07 – 1.87 (m, 4H), 1.80 – 1.38 (m, 12H), 1.38 – 1.04 (m, 7H), 0.95 (d, J = 6.8 Hz, 4H), 0.87 (s, 3H), 0.81 – 0.73 (m, 6H), 0.72 – 0.60 (m, 1H).

^{13}C NMR (75 MHz, $CDCl_3$): δ 150.1, 109.4, 106.2, 81.0, 67.0, 62.3, 56.4, 54.5, 48.2, 41.7, 40.7, 40.2, 39.9, 38.1, 36.2, 35.2, 32.3, 31.9, 31.5, 31.1, 30.4, 28.9, 28.9, 21.1, 17.3, 16.6, 14.6, 11.9.

HRMS (ESI-TOF) m/z: $[M + H]^+$ Calcd for $C_{28}H_{45}O_2$ 413.3414; Found 413.3430.

Compound 1t

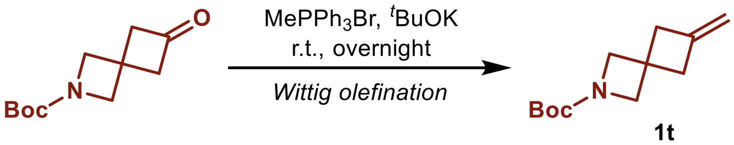

To a vigorously stirred suspension of Wittig salt (1.20 equiv., 11.36 mmol, 4.06 g) in anhydrous THF (50 mL), ^{t}BuOK (1.20 equiv., 11.36 mmol, 1.27 g) was added in one portion, the mixture was then allowed to stir for 15 minutes then the ketone (9.47 mmol, 2 g) was added in one portion and stirred overnight, The mixture was then quenched with saturated NH_4Cl and extracted 3 times with Et_2O, the organic layers were combined and washed again with brine, dried over Na_2SO_4 and concentrated via rotavap then purified via silica gel column chromatography (eluent: petroleum ether/ethyl acetate = 20/1, stained with $KMnO_4$) to afford the desired olefin **1t**.

Physical Appearance: white solid

^{1}H NMR (300 MHz, $CDCl_3$): δ 4.80 – 4.75 (m, 2H), 3.90 (s, 4H), 2.82 (t, J = 2.5 Hz, 4H), 1.41 (s, 9H).

^{13}C NMR (75 MHz, $CDCl_3$): δ 156.3, 142.9, 107.3, 79.4, 61.3, 42.9, 33.2, 28.5.

HRMS (ESI-TOF) m/z: $[M + Na]^+$ Calcd for $C_{12}H_{19}NNaO_2$ 232.1308; Found 232.1304.

Compound 1w

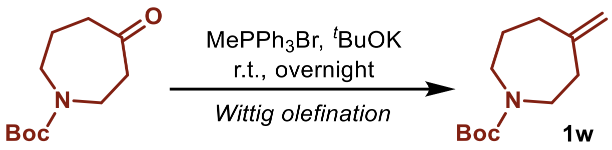

Preparation executed according to literature procedure.[6] To a vigorously stirred suspension of Wittig salt (1.20 equiv., 11.25 mmol, 4.02 g) in anhydrous THF (50 mL), ^{t}BuOK (1.20 equiv., 11.25 mmol, 1.26 g) was added in one portion, the mixture was then allowed to stir for 15 minutes then the ketone (9.38 mmol, 2.00 g) was added in one portion and stirred overnight, The mixture was then quenched with saturated NH_4Cl and extracted 3 times with Et_2O, the organic layers were combined and washed again with brine, dried over Na_2SO_4 and concentrated via rotavap then purified via silica gel column chromatography (eluent: petroleum ether/ethyl acetate = 20/1, stained with $KMnO_4$) to afford the desired olefin **1w**.

Physical Appearance: colorless oil

^{1}H NMR (300 MHz, $CDCl_3$): δ 4.82 – 4.68 (m, 2H), 3.46 – 3.26 (m, 4H), 2.47 – 2.32 (m, 2H), 2.28 – 2.15 (m, 2H), 1.74 – 1.60 (m, 2H), 1.43 (s, 9H).

^{13}C NMR (75 MHz, $CDCl_3$): δ 155.3, 149.0, 148.7, 112.9, 112.9, 79.2, 48.3, 48.0, 46.6, 36.8, 36.5, 34.9, 34.7, 29.0, 28.6.

Characterization data for this olefin was in agreement with the literature.[6]

Compound 1x

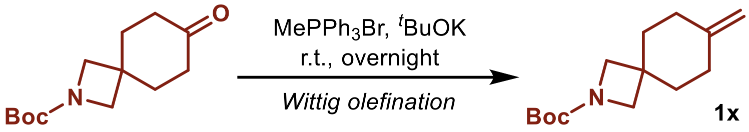

To a vigorously stirred suspension of Wittig salt (1.20 equiv., 10.03 mmol, 3.58 g) in anhydrous THF (50 mL), *t*BuOK (1.20 equiv., 10.03 mmol, 1.13 g) was added in one portion, the mixture was then allowed to stir for 15 minutes then the ketone (8.36 mmol, 2.00 g) was added in one portion and stirred overnight, The mixture was then quenched with saturated NH_4Cl and extracted 3 times with Et_2O, the organic layers were combined and washed again with brine, dried over Na_2SO_4 and concentrated via rotavap then purified via silica gel column chromatography (eluent: petroleum ether/ethyl acetate = 9/1, stained with $KMnO_4$) to afford the desired olefin **1x**.

Physical Appearance: white solid

^{1}H NMR (300 MHz, $CDCl_3$): δ 4.58 (s, 2H), 3.56 (s, 4H), 2.10 – 2.00 (m, 4H), 1.71 – 1.61 (m, 4H), 1.39 (s, 9H).

^{13}C NMR (75 MHz, $CDCl_3$): δ 156.5, 147.1, 108.0, 79.2, 59.4, 58.4, 37.1, 34.8, 31.2, 28.4.

HRMS (ESI-TOF) m/z: $[M + Na]^+$ Calcd for $C_{14}H_{23}NNaO_2$ 260.1621; Found 260.1620.

Compound 1y

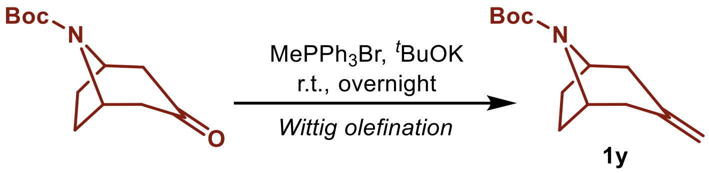

To a vigorously stirred suspension of Wittig salt (2.50 equiv., 11.10 mmol, 3.96 g) in anhydrous THF (50 mL), ^{t}BuOK (2.50 equiv., 11.10 mmol, 1.25 g) was added in one portion, the mixture was then allowed to stir for 15 minutes then the ketone (4.44 mmol, 1.00 g) was added in one portion and stirred overnight, The mixture was then quenched with saturated NH_4Cl and extracted 3 times with Et_2O, the organic layers were combined and washed again with brine, dried over Na_2SO_4 and concentrated via rotavap then purified via silica gel column chromatography (eluent: petroleum ether/ethyl acetate = 9/1, stained with $KMnO_4$) to afford the desired olefin **1y**.

Physical Appearance: colorless oil

^{1}H NMR (300 MHz, $CDCl_3$): δ 4.82 – 4.80 (m, 2H), 4.33 – 4.06 (m, 2H), 2.55 – 2.31 (m, 2H), 2.12 – 1.98 (m, 2H), 1.92 – 1.72 (m, 2H), 1.60 – 1.48 (m, 2H), 1.44 (s, 9H).

^{13}C NMR (75 MHz, $CDCl_3$): δ 153.6, 142.4, 113.4, 79.3, 54.5, 54.0, 41.2, 40.5, 28.6, 27.9.

HRMS (ESI-TOF) m/z: $[M + Na]^+$ Calcd for $C_{13}H_{21}NNaO_2$ 246.1464; Found 246.1460.

Compound 1aa

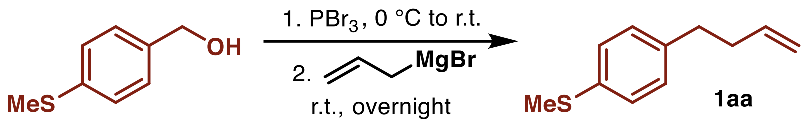

1aa

To a stirred solution of alcohol (12.97 mmol, 2.00 g) in Et_2O (15 mL) was added PBr_3 (1.00 equiv., 12.97 mmol, 3.51 g) dropwise at 0 °C, upon addition the mixture was allowed to raise to room temperature and stirred for a few hours (monitored by TLC), the mixture was then poured into water at room temperature and diluted with additional Et_2O (50 mL), the organic layer was then washed with saturated solution of $NaHCO_3$, brine, then dried over Na_2SO_4 and concentrated via rotavap, the crude bromide was used without further purification.

Preparation executed according to literature procedure.[7] To a stirred solution of aforementioned alkyl bromide (9.44 mmol, 2.05 g) in anhydrous THF (20 mL) at 0 °C, allylmagnesium bromide (1.50 equiv., 14.16 mmol, 1.0 M in Et_2O, 14 mL) was added via syringe under argon. Upon addition the mixture was allowed to raise to room temperature and stirred overnight. The mixture was then quenched with saturated NH_4Cl and extracted 3 times with Et_2O, the organic layers were combined and washed again with brine, dried over Na_2SO_4 and concentrated via rotavap then purified via silica gel column chromatography (eluent: petroleum ether) to afford the desired olefin **1aa**.

Physical Appearance: colorless oil

^{1}H NMR (300 MHz, $CDCl_3$): δ 7.15 – 7.09 (m, 2H), 7.06 – 7.00 (m, 2H), 5.86 – 5.67 (m, 1H), 5.06 – 4.84 (m, 1H), 2.65 – 2.54 (m, 2H), 2.39 (s, 3H), 2.34 – 2.20 (m, 2H).

^{13}C NMR (75 MHz, $CDCl_3$): δ 139.1, 138.1, 135.4, 129.1, 127.2, 115.2, 35.6, 34.9, 16.4.

Characterization data for this olefin was in agreement with the literature.[7]

Compound 1ab

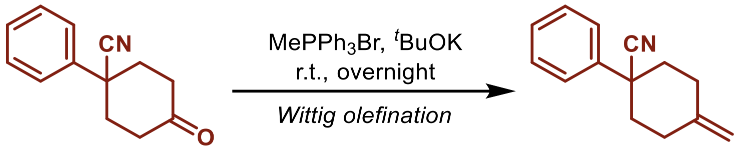

1ab

To a vigorously stirred suspension of Wittig salt (1.50 equiv., 15.06 mmol, 5.38 g) in anhydrous THF (50 mL), ^{t}BuOK (1.50 equiv., 15.06 mmol, 1.69 g) was added in one portion, the mixture was then allowed to stir for 15 minutes then the ketone (10.04 mmol, 2.00 g) was added in one portion and stirred overnight, The mixture was then quenched with saturated NH_4Cl and extracted 3 times with Et_2O, the organic layers were combined and washed again with brine, dried over Na_2SO_4 and concentrated via rotavap then purified via silica gel column chromatography (eluent: petroleum ether/ethyl acetate = 20/1, stained with $KMnO_4$) to afford the desired olefin **1ab**.

Physical Appearance: colorless oil

^{1}H NMR (300 MHz, $CDCl_3$): δ 7.45 – 7.19 (m, 5H), 4.69 (t, J = 1.8 Hz, 2H), 2.62 – 2.30 (m, 4H), 2.21 – 2.09 (m, 2H), 1.81 (td, J = 13.3, 4.3 Hz, 2H).

^{13}C NMR (75 MHz, $CDCl_3$): δ 145.4, 140.5, 129.1, 128.1, 125.7, 122.3, 109.4, 44.2, 38.4, 32.1.

HRMS (ESI-TOF) m/z: $[2M + H]^+$ Calcd for $C_{28}H_{31}N_2$ 395.2482; Found 395.2482.

3.2 Graphical guide

Before setting up the reaction, the following parts need to prepared:

1 × Mg electrode as anode,

1 × Ti electrode as cathode,

1 × Parafilm (2 cm x 3 cm)

1 × stirring bar

1 × ElectraSyn 2.0 accessories

1 × ElectraSyn 2.0 cap,

1 × ElectraSyn 2.0 vial (10 mL)

Note: For gram-scale synthesis, 20 mL vial and the corresponding cap was used.

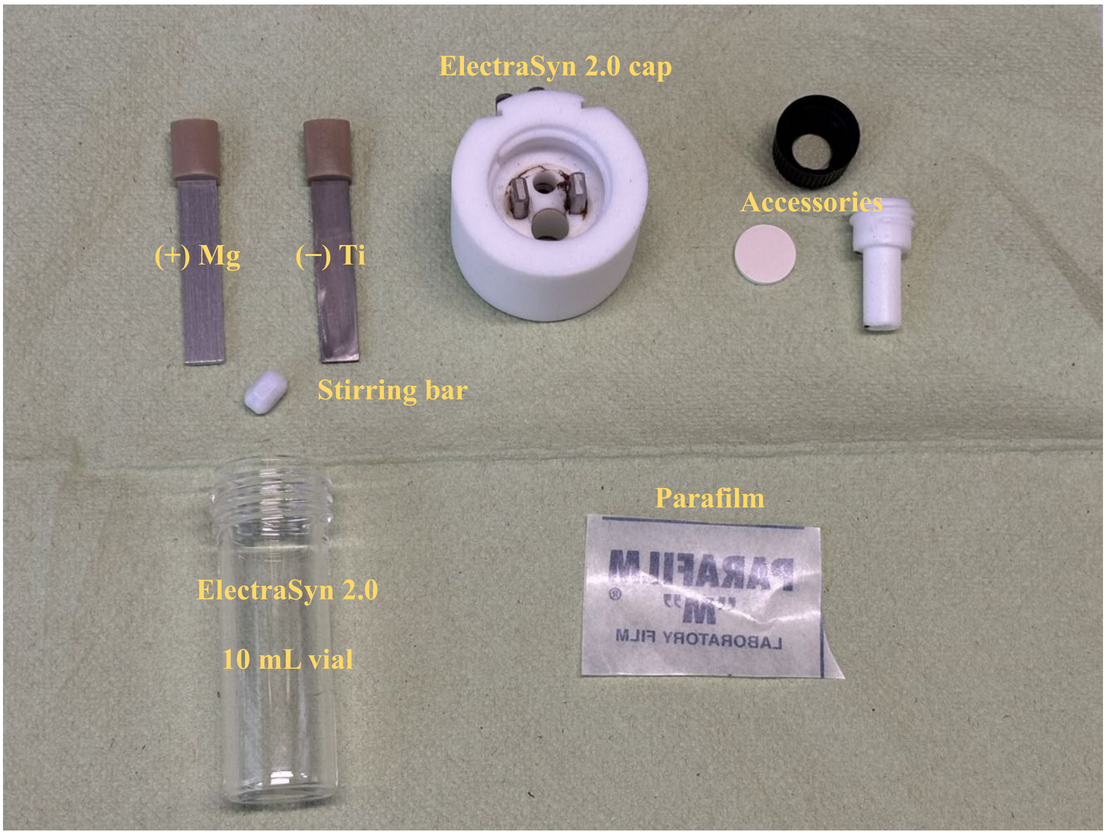

The following chemicals need to be prepared as well:

Starting material: 4-phenyl-1-butene (1.00 mmol, 132 mg),

Electrolyte: TBABr (0.1 M, 1.00 mmol, 322 mg)

Note: For dimethylamination and gram-scale synthesis, TEABr (0.1 M, 1.00 mmol, 210 mg) was used as electrolyte, as the generated triethylamine can be easily removed via rotavap.

Solvent A: DMF (5 mL)

Solvent B: 2-MeTHF (4 mL)

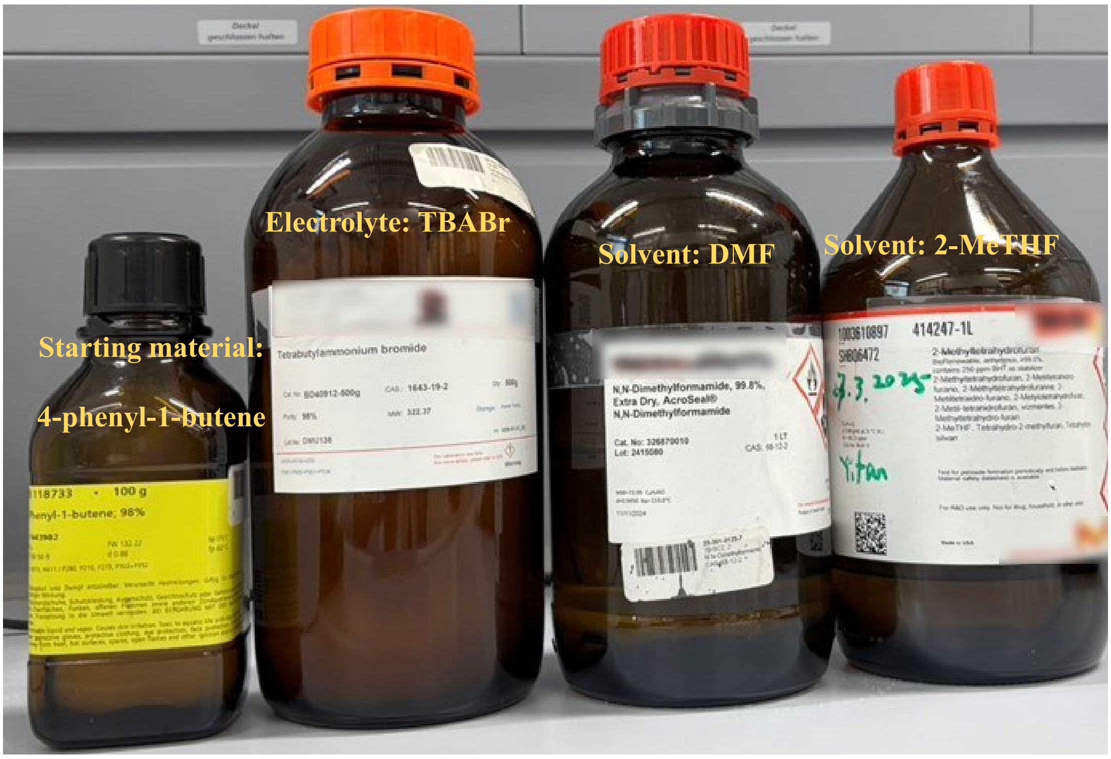

Step 1: Assemble ElectraSyn 2.0 cap with Mg plate as anode and Ti as cathode. Warp around ElectraSyn 2.0 vial with parafilm to prevent leakage. Assemble the accessories.

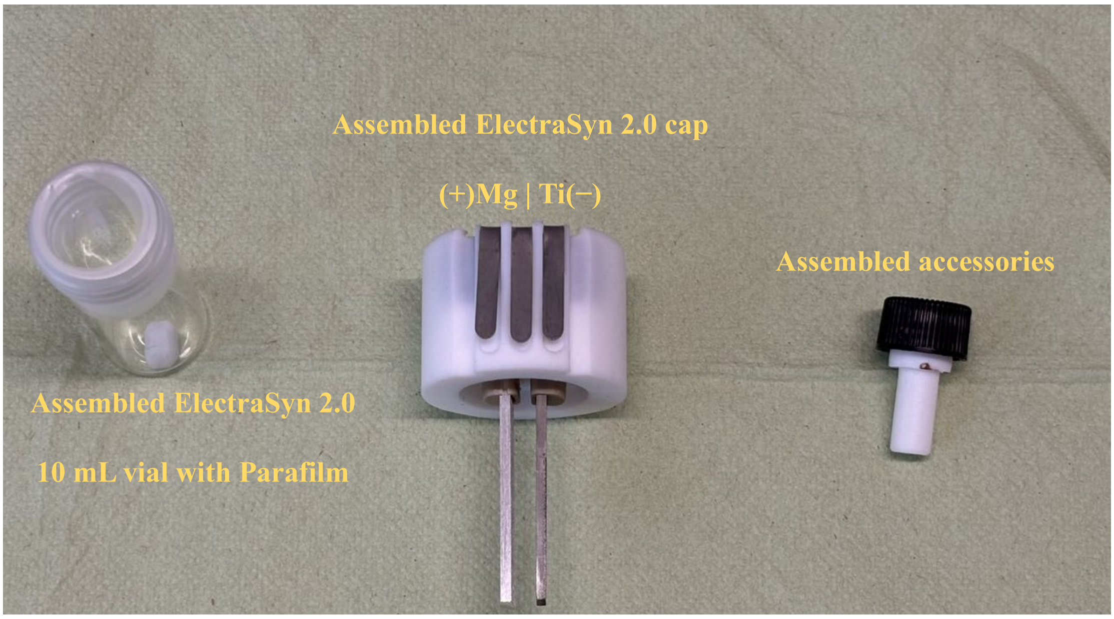

Step 2: Weigh and add correct amount of the electrolyte: TBABr: (0.1 M, 1.00 mmol, 322 mg).

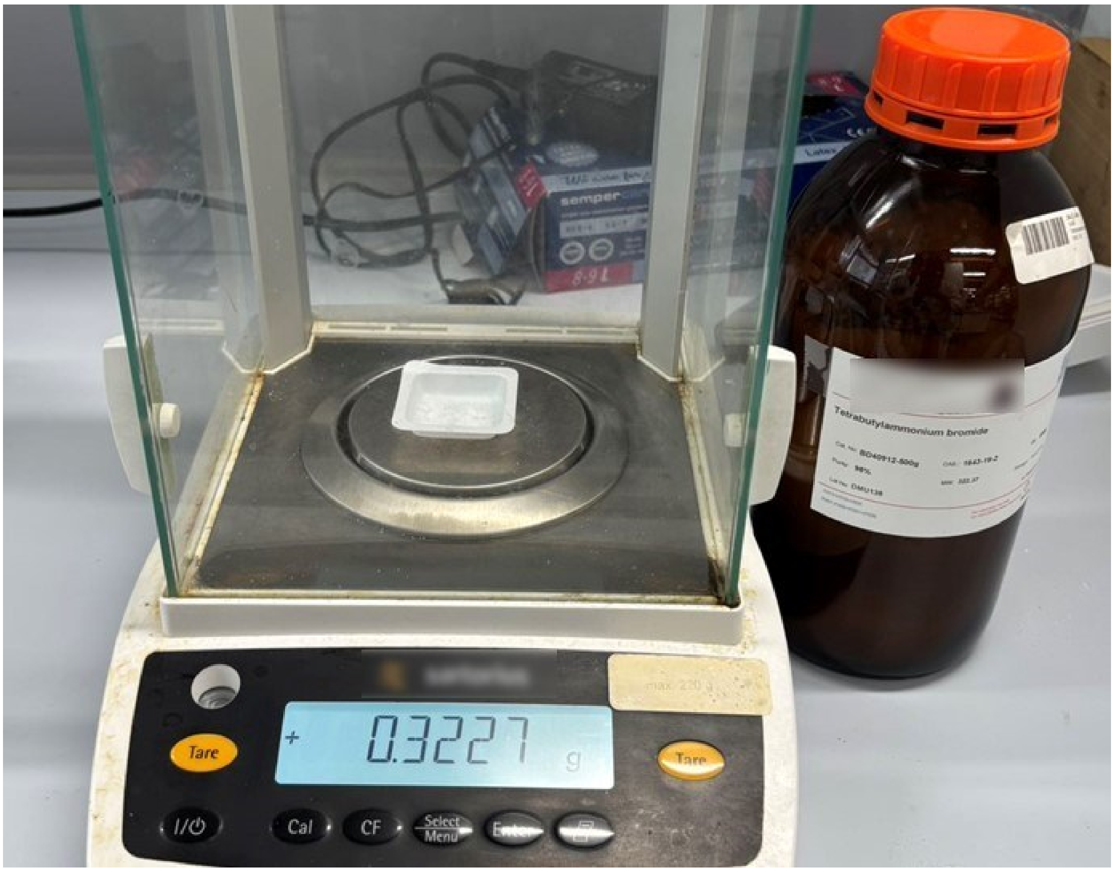

Step 3: Add the mixture of DMF (5 mL) and 2-MeTHF (4 mL).

Note: For gram-scale synthesis DMF (10 mL) and 2-MeTHF (4 mL) were added instead. For ketone synthesis, DMA (5 mL) was added instead.

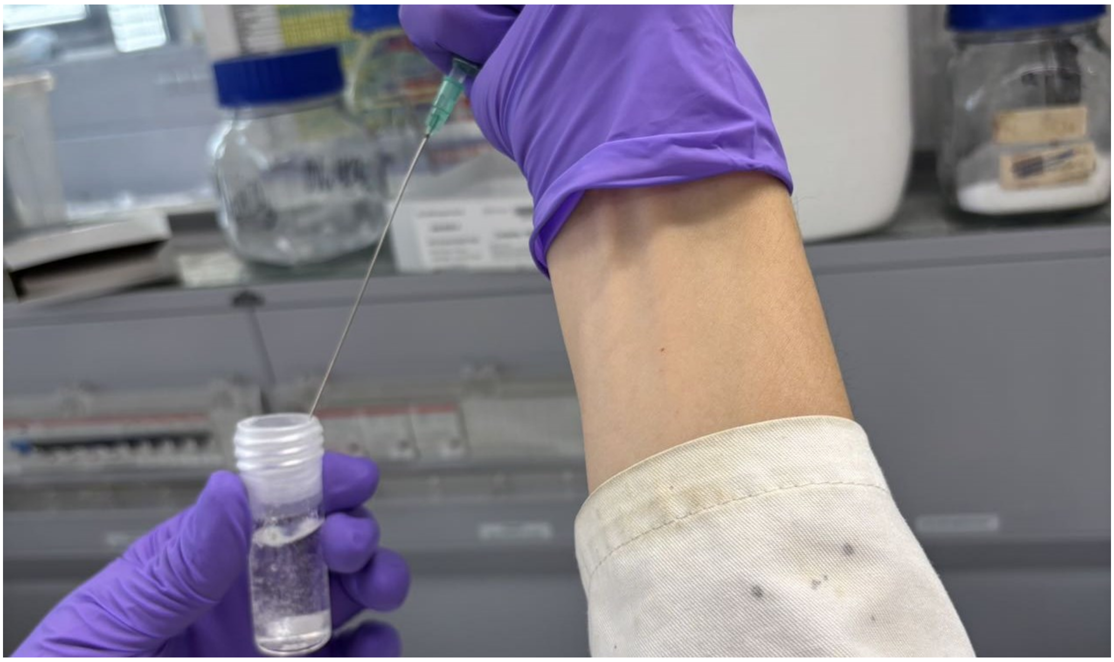

Step 4: Purge the solution with an argon balloon for 5 minutes.

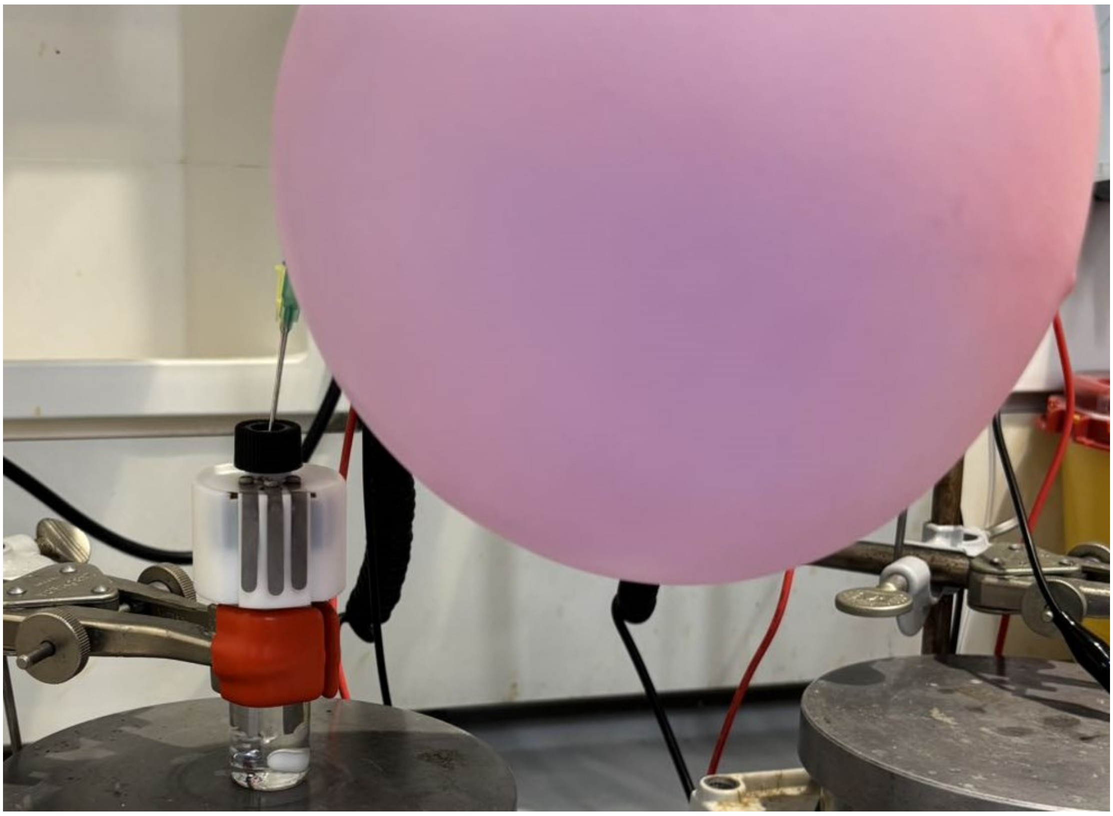

Step 5: Weigh and add the starting material **1a**: 4-phenyl-1-butene (1.00 mmol, 132 mg). via Hamilton

Note: If starting material is a solid or a high boiling point oil, it can also be added before purging for convenience.

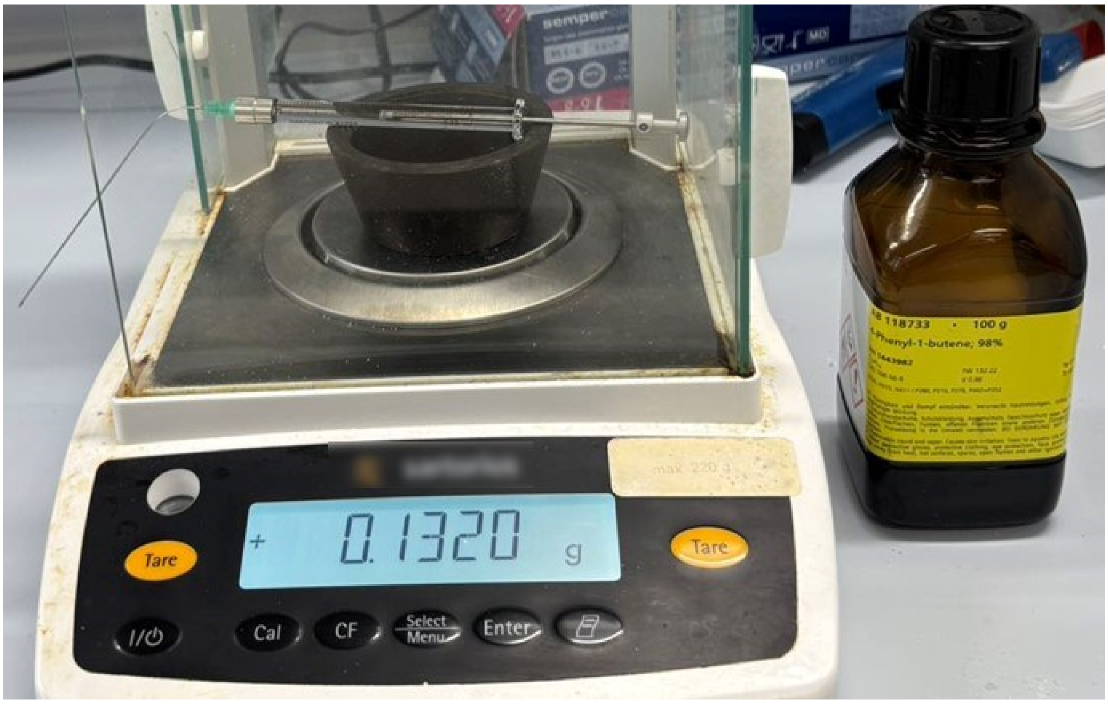

Step 6: Set the parameters for electrolysis: (+)Mg | Ti(−); Constant current, 40 mA, 1.00 mmol, 10 h, and start the reaction under argon.

Note: Electrolysis time can be extended for some starting materials.

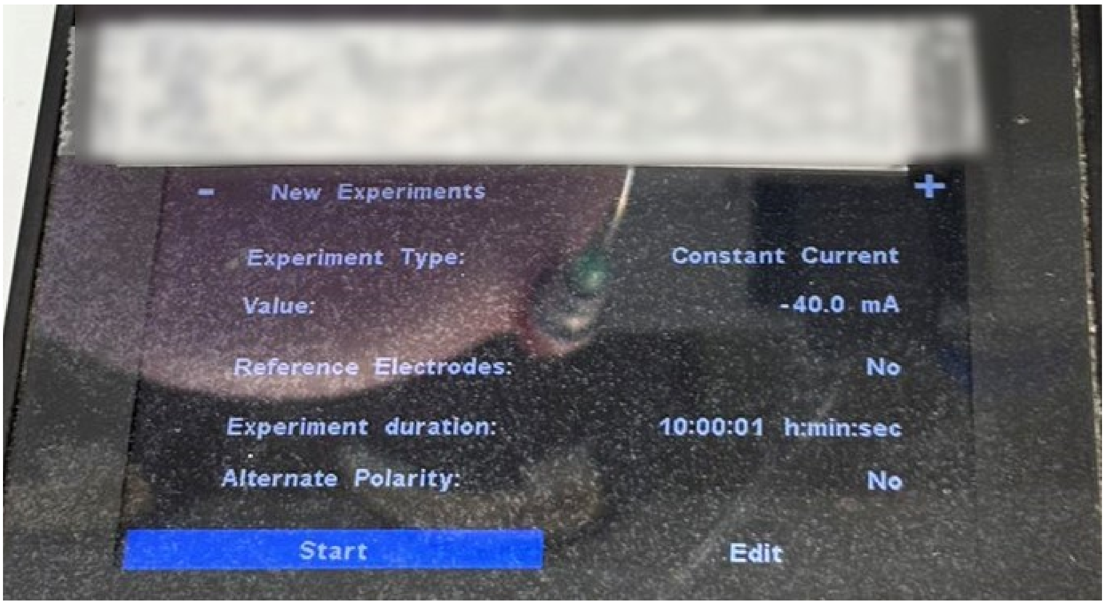

Step 7: Remove the electrolysis vial after 10 hours.

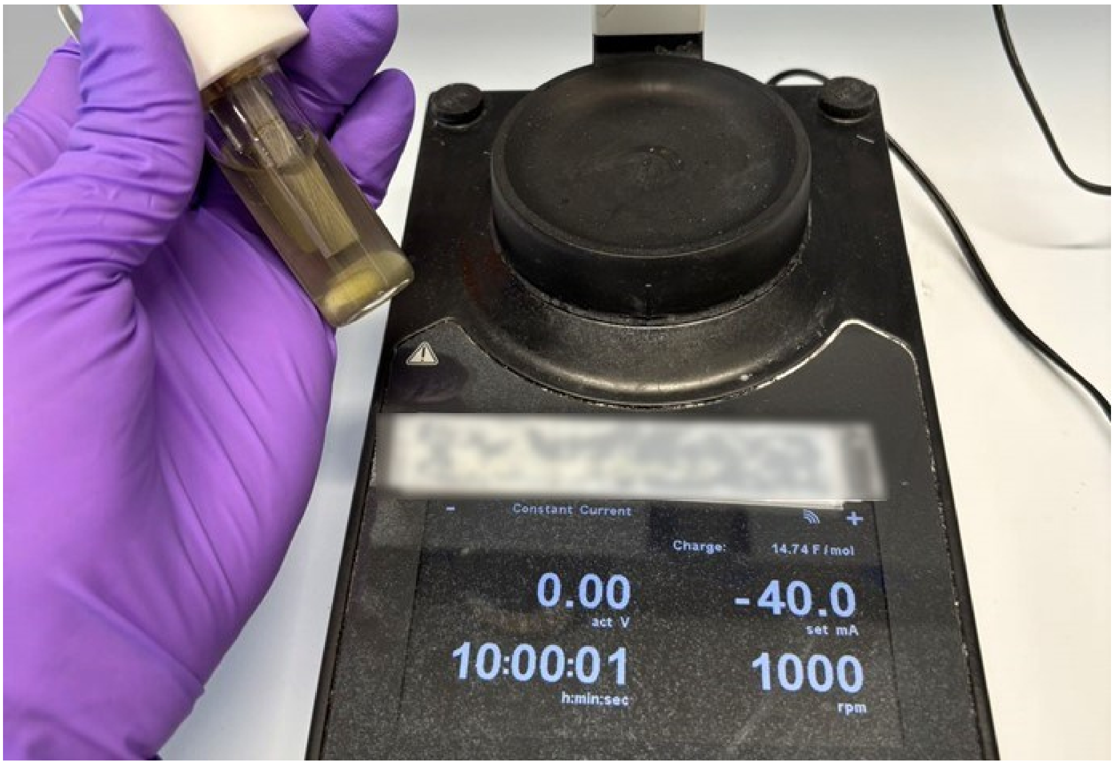

Step 8: Transfer the reaction mixture to a separatory funnel, rinse both electrodes and the vial with Et_2O and water.

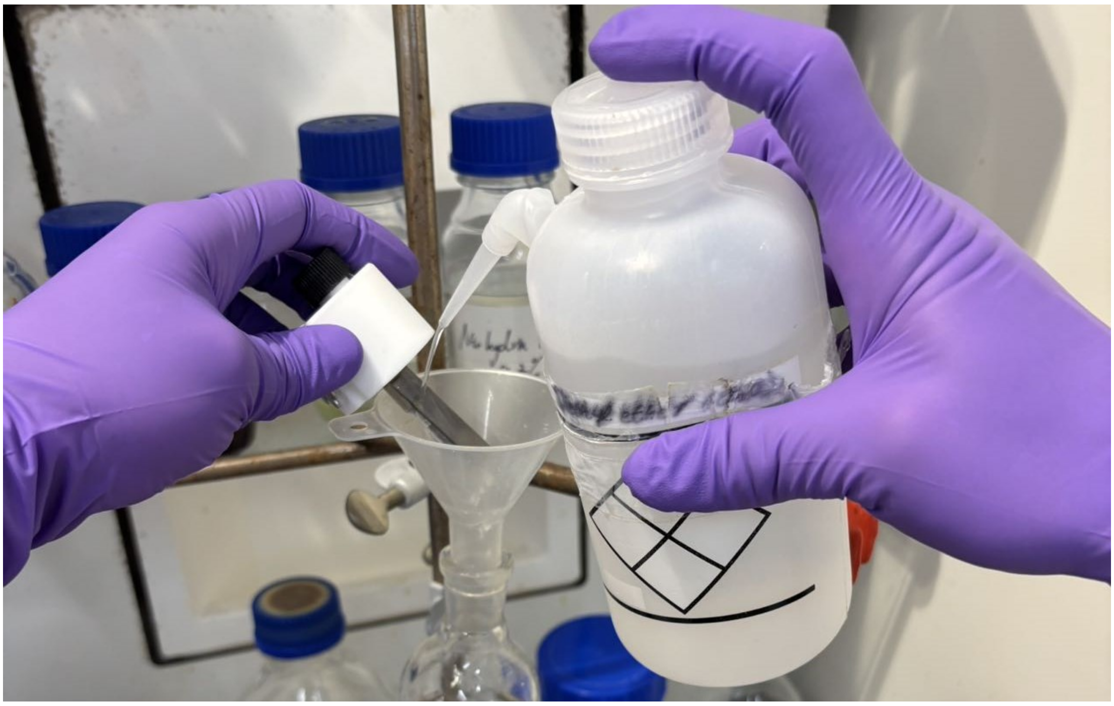

Step 9: Add HCl (3.2 M) to the mixture.

Note: If liable functional group (for example Boc protected amines) exist, weak acid like citric acid (10 wt%) can also be used. Due to large amount of tributylamine generated during the electrolysis, excess amount of acid is recommended to add.

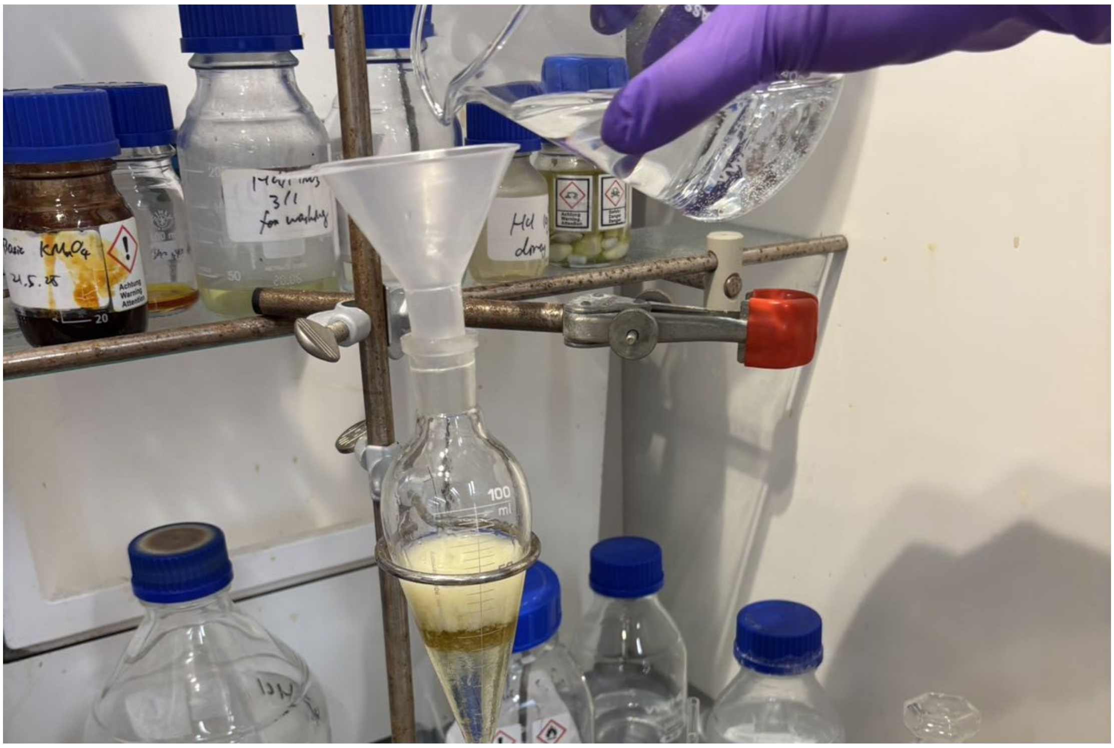

Step 10: Shake the mixture vigorously and extract the aqueous layer 3 times with Et_2O, combined and wash organic layers with brine.

Note: Special smell of aldehyde can be spotted if the product is volatile (lauric aldehyde for example).

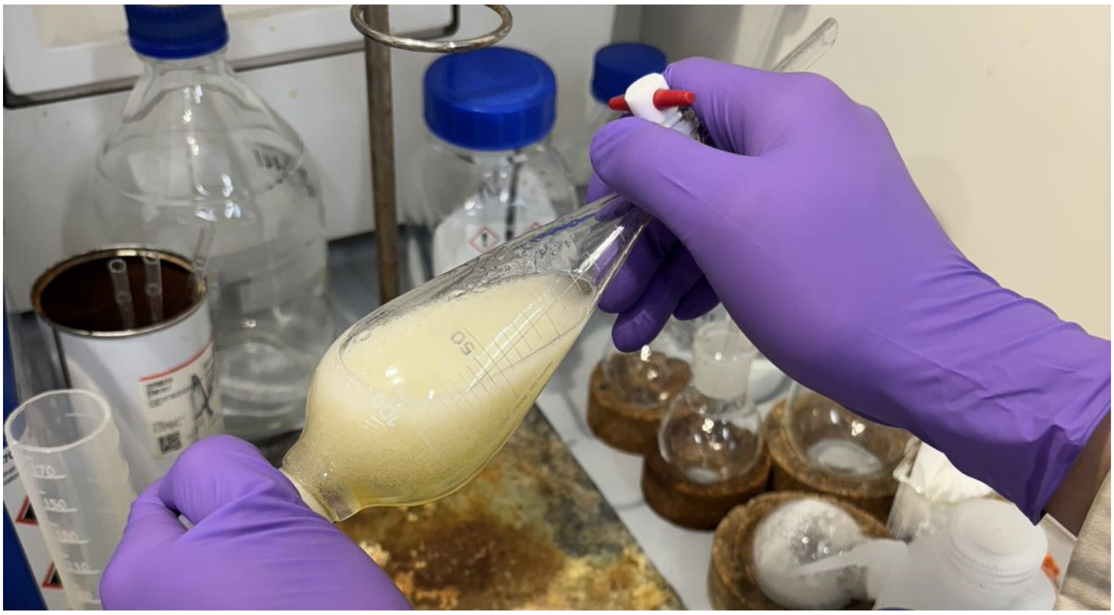

Step 11: Add Na_2SO_4 to organic phase till it turns transparent.

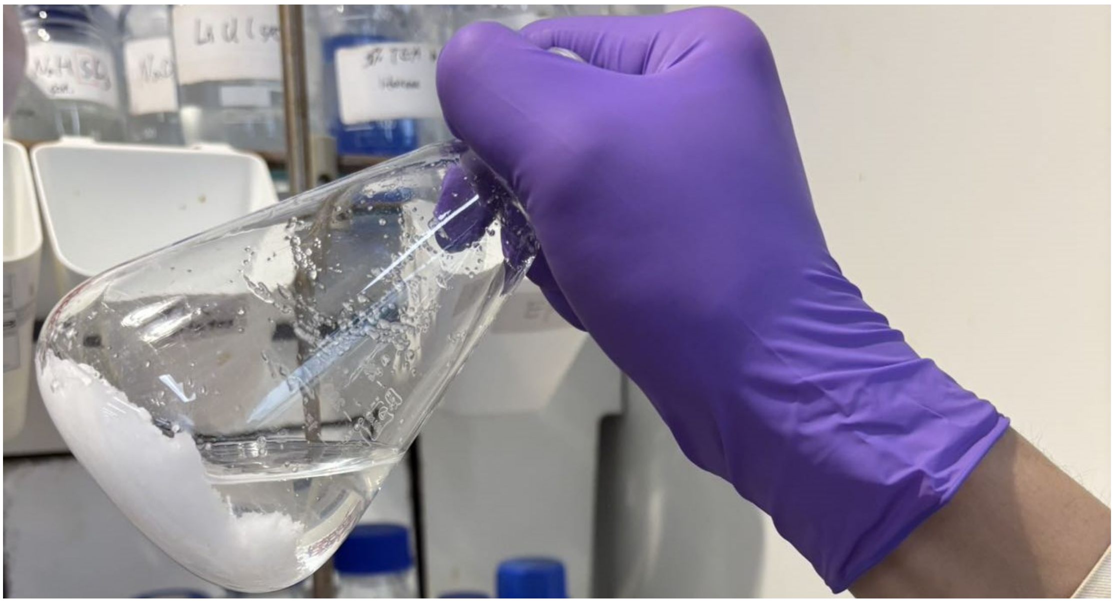

Step 12: Concentrate the mixture via rotavap.

Note: If not add enough acid, tributylamine (b.p. 214 °C) will stay in the mixture and significantly tail the column chromatography afterwards.

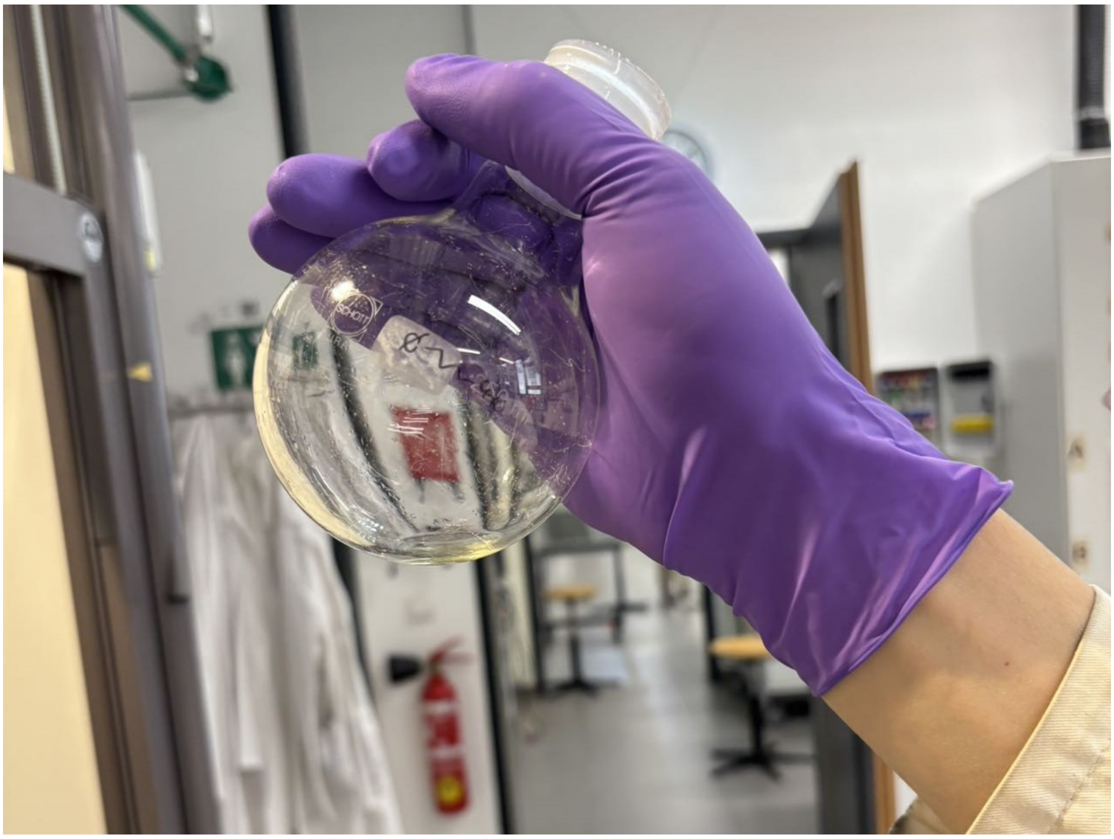

Step 13: Separate the product via silica gel column chromatography.

The following TLCs were prepared with petroleum ether/ethyl acetate : 9/1.

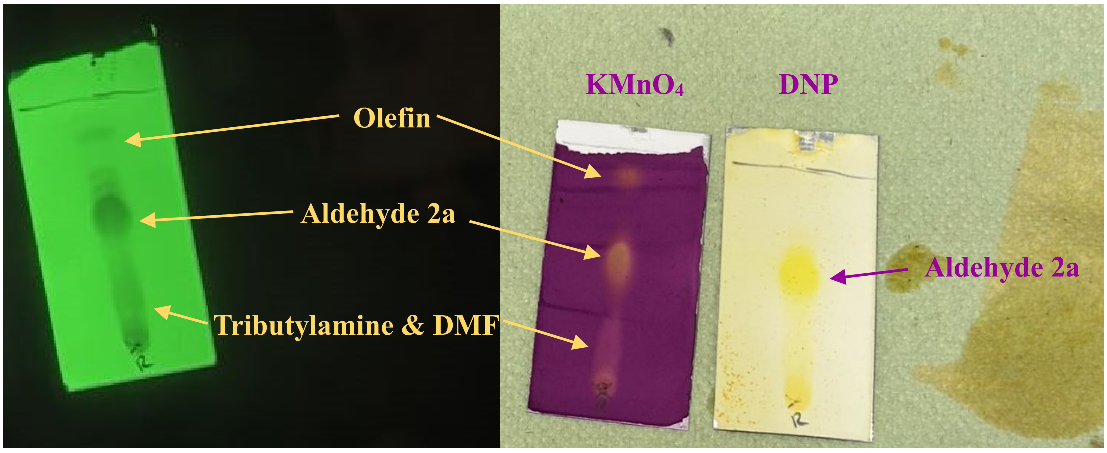

Column chromatography was performed using the following parameters:

Column: Biotage® Sfär Silica D;

Column volume: 15 mL;

Flowrate: 40 mL/min;

Equilibrate: 3 CV;

Eluent: petroleum ether/ethyl acetate: 100/0, 2 CV; from 100/0 to 95/5, 10 CV; 95/5, 5 CV;

UV threshold: 254 nm, 20 mAu;

Collection mode: collect all;

Note: Normally, product will flush out between 9 to 14 CV;

3.3 Troubleshooting & FAQ

Q1: What's the most common factor contributes to non-repeatability?

A1: The most common problem comes from the electrode side, especially from Magnesium. The quality of Magnesium is connected to the reactivity. Some Magnesium plates we purchased from Amazon (nominal 99.9% purity) can't perform the reaction at all. When using these Magnesium plates, grey or black dots can be spotted on the electrode, and the batch potential reached maximum 30V shortly after. We recommend using Goodfellow Magnesium (99.9% purity, 1 mm thickness) or IKA Magnesium electrodes.

Q2 My Argon balloon failed during the electrolysis, will it affect a lot on yield?

A2: We have experienced this problem for a few times when the vial was not sealed properly. This may affect the yield, but did not lead to complete obliteration of the reaction. However, purging and reacting under argon prior to adding the olefin is always recommended. Based on our experience, the reaction is more sensitive towards moisture. When the IKA vial is not dry enough or the humidity is simply too high, some precipitates will generate during the reaction, and the batch potential will raise dramatically at the very beginning.

Q3: What does a successful reaction looks like?

A3: At the beginning, the batch potential will stabilize at around 3.6-4.2V, if the potential is much lower than this limit, it's either due to reductive potential mismatch of starting material, or some contaminants within the vial. During the reaction, the potential will gradually raise up, and the mixture will turn cloudy and we believe it is due to generation of tributylamine or triethylamine. At the end of the reaction, the mixture usually looks brownish tea color (see graphical guide), grey solids can sometimes be observed floating inside the vial as well, but they will not affect the reaction.

Q4: What's the most common way to monitor the reaction?

A4: GC-MS is used to monitor the reaction, the size reduction of Magnesium electrode also might indicate the success. After quenching, the reaction can be monitored via TLC analysis. For aldehydes, stain with DNP or $KMnO_4$. For amines, stain with Dragendorff's reagent or I_2 with silica. For steroids or some long alkyl-chain substrates, stain with H_2SO_4 in ethanol and bake it with a heat gun.

Q5: What should I do if I don't have a 2mm-thick IKA Magnesium plates?

A5: It is not necessary to use a 2mm-thick IKA Magnesium plate, 1mm-thick plates can also be used. In order to fit them in the IKA electrode clamp, an additional 1mm-thick PTFE spacer can be put between the gap. However, it's recommended to wash them thoroughly following these sequences:

1. Sonicate the plate with addition of a few drops of 35% HCl in acetone for 10-15 min;

2. Polish the Magnesium plate after sonication until its shiny surface is repristinated;

3. Wash several times with water to remove all the acid and metal dust;

4. Wash with acetone again to remove water;

5. Oven dry the washed plates under 50 °C.

Note: The electrolysis usually fails if black or grey dots spotted during sonication and polishing.

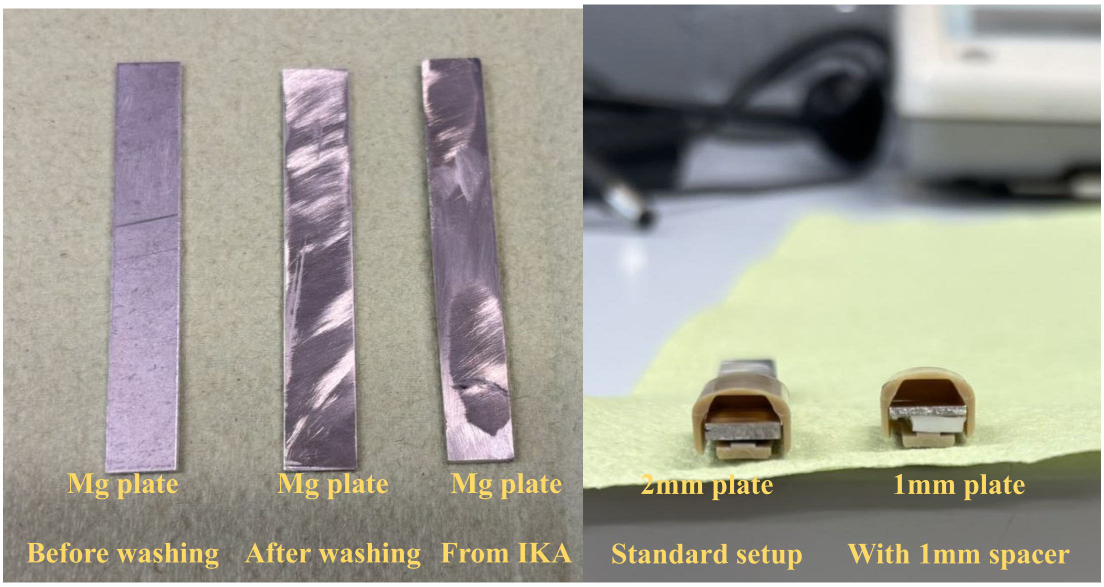

Q6: What's the mass balance of the reaction?

In general, this discrepancy is mainly due to decomposition of the starting material caused by prolonged electrolysis. Certain starting materials (e.g. **2s**, **2r**) could be recovered at the end of the reaction, as the reaction stalled and further electrolysis did not lead to any yield improvement. We tried to accommodate the reaction conditions to specific substrates, but this did not lead to any specific improvement. Notably, no reduction of the alkene has been observed as side product for the reaction.

3.4 General procedure 1: Electrochemical hydroformylation

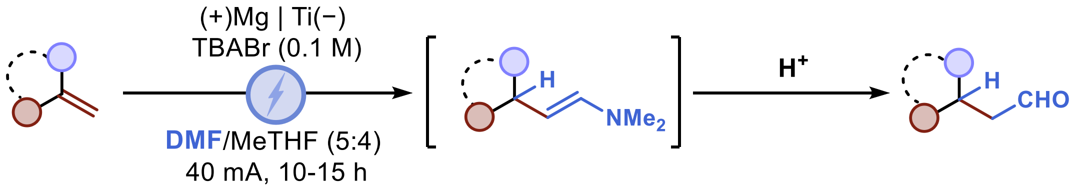

To an oven dried 10 mL ElectraSyn 2.0 vial was charged TBABr (1.00 equiv., 1.00 mmol, 322 mg),anhydrous DMF (5 mL), 2-MeTHF (4 mL) and a stirring bar, The vial was closed with an ElectraSyn 2.0 vial cap with a magnesium anode and a titanium cathode, then purged with argon for 5 minutes, the olefin (1.00 mmol or 0.50 mmol) was then added to the solution via syringe (if the olefin is a solid or a sticky oil it can be added before purging). Then the vial was placed on an IKA ElectraSyn 2.0 stirring plate and electrolysis was set to 40 mA, 1.00 mmol, 10 hours. The reaction underwent the programmed electrolysis under argon. After the reaction, the mixture was transferred to a separatory funnel, the electrodes were rinsed with Et_2O and water, then HCl (3.2 M) or citric acid (10 wt%) was added depending on the substrate's structure and the mixture was shaken vigorously. The aqueous layer was extracted 3 times with Et_2O. The combined organic layers were washed with brine, then dried over Na_2SO_4 before being concentrated by rotavap. The crude oil was purified via silica gel column chromatography to afford the desired product.

Note: For compounds whose preparation led to mixture of diastereoisomers, they were purified and used. In all cases, the same ratio of the starting material was observed after electrolysis.

Compound 2a

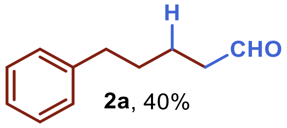

2a, 40%

Following the General Procedure 1 and quenched by HCl (3.2 M), the corresponding olefin (1.00 mmol, 132 mg) afforded 65 mg (40% isolated yield) of the titled compound **2a** as a colorless oil after purification by silica gel column chromatography (eluent: petroleum ether/ethyl acetate = 95/5, stained with DNP).

Physical Appearance: colorless oil

^{1}H NMR (300 MHz, $CDCl_3$): δ 9.66 (t, J = 1.7 Hz, 1H), 7.24 – 7.05 (m, 5H), 2.69 – 2.44 (m, 2H), 2.36 (m, 2H), 1.72 – 1.45 (m, 4H).

^{13}C NMR (75 MHz, $CDCl_3$): δ 202.7, 142.0, 128.5, 128.4, 125.9, 43.8, 35.7, 31.0, 21.8.

Characterization data for this aldehyde was in agreement with the literature.[8]

Compound 2b

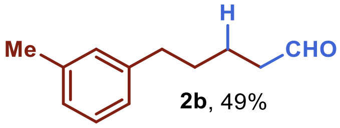

2b, 49%

Following the General Procedure 1 and quenched by HCl (3.2 M), the corresponding olefin (1.00 mmol, 146 mg) afforded 80 mg (49% isolated yield) of the titled compound **2b** as a colorless oil after purification by silica gel column chromatography (eluent: petroleum ether/ethyl acetate = 95/5, stained with DNP).

Physical Appearance: colorless oil

^{1}H NMR (300 MHz, $CDCl_3$): δ 9.76 (t, J = 1.8 Hz, 1H), 7.21 – 7.11 (m, 1H), 7.05 – 6.93 (m, 3H), 2.60 (m, 2H), 2.52 – 2.40 (m, 2H), 2.33 (s, 3H), 1.75 – 1.59 (m, 4H).

^{13}C NMR (75 MHz, $CDCl_3$): δ 202.8, 142.0, 138.0, 129.3, 128.4, 126.7, 125.5, 43.9, 35.7, 31.0, 21.9, 21.5.

HRMS (APCI-TOF) m/z: $[M + MeOH + H]^+$ Calcd for $C_{13}H_{21}O_2$ 209.1536; Found 209.1535.

Compound 2c

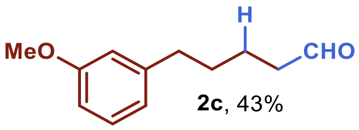

2c, 43%

Following the General Procedure 1 and quenched by HCl (3.2 M), the corresponding olefin (1.00 mmol, 162 mg) afforded 70 mg (43% isolated yield) of the titled compound **2c** as a colorless oil after purification by silica gel column chromatography (eluent: petroleum ether/ethyl acetate = 95/5, stained with DNP).

Physical Appearance: colorless oil

^{1}H NMR (300 MHz, $CDCl_3$): δ 9.75 (t, J = 1.7 Hz, 1H), 7.25 – 7.15 (m, 1H), 6.90 – 6.69 (m, 3H), 3.80 (s, 3H), 2.75 – 2.54 (m, 2H), 2.45 (m , 2H), 1.87 – 1.57 (m, 4H).

^{13}C NMR (75 MHz, $CDCl_3$): δ 202.6, 159.7, 143.7, 129.4, 120.9, 114.3, 111.1, 55.2, 43.8, 35.8, 30.8, 21.7.

Characterization data for this aldehyde was in agreement with the literature.[9]

Compound 2d

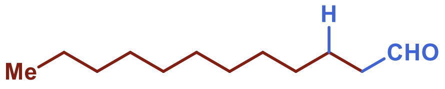

2d, 50%

Following the General Procedure 1 and quenched by HCl (3.2 M), the corresponding olefin (1.00 mmol, 154 mg) afforded 93 mg (50% isolated yield) of the titled compound **2d** as a colorless oil after purification by silica gel column chromatography (eluent: petroleum ether/ethyl acetate = 95/5, stained with DNP).

Physical Appearance: colorless oil

^{1}H NMR (300 MHz, $CDCl_3$): δ 9.72 (t, J = 1.9 Hz, 1H), 2.38 (td, J = 7.3, 1.8 Hz, 2H), 1.59 (t, J = 7.4 Hz, 2H), 1.28 – 1.19 (m, 16H), 0.89 – 0.79 (m, 3H).

^{13}C NMR (75 MHz, $CDCl_3$): δ 202.9, 44.0, 32.0, 29.7, 29.7, 29.5, 29.5, 29.4, 29.3, 22.8, 22.2, 14.2.

Characterization data for this aldehyde was in agreement with the literature.[10]

Compound 2e

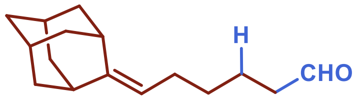

2e, 37%

Following the General Procedure 1 and quenched by HCl (3.2 M), the corresponding olefin (1.00 mmol, 202 mg) afforded 87 mg (37% isolated yield) of the titled compound **2e** as a colorless oil after purification by silica gel column chromatography (eluent: petroleum ether/ethyl acetate = 20/1, stained with DNP).

Physical Appearance: colorless oil

^{1}H NMR (300 MHz, $CDCl_3$): δ 9.73 (t, J = 1.9 Hz, 1H), 4.98 (t, J = 7.3 Hz, 1H), 2.79 – 2.73 (m, 1H), 2.40 (td, J = 7.4, 1.9 Hz, 2H), 2.31 – 2.25 (m, 1H), 2.03 – 1.88 (m, 4H), 1.88 – 1.76 (m, 6H), 1.76 – 1.58 (m, 6H), 1.42 – 1.26 (m, 2H).

^{13}C NMR (75 MHz, $CDCl_3$): δ 202.9, 148.1, 115.6, 43.9, 40.6, 39.9, 39.0, 37.4, 32.2, 29.9, 28.7, 26.2, 21.7.

HRMS (ESI-TOF) m/z: $[M + H]^+$ Calcd for $C_{16}H_{25}O$ 233.1900; Found 233.1860.

Compound 2f

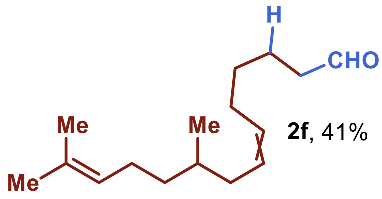

2f, 41%

Following the General Procedure 1 and quenched by HCl (3.2 M), the corresponding olefin (1.00 mmol, 206 mg) afforded 107 mg (41% isolated yield) of the titled compound **2f** as a colorless oil (mixture of E/Z isomers) after purification by silica gel column chromatography (eluent: petroleum ether/ethyl acetate = 20/1, stained with DNP).

Physical Appearance: colorless oil

^{1}H NMR (300 MHz, $CDCl_3$): δ 9.73 (t, J = 1.8 Hz, 1H), 5.43 – 5.31 (m, 2H), 5.19 – 5.02 (m, 1H), 2.50 – 2.31 (m, 2H), 2.14 – 1.78 (m, 6H), 1.73 – 1.54 (m, 8H), 1.51 – 1.25 (m, 4H), 1.21 – 1.05 (m, 1H), 0.85 (d, J = 6.6 Hz, 3H).

^{13}C NMR (75 MHz, $CDCl_3$): δ 202.6, 131.1, 129.8, 129.1, 124.9, 43.9, 36.8, 34.5, 33.1, 29.3, 27.1, 25.8, 25.7, 21.8, 19.6, 17.7.

HRMS (ESI-TOF) m/z: $[M + H]^+$ Calcd for $C_{16}H_{29}O$ 237.2213; Found 237.2215.

Compound 2g

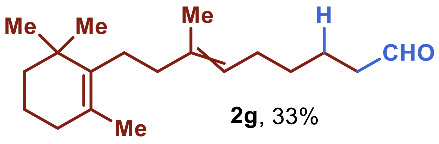

2g, 33%

Following the General Procedure 1 and quenched by HCl (3.2 M), the corresponding olefin (1.00 mmol, 262 mg) afforded 92 mg (33% isolated yield, mixture of Z/E isomers) of the titled compound **2g** as a colorless oil after purification by silica gel column chromatography (eluent: petroleum ether/ethyl acetate = 9/1, stained with DNP).

Physical Appearance: colorless oil

^{1}H NMR (300 MHz, CDCl$_3$): δ 9.76 – 9.72 (m, 1H), 5.18 – 5.00 (m, 1H), 2.49 – 2.32 (m, 2H), 2.11 – 1.83 (m, 9H), 1.78 – 1.49 (m, 11H), 1.47 – 1.38 (m, 2H), 1.06 – 0.93 (m, 6H).

^{13}C NMR (75 MHz, CDCl$_3$): δ 202.8, 202.7, 137.2, 137.2, 136.6, 136.6, 127.2, 127.0, 124.2, 123.3, 43.9, 40.4, 40.0, 39.9, 35.1, 35.0, 32.9, 32.9, 32.7, 29.8, 29.5, 28.7, 28.7, 28.0, 27.8, 27.7, 27.5, 23.4, 21.9, 21.8, 19.9, 19.9, 19.7, 19.6, 16.2.

Compound 2h

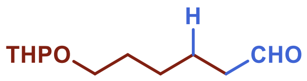

2h, 41%

Following the General Procedure 1 and quenched by citric acid (10 wt%), the corresponding olefin (1.00 mmol, 170 mg) afforded 82 mg (41% isolated yield) of the titled compound **2h** as a colorless oil after purification by silica gel column chromatography (eluent: petroleum ether/ethyl acetate = 3/1, stained with DNP).

Physical Appearance: colorless oil

^{1}H NMR (300 MHz, $CDCl_3$): δ 9.71 – 9.68 (m, 1H), 4.58 – 4.42 (m, 1H), 3.98 – 3.59 (m, 2H), 3.54 – 3.23 (m, 2H), 2.38 (t, *J* = 7.3 Hz, 2H), 1.91 – 1.27 (m, 12H).

^{13}C NMR (75 MHz, $CDCl_3$): δ 202.6, 98.9, 67.2, 62.3, 43.8, 30.7, 29.5, 25.9, 25.5, 21.9, 19.7.

Characterization data for this aldehyde was in agreement with the literature.[11]

Compound 2i

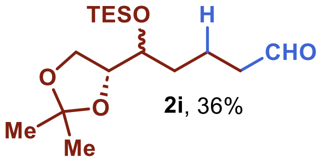

2i, 36%

Following the General Procedure 1 and quenched by citric acid (10 wt%), the corresponding olefin (1.00 mmol, 286 mg) afforded 113 mg (36% isolated yield) of the titled compound **2i** (mixture of 2 diastereomers) as a colorless oil after purification by silica gel column chromatography (eluent: petroleum ether/ethyl acetate = 9/1, stained with DNP).

Physical Appearance: colorless oil

^{1}H NMR (300 MHz, $CDCl_3$): δ 9.75 – 9.70 (m, 1H), 4.14 – 3.86 (m, 2H), 3.83 – 3.59 (m, 2H), 2.56 – 2.25 (m, 2H), 1.89 – 1.25 (m, 10H), 0.92 (t, J = 7.9 Hz, 9H), 0.71 – 0.50 (m, 6H).

^{13}C NMR (75 MHz, $CDCl_3$): δ 202.4, 202.3, 109.2, 109.2, 78.7, 78.1, 73.1, 72.6, 66.9, 65.6, 44.1, 43.9, 34.0, 32.0, 26.8, 26.4, 25.4, 25.2, 18.4, 17.1, 6.9, 6.9, 5.2, 5.2.

HRMS (ESI-TOF) m/z: $[M + Na]^+$ Calcd for $C_{16}H_{32}NaO_4Si$ 339.1962; Found 339.1965.

Compound 2j

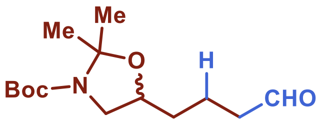

2j, 42%

Following the General Procedure 1 and quenched by citric acid (10 wt%), the corresponding olefin (1.00 mmol, 241 mg) afforded 115 mg (42% isolated yield) of the titled compound **2j** as a colorless oil after purification by silica gel column chromatography (eluent: petroleum ether/ethyl acetate = 4/1, stained with DNP).

Physical Appearance: colorless oil

^{1}H NMR (300 MHz, $CDCl_3$): δ 9.74 – 9.71 (m, 1H), 4.05 – 3.91 (m, 1H), 3.75 – 3.51 (m, 1H), 3.06 – 2.89 (m, 1H), 2.53 – 2.36 (m, 2H), 1.86 – 1.26 (m, 19H).

^{13}C NMR (75 MHz, $CDCl_3$): δ 202.1, 152.3, 151.9, 93.5, 93.0, 80.1, 79.5, 73.4, 73.2, 50.8, 43.6, 32.5, 28.5, 27.3, 26.3, 25.2, 24.3, 18.3.

HRMS (ESI-TOF) m/z: $[M + Na]^+$ Calcd for $C_{14}H_{25}NNaO_4$ 294.1676; Found 294.1684.

Compound 2k

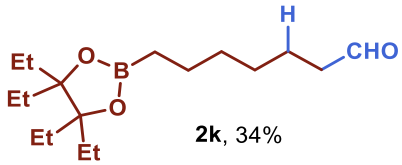

2k, 34%

Following the General Procedure 1 and quenched by citric acid (10 wt%), the corresponding olefin (1.00 mmol, 266 mg) afforded 99 mg (34% isolated yield) of the titled compound **2k** as a colorless oil after purification by silica gel column chromatography (eluent: petroleum ether/ethyl acetate = 10/1, stained with DNP).

Physical Appearance: colorless oil

^{1}H NMR (300 MHz, $CDCl_3$): δ 9.72 (t, J = 1.9 Hz, 1H), 2.37 (td, J = 7.4, 1.9 Hz, 2H), 1.69 – 1.52 (m, 10H), 1.43 – 1.22 (m, 6H), 0.92 – 0.66 (m, 14H).

^{13}C NMR (75 MHz, $CDCl_3$): δ 203.0, 87.9, 44.0, 32.1, 29.0, 26.4, 24.0, 22.1, 11.3, 8.9.

HRMS (ESI-TOF) m/z: $[M + H]^+$ Calcd for $C_{17}H_{34}BO_3$ 297.2596; Found 297.2586.

Compound 2l

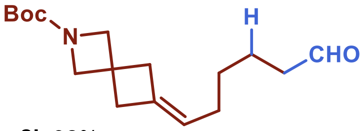

2l, 32%

Following the General Procedure 1 and quenched by citric acid (10 wt%), the corresponding olefin (1.00 mmol, 263 mg) afforded 92 mg (32% isolated yield) of the titled compound **2l** as a colorless oil after purification by silica gel column chromatography (eluent: petroleum ether/ethyl acetate = 10/1, stained with DNP).

Physical Appearance: colorless oil

^{1}H NMR (300 MHz, $CDCl_3$): δ 9.71 (t, *J* = 1.8 Hz, 1H), 5.08 (tt, *J* = 7.3, 2.4 Hz, 1H), 3.96 – 3.76 (m, 4H), 2.74 (s, 4H), 2.37 (td, *J* = 7.3, 1.8 Hz, 2H), 1.93 – 1.76 (m, 2H), 1.69 – 1.49 (m, 2H), 1.38 (s, 9H), 1.36 – 1.24 (m, 2H).

^{13}C NMR (75 MHz, $CDCl_3$): δ 202.6, 156.3, 132.6, 122.0, 79.3, 61.4, 43.8, 42.0, 40.5, 33.5, 29.0, 28.4, 28.0, 21.6.

HRMS (ESI-TOF) m/z: $[M + Na]^+$ Calcd for $C_{17}H_{27}NNaO_3$ 316.1883; Found 316.1896.

Compound 2m

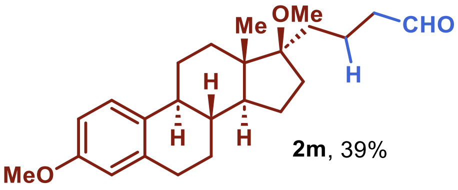

2m, 39%

Following the General Procedure 1 but extending the electrolysis time overnight and quenched by citric acid (10 wt%), the corresponding olefin (0.50 mmol, 170 mg) afforded 73 mg (39% isolated yield) of the titled compound **2m** as a white solid after purification by silica gel column chromatography (eluent: petroleum ether/ethyl acetate = 5/1, stained with DNP).

Physical Appearance: white solid

^{1}H NMR (300 MHz, $CDCl_3$): δ 9.82 (t, J = 1.7 Hz, 1H), 7.19 (d, J = 8.6 Hz, 1H), 6.71 (dd, J = 8.5, 2.8 Hz, 1H), 6.63 (d, J = 2.8 Hz, 1H), 3.78 (s, 3H), 3.23 (s, 3H), 2.91 – 2.80 (m, 2H), 2.52 (td, J = 7.0, 1.7 Hz, 2H), 2.36 – 2.20 (m, 1H), 2.19 – 2.08 (m, 1H), 2.07 – 1.25 (m, 15H), 0.96 (s, 3H).

^{13}C NMR (75 MHz, $CDCl_3$): δ 202.7, 157.5, 138.0, 132.6, 126.3, 113.8, 111.5, 87.7, 55.3, 51.4, 50.9, 47.1, 44.4, 43.6, 39.4, 34.7, 33.9, 30.2, 29.9, 27.5, 26.6, 23.3, 16.1, 13.9.

HRMS (ESI-TOF) m/z: $[M + K]^+$ Calcd for $C_{24}H_{34}KO_3$ 409.2140; Found 409.2193.

Compound 2n

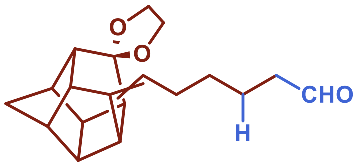

2n, 54%

Following the General Procedure 1 and quenched by citric acid (10 wt%), the corresponding olefin (0.38 mmol, 102 mg) afforded 61 mg (54% isolated yield, mixture of Z/E isomers) of the titled compound **2n** as a yellow oil after purification by silica gel column chromatography (eluent: petroleum ether/ethyl acetate = 5/1, stained with DNP).

Physical Appearance: yellow oil

^{1}H NMR (300 MHz, $CDCl_3$): δ 9.73 (t, J = 2.0 Hz, 1H), 5.20 – 4.96 (m, 1H), 3.94 – 3.66 (m, 4H), 3.17 – 3.06 (m, 1H), 2.75 – 2.52 (m, 4H), 2.49 – 2.32 (m, 3H), 2.28 – 2.12 (m, 2H), 2.08 – 1.85 (m, 2H), 1.78 – 1.49 (m, 3H), 1.46 – 1.18 (m, 3H).

^{13}C NMR (75 MHz, $CDCl_3$): δ ^{13}C NMR (75 MHz, $CDCl_3$) δ 203.1, 144.2, 116.8, 116.2, 65.5, 63.0, 50.6, 49.6, 46.8, 44.9, 44.0, 42.3, 41.8, 40.0, 36.3, 35.1, 29.5, 28.5, 21.6.

HRMS (ESI-TOF) m/z: $[M + H]^+$ Calcd for $C_{19}H_{25}O_3$ 301.1798; Found 301.1801.

Compound 2o

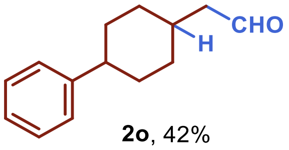

2o, 42%

Following the General Procedure 1 and quenched by HCl (3.2 M), the corresponding olefin (1.00 mmol, 172 mg) afforded 85 mg (42% isolated yield) of the titled compound **2o** as a colorless oil after purification by silica gel column chromatography (eluent: petroleum ether/ethyl acetate = 10/1, stained with DNP).

Physical Appearance: colorless oil

^{1}H NMR (300 MHz, $CDCl_3$): δ 9.72 (t, J = 2.2 Hz, 1H), 7.29 – 7.03 (m, 5H), 2.52 – 2.22 (m, 3H), 1.97 – 1.75 (m, 4H), 1.71 – 1.36 (m, 3H), 1.24 – 1.02 (m, 2H).

^{13}C NMR (75 MHz, $CDCl_3$): δ 202.8, 147.3, 128.5, 126.9, 126.1, 51.4, 44.1, 34.0, 33.5, 32.4.

HRMS (ESI-TOF) m/z: $[M + H]^+$ Calcd for $C_{14}H_{19}O$ 203.1430; Found 203.1426.

Compound 2p

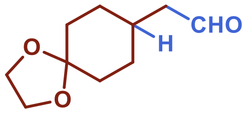

2p, 32%

Following the General Procedure 1 and quenched by citric acid (10 wt%), the corresponding olefin (1.00 mmol, 154 mg) afforded 60 mg (32% isolated yield) of the titled compound **2p** as a colorless oil after purification by silica gel column chromatography (eluent: petroleum ether/ethyl acetate = 5/1, stained with DNP).

Physical Appearance: colorless oil

^{1}H NMR (300 MHz, $CDCl_3$): δ 9.74 (t, J = 2.0 Hz, 1H), 3.95 – 3.88 (m, 4H), 2.33 (dd, J = 6.8, 2.1 Hz, 2H), 2.00 – 1.83 (m, 1H), 1.78 – 1.65 (m, 4H), 1.63 – 1.47 (m, 2H), 1.43 – 1.26 (m, 1H), 0.88 – 0.81 (m, 1H).

^{13}C NMR (75 MHz, $CDCl_3$): δ 202.3, 108.5, 64.3, 50.3, 34.4, 31.2, 30.2.

HRMS (ESI-TOF) m/z: $[M + Na]^+$ Calcd for $C_{10}H_{16}NaO_3$ 207.0992; Found 207.0984.

Compound 2q

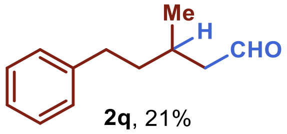

2q, 21%

Following the General Procedure 1 and quenched by HCl (3.2 M), the corresponding olefin (1.00 mmol, 146 mg) afforded 37 mg (21% isolated yield) of the titled compound **2q** as a colorless oil after purification by silica gel column chromatography (eluent: petroleum ether/ethyl acetate = 9/1, stained with DNP).

Physical Appearance: colorless oil

^{1}H NMR (300 MHz, $CDCl_3$): δ 9.66 (t, J = 2.3 Hz, 1H), 7.26 – 7.02 (m, 5H), 2.67 – 2.48 (m, 2H), 2.43 – 2.30 (m, 1H), 2.26 – 2.14 (m, 1H), 2.11 – 1.94 (m, 1H), 1.67 – 1.55 (m, 1H), 1.53 – 1.40 (m, 1H), 0.95 (d, J = 6.6 Hz, 3H).

^{13}C NMR (75 MHz, $CDCl_3$): δ 202.9, 142.2, 128.5, 128.4, 126.0, 51.1, 38.7, 33.4, 27.9, 19.9.

Compound 2r

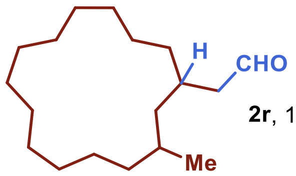

2r, 15%

Following the General Procedure 1 and quenched by HCl (3.2 M), the corresponding olefin (1.00 mmol, 236 mg) afforded 41 mg (15% isolated yield, 44% brsm yield.) of the titled compound **2r** (mixture of 2 diastereomers) as a colorless oil and 157 mg (66% isolated yield) of starting material after purification by silica gel column chromatography (eluent: petroleum ether/ethyl acetate = 20/1, stained with DNP).

Physical Appearance: colorless oil

^{1}H NMR (300 MHz, CDCl$_3$): δ 9.74 (t, J = 2.5 Hz, 1H), 2.50 – 1.98 (m, 3H), 1.68 – 1.00 (m, 26H), 0.97 – 0.79 (m, 4H).

^{13}C NMR (75 MHz, CDCl$_3$): δ 203.6, 203.5, 49.9, 49.8, 43.9, 41.2, 36.1, 33.7, 33.1, 32.2, 30.3, 30.0, 29.5, 29.1, 27.7, 27.4, 27.3, 27.3, 27.0, 26.9, 26.8, 26.8, 26.7, 26.7, 26.7, 26.7, 26.6, 26.5, 26.4, 25.5, 25.4, 24.6, 23.5, 21.3, 20.4.

HRMS (ESI-TOF) m/z: [M + H]$^+$ Calcd for $C_{18}H_{35}O$ 267.2682; Found 267.2681.

Compound 2s

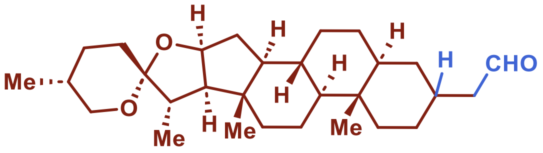

2s, 25%

Following the General Procedure 1 but extending the electrolysis time overnight and quenched by citric acid (10 wt%), the corresponding olefin (0.50 mmol, 206 mg) afforded 55 mg (25% isolated yield, 37% brsm yield) of the titled compound **2s** as a white solid and 67 mg (32% isolated yield) of starting material **1s** after purification by silica gel column chromatography (eluent: petroleum ether/ethyl acetate = 20/1, stained with H_2SO_4 in EtOH).

Physical Appearance: white solid

^{1}H NMR (300 MHz, $CDCl_3$): δ 9.74 (t, J = 2.3 Hz, 1H), 4.48 – 4.32 (m, 1H), 3.54 – 3.29 (m, 2H), 2.28 (dd, J = 6.8, 2.4 Hz, 2H), 2.06 – 1.80 (m, 3H), 1.79 – 1.43 (m, 12H), 1.34 – 1.02 (m, 11H), 1.00 – 0.88 (m, 4H), 0.83 – 0.60 (m, 10H).

^{13}C NMR (75 MHz, $CDCl_3$): δ 203.1, 109.4, 81.0, 67.0, 62.3, 56.5, 54.6, 51.4, 46.6, 41.7, 40.7, 40.2, 38.4, 36.0, 35.6, 35.2, 33.2, 32.3, 31.9, 31.5, 30.4, 28.9, 28.9, 28.8, 20.9, 17.3, 16.6, 14.6, 12.4.

HRMS (ESI-TOF) m/z: $[M + H]^+$ Calcd for $C_{29}H_{47}O_3$ 443.3520; Found 443.3538.

Compound 2t

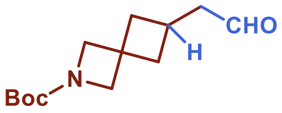

2t, 54%

Following the General Procedure 1 and quenched by citric acid (10 wt%), the corresponding olefin (1.00 mmol, 209 mg) afforded 128 mg (54% isolated yield) of the titled compound **2t** as a colorless oil after purification by silica gel column chromatography (eluent: petroleum ether/ethyl acetate = 5/1, stained with DNP).

Physical Appearance: colorless oil

^{1}H NMR (300 MHz, $CDCl_3$): δ 9.64 (t, J = 1.5 Hz, 1H), 3.89 (s, 2H), 3.75 (s, 2H), 2.65 – 2.44 (m, 3H), 2.39 – 2.27 (m, 2H), 1.94 – 1.74 (m, 2H), 1.37 (s, 9H).

^{13}C NMR (75 MHz, $CDCl_3$): δ 201.2, 156.2, 79.3, 62.3, 60.7, 50.4, 39.0, 34.9, 28.4, 23.9.

HRMS (ESI-TOF) m/z: $[M + H]^+$ Calcd for $C_{13}H_{22}NO_3$ 240.1594; Found 240.1603.

Compound 2u

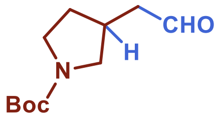

2u, 58%

Following the General Procedure 1 and DMF (10 mL) and 2-MeTHF (8 mL) was added and quenched by citric acid (10 wt%), the corresponding olefin (5.00 mmol, 916 mg) afforded 617 mg (58% isolated yield) of the titled compound **2u** as a slightly yellow oil after purification by silica gel column chromatography (eluent: petroleum ether/ethyl acetate = 5/1, stained with DNP).

Physical Appearance: yellow oil

^{1}H NMR (300 MHz, $CDCl_3$): δ 9.77 (s, 1H), 3.77 – 3.19 (m, 3H), 2.89 (m, 1H), 2.56 (m, 3H), 2.13 (m, 1H), 1.44 (m, 10H).

^{13}C NMR (75 MHz, $CDCl_3$): δ 200.8, 154.6, 79.4, 51.2, 50.8, 47.4, 47.3, 45.4, 45.0, 33.0, 32.2, 31.6, 30.9, 28.6.

HRMS (ESI-TOF) m/z: $[M + Na]^+$ Calcd for $C_{11}H_{19}NNaO_3$ 236.1257; Found 236.1263.

Compound 2v

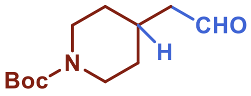

2v, 60%

Following the General Procedure 1 and DMF (10 mL) and 2-MeTHF (8 mL) was added and quenched by citric acid (10 wt%), the corresponding olefin (5.00 mmol, 986 mg) afforded 698 mg (60% isolated yield) of the titled compound **2v** as a colorless oil after purification by silica gel column chromatography (eluent: petroleum ether/ethyl acetate = 5/1, stained with DNP).

Physical Appearance: colorless oil

^{1}H NMR (300 MHz, $CDCl_3$): δ 9.67 (t, J = 1.7 Hz, 1H), 3.97 (m, 2H), 2.64 (m, 2H), 2.29 (m, 2H), 2.09 – 1.84 (m, 1H), 1.59 (m, 2H), 1.34 (s, 9H), 1.07 (m, 2H).

^{13}C NMR (75 MHz, $CDCl_3$): δ 201.4, 154.7, 79.3, 50.2, 43.7, 31.8, 30.6, 28.3.

Characterization data for this aldehyde was in agreement with the literature.[12]

Compound 2w

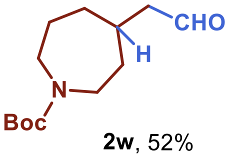

2w, 52%

Following the General Procedure 1 and quenched by citric acid (10 wt%), the corresponding olefin (1.00 mmol, 211 mg) afforded 126 mg (52% isolated yield) of the titled compound **2w** as a colorless oil after purification by silica gel column chromatography (eluent: petroleum ether/ethyl acetate = 5/1, stained with DNP).

Physical Appearance: colorless oil

^{1}H NMR (300 MHz, $CDCl_3$): δ 9.74 – 9.62 (m, 1H), 4.00 – 2.94 (m, 4H), 2.49 – 2.17 (m, 2H), 2.16 – 1.90 (m, 1H), 1.88 – 1.70 (m, 2H), 1.68 – 1.48 (m, 2H), 1.45 – 1.08 (m, 11H)

^{13}C NMR (75 MHz, $CDCl_3$): δ 202.0, 201.9, 155.5, 79.1, 51.2, 51.1, 46.7, 46.0, 45.0, 44.6, 34.9, 34.8, 33.8, 33.5, 33.3, 33.1, 28.5, 26.9, 26.8.

HRMS (ESI-TOF) m/z: $[M + Na]^+$ Calcd for $C_{13}H_{23}NNaO_3$ 264.1570; Found 264.1576.

Compound 2x

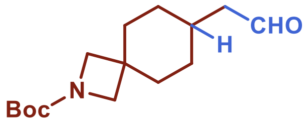

2x, 38%

Following the General Procedure 1 and quenched by citric acid (10 wt%), the corresponding olefin (1.00 mmol, 237 mg) afforded 102 mg (38% isolated yield) of the titled compound **2x** as a colorless oil after purification by silica gel column chromatography (eluent: petroleum ether/ethyl acetate = 5/1, stained with DNP).

Physical Appearance: colorless oil

^{1}H NMR (300 MHz, $CDCl_3$): δ 9.67 (t, J = 2.0 Hz, 1H), 3.50 (s, 2H), 3.46 (s, 2H), 2.23 (dd, J = 6.8, 2.0 Hz, 2H), 1.88 – 1.71 (m, 3H), 1.67 – 1.55 (m, 2H), 1.47 – 1.29 (m, 11H), 1.00 – 0.85 (m, 2H).

^{13}C NMR (75 MHz, $CDCl_3$): δ 202.0, 156.5, 79.1, 59.2, 50.5, 35.3, 34.5, 31.1, 29.2, 28.4.

HRMS (ESI-TOF) m/z: $[M + Na]^+$ Calcd for $C_{15}H_{25}NNaO_3$ 290.1727; Found 290.1735.

Compound 2y

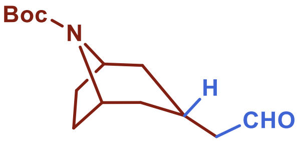

2y, 64%

Following the General Procedure 1 and quenched by citric acid (10 wt%), the corresponding olefin (1.00 mmol, 223 mg) afforded 162 mg (64% isolated yield) of the titled compound **2y** as a colorless oil after purification by silica gel column chromatography (eluent: petroleum ether/ethyl acetate = 5/1, stained with DNP).

Physical Appearance: colorless oil

^{1}H NMR (300 MHz, $CDCl_3$): δ 9.66 (t, J = 1.8 Hz, 1H), 4.39 – 3.74 (m, 2H), 2.45 – 2.28 (m, 1H), 2.29 – 2.18 (m, 2H), 2.01 – 1.81 (m, 2H), 1.69 – 1.46 (m, 4H), 1.37 (s, 9H).

^{13}C NMR (75 MHz, $CDCl_3$): δ 201.6, 153.3, 79.1, 53.6, 52.9, 50.6, 37.6, 36.9, 28.4, 28.3, 27.7, 23.5.

HRMS (ESI-TOF) m/z: $[M + Na]^+$ Calcd for $C_{14}H_{23}NNaO_3$ 276.1570; Found 276.1571.

3.5 General procedure 2: Electrochemical amine synthesis

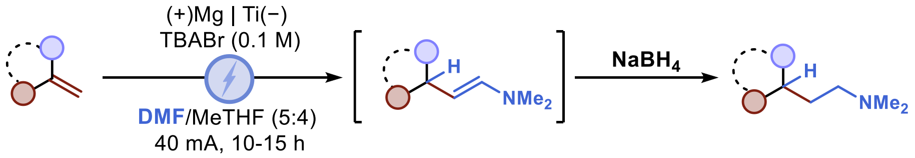

To an oven dried 10 mL ElectraSyn 2.0 vial was charged TEABr (1.00 equiv., 1.00 mmol, 322 mg),anhydrous DMF (5 mL), 2-MeTHF (4 mL) and a stirring bar, The vial was closed with an ElectraSyn 2.0 vial cap with a magnesium anode and a titanium cathode, then purged with argon for 5 minutes, the olefin (1.00 mmol) was then added to the solution via syringe (if the olefin is a solid or a sticky oil it can be added before purging). Then the vial was placed on an IKA ElectraSyn 2.0 stirring plate and electrolysis was set to 40 mA, 1.00 mmol, 10 hours. The reaction underwent the programmed electrolysis under argon. After the reaction, the mixture was transferred to 100 mL round bottom flask and MeOH (10 mL) was added. Then $NaBH_4$ (5.00 equiv., 5.00 mmol, 189 mg) was added and stirred for 2 hours. The mixture was then concentrated via rotavap to remove most of MeOH within, then was quenched with saturated NH_4Cl and extracted 3 times with DCM, the organic layers were combined and washed again with saturated LiCl, dried over Na_2SO_4 then concentrated via rotavap and purified via silica gel column chromatography to afford the amine.

Compound 3a

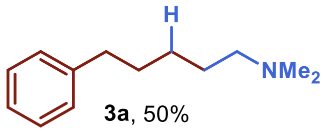

3a, 50%

Following the General Procedure 2, the corresponding olefin (1.00 mmol, 132 mg) afforded 96 mg (50% isolated yield) of the titled compound **3a** as a yellow oil after purification by silica gel column chromatography (eluent: petroleum ether/ethyl acetate/triethylamine = 11/18/1, stained with Dragendorff's reagent).

Physical Appearance: yellow oil.

^{1}H NMR (300 MHz, $CDCl_3$): δ 7.24 – 7.07 (m, 5H), 2.53 (t, J = 7.7 Hz, 2H), 2.18 – 2.13 (m, 8H), 1.61 – 1.51 (m, 2H), 1.44 – 1.39 (m, 2H), 1.31 – 1.23 (m, 2H).

^{13}C NMR (75 MHz, $CDCl_3$): δ 142.7, 128.4, 128.3, 125.6, 59.9, 45.5, 36.0, 31.5, 27.7, 27.2.

Characterization data for this amine was in agreement with the literature.[13]

Compound 3c

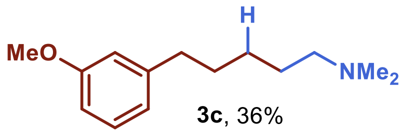

3c, 36%

Following the General Procedure 2, the corresponding olefin (1.00 mmol, 162 mg) afforded 83 mg (36% isolated yield) of the titled compound **3c** as a colorless oil after purification by silica gel column chromatography (eluent: ethyl acetate /petroleum ether/triethylamine = 11/8/1, stained with Dragendorff's reagent).

Physical Appearance: colorless oil

^{1}H NMR (300 MHz, $CDCl_3$): δ 7.14– 7.08 (m, 1H), 6.71 – 6.62 (m, 3H), 3.72 (s, 3H), 2.54 – 2.47 (m, 2H), 2.18 – 2.13 (m, 8H), 1.61 – 1.51 (m, 2H), 1.47 – 1.37 (m, 2H), 1.32 – 1.24 (m, 2H).

^{13}C NMR (75 MHz, $CDCl_3$): δ 159.7, 144.5, 129.3, 120.9, 114.3, 110.9, 59.9, 55.2, 45.6, 36.1, 31.5, 27.8, 27.3.

HRMS (ESI-TOF) m/z: $[M + H]^+$ Calcd for $C_{14}H_{24}NO$ 222.1852; Found 222.1861.

Compound 3d

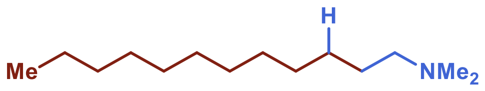

3d, 71%

Following the General Procedure 2, the corresponding olefin (1.00 mmol, 154 mg) afforded 151 mg (71% isolated yield) of the titled compound **3d** as a yellow oil after purification by silica gel column chromatography (eluent: DCM/MeOH/25% NH_3 (aq.) = 17/1/2, stained with Dragendorff's reagent).

Physical Appearance: yellow oil

^{1}H NMR (300 MHz, $CDCl_3$): δ 2.31 – 2.20 (m, 8H), 1.49 – 1.39 (m, 2H), 1.22 – 1.19 (m, 16H), 0.88 – 0.78 (m, 3H).

^{13}C NMR (75 MHz, $CDCl_3$): δ 59.7, 45.0, 31.9, 29.7, 29.7, 29.6, 29.6, 29.6, 29.4, 27.4, 27.16, 22.7, 14.1.

Characterization data for this amine was in agreement with the literature.[14]

Compound 3e

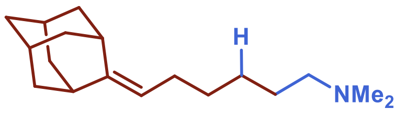

3e, 64%

Following the General Procedure 2, the corresponding olefin (1.00 mmol, 202 mg) afforded 166 mg (64% isolated yield) of the titled compound **3e** as a yellow oil after purification by silica gel column chromatography (eluent: DCM/MeOH/25% NH_3 (aq.) = 17/1/2, stained with Dragendorff's reagent).

Physical Appearance: yellow oil

^{1}H NMR (300 MHz, $CDCl_3$): δ 5.00 (t, *J* = 7.3 Hz, 1H), 2.78 (t, *J* = 3.3 Hz, 1H), 2.28 – 2.21 (m, 8H), 1.96 – 1.64 (m, 14H), 1.49 – 1.25 (m, 7H).

^{13}C NMR (75 MHz, $CDCl_3$): δ 147.4, 116.2, 59.9, 45.4, 40.5, 40.0, 38.9, 37.3, 32.0, 30.1, 28.7, 27.5, 27.0, 26.4.

HRMS (ESI-TOF) m/z: $[M + H]^+$ Calcd for $C_{18}H_{32}N$ 262.2529; Found 262.2558.

Compound 3h

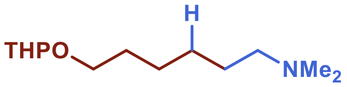

3h, 51%

Following the General Procedure 2, the corresponding olefin (1.00 mmol, 170 mg) afforded 116 mg (51% isolated yield) of the titled compound **3h** as a colorless oil after purification by silica gel column chromatography (eluent: DCM/MeOH/25% NH_3 (aq.) = 17/1/2, stained with Dragendorff's reagent).

Physical Appearance: colorless oil

^{1}H NMR (300 MHz, $CDCl_3$): δ 4.48 – 4.45 (m, 1H), 3.80 – 3.73 (m, 1H), 3.67 – 3.59 (m, 1H), 3.43 – 3.36 (m, 1H), 3.32 – 3.24 (m, 1H), 2.22 – 2.15 (m, 8H), 1.74 – 1.25 (m, 14H).

^{13}C NMR (75 MHz, $CDCl_3$): δ 98.8, 67.5, 62.3, 59.7, 45.2, 30.7, 29.6, 27.4, 27.2, 26.2, 25.5, 19.6.

HRMS (ESI-TOF) m/z: $[M + H]^+$ Calcd for $C_{13}H_{28}NO_2$ 230.2115; Found 230.2133.

Compound 3o

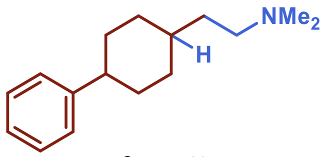

3o, 35%

Following the General Procedure 2, the corresponding olefin (1.00 mmol, 172 mg) afforded 82 mg (35% isolated yield) of the titled compound **3o** as a colorless oil after purification by silica gel column chromatography (eluent: DCM/MeOH/25% NH_3 (aq.) = 17/1/2, stained with Dragendorff's reagent).

Physical Appearance: colorless oil

^{1}H NMR (300 MHz, $CDCl_3$): δ 7.23 – 7.09 (m, 5H), 2.41 – 2.29 (m, 8H), 1.85 – 1.78 (m, 4H), 1.62 – 1.57 (m, 1H), 1.47 – 1.33 (m, 5H), 1.11 – 0.97 (m, 2H).

^{13}C NMR (75 MHz, $CDCl_3$): δ 147.6, 128.4, 126.9, 125.9, 57.4, 45.0, 44.5, 35.6, 34.4, 34.2, 33.6.

HRMS (ESI-TOF) m/z: $[M + H]^+$ Calcd for $C_{16}H_{26}N$ 232.2060; Found 232.2081.

Compound 3p

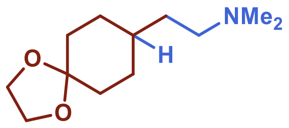

3p, 37%

Following the General Procedure 2, the corresponding olefin (1.00 mmol, 154 mg) afforded 78 mg (37% isolated yield) of the titled compound **3p** as a colorless oil after purification by silica gel column chromatography (eluent: DCM/MeOH/25% NH_3 (aq.) = 17/1/2, stained with Dragendorff's reagent).

Physical Appearance: colorless oil

^{1}H NMR (300 MHz, $CDCl_3$): δ 3.87 (s, 1H), 2.23 – 2.14 (m, 8H), 1.67 – 1.20 (m, 11H).

^{13}C NMR (75 MHz, $CDCl_3$): δ 109.1, 64.2, 64.2, 57.8, 45.6, 34.5, 34.5, 34.1, 30.3.

HRMS (ESI-TOF) m/z: $[M + H]^+$ Calcd for $C_{12}H_{24}NO_2$ 214.1802; Found 214.1819.

Compound 3t

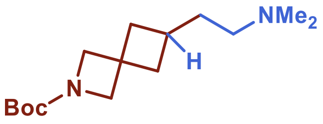

3t, 48%

Following the General Procedure 2, the corresponding olefin (1.00 mmol, 209 mg) afforded 129 mg (48% isolated yield) of the titled compound **3t** as a colorless oil after purification by silica gel column chromatography (eluent: ethyl acetate /petroleum ether/triethylamine = 10/9/1, stained with Dragendorff's reagent).

Physical Appearance: colorless oil

^{1}H NMR (300 MHz, $CDCl_3$): δ 3.84 – 3.72 (m, 4H), 2.56 – 2.44 (m, 4H), 2.22 – 2.04 (m, 8H), 1.78 – 1.66 (m, 3H), 1.48 – 1.41 (m, 1H), 1.35 (s, 9H).

^{13}C NMR (75 MHz, $CDCl_3$): δ 156.2, 156.2, 79.2, 79.1, 62.6, 62.1, 60.8, 57.4, 51.4, 45.5, 39.0, 38.8, 34.7, 34.4, 30.7, 28.4, 27.8, 27.5.

HRMS (ESI-TOF) m/z: $[M + H]^+$ Calcd for $C_{15}H_{29}N_2O_2$ 269.2224; Found 269.2248.

Compound 3u

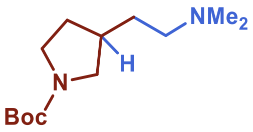

3u, 29%

Following the General Procedure 2, the corresponding olefin (1.00 mmol, 183 mg) afforded 70 mg (29% isolated yield) of the titled compound **3u** as a colorless oil after purification by silica gel column chromatography (eluent: DCM/MeOH/25% NH_3 (aq.) = 17/1/2, stained with Dragendorff's reagent).

Physical Appearance: colorless oil

^{1}H NMR (300 MHz, $CDCl_3$): δ 3.53 – 2.74 (m, 5H), 2.23 – 2.00 (m, 9H), 1.97 – 1.87 (m, 1H), 1.51 – 1.44 (m, 2H), 1.38 (s, 9H).

^{13}C NMR (75 MHz, $CDCl_3$): δ 154.6, 79.0, 58.3, 51.6, 51.2, 45.7, 45.5, 45.4, 37.2, 36.4, 31.9, 31.3, 31.2, 28.6.

HRMS (ESI-TOF) m/z: $[M + H]^+$ Calcd for $C_{13}H_{27}N_2O_2$ 243.2067; Found 243.2072.

Compound 3v

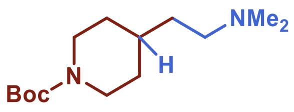

3v, 61%

Following the General Procedure 2, the corresponding olefin (1.00 mmol, 197 mg) afforded 155 mg (61% isolated yield) of the titled compound **3v** as a colorless oil after purification by silica gel column chromatography (eluent: DCM/MeOH/25% NH_3 (aq.) = 17/1/2, stained with Dragendorff's reagent).

Physical Appearance: colorless oil

^{1}H NMR (300 MHz, $CDCl_3$): δ 3.98 – 3.95 (m, 2H), 2.59 (t, *J* = 11.3 Hz, 2H), 2.28 – 2.17 (m, 8H), 1.56 (d, *J* = 12.6 Hz, 2H), 1.36 – 1.33 (m, 12H), 1.08 – 0.95 (m, 2H).

^{13}C NMR (75 MHz, $CDCl_3$): δ 154.8, 79.1, 57.0, 45.3, 43.9, 34.1, 34.0, 32.2, 28.4.

HRMS (ESI-TOF) m/z: $[M + H]^+$ Calcd for $C_{14}H_{29}N_2O_2$ 257.2224; Found 257.2228.

Compound 3w

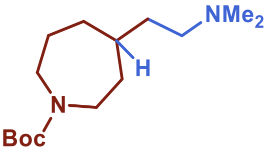

3w, 48%

Following the General Procedure 2, the corresponding olefin (1.00 mmol, 211 mg) afforded 129 mg (48% isolated yield) of the titled compound **3w** as a colorless oil after purification by silica gel column chromatography (eluent: (eluent: DCM/MeOH/25% NH_3 (aq.) = 17/1/2, stained with Dragendorff's reagent).

Physical Appearance: colorless oil

^{1}H NMR (300 MHz, $CDCl_3$): δ 3.93 – 2.99 (m, 5H), 2.21 – 2.13 (m, 8H), 1.73 – 1.36 (m, 17H).

^{13}C NMR (75 MHz, $CDCl_3$): δ 155.6, 78.9, 57.7, 46.7, 46.1, 45.5, 45.4, 45.2, 44.8, 37.4, 36.9, 35.1, 35.0, 35.0, 34.8, 33.7, 33.1, 28.5, 27.0, 26.8.

HRMS (ESI-TOF) m/z: $[M + H]^+$ Calcd for $C_{15}H_{31}N2O_2$ 271.2380; Found 271.2402.

Compound 3x

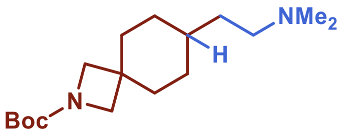

3x, 59%

Following the General Procedure 2, the corresponding olefin (1.00 mmol, 237 mg) afforded 174 mg (59% isolated yield) of the titled compound **3x** as a yellow oil after purification by silica gel column chromatography (eluent: DCM/MeOH/25% NH_3 (aq.) = 17/1/2, stained with Dragendorff's reagent).

Physical Appearance: yellow oil

^{1}H NMR (300 MHz, $CDCl_3$): δ 4.03 – 3.99 (m, 1H), 3.41 (d, J = 14.2 Hz, 2H), 3.27 (s, 2H), 2.28 – 2.16 (m, 8H), 1.72 (d, J = 11.4 Hz, 2H), 1.52 (d, J = 13.6 Hz, 2H), 1.29 – 1.23 (m, 11H), 1.17 – 1.08 (m, 1H).

^{13}C NMR (75 MHz, $CDCl_3$): δ 156.5, 79.0, 59.2, 57.1, 49.9, 44.8, 35.3, 34.8, 34.4, 33.6, 29.3, 28.3, 25.2.

HRMS (ESI-TOF) m/z: $[M + H]^+$ Calcd for $C_{17}H_{33}N_2O_2$ 297.2537; Found 297.2555.

3.6 Electrochemical ketone synthesis using DMA as solvent.

Compound 4a

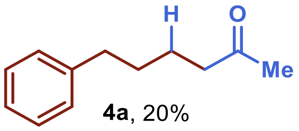

4a, 20%

Following the General Procedure 1 but using DMA (5 mL)and 2-MeTHF (4.5 mL) as solvent and quenched by HCl (3.2 M), the corresponding olefin (1.00 mmol, 132 mg) afforded 35 mg (20% isolated yield) of the titled compound **4a** as a colorless oil after purification by silica gel column chromatography (eluent: petroleum ether/ethyl acetate = 10/1, stained with DNP).

Physical Appearance: colorless oil

^{1}H NMR (300 MHz, $CDCl_3$): δ 7.35 – 7.16 (m, 5H), 2.75 – 2.56 (m, 2H), 2.56 – 2.37 (m, 2H), 2.15 (s, 3H), 1.69 – 1.55 (m, 4H).

^{13}C NMR (75 MHz, $CDCl_3$): δ 209.0, 142.2, 128.4, 128.3, 125.8, 43.6, 35.7, 31.0, 29.9, 23.5.

Characterization data for this ketone was in agreement with the literature.[15]

Compound 4d

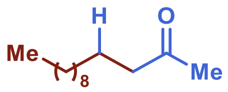

4d, 17%

Following the General Procedure 1 but using DMA (5 mL) and 2-MeTHF (4.5 mL) as solvent and quenched by HCl (3.2 M), the corresponding olefin (1.00 mmol, 154 mg) afforded 35 mg (17% isolated yield) of the titled compound **4d** as a colorless oil after purification by silica gel column chromatography (eluent: petroleum ether/ethyl acetate = 9/1, stained with DNP).

Physical Appearance: colorless oil

^{1}H NMR (300 MHz, CDCl$_3$): δ 2.40 (t, *J* = 7.5 Hz, 2H), 2.12 (s, 3H), 1.61 – 1.50 (m, 2H), 1.32 – 1.19 (m, 16H), 0.94 – 0.81 (m, 3H).

^{13}C NMR (75 MHz, CDCl$_3$): δ 209.5, 44.0, 32.0, 30.0, 29.7, 29.7, 29.6, 29.5, 29.5, 29.3, 24.0, 22.8, 14.2.

Characterization data for this aldehyde was in agreement with the literature.[16]

3.7 Aldehyde Homologation

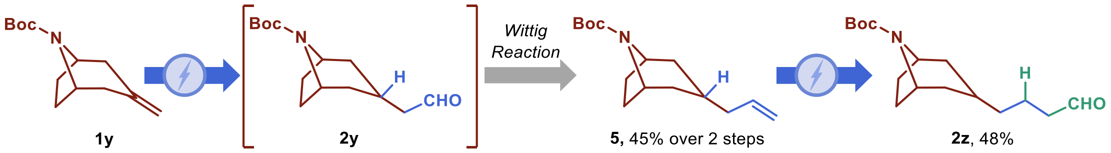

To an oven dried 20 mL ElectraSyn 2.0 vial was charged TEABr (1.00 equiv., 1.00 mmol, 322 mg), olefin **1y** (5.00 mmol, 1.11 g), anhydrous DMF (10 mL), 2-MeTHF (8 mL) and a stirring bar, The vial was closed with an ElectraSyn 2.0 vial cap with a magnesium anode and a titanium cathode, then the suspension was purged with argon for 5 minutes, then the vial was placed on an IKA ElectraSyn 2.0 stirring plate and electrolysis was set to 40 mA, 5.00 mmol, 48 hours. The reaction underwent the programmed electrolysis under argon. After the reaction, the mixture was transferred to a separatory funnel, the electrodes were rinsed with Et_2O and water, then citric acid (10 wt%) was added and the mixture was shaken vigorously. The aqueous layer was extracted 3 times with Et_2O. The combined organic layers were washed with brine, then dried over Na_2SO_4 before being concentrated via rotavap. The crude oil **2y** was used without further purification.

To a vigorously stirred suspension of $MePh_3Br$ (2.00 equiv., 10.00 mmol, 3.57 g) in anhydrous THF (50 mL), ^{t}BuOK (2.00 equiv., 10.00 mmol, 1.12 g) was added in one portion, the mixture was then allowed to stir for 15 minutes then the aforementioned crude aldehyde mixture was added in one portion and stirred overnight, The mixture was then quenched with saturated NH_4Cl and extracted 3 times with Et_2O, the organic layers were combined and washed again with brine, dried over Na_2SO_4 and concentrated via rotavap then purified via silica gel column chromatography (eluent: petroleum ether/ethyl acetate = 9/1, stained with $KMnO_4$) to afford 300.0 mg of olefin **5** (45% isolated yield over 2 steps) as colorless oil.

Following the General Procedure and quenched by citric acid (10 wt%), the corresponding olefin (1.00 mmol, 251 mg) afforded 135.0 mg (48% isolated yield) of aldehyde **2z** as a colorless oil after purification by silica gel column chromatography (eluent: petroleum ether/ethyl acetate = 5/1, stained with DNP).

Compound 5

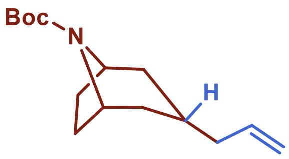

5, 45% over 2 steps

Physical Appearance: colorless oil

^{1}H NMR (300 MHz, $CDCl_3$): δ 5.74 – 5.54 (m, 1H), 5.00 – 4.83 (m, 2H), 4.26 – 3.93 (m, 2H), 1.90 – 1.67 (m, 5H), 1.63 – 1.42 (m, 4H), 1.40 – 1.18 (m, 11H).

^{13}C NMR (75 MHz, $CDCl_3$): δ 153.3, 136.5, 115.9, 78.8, 53.8, 53.1, 41.1, 37.7, 37.0, 28.4, 28.4, 28.2, 27.8.

HRMS (ESI-TOF) m/z: $[M + H]^+$ Calcd for $C_{15}H_{26}NO_2$ 252.1958; Found 252.1964.

Compound 2z

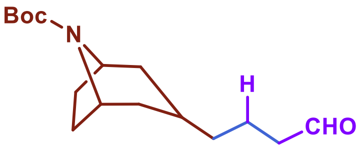

2z, 48%

Physical Appearance: colorless oil

^{1}H NMR (300 MHz, $CDCl_3$): δ 9.69 (t, J = 1.7 Hz, 1H), 4.25 – 3.97 (m, 2H), 2.35 (td, J = 7.3, 1.7 Hz, 2H), 1.95 – 1.81 (m, 2H), 1.80 – 1.66 (m, 1H), 1.62 – 1.44 (m, 6H), 1.40 (s, 9H), 1.34 – 1.09 (m, 4H).

^{13}C NMR (75 MHz, $CDCl_3$): δ 202.5, 153.5, 79.0, 53.9, 53.1, 44.0, 38.0, 37.3, 36.5, 28.5, 28.3, 27.9, 19.3.

HRMS (ESI-TOF) m/z: $[M + Na]^+$ Calcd for $C_{16}H_{27}NNaO_3$ 304.1883; Found 304.1897.

4. Mechanistic studies

4.1. Competitive KIE experiment

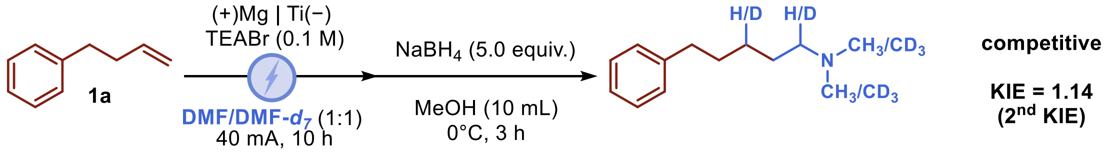

To an oven dried 5 mL ElectraSyn 2.0 vial was charged TEABr (1.00 mmol, 210 mg), DMF (1.5 mL), DMF-d_7 (1.5 mL), and a stirring bar, The vial was closed with an ElectraSyn 2.0 vial cap with a magnesium anode and a titanium cathode, then purged with argon for 5 minutes, 4-phenyl-butene **1a** (1.00 mmol, 132 mg) was then added to the solution via syringe Then the vial was placed on an IKA ElectraSyn 2.0 stirring plate and electrolysis was set to 40 mA, 10 hours. The reaction underwent the programmed electrolysis under argon, the mixture was then transferred to a 25 mL round bottom flask, MeOH (10 mL) was added and the mixture was cooled down to 0 °C, then $NaBH_4$ (5.00 equiv., 5.00 mmol, 189 mg) was added in one portion, upon addition the mixture was allowed to raise to room temperature and stirred for 3 hours, then quenched with saturated NH_4Cl and extracted with DCM for 3 times, the combined organic layers were washed with brine, then dried over Na_2SO_4 before being concentrated via rotavap. The crude mixture was purified via silica gel column chromatography (eluent: petroleum ether/ethyl acetate = 1/1, 5% TEA as additive, stained with Dragendorff's reagent). Competitive KIE value was depended on hydrogen loss in ^{1}H NMR.

Physical Appearance: colorless oil

^{1}H NMR (300 MHz, $CDCl_3$): δ 7.36 – 7.14 (m, 5H), 2.70 – 2.59 (m, 2H), 2.24 (m, 4.73H), 1.67 (p, J = 7.5 Hz, 2H), 1.51 (q, J = 7.8 Hz, 2H), 1.45 – 1.33 (m, 2H).

Depending on 4-phenyl-butene was attacked by DMF or DMF-d_7 and quenched by DMF or DMF-d_7, the mixture is mainly made of 4 different species.

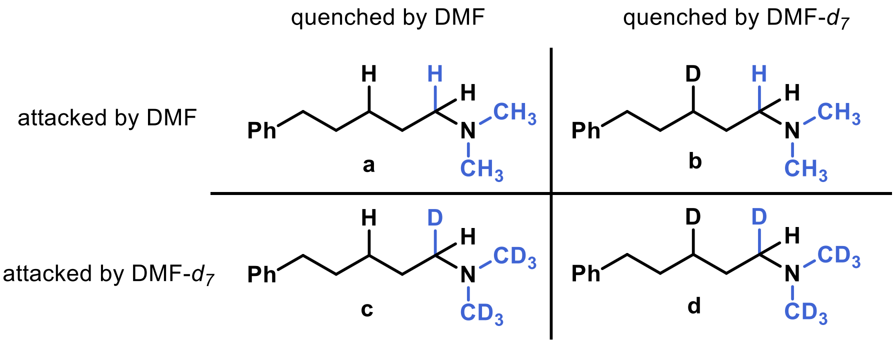

Protons at α-position to these amine share similar chemical shift and overlap with each other (δ 2.24 (m, 4.73H)), the competitive KIE of distonic radical addition step can be calculated via the following equations:

$$8a + 8b + 1c + 1d = 4.73$$

$$5a + 5b + 5c + 5d = 5$$

$$Competitive\ KIE = \frac{a+b}{c+d} = \frac{0.53}{0.46} = 1.14\ (secondary\ KIE)$$

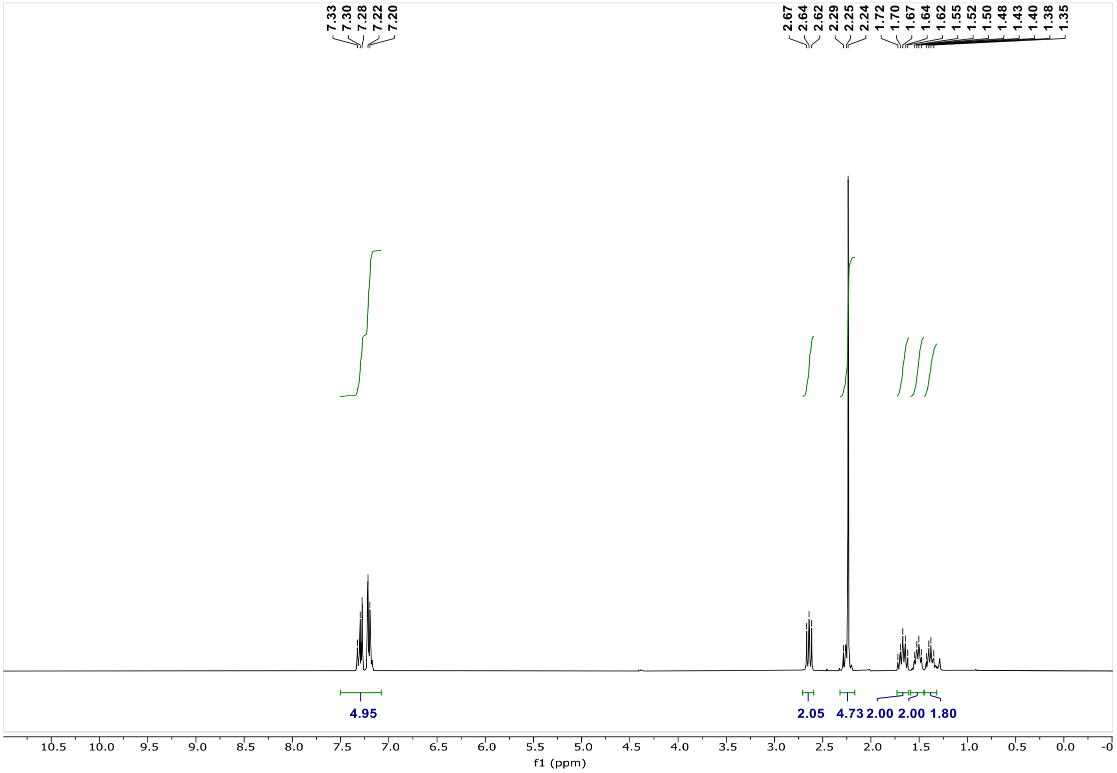

4.2. 2,4-Dinitrophenylhydrazine capturing experiment

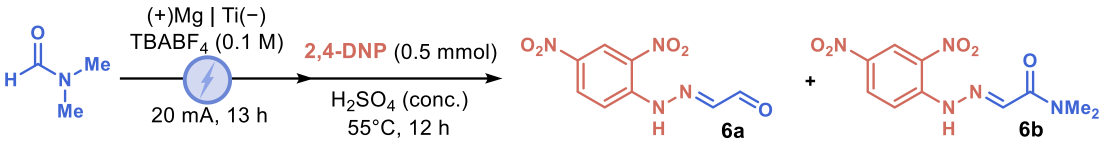

To an oven dried 5 mL ElectraSyn 2.0 vial was charged $TBABF_4$ (0.50 mmol, 164 mg), anhydrous DMF (4.5 mL), and a stirring bar, The vial was closed with an ElectraSyn 2.0 vial cap with a magnesium anode and a titanium cathode, then purged with argon for 5 minutes. Then the vial was placed on an IKA ElectraSyn 2.0 stirring plate and electrolysis was set to 20 mA, 13 hours. The reaction underwent the programmed electrolysis under argon. After the reaction, the mixture was transferred to a 25 mL round bottom flask and H_2SO_4 (95 wt%) was added dropwise until the pH dropped below 1 (determined via pH paper), then 2,4-Dinitrophenylhydrazine (0.50 mmol, 99 mg) was added and the mixture was heated up to 55 °C and stirred overnight, the mixture was quenched with saturated NH_4Cl and extracted with DCM for 3 times, the combined organic layers were washed with brine, then dried over Na_2SO_4 before being concentrated via rotavap. The crude mixture was purified via silica gel column chromatography (eluent: petroleum ether/DCM = 50/50 to pure DCM) to afford the desired product **6a** and **6b**.

As the *N*-methyl C–H derivative was not observed under these conditions, this might indicate that the formyl radical is major product of the HAT quenching step. Nevertheless, owing to the BDE similarities between these two C–H bonds, we cannot exclude that both the HAT events are occurring.

Compound 6a

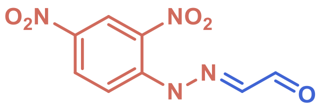
6a

Physical Appearance: orange solid

^{1}H NMR (300 MHz, DMSO-*d*$_6$): δ 12.12 (s, 1H), 9.61 (d, *J* = 7.8 Hz, 1H), 8.86 (d, *J* = 2.7 Hz, 1H), 8.51 (dd, *J* = 9.5, 2.7 Hz, 1H), 8.12 (d, *J* = 7.8 Hz, 1H), 8.08 (d, *J* = 9.5 Hz, 1H).

^{13}C NMR (75 MHz, DMSO-*d*$_6$): δ ^{13}C NMR (126 MHz, DMSO) δ 191.5, 144.8, 143.1, 139.7, 132.5, 129.9, 122.5, 117.7.

HRMS (ESI-TOF) m/z: $[M - H]^-$ Calcd for $C_8H_5N_4O_5$ 237.0265; Found 237.0265.

Compound 6b

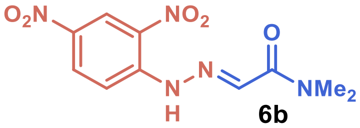

Physical Appearance: orange solid

^{1}H NMR (300 MHz, DMSO-*d*$_6$): δ 15.07 (s, 1H), 8.90 – 8.84 (m, 1H), 8.48 – 8.42 (m, 1H), 8.07 – 8.00 (m, 1H), 7.77 – 7.73 (m, 1H), 3.25 (s, 3H), 2.97 (s, 3H).

^{13}C NMR (75 MHz, DMSO-*d*$_6$): δ 161.7, 144.1, 138.7, 130.9, 130.4, 130.0, 122.7, 116.2, 37.1, 35.1.

HRMS (ESI-TOF) m/z: $[M - H]^-$ Calcd for $C_{10}H_{10}N_5O_5$ 280.0687; Found 280.0689.

4.3. Radical clock experiment

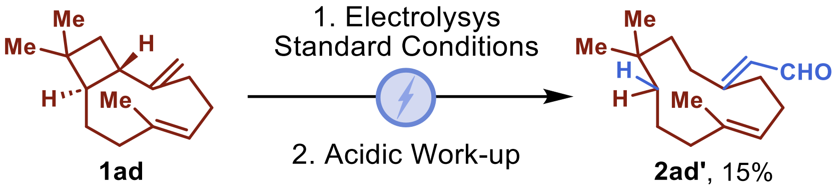

To an oven dried 5 mL ElectraSyn 2.0 vial was charged TBABr (1.00 mmol, 322 mg), anhydrous DMF (2.5 mL), 2-MeTHF (2.0 mL), and a stirring bar, The vial was closed with an ElectraSyn 2.0 vial cap with a magnesium anode and a titanium cathode, then purged with argon for 5 minutes, β-carophyllene (1.00 mmol, 204 mg) was then added to the solution via syringe Then the vial was placed on an IKA ElectraSyn 2.0 stirring plate and electrolysis was set to 40 mA, 10 hours. The reaction underwent the programmed electrolysis under argon, the mixture was quenched with HCl (3.2 M) and extracted with Et_2O for 3 times, the combined organic layers were washed with brine, then dried over Na_2SO_4 before being concentrated via rotavap. The crude mixture was purified via silica gel column chromatography (eluent: petroleum ether/ethyl acetate = 95/5, product is UV active and can be stained with $KMnO_4$) to afford the ring opening product **2ad’** (35 mg, 15% isolated yield).

Compound 2ad’

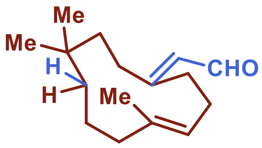

2ad', 15%

Physical Appearance: colorless oil

^{1}H NMR (300 MHz, $CDCl_3$): δ 10.04 (d, J = 8.2 Hz, 1H), 5.83 (d, J = 8.2 Hz, 1H), 5.29 (t, J = 7.2 Hz, 1H), 2.82 – 2.56 (m, 2H), 2.38 (q, J = 6.8 Hz, 2H), 2.21 – 2.06 (m, 2H), 1.95 – 1.85 (m, 2H), 1.56 (s, 3H), 1.42 – 1.20 (m, 6H), 0.80 (s, 6H).

^{13}C NMR (75 MHz, $CDCl_3$): δ 191.1, 173.4, 136.4, 127.1, 123.6, 40.6, 36.7, 35.1, 34.8, 33.4, 33.3, 30.2, 28.3, 20.6, 17.0.

HRMS (ESI-TOF) m/z: $[M + H]^+$ Calcd for $C_{16}H_{27}O$ 235.2056; Found 235.2053.

4.4. Deuteration experiment

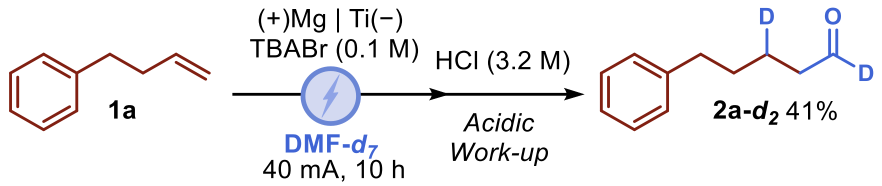

To an oven dried 5 mL ElectraSyn 2.0 vial was charged TBABr (1.00 mmol, 322 mg), DMF-d_7 (2.5 mL), and a stirring bar, The vial was closed with an ElectraSyn 2.0 vial cap with a magnesium anode and a titanium cathode, then purged with argon for 5 minutes, 4-phenyl-butene **1a** (1.00 mmol, 132 mg) was then added to the solution via syringe Then the vial was placed on an IKA ElectraSyn 2.0 stirring plate and electrolysis was set to 40 mA, 10 hours. The reaction underwent the programmed electrolysis under argon, the mixture was quenched with HCl (3.2 M) and extracted with Et_2O for 3 times, the combined organic layers were washed with brine, then dried over Na_2SO_4 before being concentrated via rotavap. The crude mixture was purified via silica gel column chromatography (eluent: petroleum ether/ethyl acetate = 9/1, stained with DNP) to afford deuterated product **2a-d_2** (67 mg, 41% isolated yield).

Compound 2a-d_2

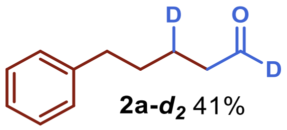

Physical Appearance: colorless oil

^{1}H NMR (300 MHz, $CDCl_3$): δ 7.25 – 7.05 (m, 5H), 2.61 – 2.30 (m, 4H), 1.62 – 1.50 (m, 3H).

^{13}C NMR (75 MHz, $CDCl_3$): δ 202.7, 202.4, 202.0, 142.1, 128.6, 128.5, 128.5, 128.5, 125.9, 43.6, 43.6, 43.5, 35.7, 30.9, 21.6, 21.4, 21.1.

4.5. HAT probe experiment

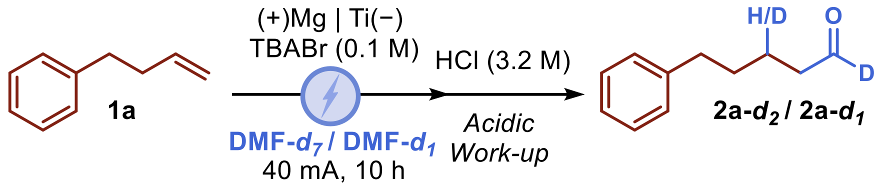

To an oven dried 5 mL ElectraSyn 2.0 vial was charged TBABr (1.00 mmol, 322 mg), DMF-d_7 (1.8 mL) and DMF-d_1 (0.8 mL), and a stirring bar, The vial was closed with an ElectraSyn 2.0 vial cap with a magnesium anode and a titanium cathode, then purged with argon for 5 minutes, 4-phenyl-butene **1a** (1.00 mmol, 132 mg) was then added to the solution via syringe Then the vial was placed on an IKA ElectraSyn 2.0 stirring plate and electrolysis was set to 40 mA, 10 hours. The reaction underwent the programmed electrolysis under argon, the mixture was quenched with HCl (3.2 M) and extracted with Et_2O for 3 times, the combined organic layers were washed with brine, then dried over Na_2SO_4 before being concentrated via rotavap. The crude mixture was purified via silica gel column chromatography (eluent: petroleum ether/ethyl acetate = 9/1, stained with DNP) to afford deuterated product **2a-d_2 / 2a-d_1**.

Compound 2a-d_2/ 2a-d_1

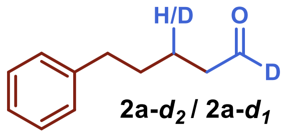

Physical Appearance: colorless oil

^{1}H NMR (300 MHz, $CDCl_3$): δ 7.38 – 7.16 (m, 5H), 2.58 – 2.76 (m, 2H), 2.39 – 2.55 (m, 1.86H), 1.58 – 1.78 (m, 3.41H).

^{13}C NMR (75 MHz, $CDCl_3$): δ 202.6, 202.3, 201.9, 142.0, 128.4, 128.4, 125.9, 43.6, 43.6, 43.5, 43.5, 43.5, 35.7, 35.6, 30.9, 30.8, 21.7, 21.6, 21.3, 21.0.

Compound **2a-*d₂*/ 2a-*d₁***: ^{1}H NMR (300 MHz, $CDCl_3$)

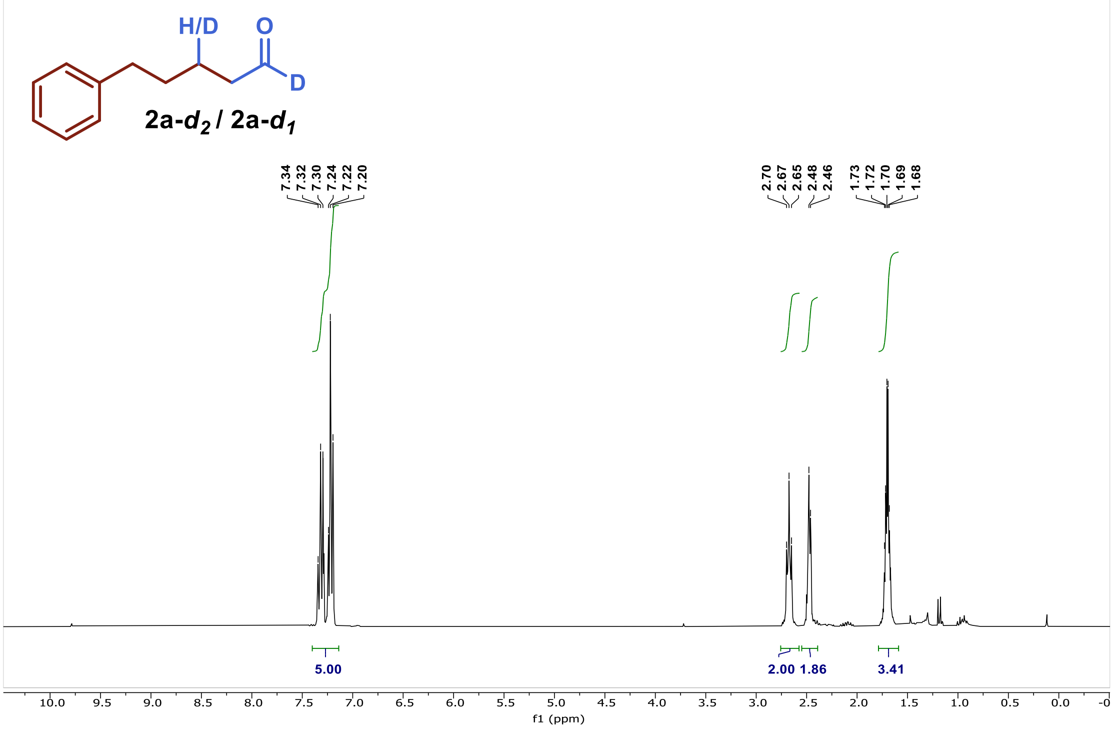

Compound **2a-*d₂*/ 2a-*d₁***: [1]C NMR (75 MHz, $CDCl_3$)

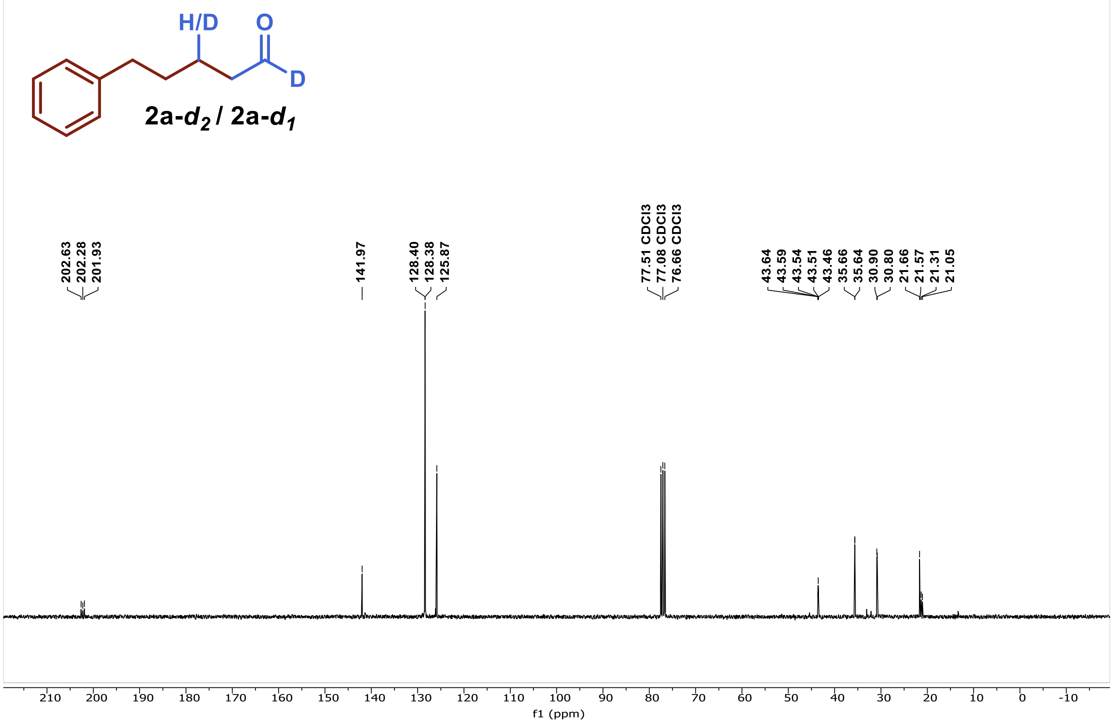

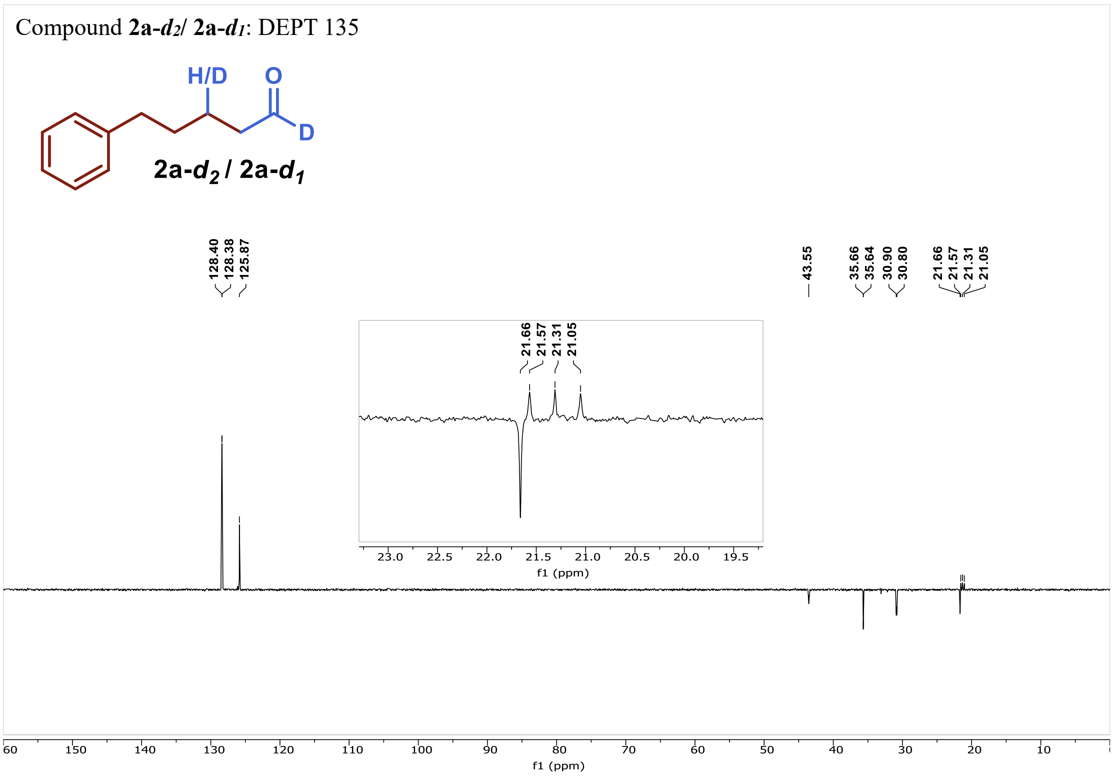
Compound 2a-d2/ 2a-d1: DEPT 135
H/D
O
D
2a-d2 / 2a-d1
128.40
128.38
125.87
43.55
35.66
35.64
30.90
30.80
21.66
21.57
21.31
21.05
f1 (ppm)

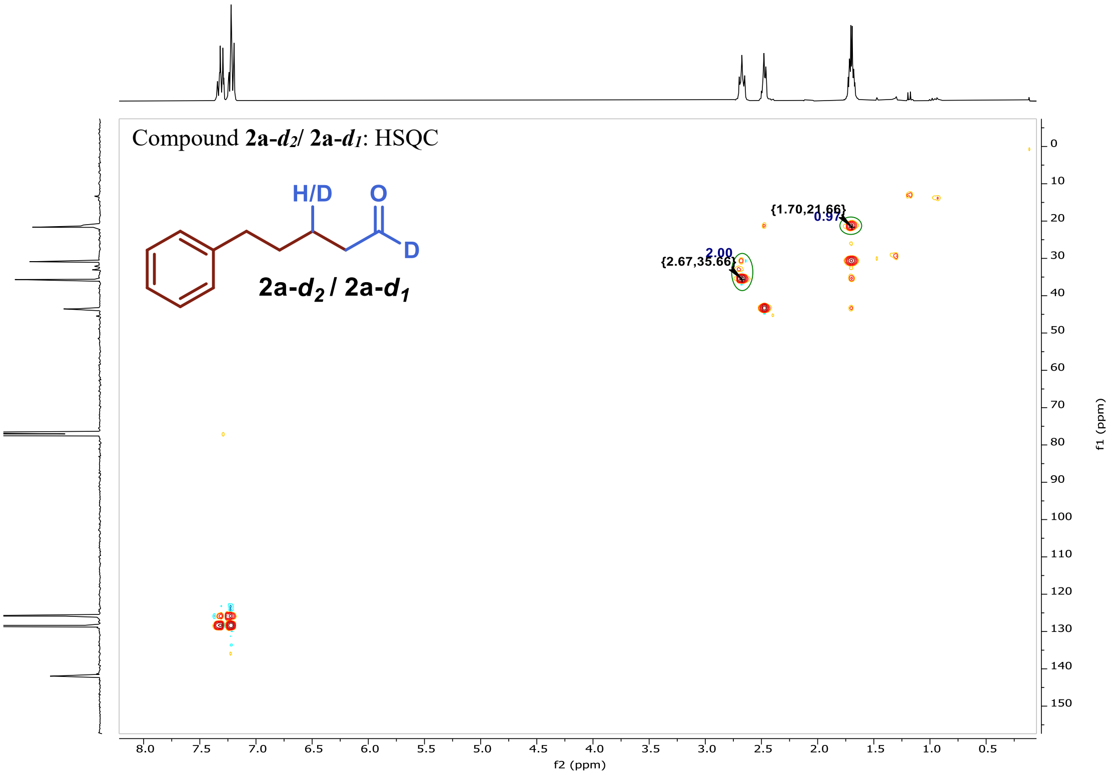
Compound 2a-d2/ 2a-d1: HSQC
H/D
O
D
2a-d2 / 2a-d1
{1.70,21.66}
0.97
2.00
{2.67,35.66}
f2 (ppm)
f1 (ppm)

4.6. Control experiments

Thermochemical control experiment with Na^0

Metallic sodium (2.18 equiv., 21.75 mmol, 500 mg) was cut to reveal its shiny surface and divided into small pieces then washed several times with hexane before weighed and added to a round bottle flask. Then depending on the control reaction, $Mg(OTf)_2$ (0.47 equiv., 4.65 mmol, 1.50g) was added, then a mixture of 4-Ph-butene **1a** (9.98 mmol, 1.32g), DMF (10 mL) and 2-MeTHF (8 mL) was added under argon. The flask was heated up to 120°C in oil bath and vigorously reflux for 6 hours. The mixture was quenched with HCl in MeOH (1.2 M) and diluted with ethyl acetate before filtered by a syringe filter. Then the sample was analyzed by GC-MS. No product can be found after analysis.

Thermochemical control experiment with Mg^0

Magnesium powder (4.12 equiv., 41.14 mmol, 1.00 g) was added to a round bottle flask. Then a mixture of 4-Ph-butene **1a** (9.98 mmol, 1.32g), DMF (10 mL) and 2-MeTHF (8 mL) was added under argon. The flask was heated up to 120°C in oil bath and vigorously reflux for 6 hours. The liquid part of the mixture was quenched with HCl in MeOH (1.2 M) and diluted with ethyl acetate before filtered by a syringe filter. Then the sample was analyzed by GC-MS. No product can be found after analysis.

Divided cell experiment

To an oven dried ElectraSyn 2.0 divided cell, both chambers were charged with TBABr (1.00 mmol, 322 mg), DMF (6 mL), and a stirring bar, The apparatus was closed with an ElectraSyn 2.0 divided cell cap with a magnesium anode and a titanium cathode, then purged with argon for 5 minutes. Then the vial was placed on an IKA ElectraSyn 2.0 stirring plate and electrolysis was set to 40 mA, 10 hours. The reaction underwent the programmed electrolysis under argon, After the reaction, the mixtures was transferred to a 25 mL round bottom flask and H_2SO_4 (95 wt%) was added dropwise until the pH dropped below 1 (determined via pH paper), then 2,4-Dinitrophenylhydrazine was added and the mixture was heated up to 55 °C and stirred overnight, both mixtures from anodic and cathodic chamber after DNP capturing were analyzed via LC-MS and **6b** was spotted only in anodic chamber.

4.7. CV analysis

Cyclic voltammograms were recorded with an EmStat4X potentiostat at 100 mV s^{-1} scan rate, using graphite working electrode and platinum counter electrode with 0.1 M TBABr as the supporting electrolyte in DMF with 10 mM substrate.

Takeaway: For substrates with reductive peaks between -3.5 V to 0 V, other reductive reactions (e.g. Birch reduction, dehalogenation) can be observed. However, no reductive peaks in this potential window doesn't necessarily mean the success of the substrate. Other factors, including steric hinderance and stability during workup, also play an important role in this reaction.

Background

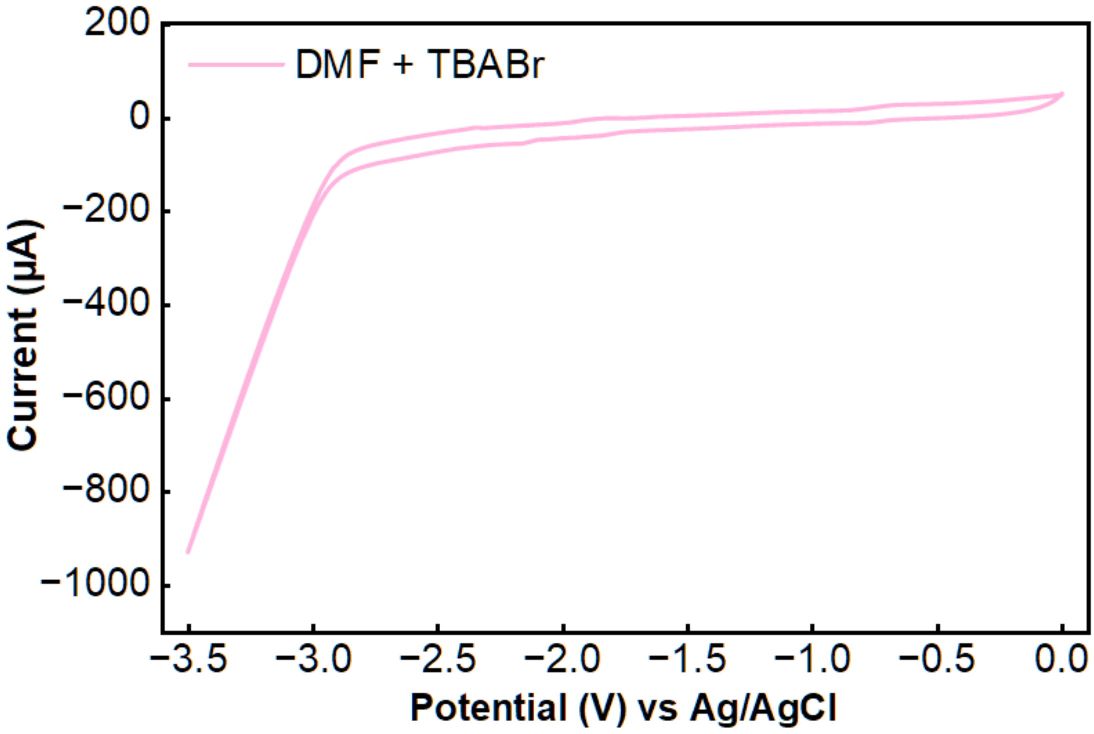

Compound 1b

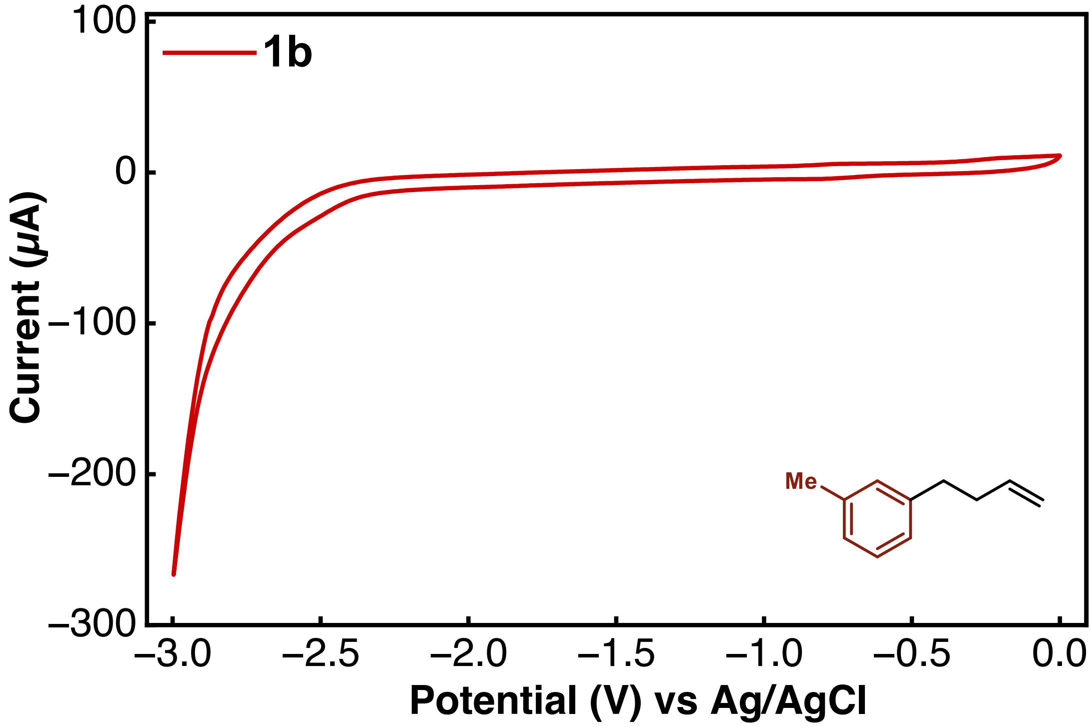

Compound S1

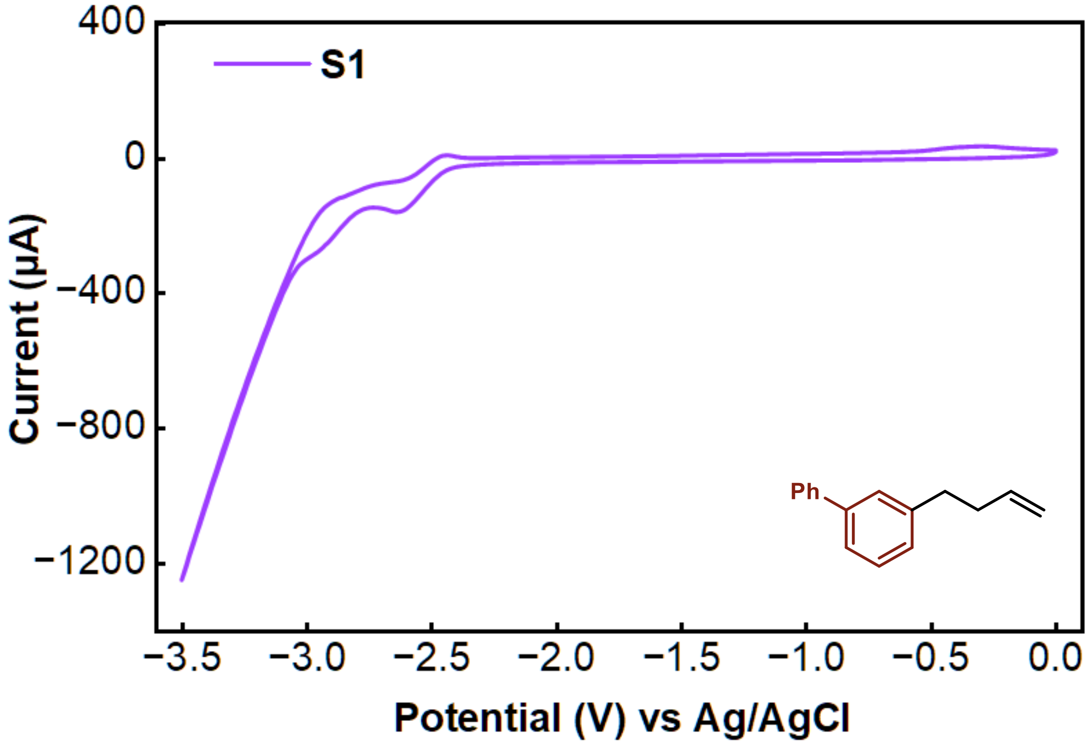

Compound S2

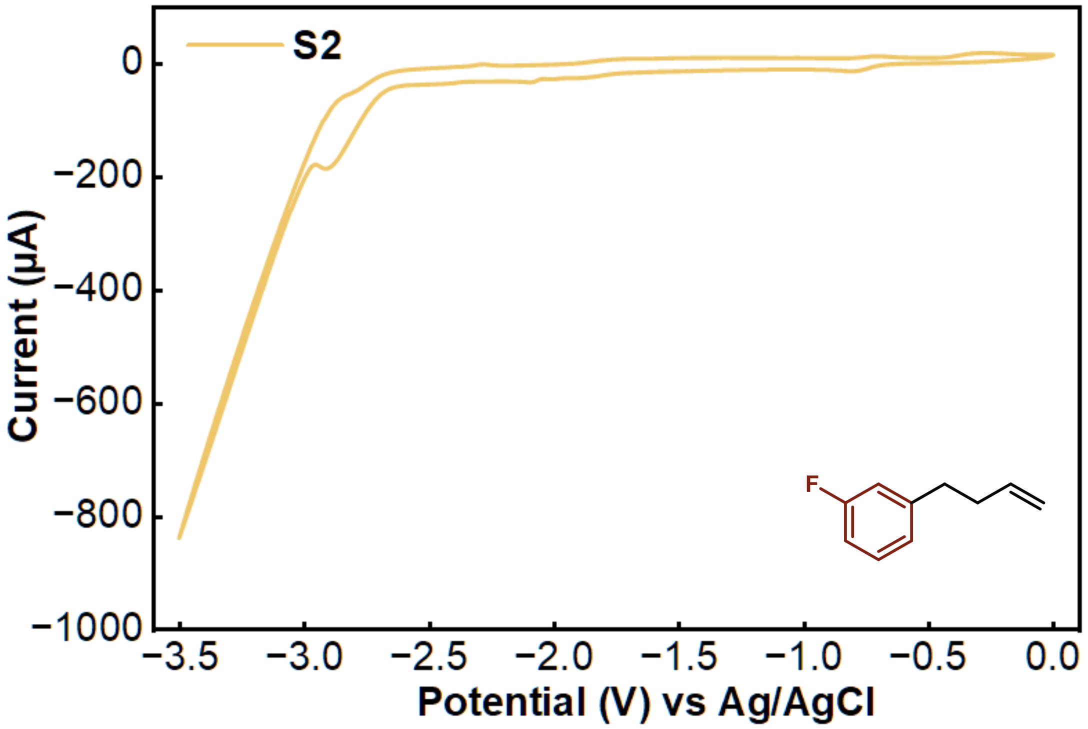

Compound S3

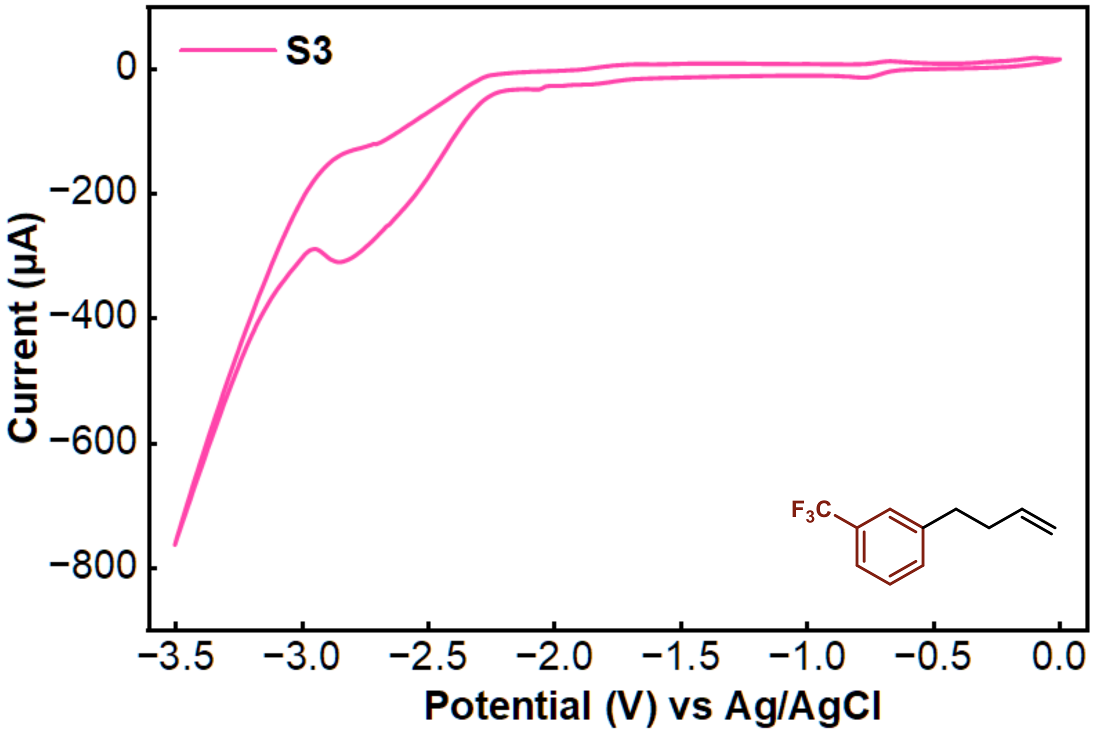

Compound S4

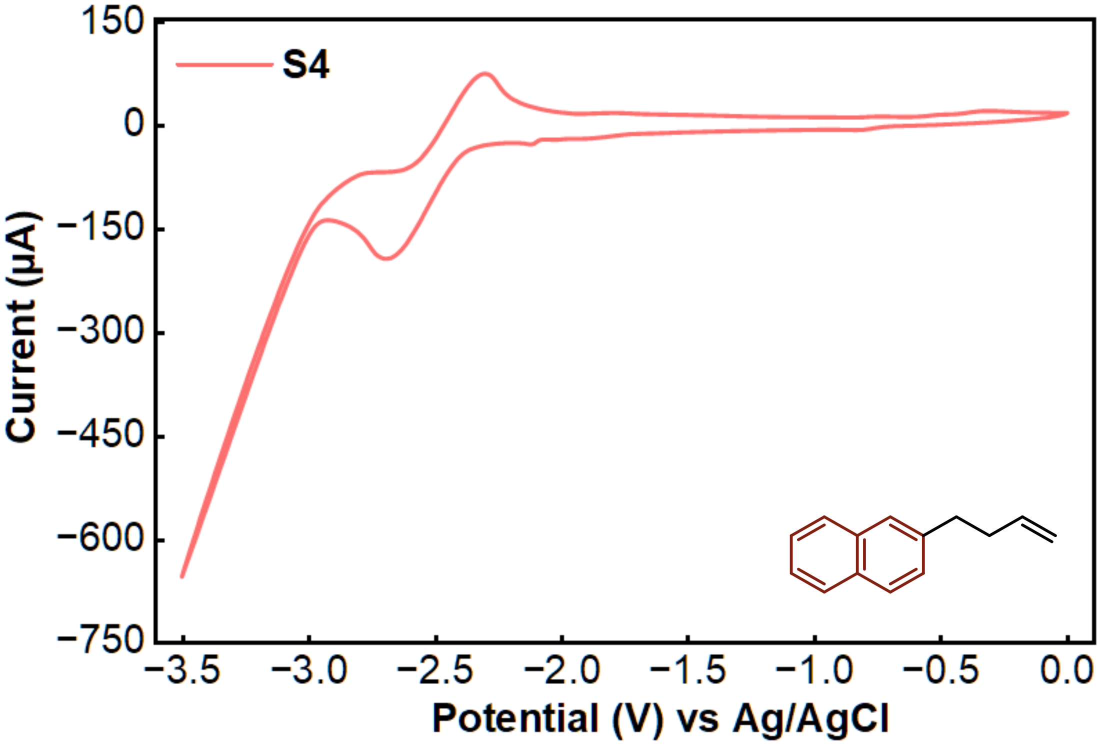

Compound S8

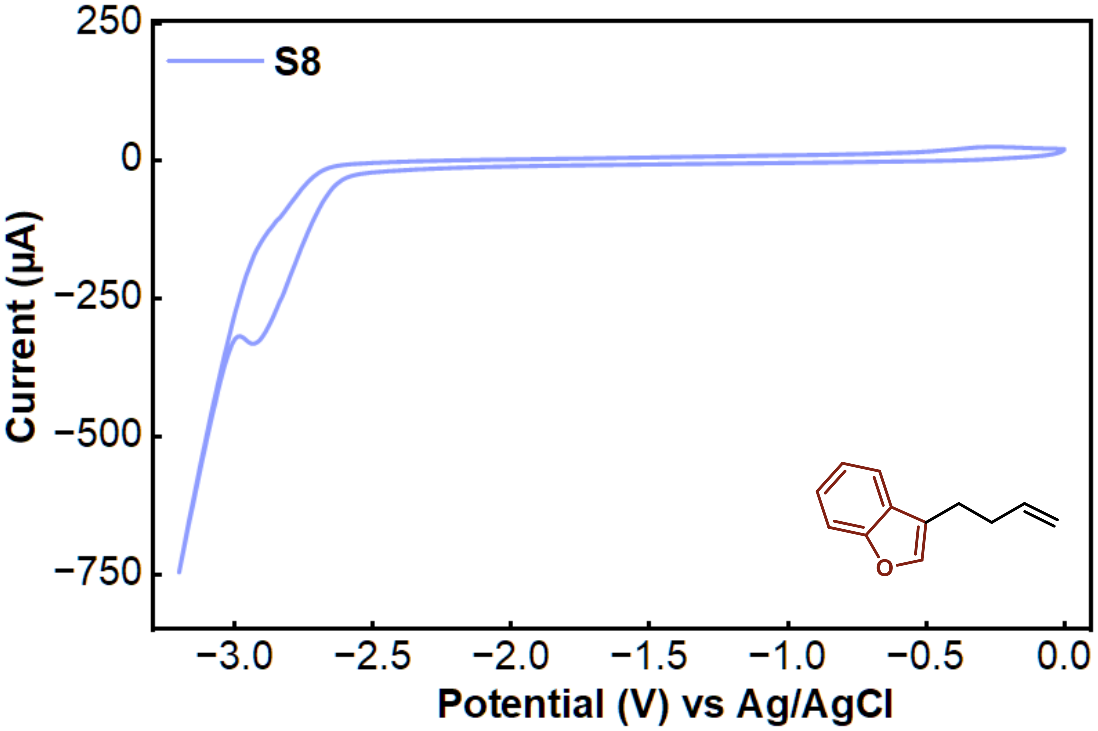

Compound S9

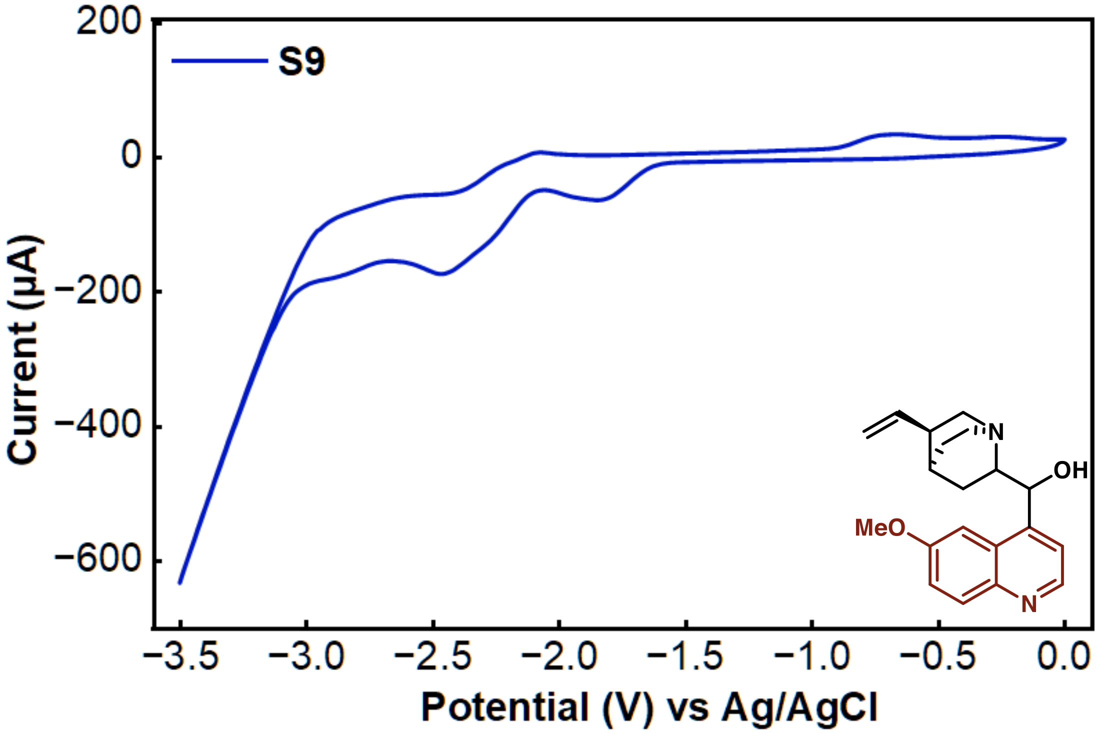

Compound S10

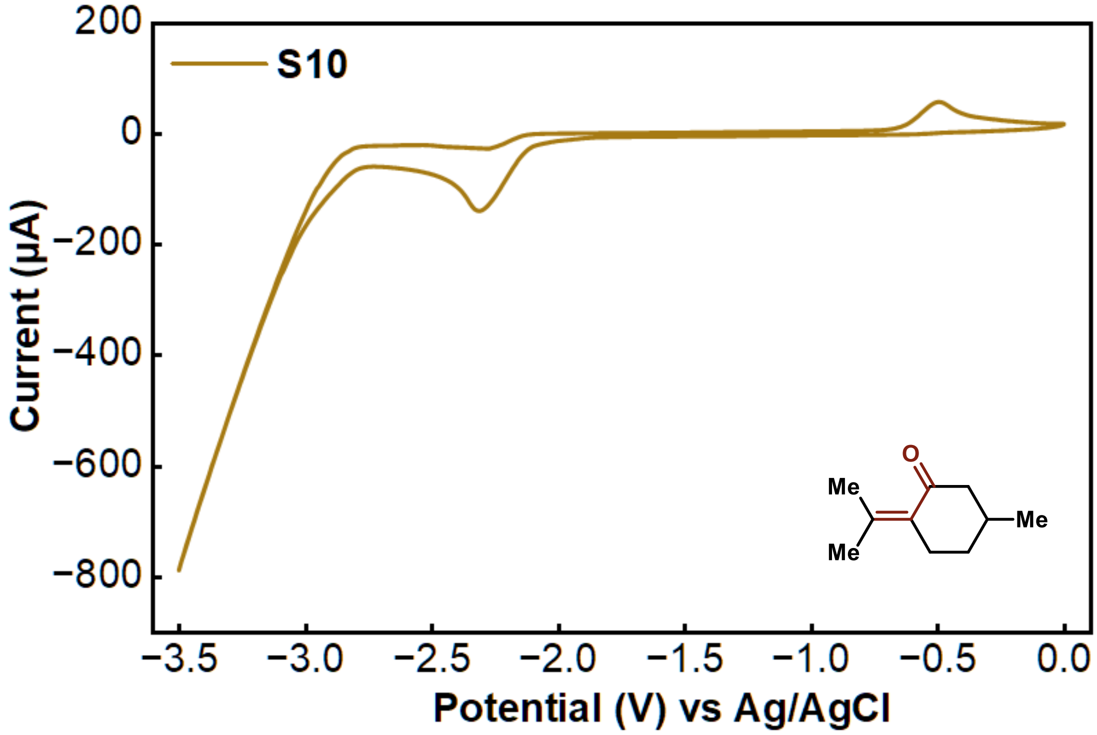

Compound S11

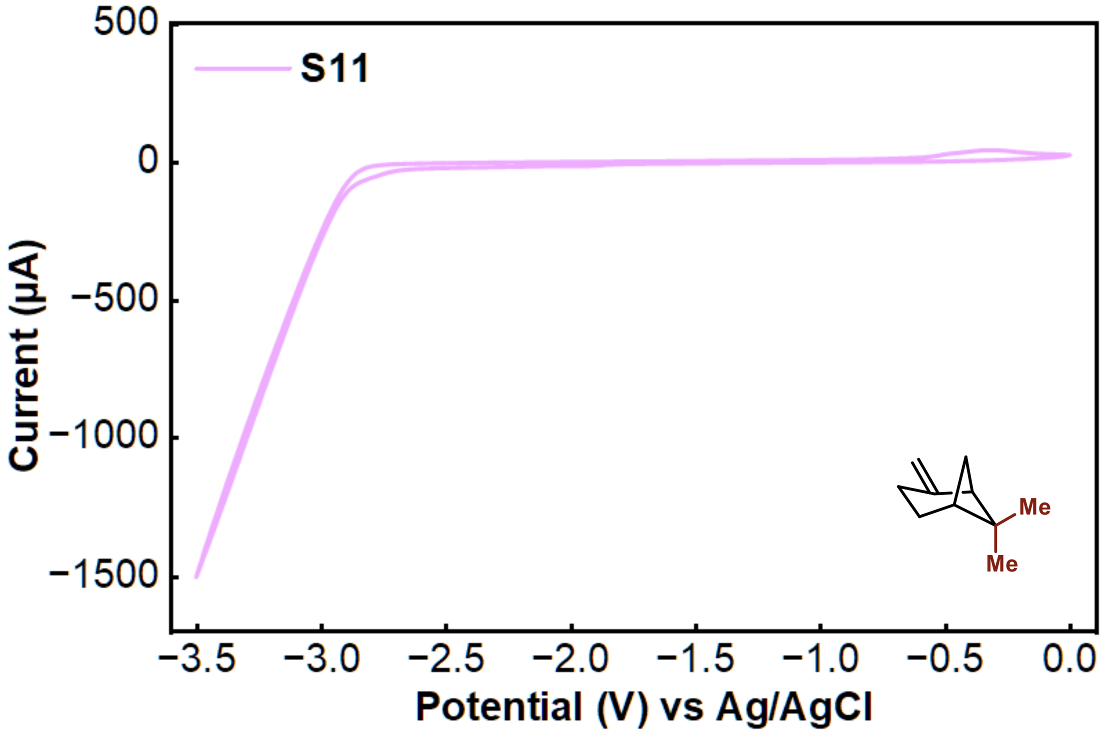

Compound S12

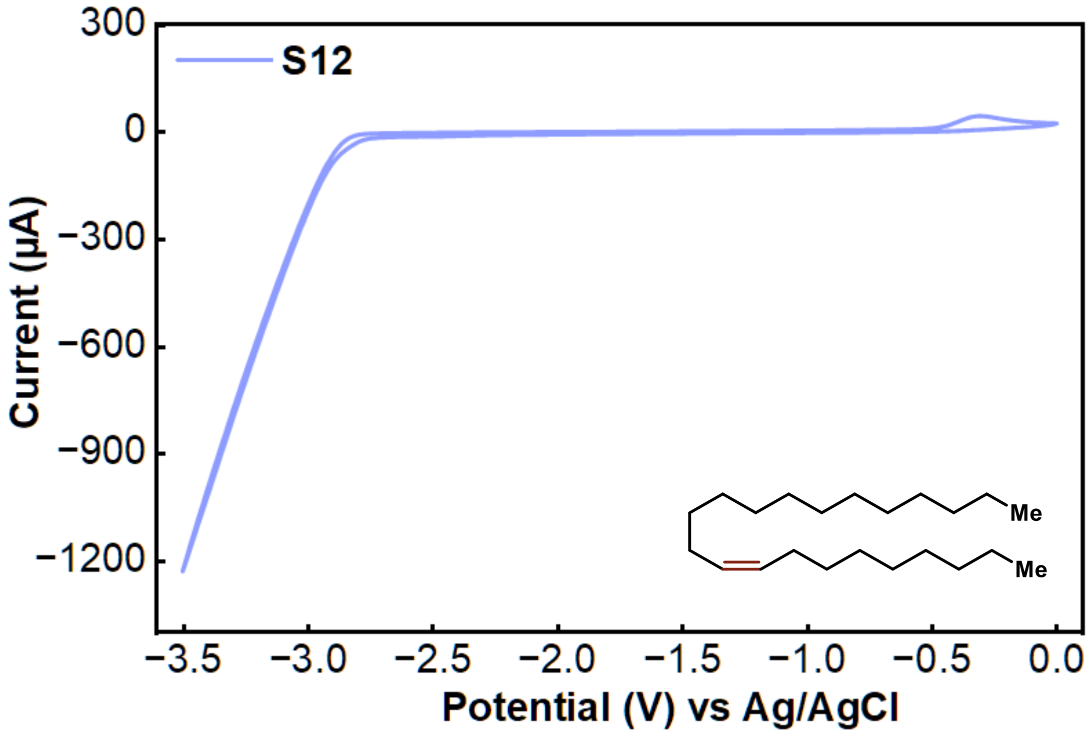

Compound S13

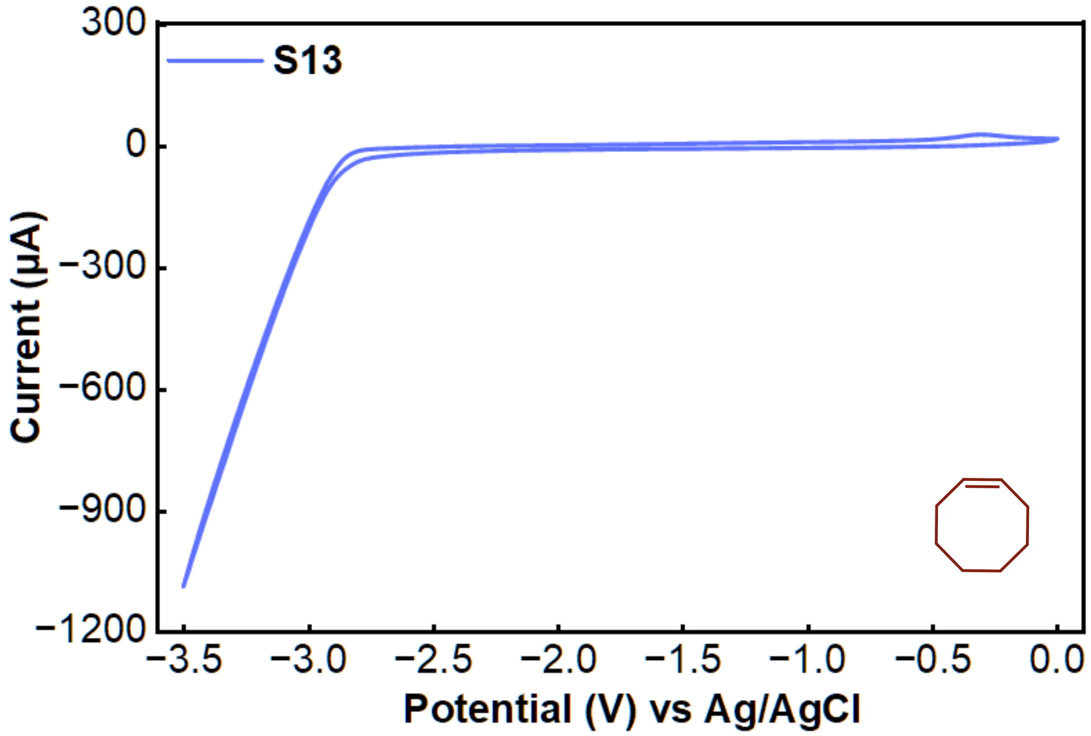

Compound S16

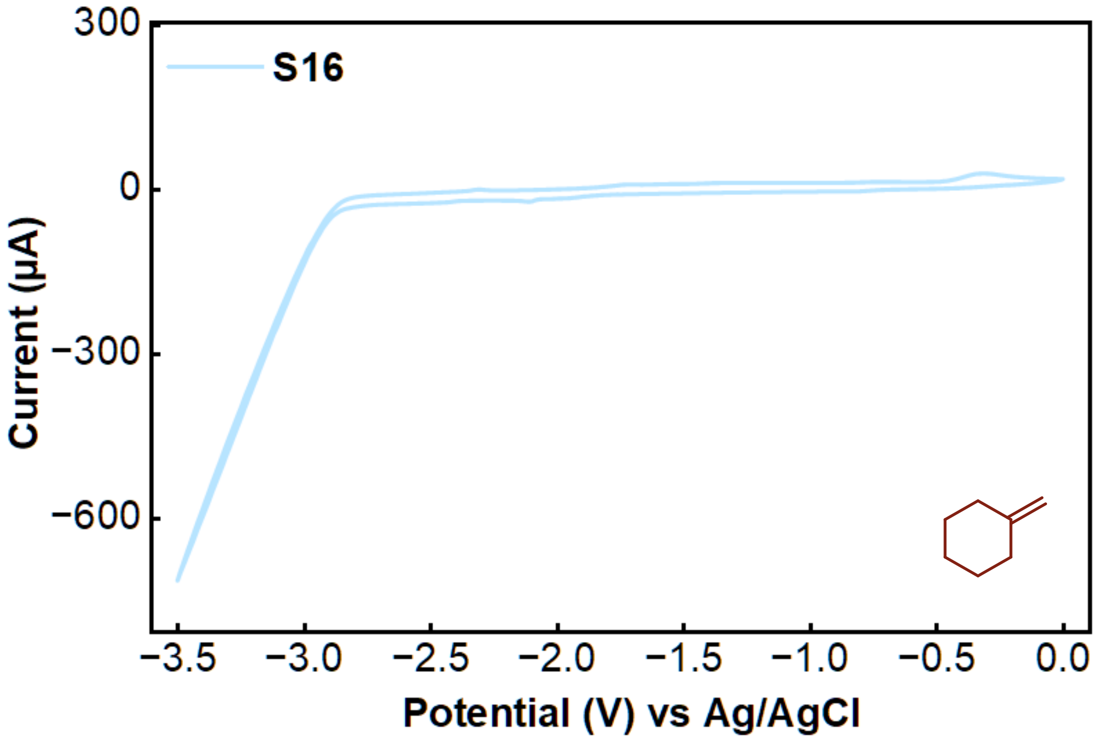

Compound S17

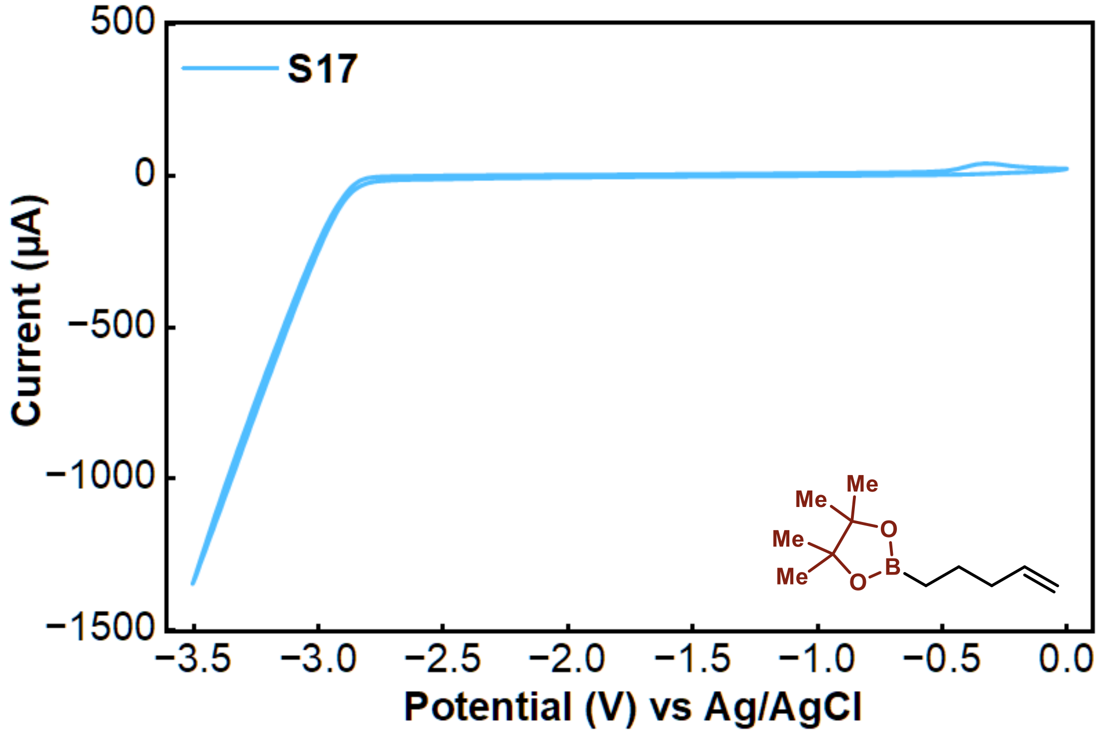

5. Unexpected products and limitations

5.1 Unexpected products

Compound 1aa'

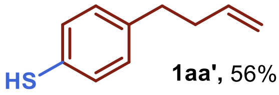

1aa', 56%

Following the General Procedure 1 and quenched by HCl (3.2 M), the corresponding olefin (1.00 mmol, 178 mg) afforded 91 mg (56% isolated yield) of the titled compound **1aa'** as a colorless oil after purification by silica gel column chromatography (eluent: petroleum ether, stained with I_2).

Physical Appearance: colorless oil

^{1}H NMR (300 MHz, $CDCl_3$): δ 7.11 (d, J = 8.3 Hz, 2H), 6.97 (d, J = 8.4 Hz, 2H), 5.74 (ddt, J = 16.8, 10.2, 6.5 Hz, 1H), 5.00 – 4.85 (m, 2H), 3.30 (s, 1H), 2.64 – 2.51 (m, 2H), 2.31 – 2.16 (m, 2H).

^{13}C NMR (75 MHz, $CDCl_3$): δ 139.7, 137.9, 129.9, 129.3, 127.4, 115.2, 35.5, 34.9.

HRMS (ESI-TOF) m/z: $[2M + H]^+$ Calcd for $C_{20}H_{25}S_2$ 329.1393; Found 329.1389.

Compound 1ab'

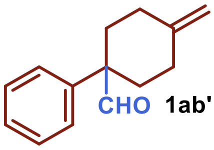

1ab', 27%

Following the General Procedure 1 and quenched by HCl (3.2 M), the corresponding olefin (1.00 mmol, 197 mg) afforded 75 mg (27% isolated yield) of the titled compound **1ab'** as a colorless oil after purification by silica gel column chromatography (eluent: petroleum ether/ethyl acetate = 10/1, stained with DNP).

Physical Appearance: colorless oil

^{1}H NMR (300 MHz, $CDCl_3$): δ 9.34 (s, 1H), 7.34 – 7.12 (m, 5H), 4.58 (s, 2H), 2.39 – 2.04 (m, 6H), 1.96 – 1.79 (m, 2H).

^{13}C NMR (75 MHz, $CDCl_3$): δ 201.8, 147.4, 138.8, 129.1, 127.5, 127.2, 108.0, 54.3, 32.4, 31.4.

HRMS (ESI-TOF) m/z: $[M + Na]^+$ Calcd for $C_{14}H_{16}NaO$ 223.1093; Found 223.1095.

5.2. Limitations

Listed here are substrates failed under our reaction condition, and can be generally separated into 3 cases: mismatch of reductive potential, too much steric hindrance, liable during workup.

Mismatch of reductive potential

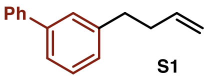

S1

depending on GC-MS reaction failed due to **Birch reduction**

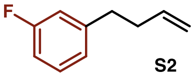

S2

depending on GC-MS reaction failed due to **dehalogenation**

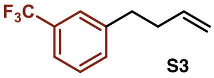

S3

depending on GC-MS reaction failed due to **Birch reduction**

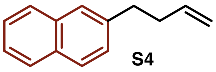

S4

depending on GC-MS reaction failed due to **Birch reduction**

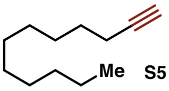

S5

depending on GC-MS reaction failed due to **alkyne reduction**

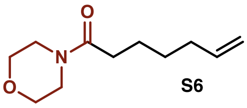

S6

depending on GC-MS reaction failed due to **amide reduction**

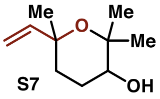

S7

depending on GC-MS reaction failed due to **allylic reduction**

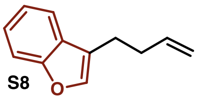

S8

depending on GC-MS reaction failed due to **ring opening**

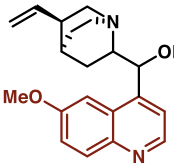

S9

depending on GC-MS reaction failed due to **Birch reduction**

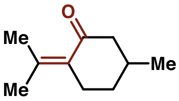

S10

depending on GC-MS reaction failed due to **conjugated reduction**

Too much steric hindrance

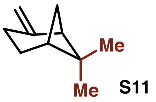

S11

depending on GC-MS most of S.M. unreacted

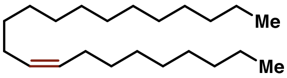

S12

depending on GC-MS most of S.M. unreacted

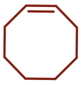

S13

depending on GC-MS most of S.M. unreacted less then 15% GC yield

Liability during workup

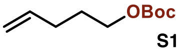

S14

hydrolysis during workup

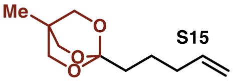

S15

hydrolysis during workup

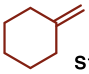

S16

low boiling point

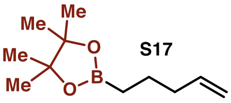

S17

severe taillig on column

Other solvent system

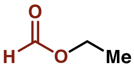

depending on GC-MS no desired product found

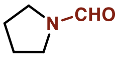

depending on GC-MS no desired product found

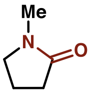

depending on GC-MS no desired product found

6. References

(1) Jiang, C.; Wu, Y.; Zhang, Y.; Zong, J.; Wang, N.; Liu, G.; Liu, R.; Yu, H. Supramolecular Modulation for Selective Mechanochemical Iron-Catalyzed Olefin Oxidation. *Angewandte Chemie International Edition* **2025**, *64*, e202413901. DOI: https://doi.org/10.1002/anie.202413901.

(2) Wang, Y.-F.; Gao, Y.-R.; Mao, S.; Zhang, Y.-L.; Guo, D.-D.; Yan, Z.-L.; Guo, S.-H.; Wang, Y.-Q. Wacker-Type Oxidation and Dehydrogenation of Terminal Olefins Using Molecular Oxygen as the Sole Oxidant without Adding Ligand. *Organic Letters* **2014**, *16*, 1610-1613. DOI: 10.1021/ol500218p.

(3) Hou, X.; Liu, H.; Huang, H. Iron-catalyzed fluoroalkylative alkylsulfonylation of alkenes via radical-anion relay. *Nature Communications* **2024**, *15*, 1480. DOI: 10.1038/s41467-024-45867-y.

(4) Feng, Q.; Wang, Q.; Zhu, J. Oxidative rearrangement of 1,1-disubstituted alkenes to ketones. *Science* **2023**, *379*, 1363-1368. DOI: doi:10.1126/science.adg3182.

(5) Wu, S.-F.; Yu, Y.; Yuan, Y.; Li, Z.; Ye, K.-Y. Electrochemical Synthesis of β-Fluoroselenides. *European Journal of Organic Chemistry* **2022**, *2022*, e202201032. DOI: https://doi.org/10.1002/ejoc.202201032.

(6) Law, J. A.; Bartfield, N. M.; Frederich, J. H. Site-Specific Alkene Hydromethylation via Protonolysis of Titanacyclobutanes. *Angewandte Chemie International Edition* **2021**, *60*, 14360-14364. DOI: https://doi.org/10.1002/anie.202103278.

(7) Ahrweiler, E.; Selmani, A.; Schoenebeck, F. Base-Catalyzed Remote Hydrogermylation of Olefins. *Angewandte Chemie International Edition* **2025**, *64*, e202503573. DOI: https://doi.org/10.1002/anie.202503573.

(8) Munnuri, S.; Adebesin, A. M.; Paudyal, M. P.; Yousufuddin, M.; Dalipe, A.; Falck, J. R. Catalyst-Controlled Diastereoselective Synthesis of Cyclic Amines via C–H Functionalization. *Journal of the American Chemical Society* **2017**, *139*, 18288-18294. DOI: 10.1021/jacs.7b09901.

(9) Nicolaou, K. C.; Reingruber, R.; Sarlah, D.; Bräse, S. Enantioselective Intramolecular Friedel−Crafts-Type α-Arylation of Aldehydes. *Journal of the American Chemical Society* **2009**, *131*, 2086-2087. DOI: 10.1021/ja809405c.

(10) Nakazaki, A.; Nakane, Y.; Ishikawa, Y.; Yotsu-Yamashita, M.; Nishikawa, T. Asymmetric synthesis of crambescin A–C carboxylic acids and their inhibitory activity on voltage-gated sodium channels. *Organic & Biomolecular Chemistry* **2016**, *14*, 5304-5309, 10.1039/C6OB00914J. DOI: 10.1039/C6OB00914J.

(11) Hayashi, Y.; Gotoh, H.; Tamura, T.; Yamaguchi, H.; Masui, R.; Shoji, M. Cysteine-Derived Organocatalyst in a Highly Enantioselective Intramolecular Michael Reaction. *Journal of the American Chemical Society* **2005**, *127*, 16028-16029. DOI: 10.1021/ja055740s.

(12) Breman, A. C.; Ruiz-Olalla, A.; van Maarseveen, J. H.; Ingemann, S.; Hiemstra, H. Synthesis of Quinuclidines by Intramolecular Silver-Catalysed Amine Additions to Alkynes. *European Journal of Organic Chemistry* **2014**, *2014*, 7413-7425. DOI: https://doi.org/10.1002/ejoc.201403099.

(13) Mbofana, C. T.; Chong, E.; Lawniczak, J.; Sanford, M. S. Iron-Catalyzed Oxyfunctionalization of Aliphatic Amines at Remote Benzylic C–H Sites. *Organic Letters* **2016**, *18*, 4258-4261. DOI: 10.1021/acs.orglett.6b02003.

(14) Dang, T. T.; Ramalingam, B.; Seayad, A. M. Efficient Ruthenium-Catalyzed N-Methylation of Amines Using Methanol. *ACS Catalysis* **2015**, *5*, 4082-4088. DOI: 10.1021/acscatal.5b00606.

(15) Tuokko, S.; Pihko, P. M. Palladium on Charcoal as a Catalyst for Stoichiometric Chemo- and Stereoselective Hydrosilylations and Hydrogenations with Triethylsilane. *Organic Process Research &*

Development **2014**, *18*, 1740-1751. DOI: 10.1021/op5003209.
(16) Ma, S.; Liu, J.; Li, S.; Chen, B.; Cheng, J.; Kuang, J.; Liu, Y.; Wan, B.; Wang, Y.; Ye, J.; et al. Development of a General and Practical Iron Nitrate/TEMPO-Catalyzed Aerobic Oxidation of Alcohols to Aldehydes/Ketones: Catalysis with Table Salt. *Advanced Synthesis & Catalysis* **2011**, *353*, 1005-1017. DOI: https://doi.org/10.1002/adsc.201100033.

7. NMR data

NMR

Starting materials

Compound **1b**: ^{1}H NMR (300 MHz, $CDCl_3$)

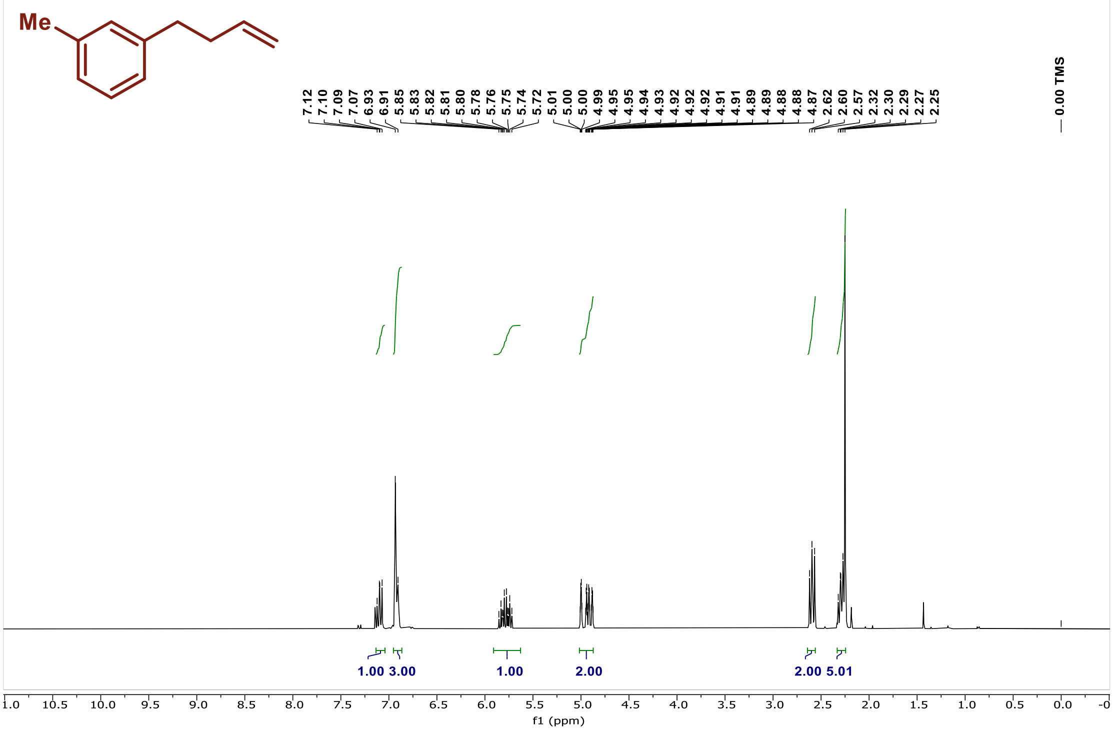

Compound **1b**: ^{13}C NMR (75 MHz, $CDCl_3$)

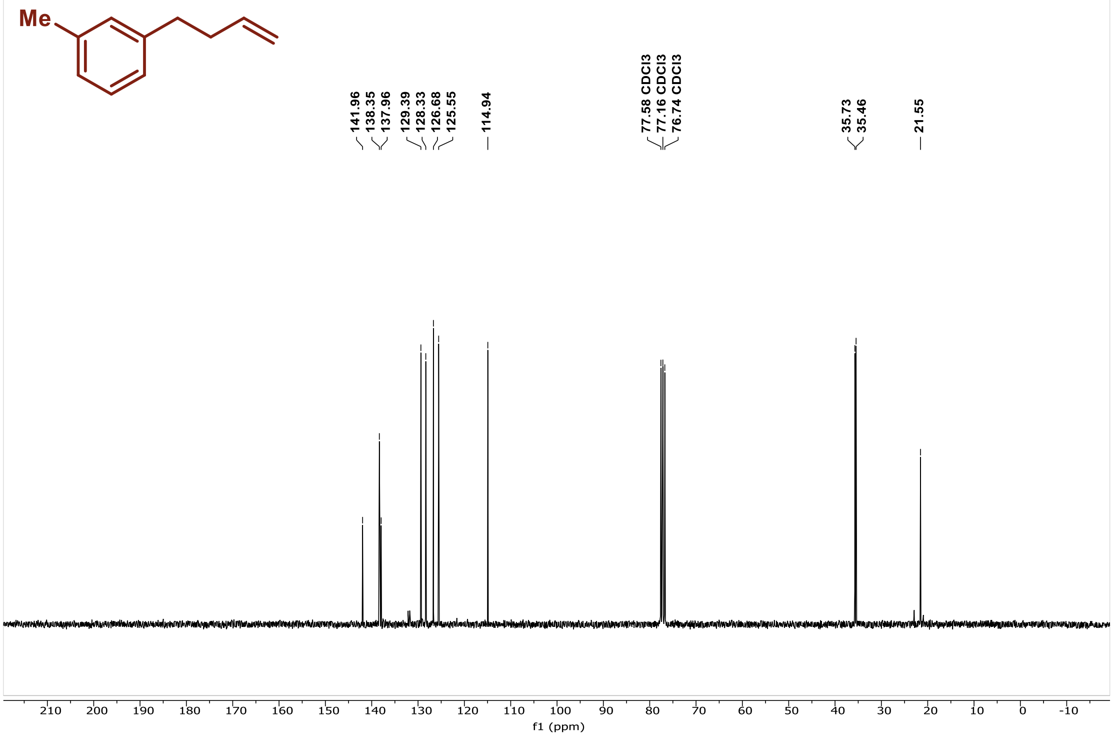

Compound **1c**: ^{1}H NMR (300 MHz, $CDCl_3$)

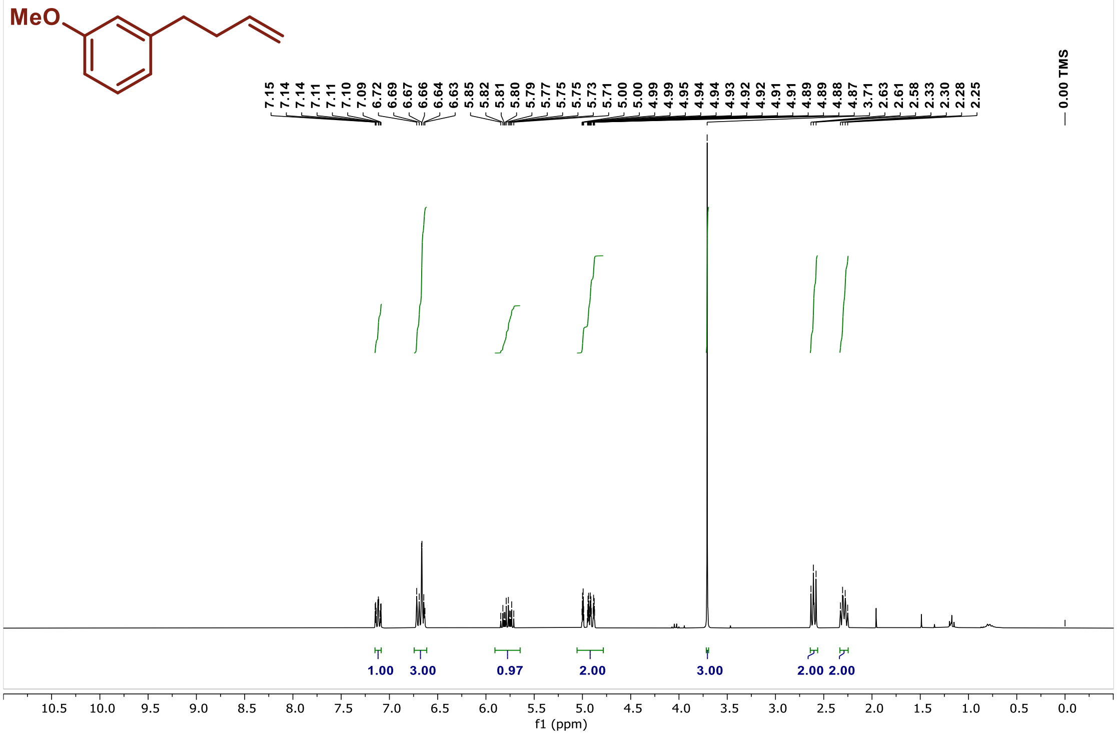

Compound **1c**: ^{13}C NMR (75 MHz, $CDCl_3$)

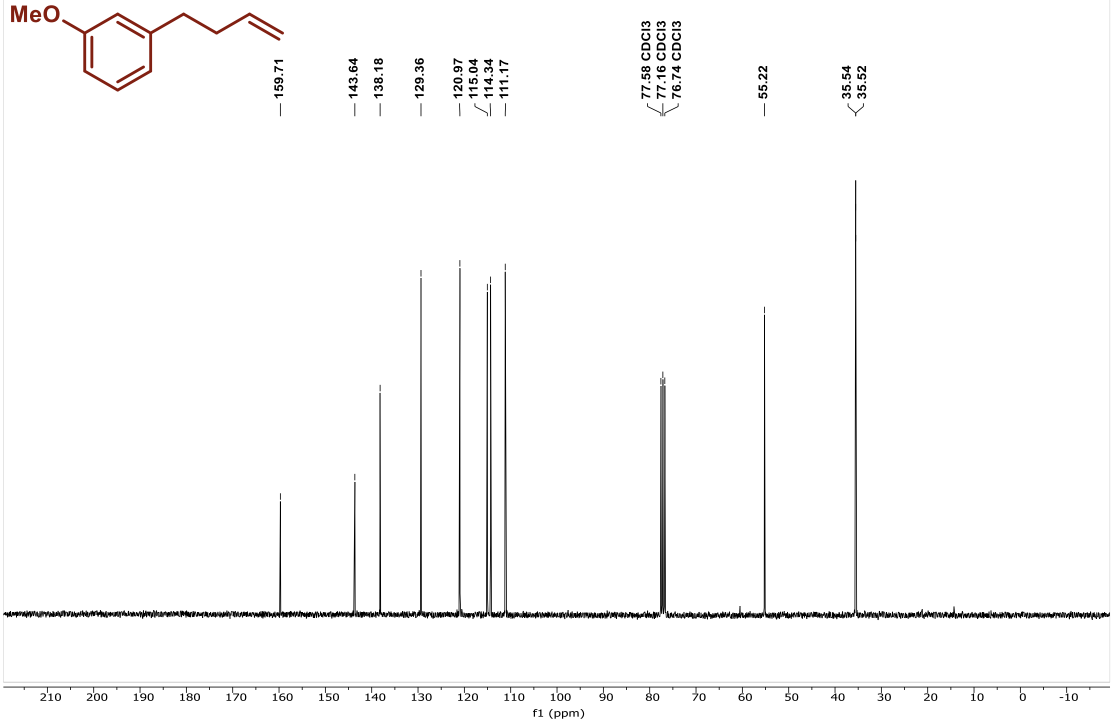

Compound **1e**: ^{1}H NMR (300 MHz, $CDCl_3$)

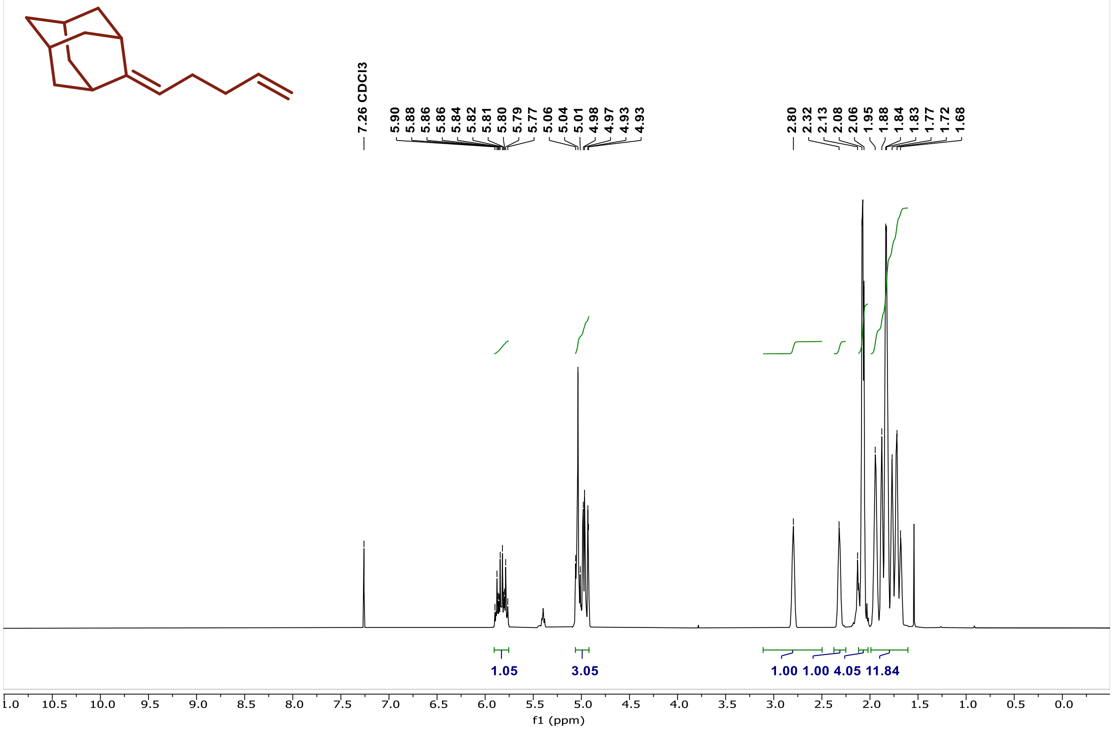

Compound **1e**: ^{13}C NMR (75 MHz, $CDCl_3$)

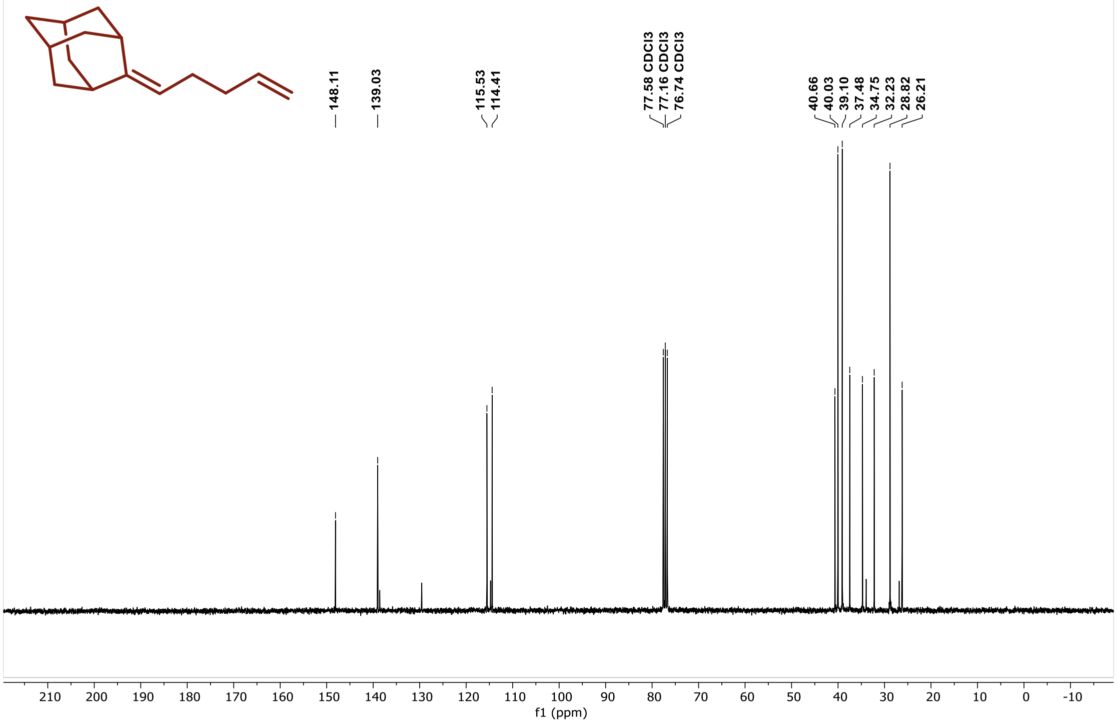

Compound **1f**: ^{1}H NMR (300 MHz, $CDCl_3$)

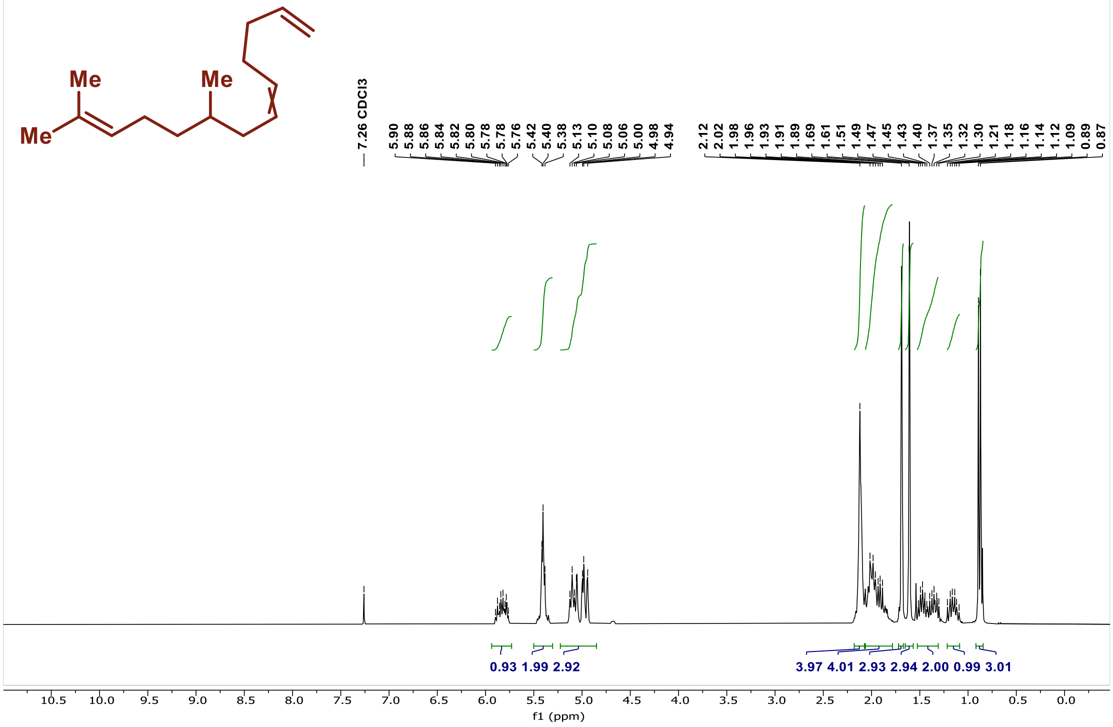

Compound **1f**: ^{13}C NMR (75 MHz, $CDCl_3$)

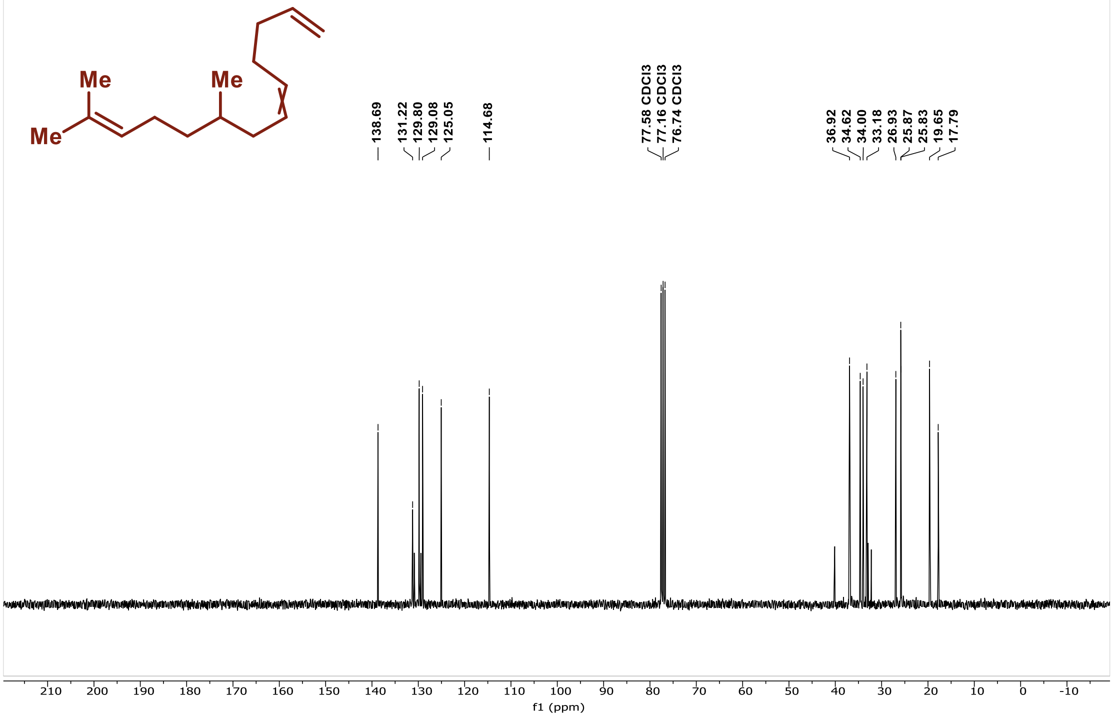

Compound **1g**: ^{1}H NMR (300 MHz, $CDCl_3$)

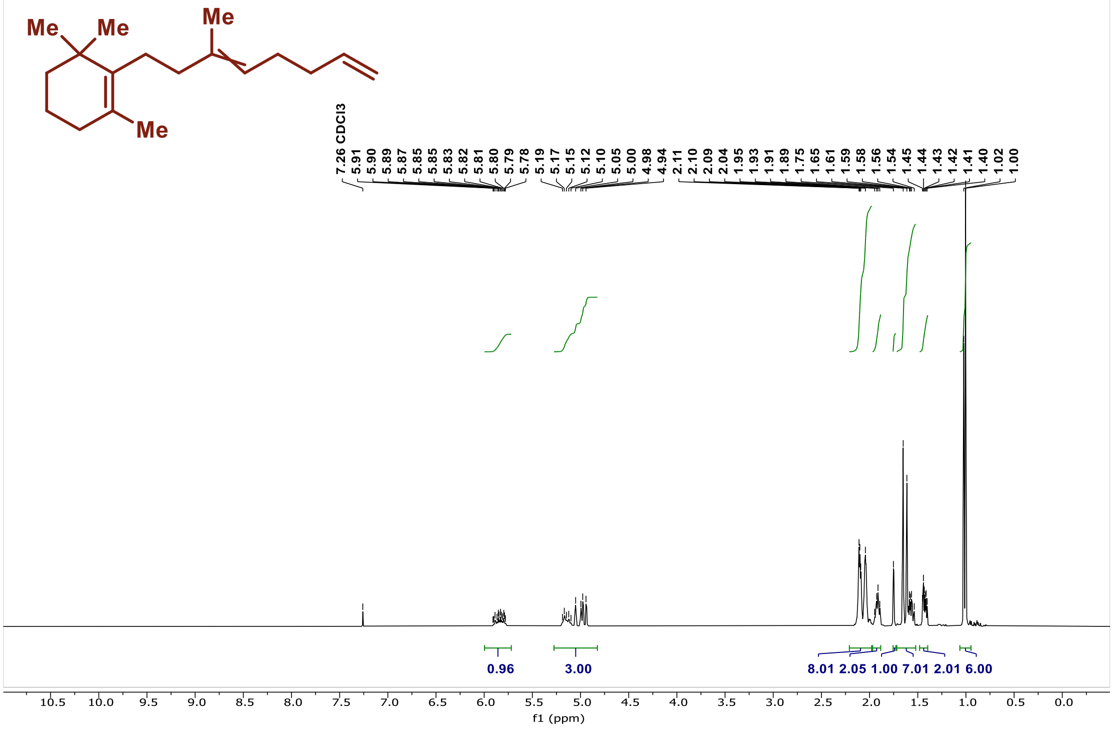

Compound **1g**: ^{13}C NMR (75 MHz, $CDCl_3$)

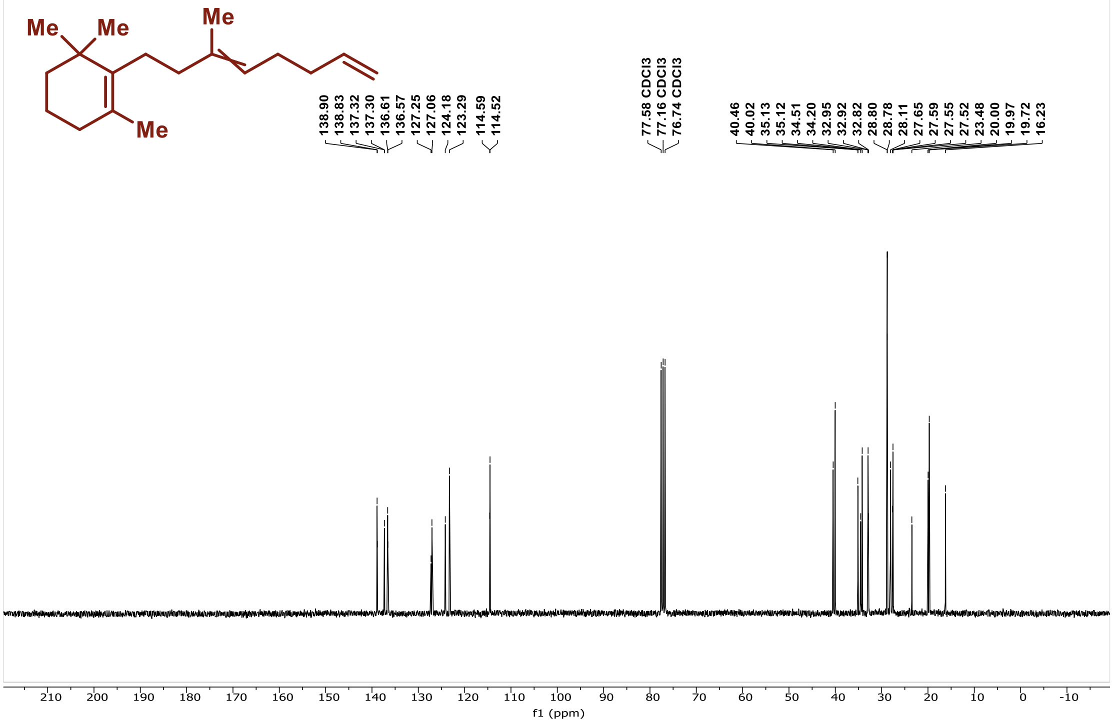

Compound **1h**: ^{1}H NMR (300 MHz, $CDCl_3$)

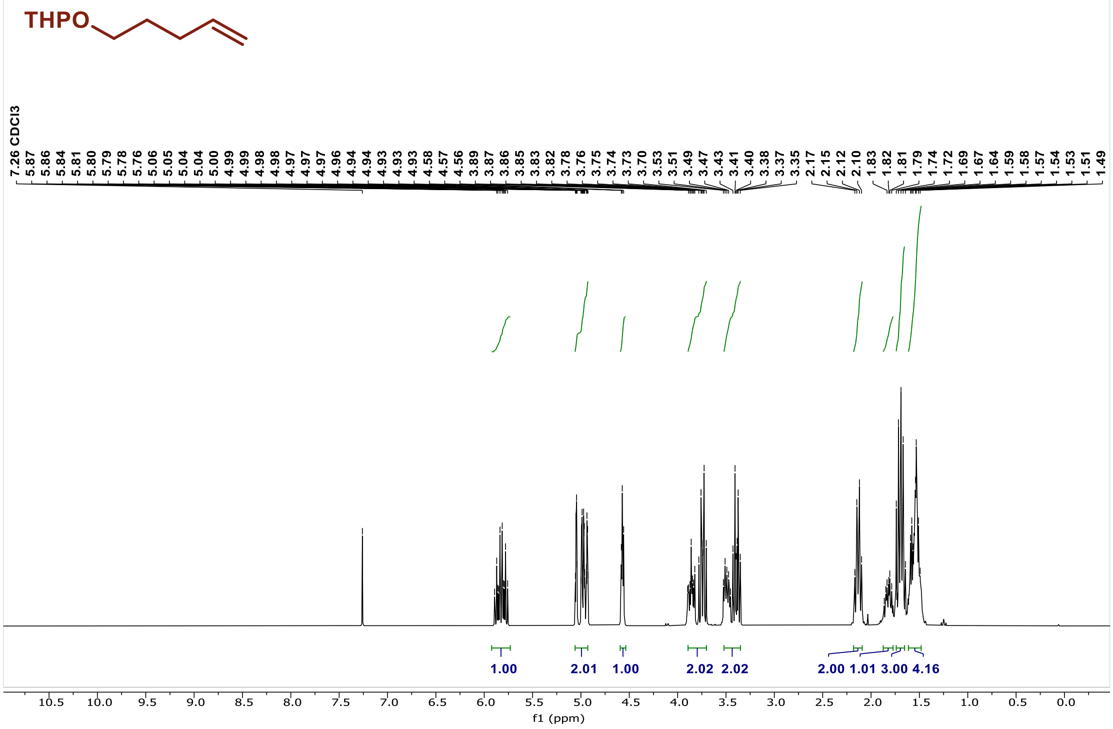

Compound **1h**: ^{13}C NMR (75 MHz, $CDCl_3$)

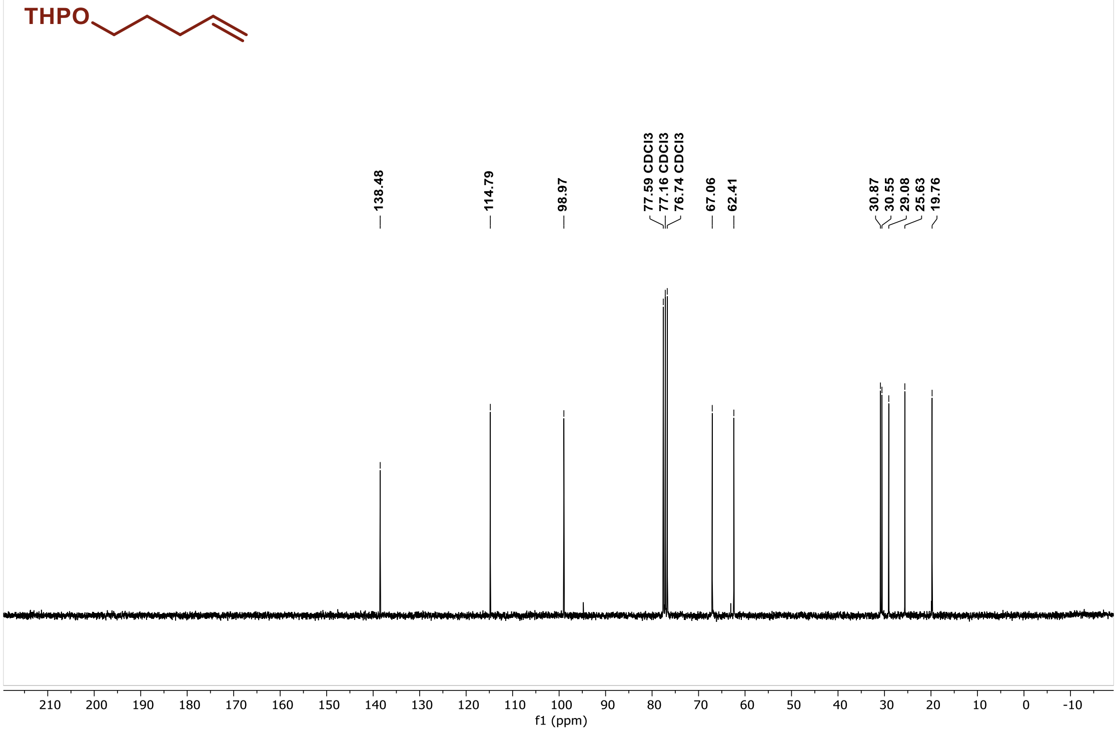

Compound **1i**: ^{1}H NMR (300 MHz, $CDCl_3$)

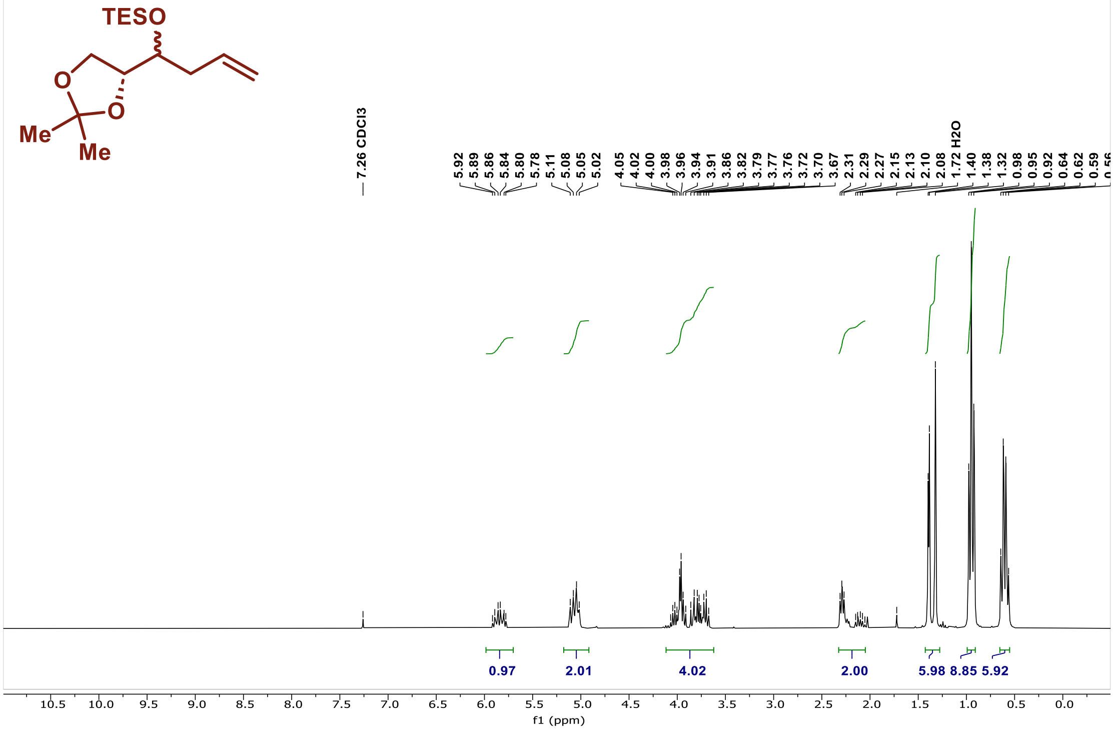

Compound **1i**: ^{13}C NMR (75 MHz, $CDCl_3$)

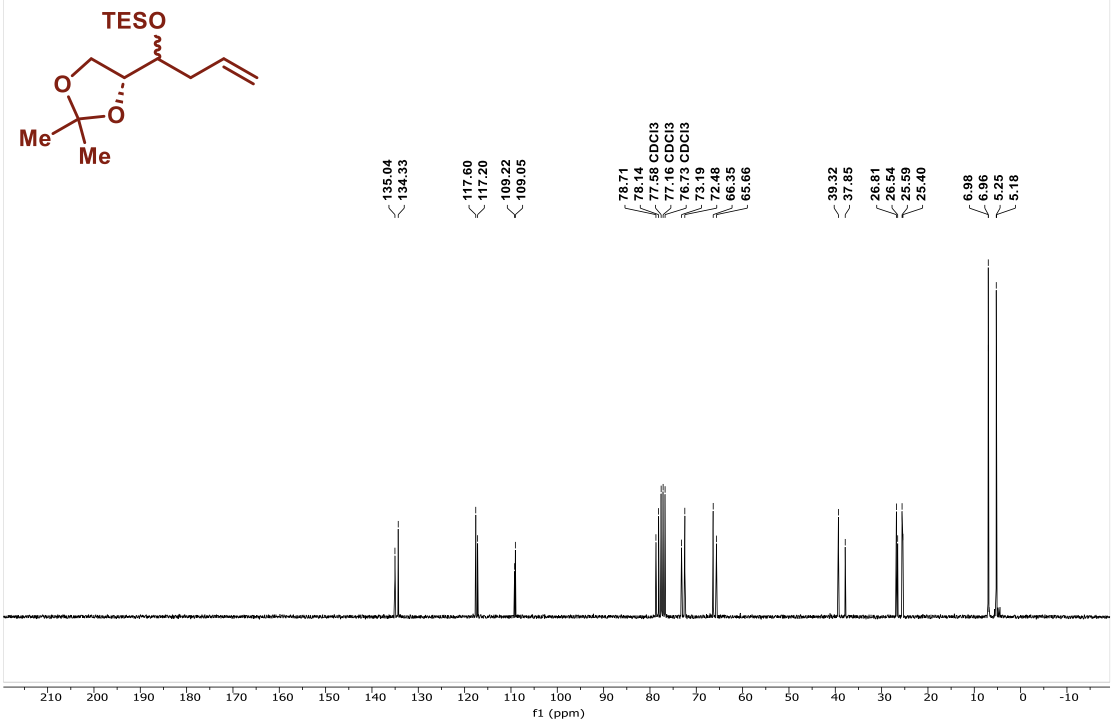

Compound **1j**: ^{1}H NMR (300 MHz, $CDCl_3$)

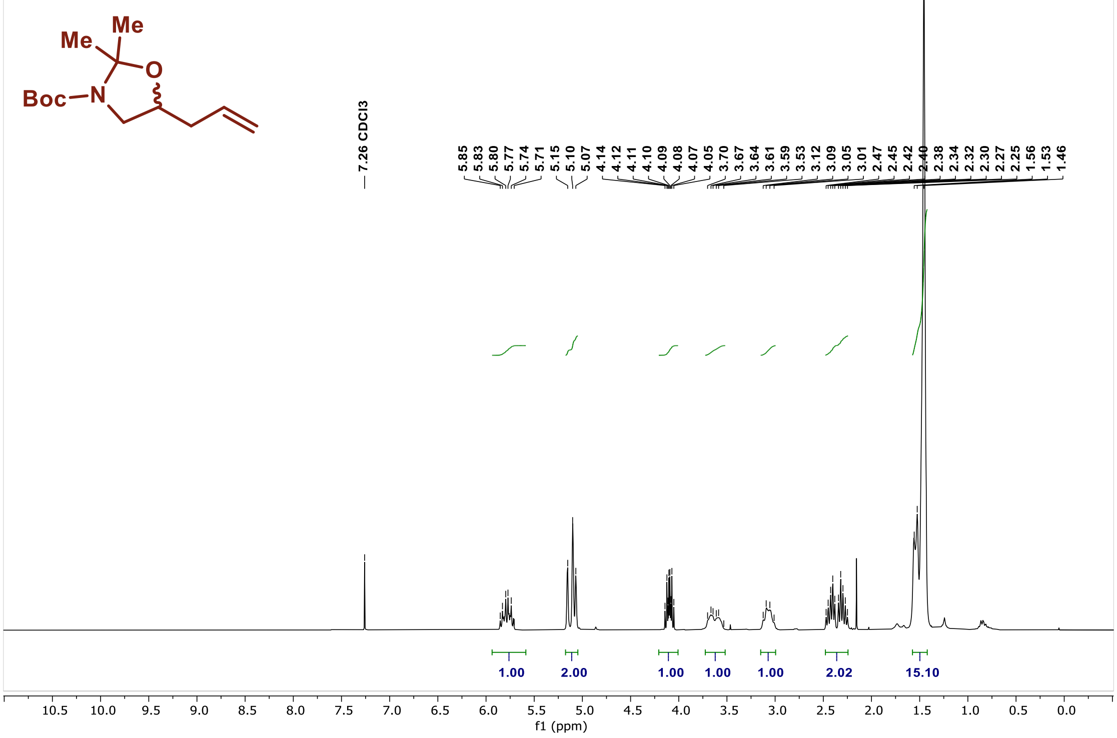

Compound **1j**: ^{13}C NMR (75 MHz, $CDCl_3$)

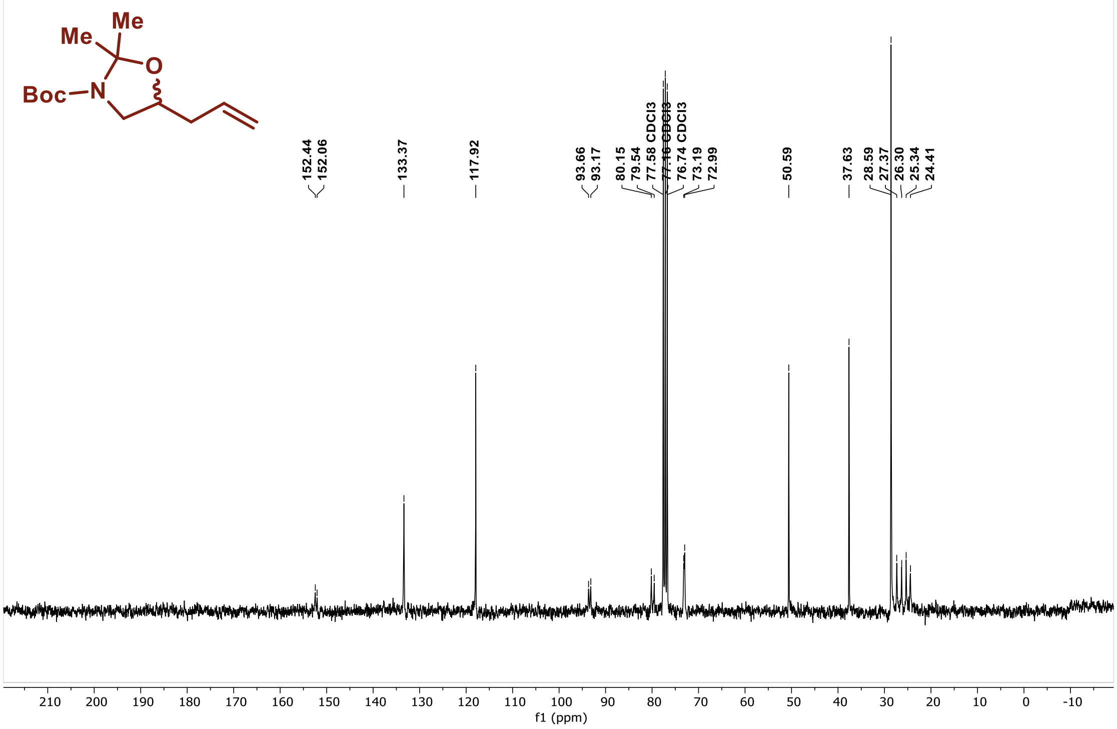

Compound **1k**: ^{1}H NMR (300 MHz, $CDCl_3$)

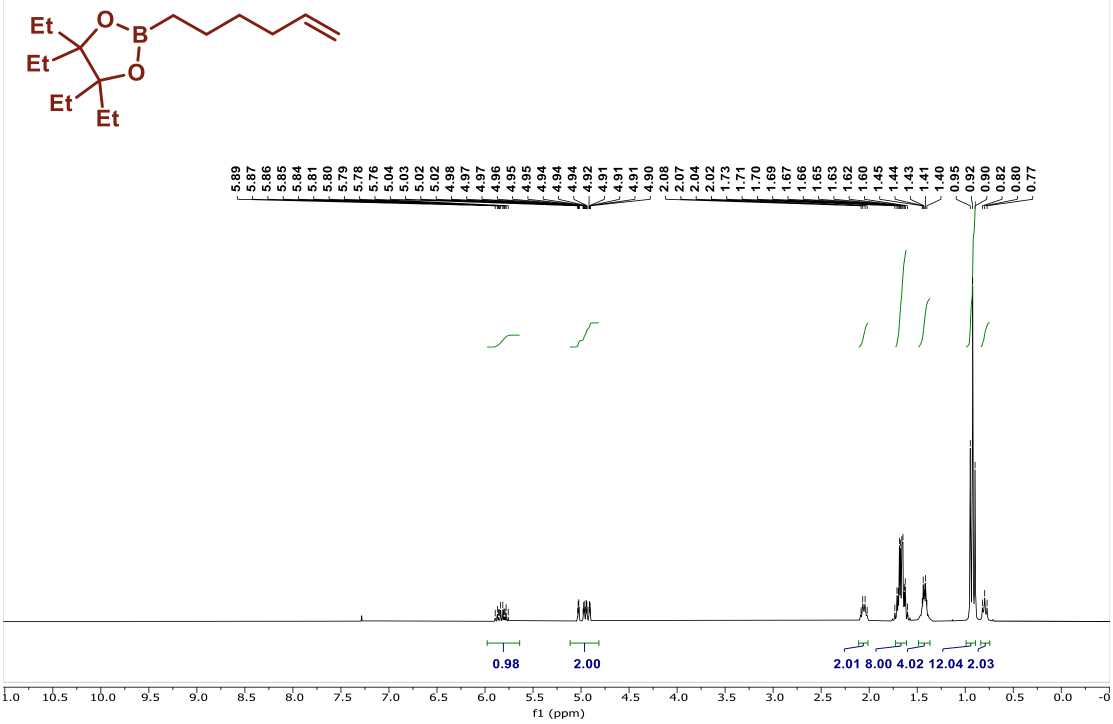

Compound **1k**: ^{13}C NMR (75 MHz, $CDCl_3$)

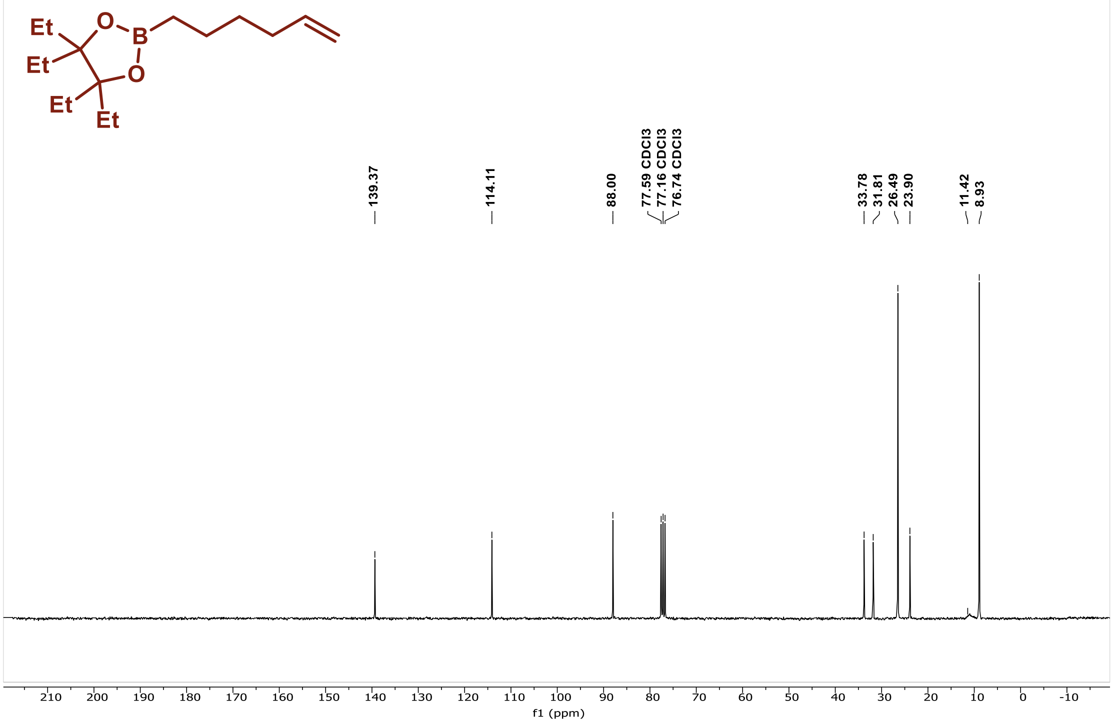

Compound **1l**: ^{1}H NMR (300 MHz, $CDCl_3$)

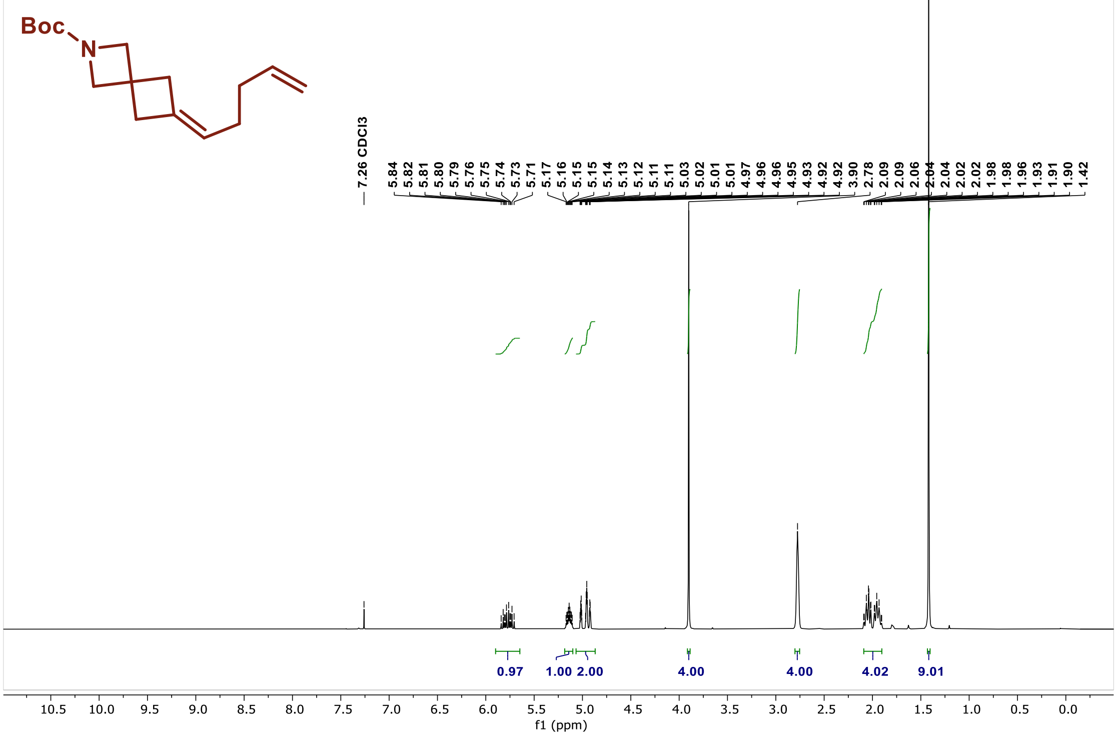

Compound **1l**: ^{13}C NMR (75 MHz, $CDCl_3$)

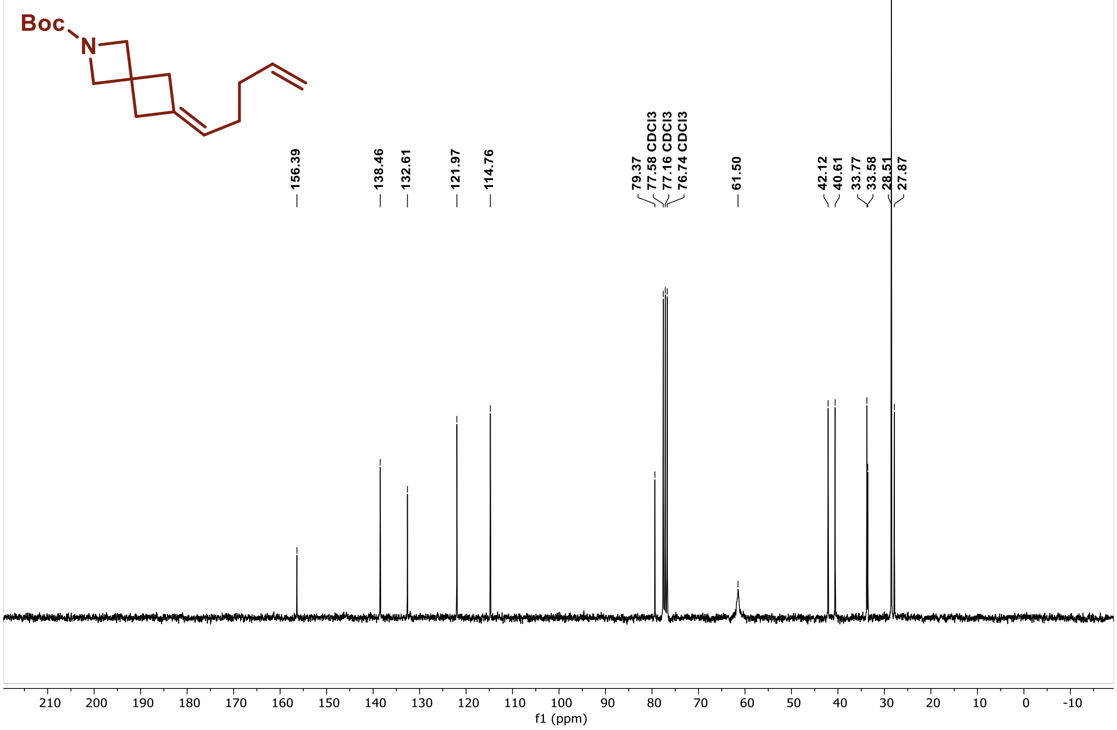

Compound **1m**: ^{1}H NMR (300 MHz, $CDCl_3$)

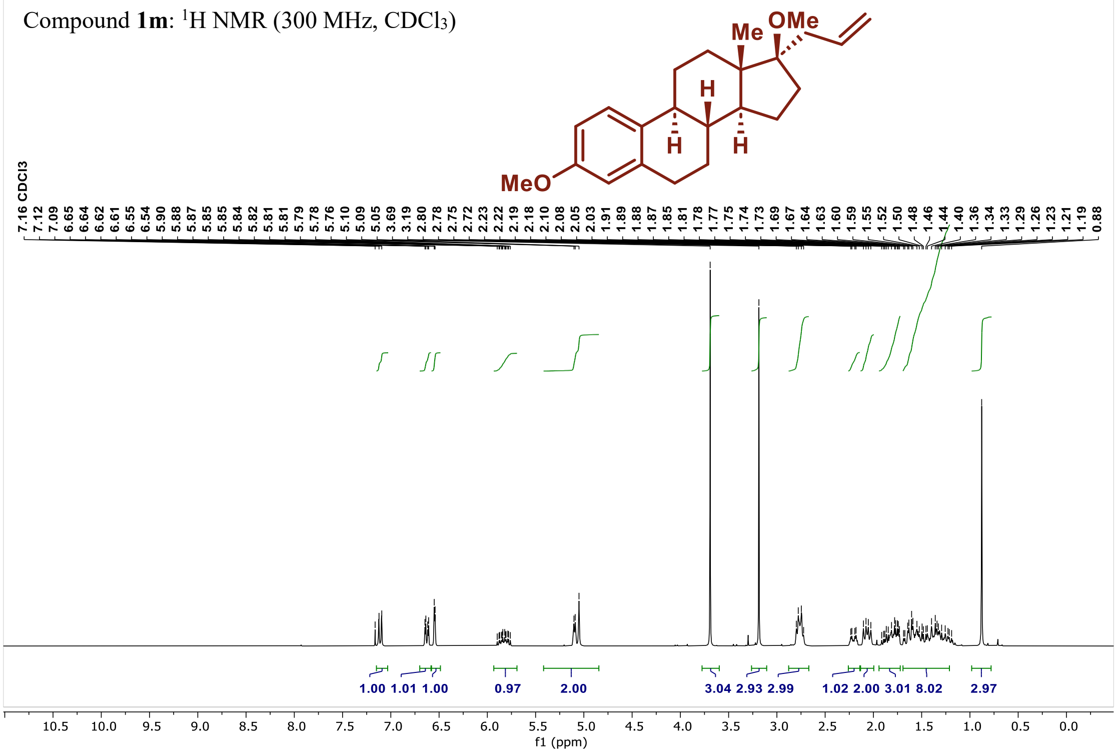

Compound **1m**: ^{13}C NMR (75 MHz, $CDCl_3$)

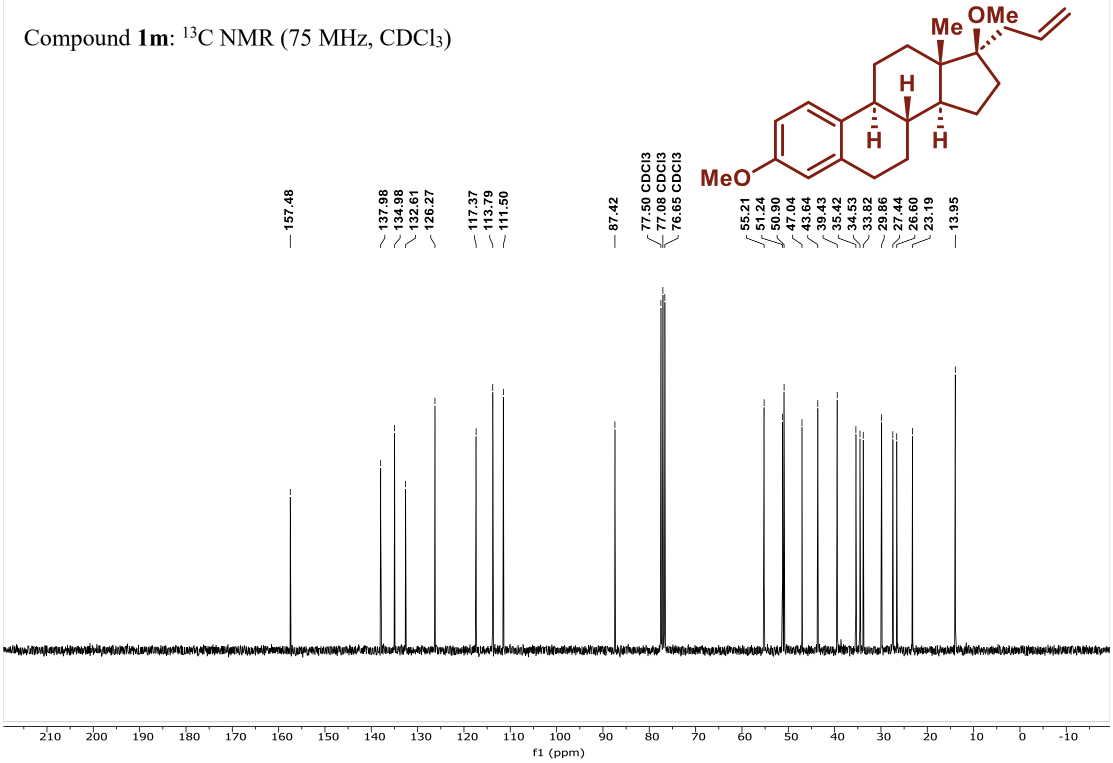

Compound **1m**: DEPT 135

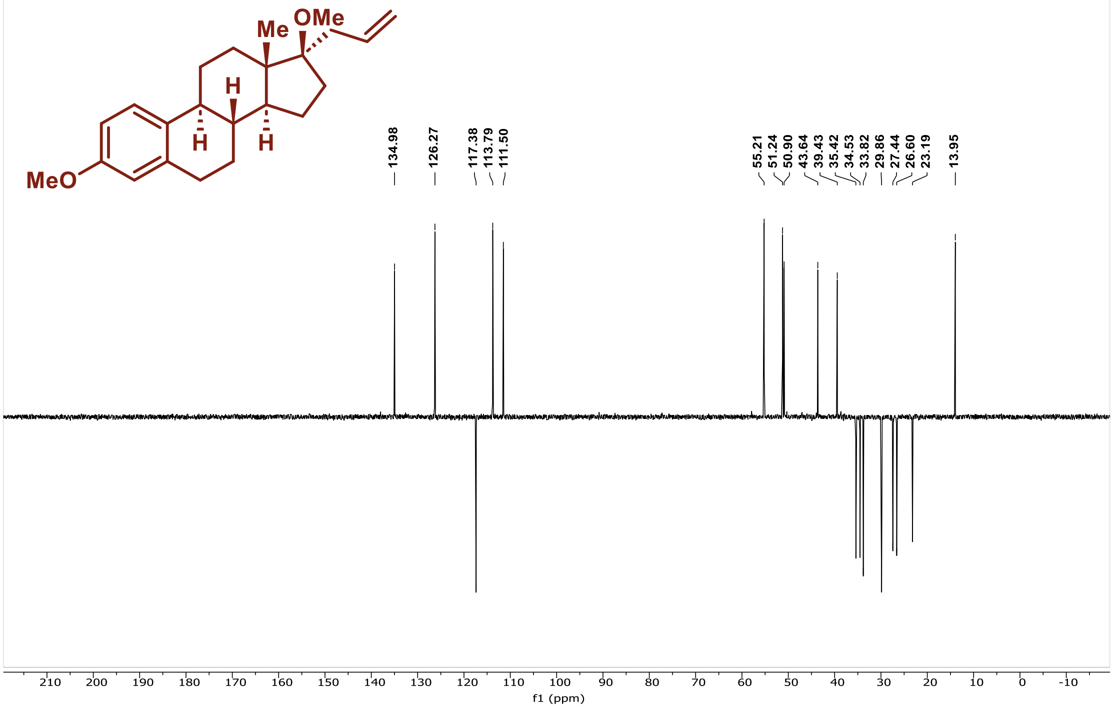

Compound **1m**: COSY

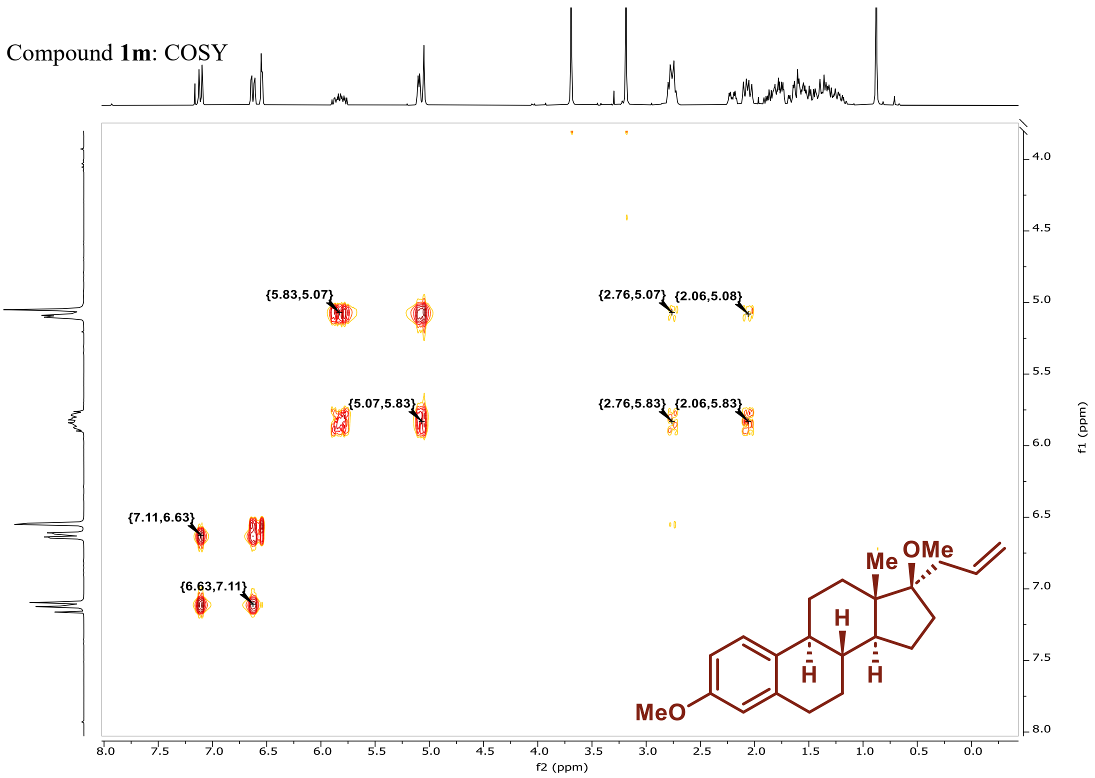

Compound **1m**: HSQC

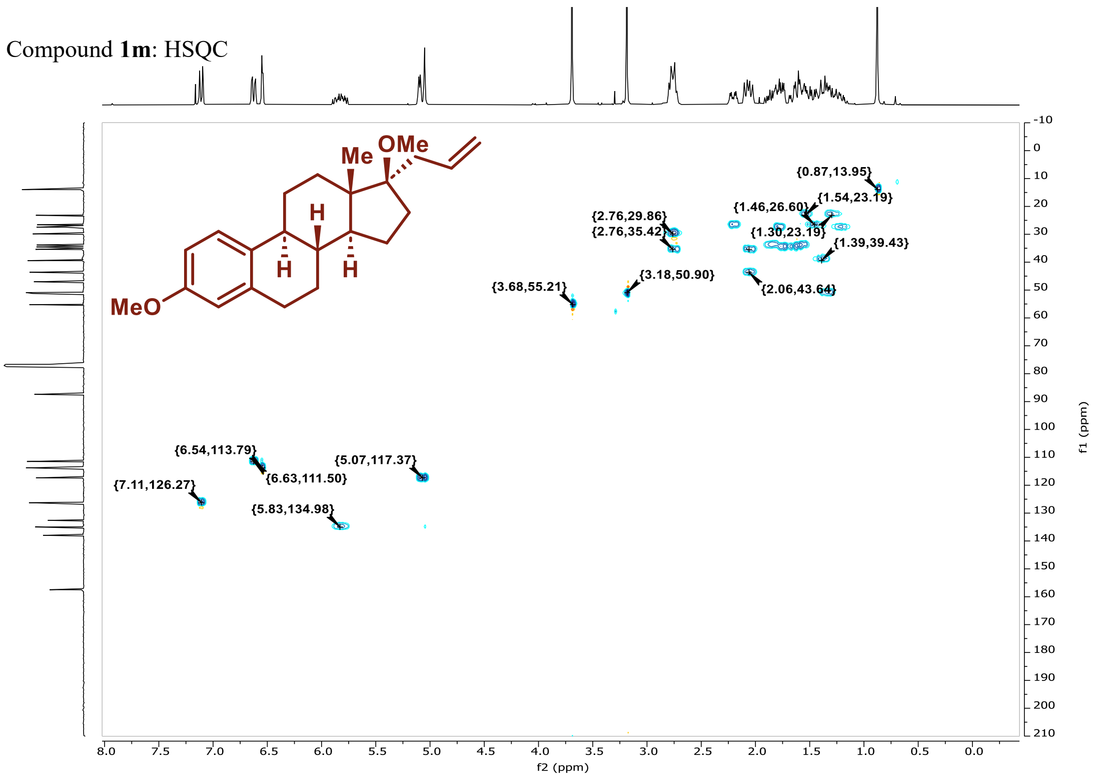

Compound **1m**: HMBC

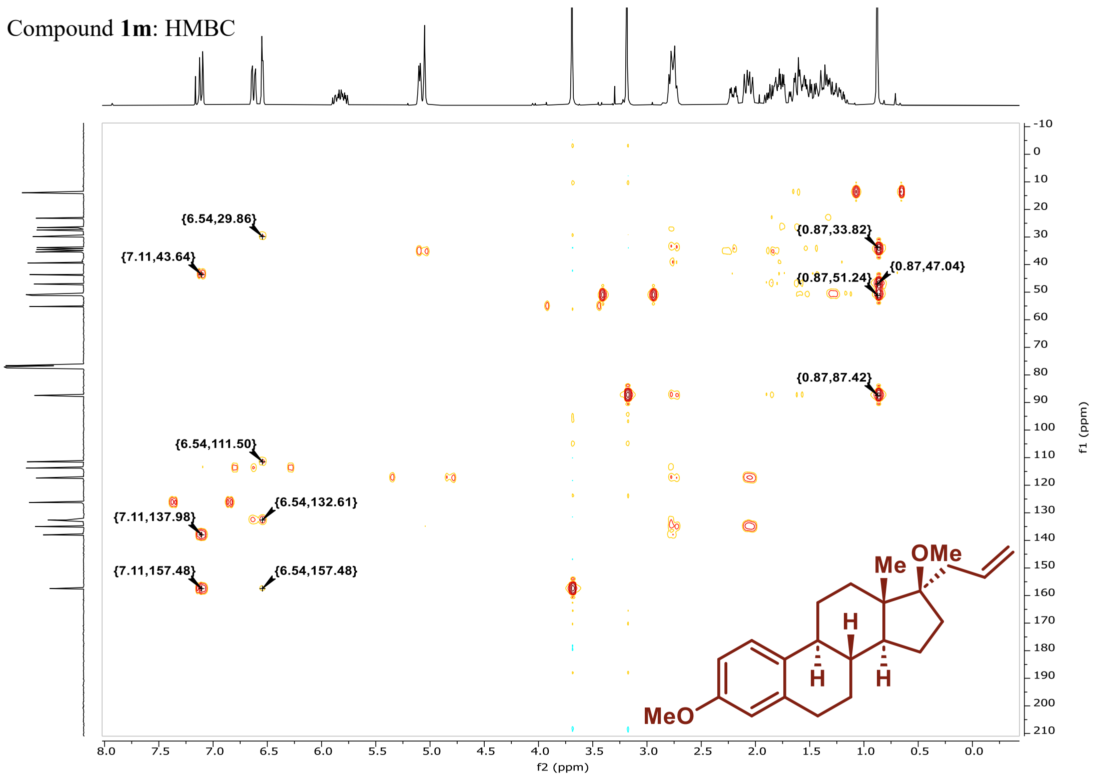

Compound **1m**: NOESY

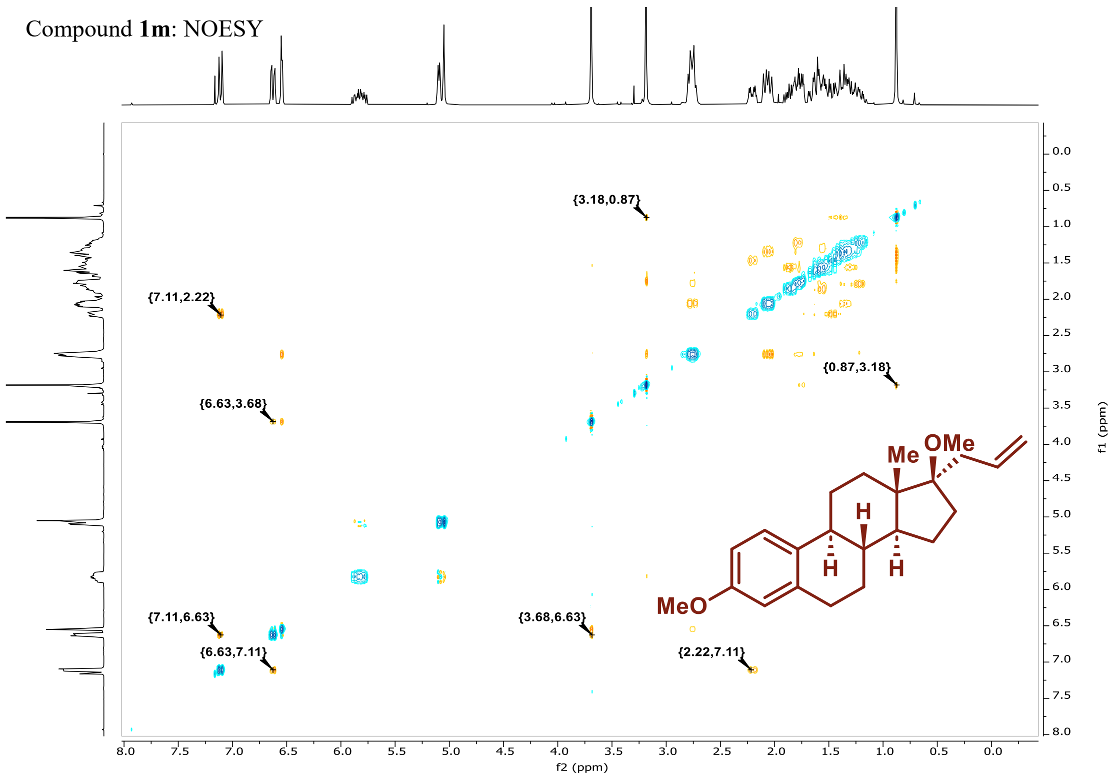

Compound **1n**: ^{1}H NMR (300 MHz, $CDCl_3$)

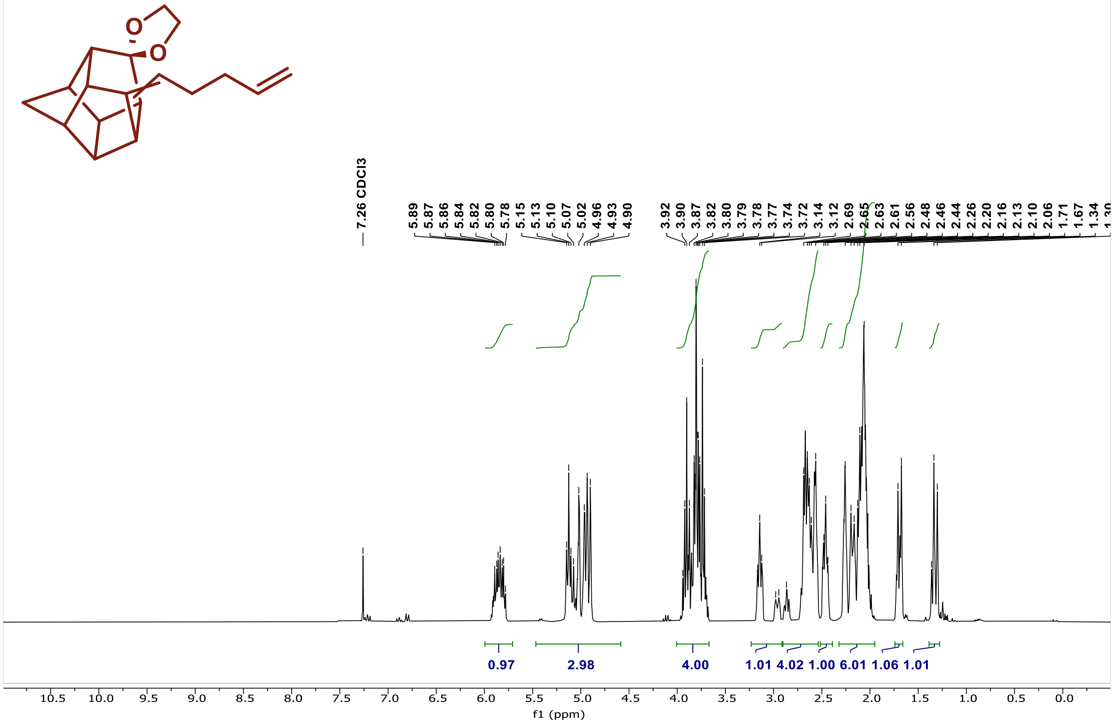

Compound **1n**: ^{13}C NMR (75 MHz, $CDCl_3$)

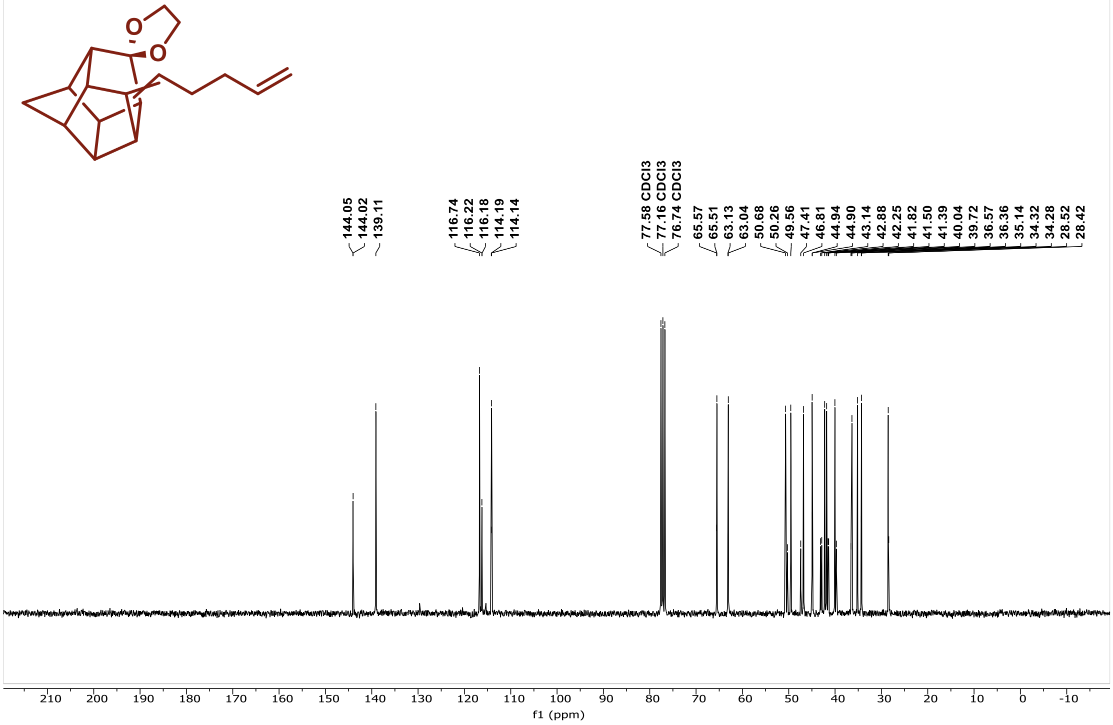

Compound **1o**: ^{1}H NMR (300 MHz, $CDCl_3$)

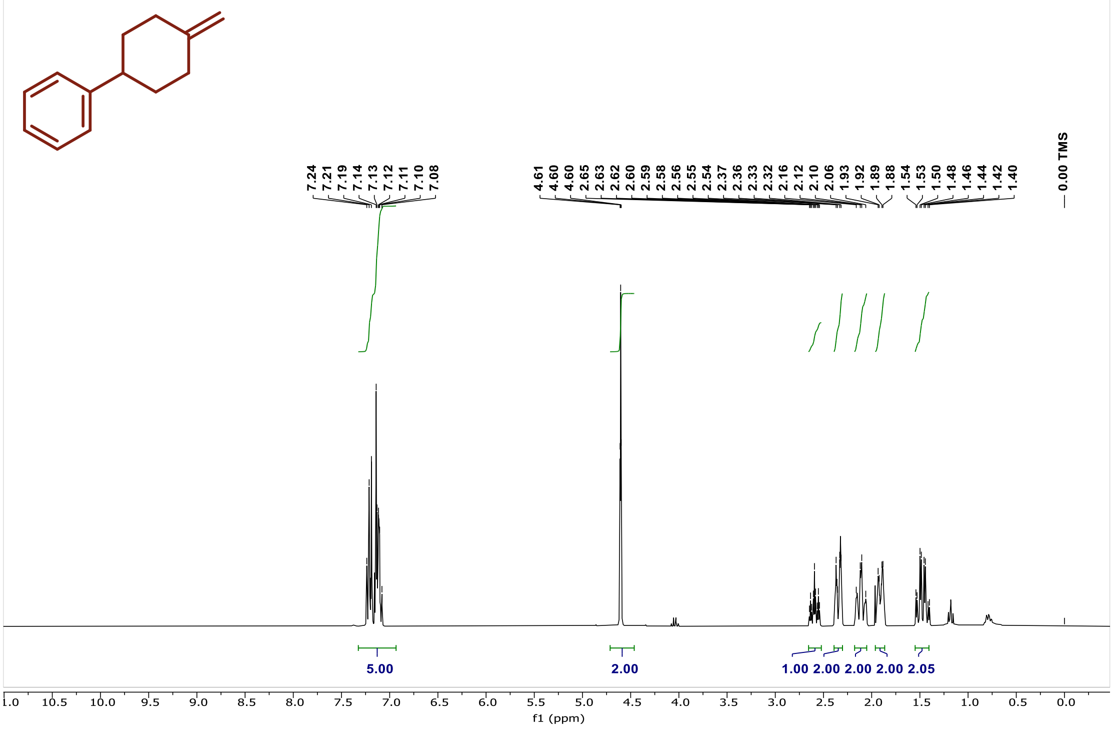

Compound **1o**: ^{13}C NMR (75 MHz, $CDCl_3$)

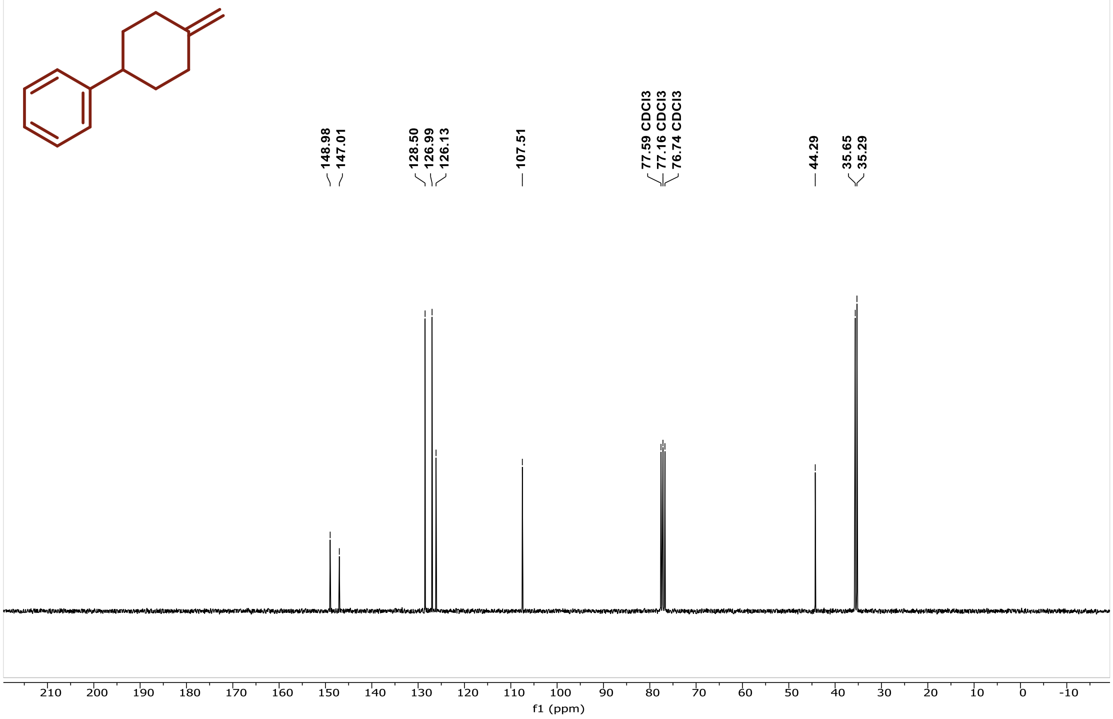

Compound **1p**: ^{1}H NMR (300 MHz, $CDCl_3$)

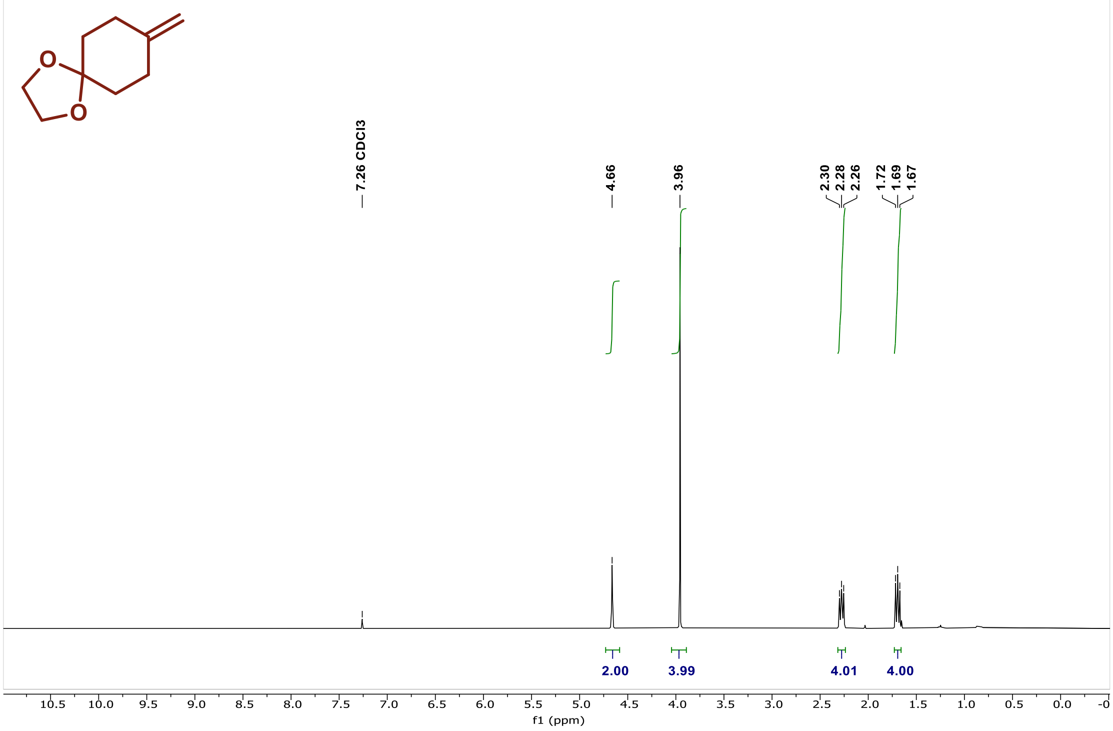

Compound **1p**: ^{13}C NMR (75 MHz, $CDCl_3$)

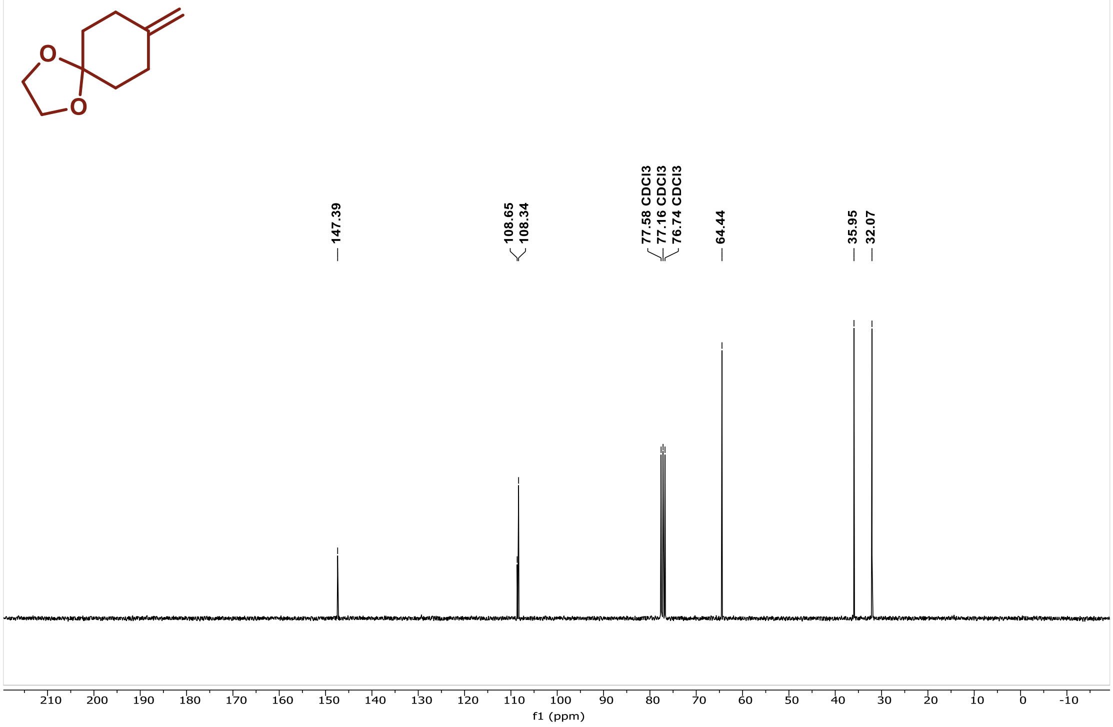

Compound **1q**: ^{1}H NMR (300 MHz, $CDCl_3$)

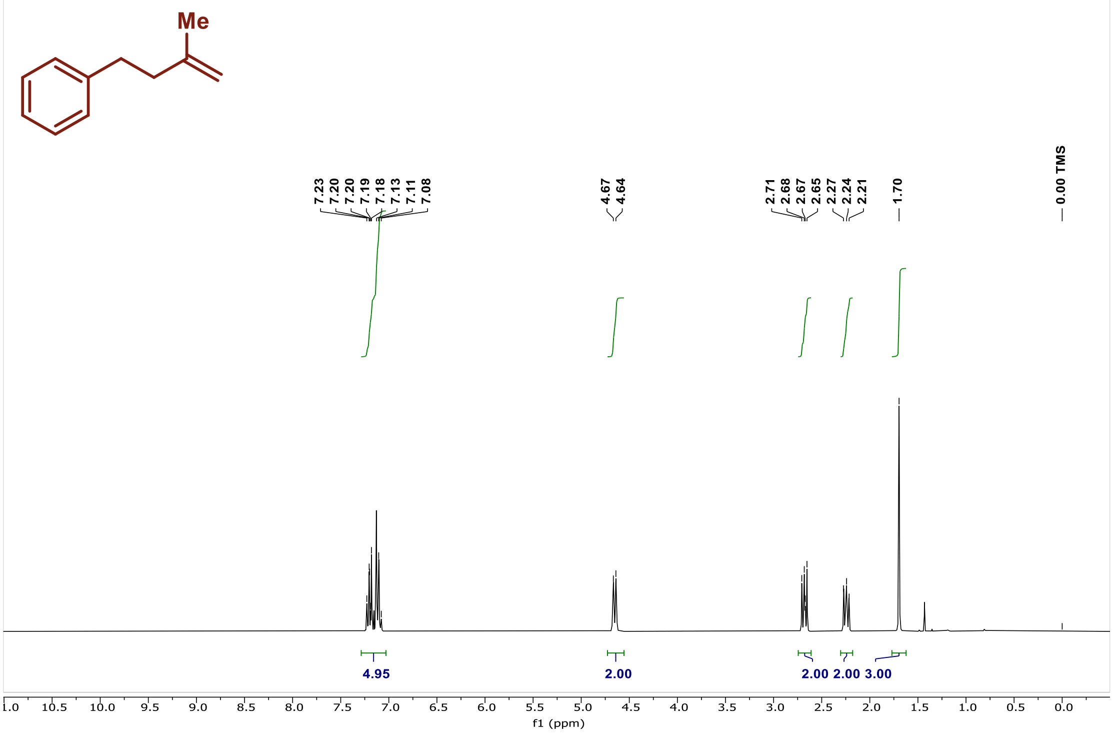

Compound **1q**: ^{13}C NMR (75 MHz, $CDCl_3$)

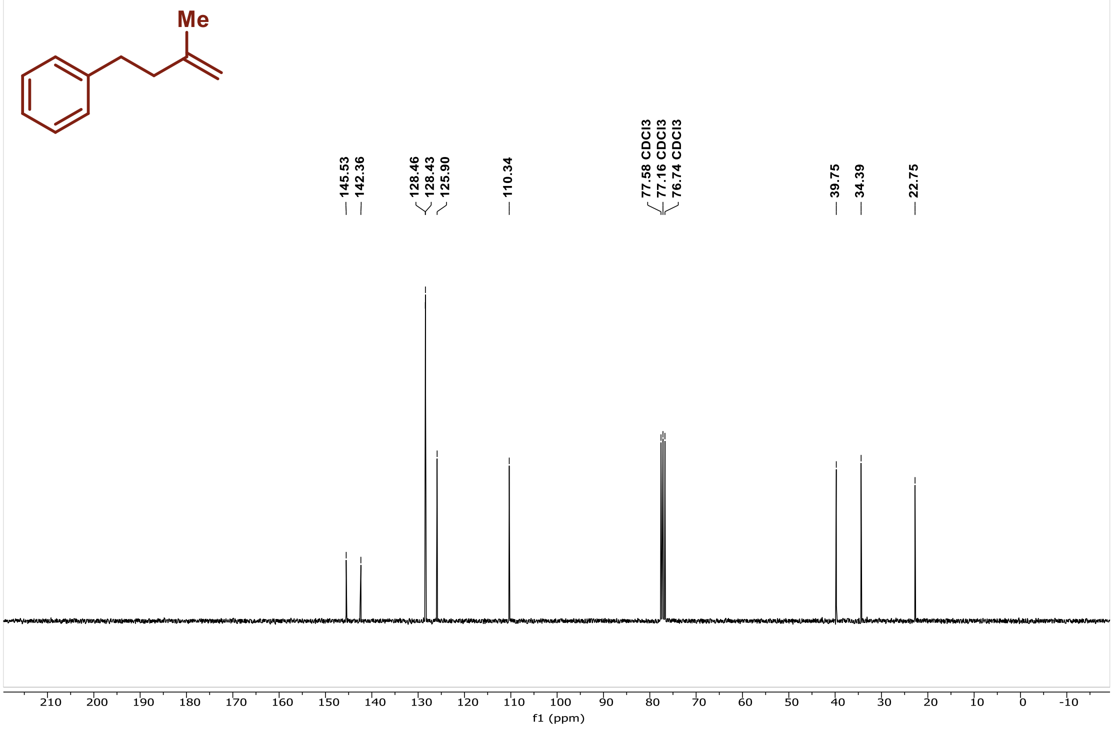

Compound **1r**: ^{1}H NMR (300 MHz, $CDCl_3$)

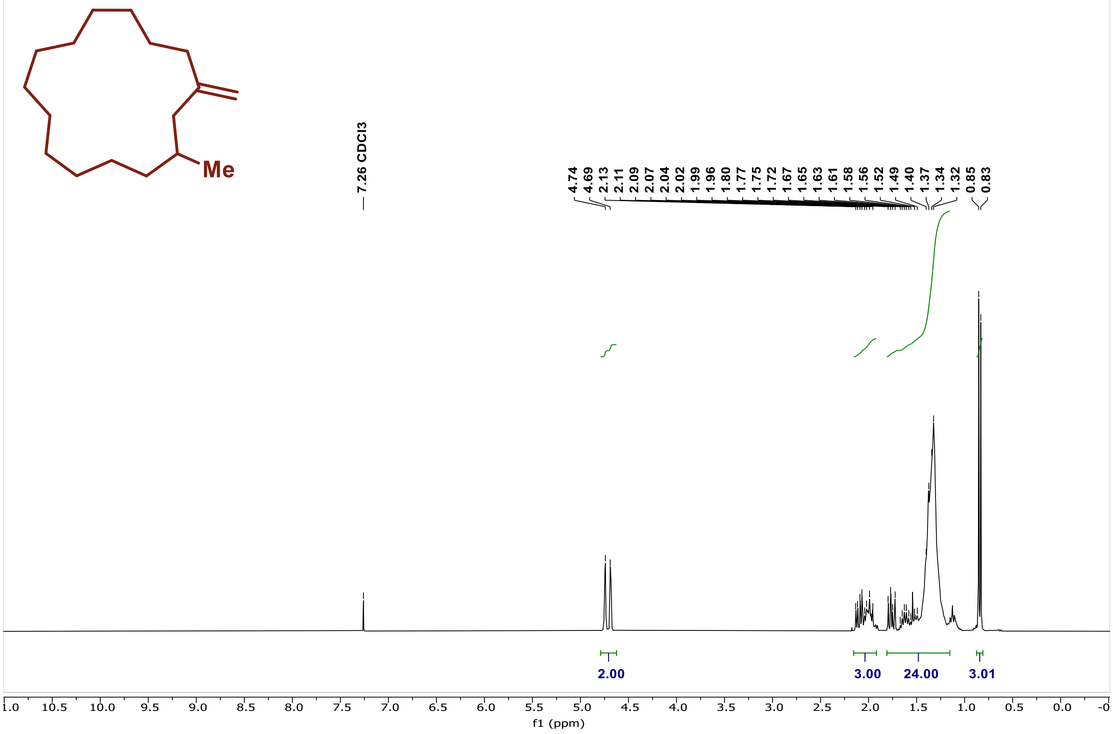

Compound **1r**: ^{13}C NMR (75 MHz, $CDCl_3$)

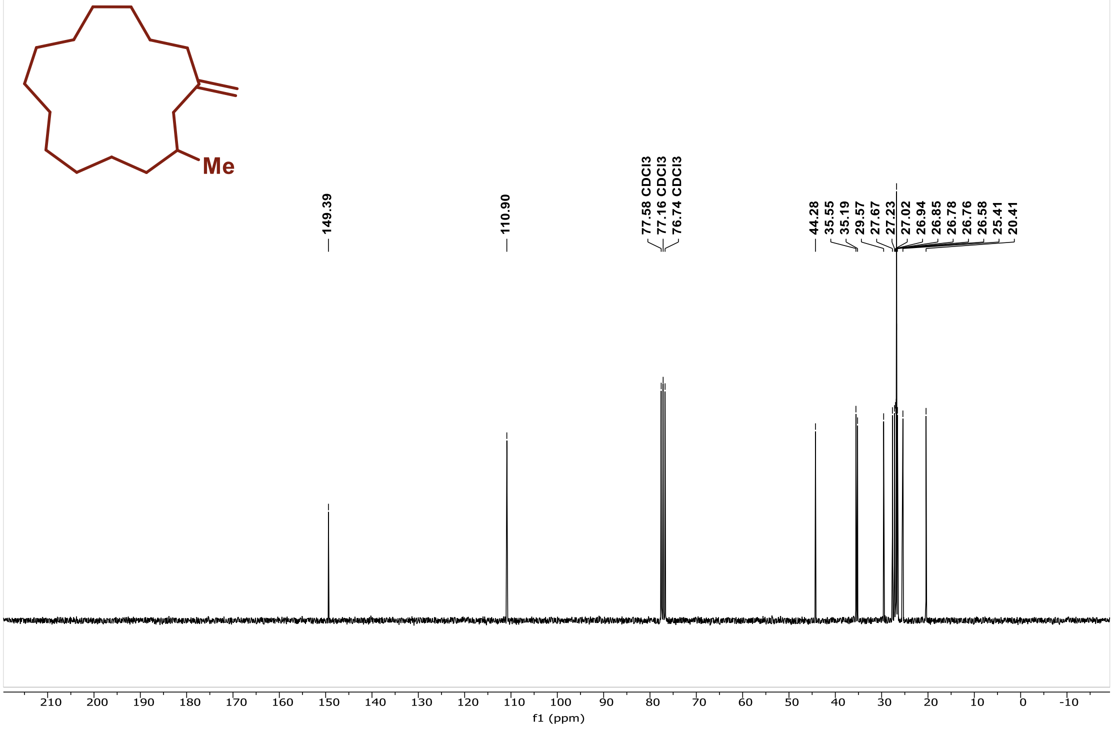

Compound **1s**: ^{1}H NMR (300 MHz, $CDCl_3$)

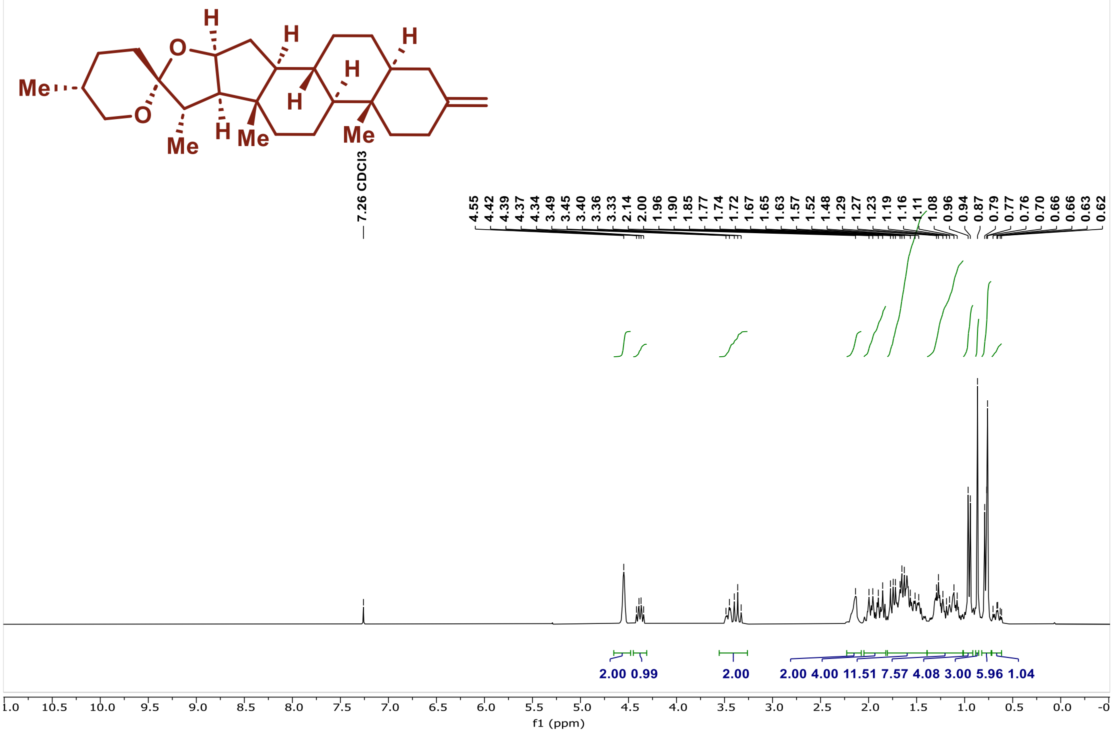

Compound **1s**: ^{13}C NMR (75 MHz, $CDCl_3$)

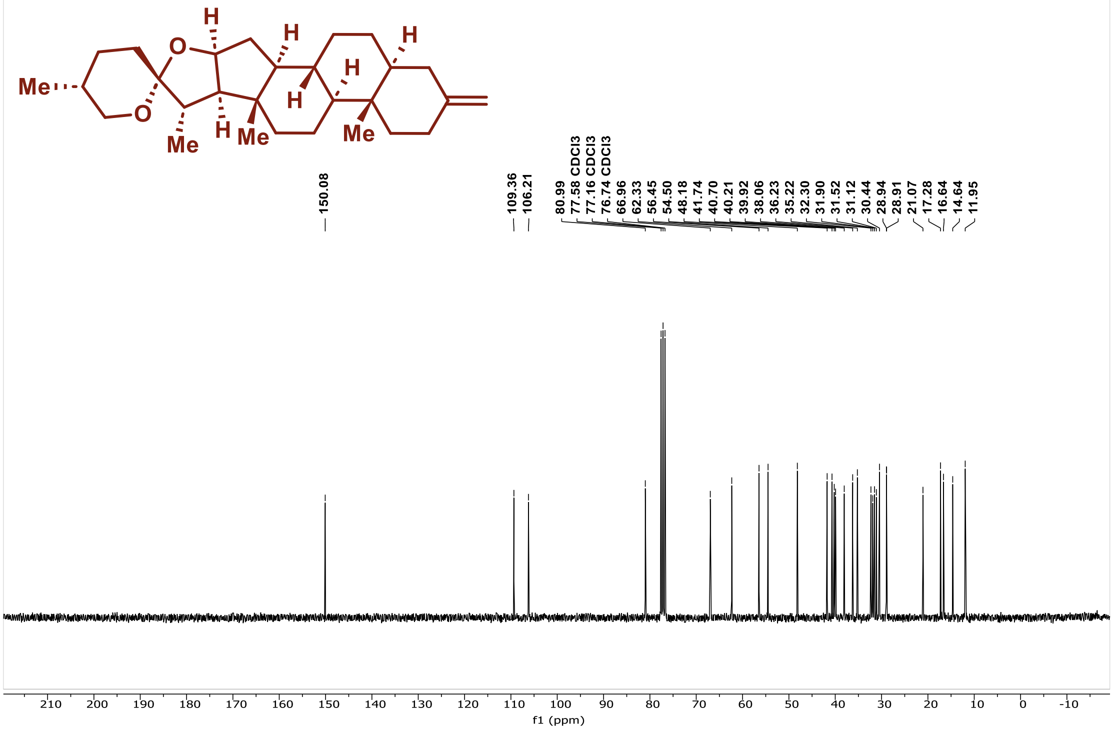

Compound **1s**: DEPT 135

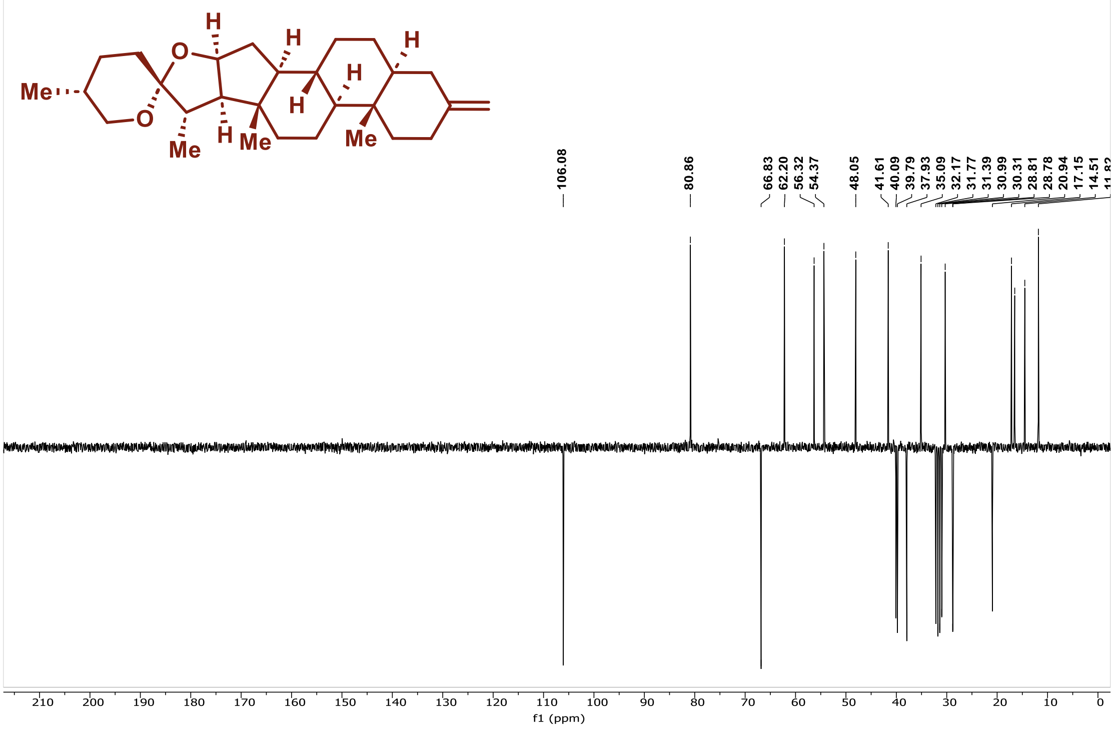

Compound **1s**: COSY

kappe_XYF_2-98.14.ser — 2D-cosy CDCl3 /opt/nmrdata allusers

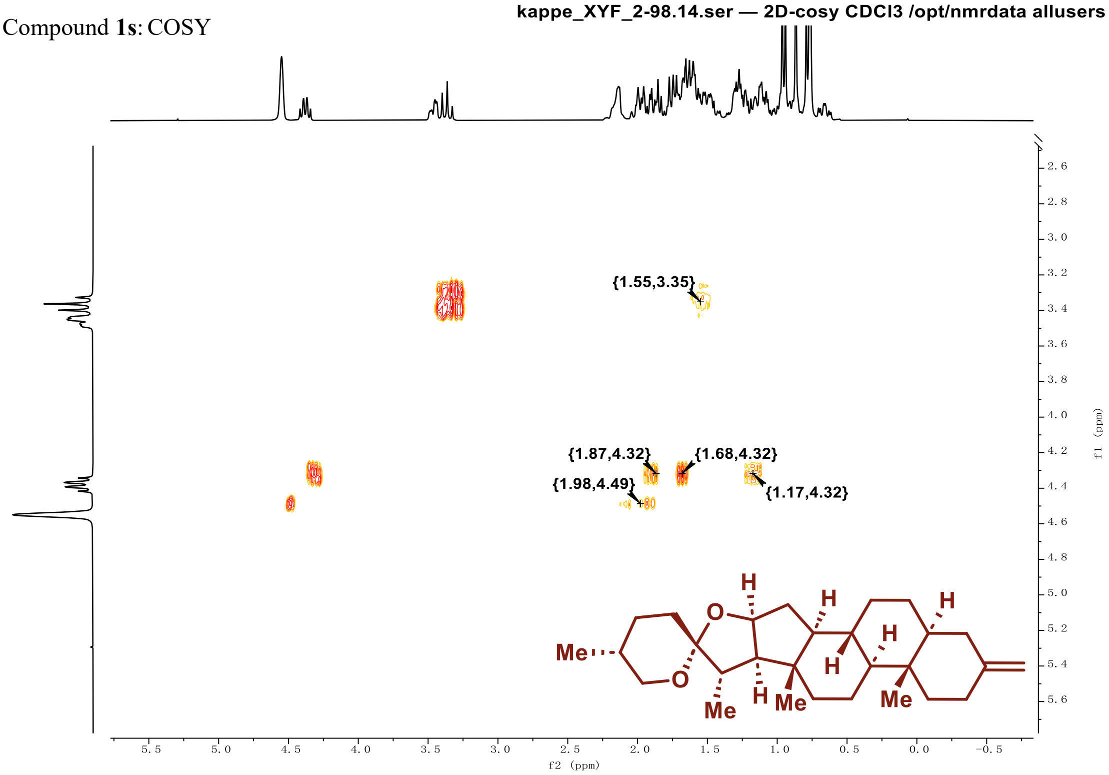

Compound **1s**: HSQC

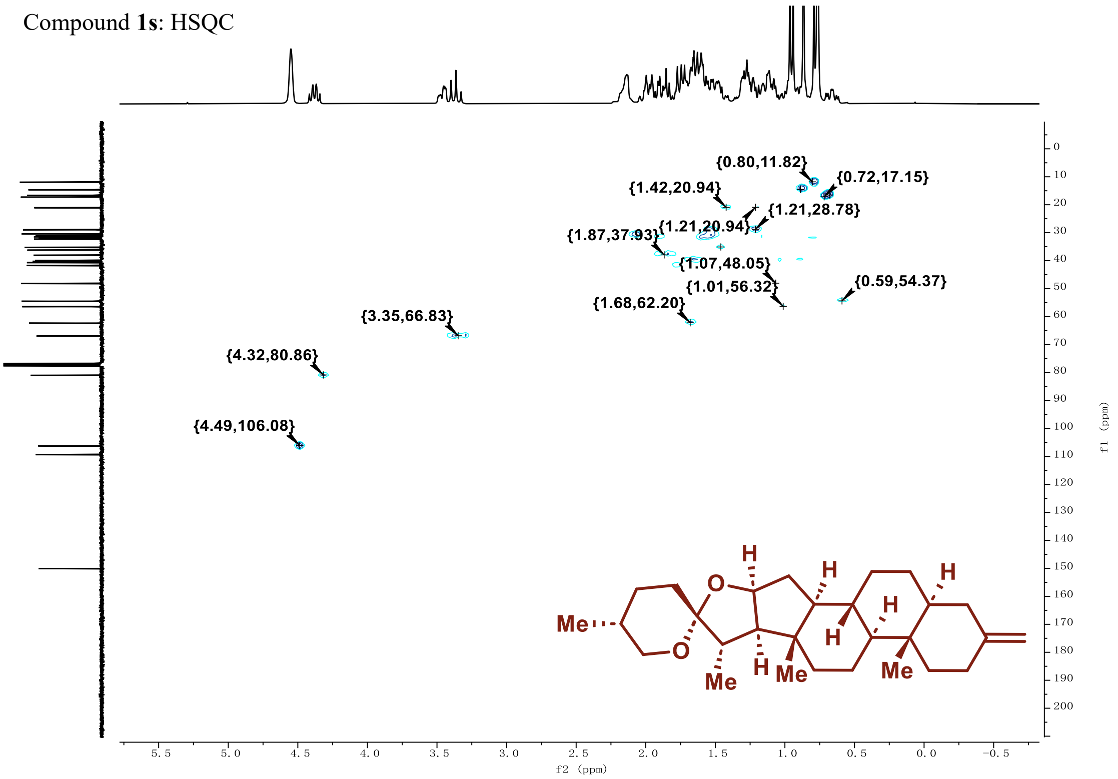

Compound **1s**: HMBC

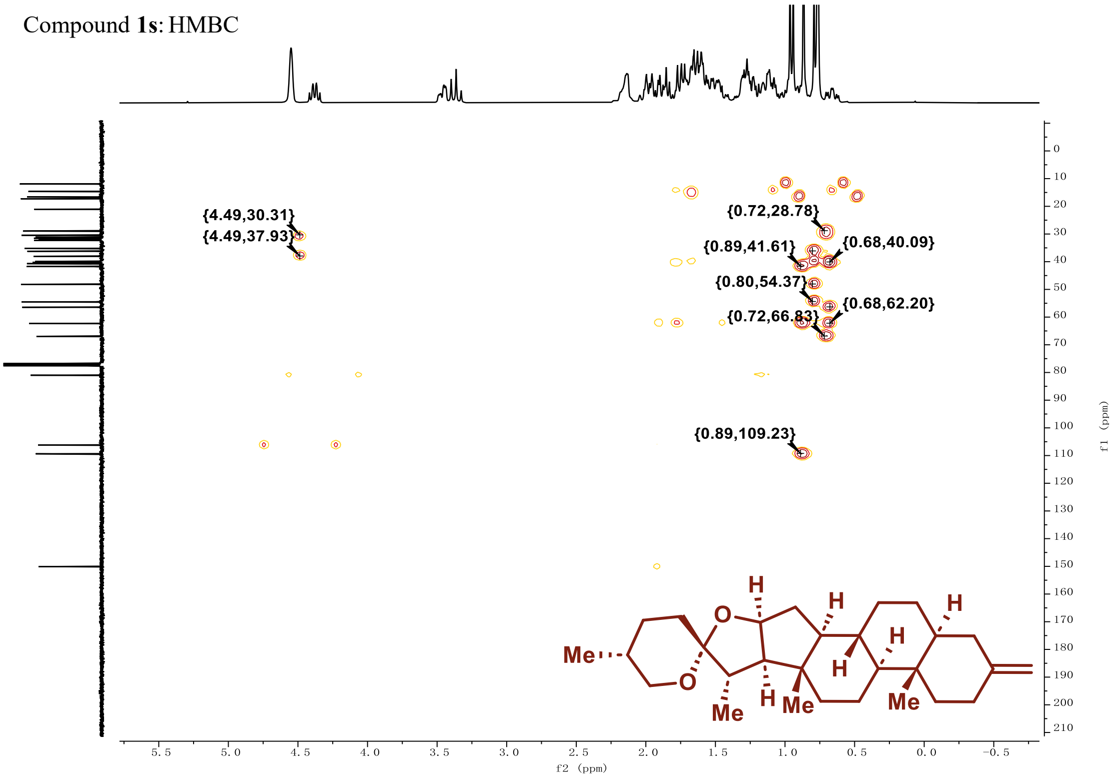

Compound **1s**: NOESY

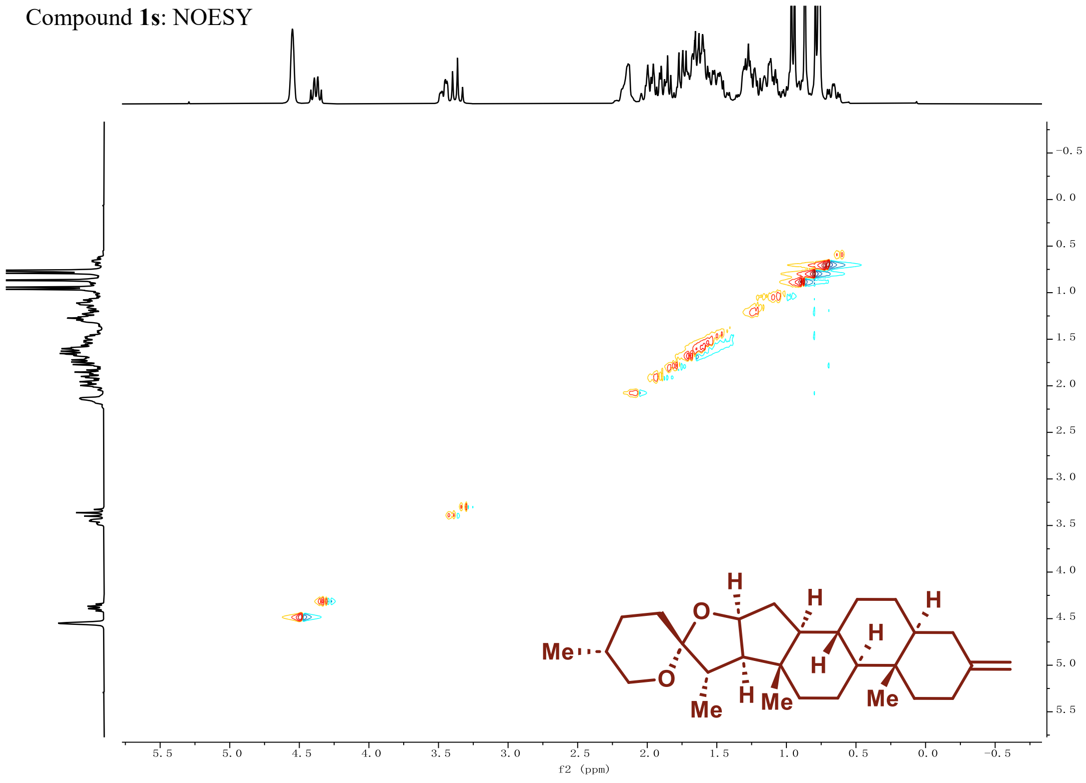

Compound **1t**: ^{1}H NMR (75 MHz, $CDCl_3$)

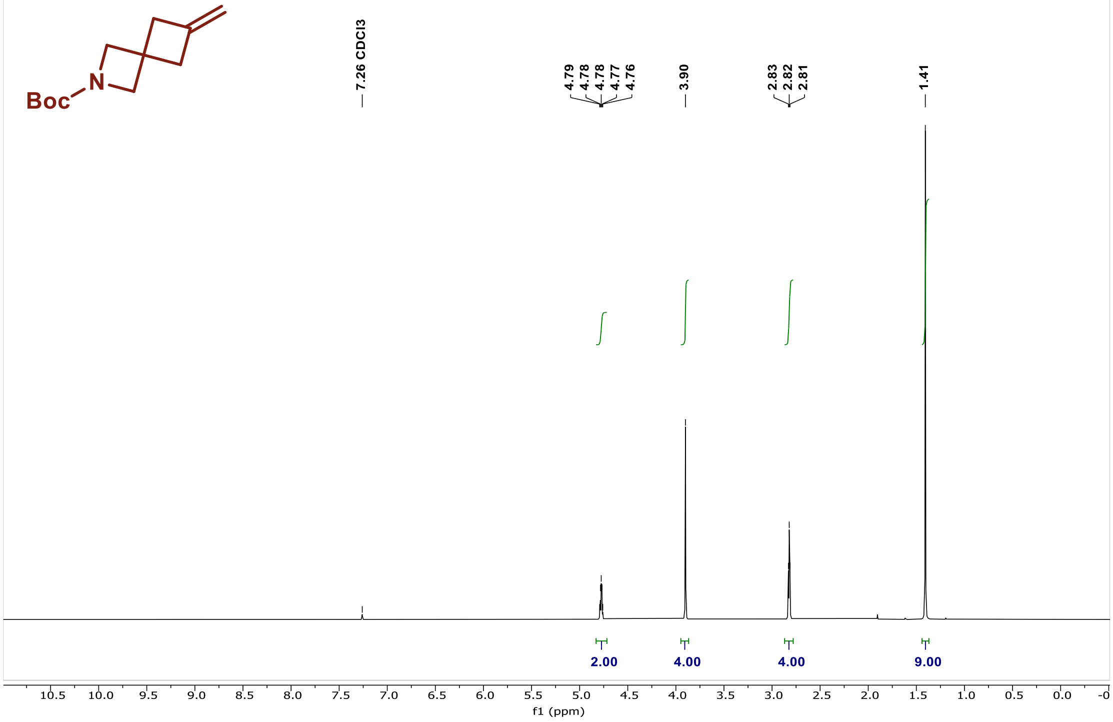

Compound **1t**: ^{13}C NMR (75 MHz, $CDCl_3$)

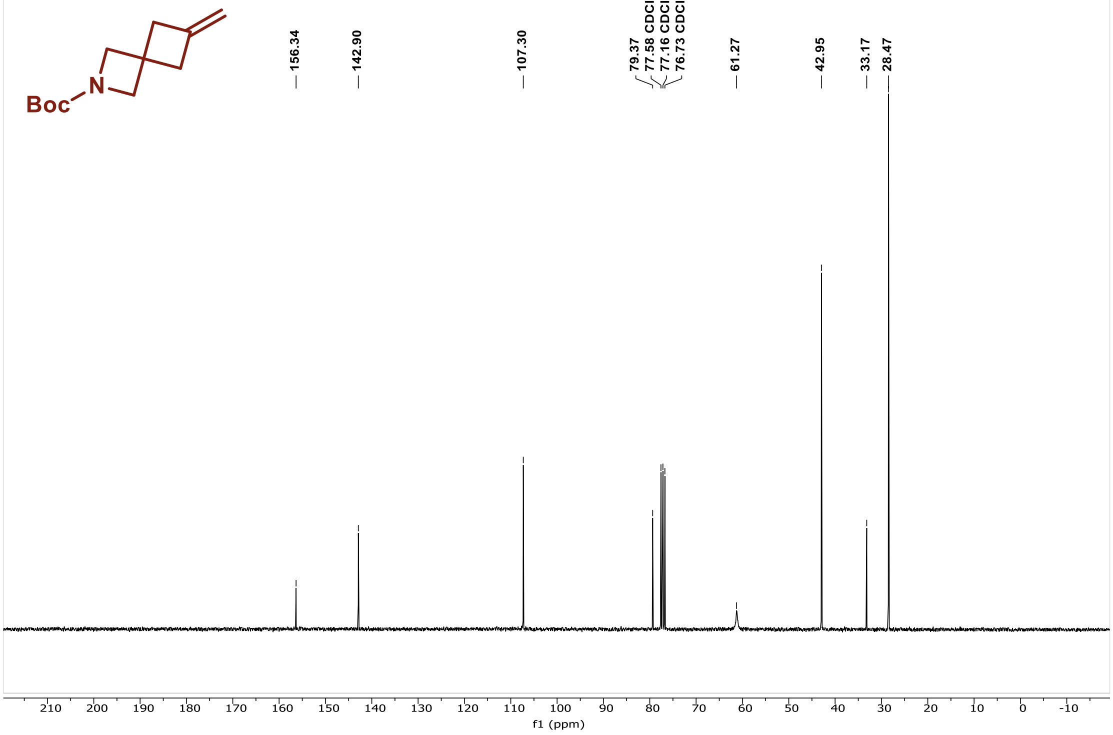

Compound **1w**: ^{1}H NMR (300 MHz, $CDCl_3$)

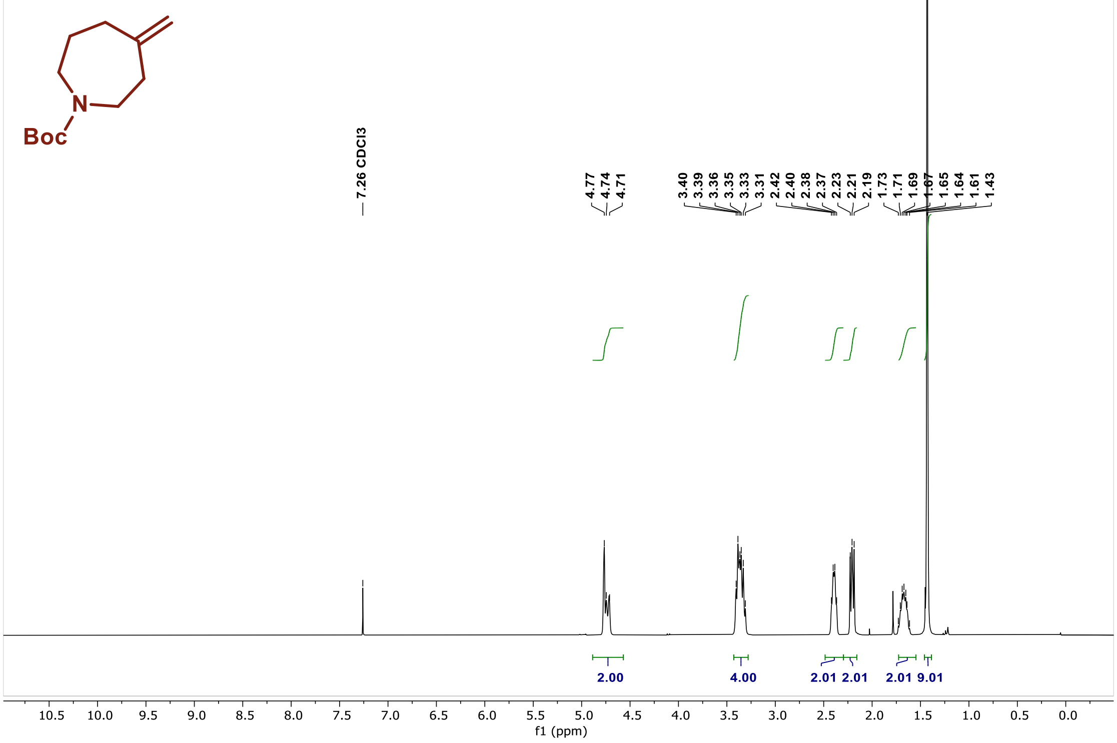

Compound **1w**: ^{13}C NMR (75 MHz, $CDCl_3$)

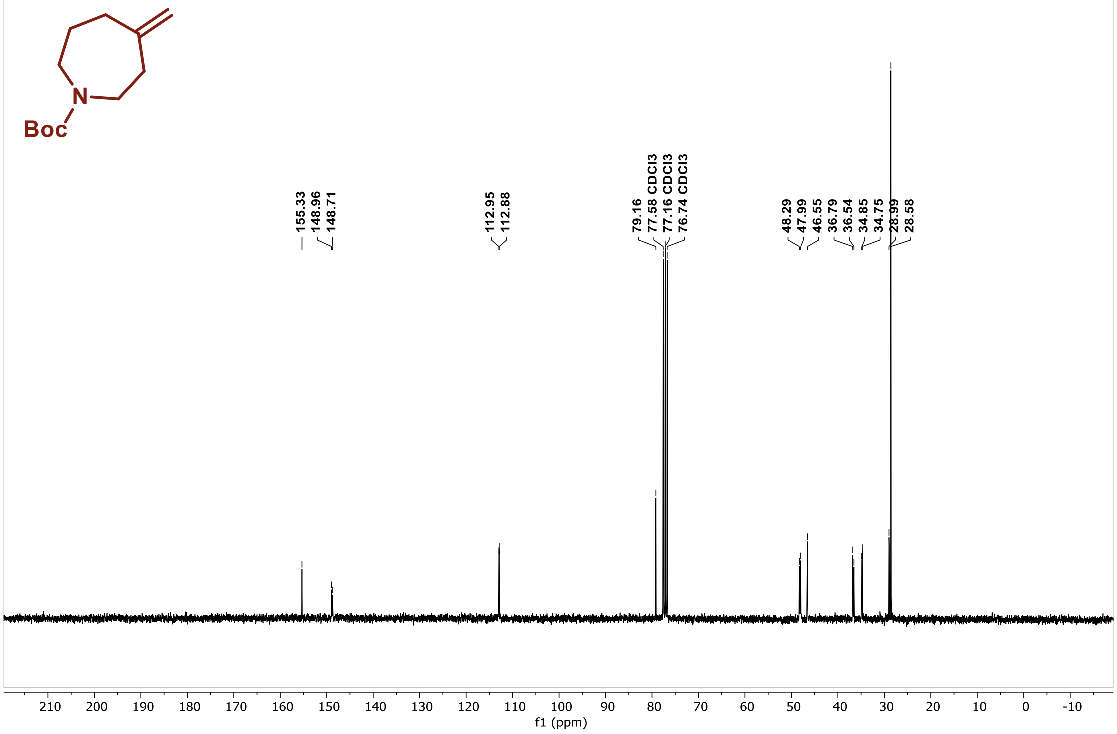

Compound **1x**: ^{1}H NMR (300 MHz, $CDCl_3$)

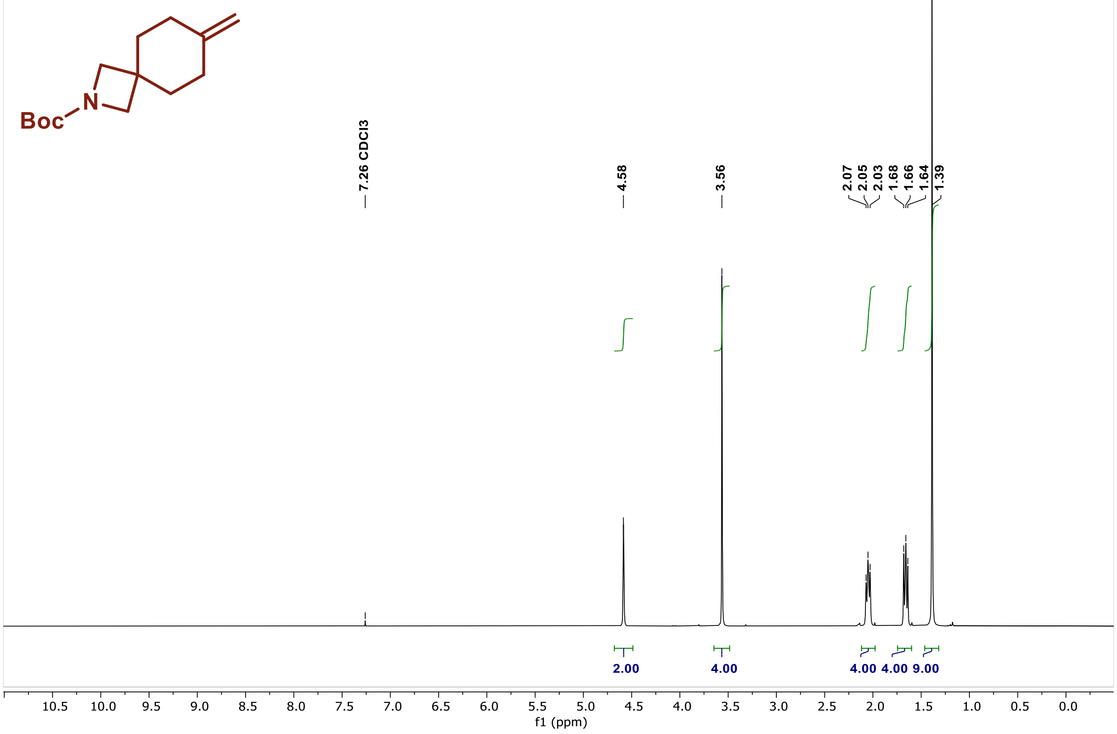

Compound **1x**: ^{13}C NMR (75 MHz, $CDCl_3$)

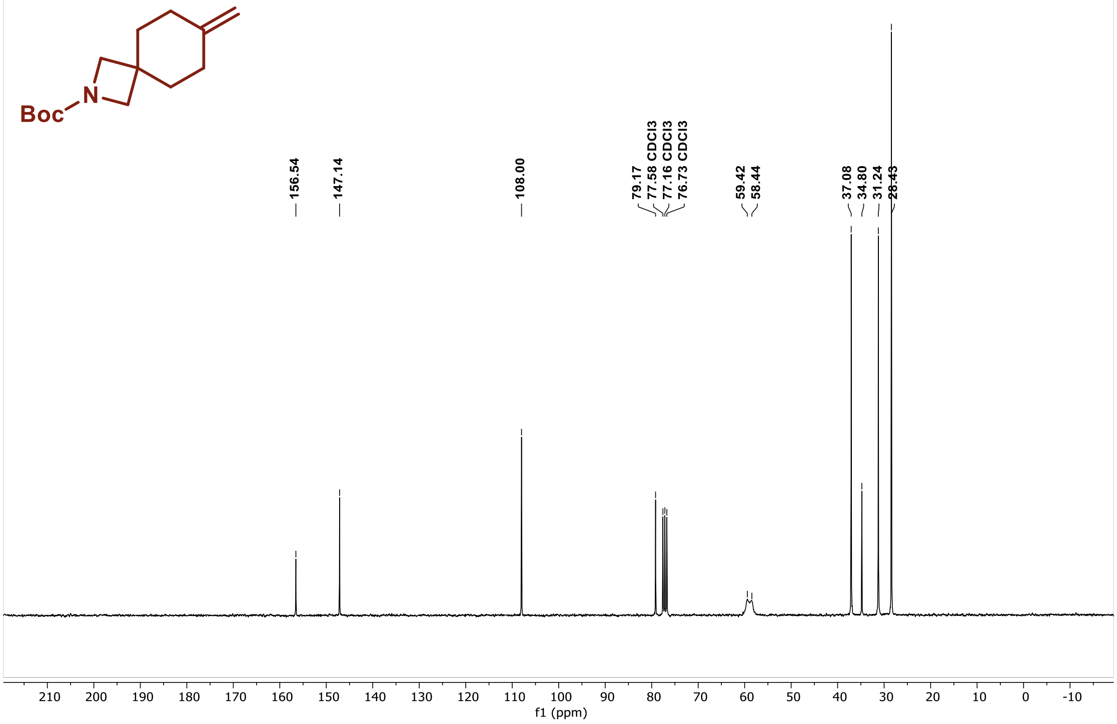

Compound **1y**: ^{1}H NMR (300 MHz, $CDCl_3$)

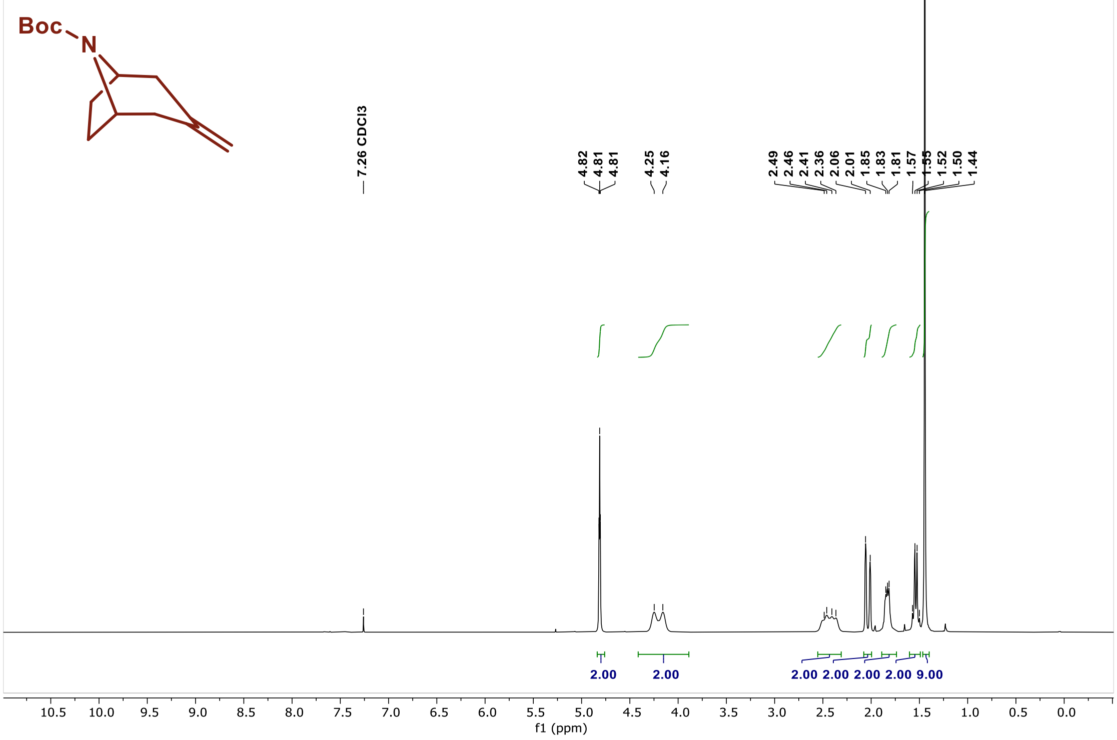

Compound **1y**: ^{13}C NMR (75 MHz, $CDCl_3$)

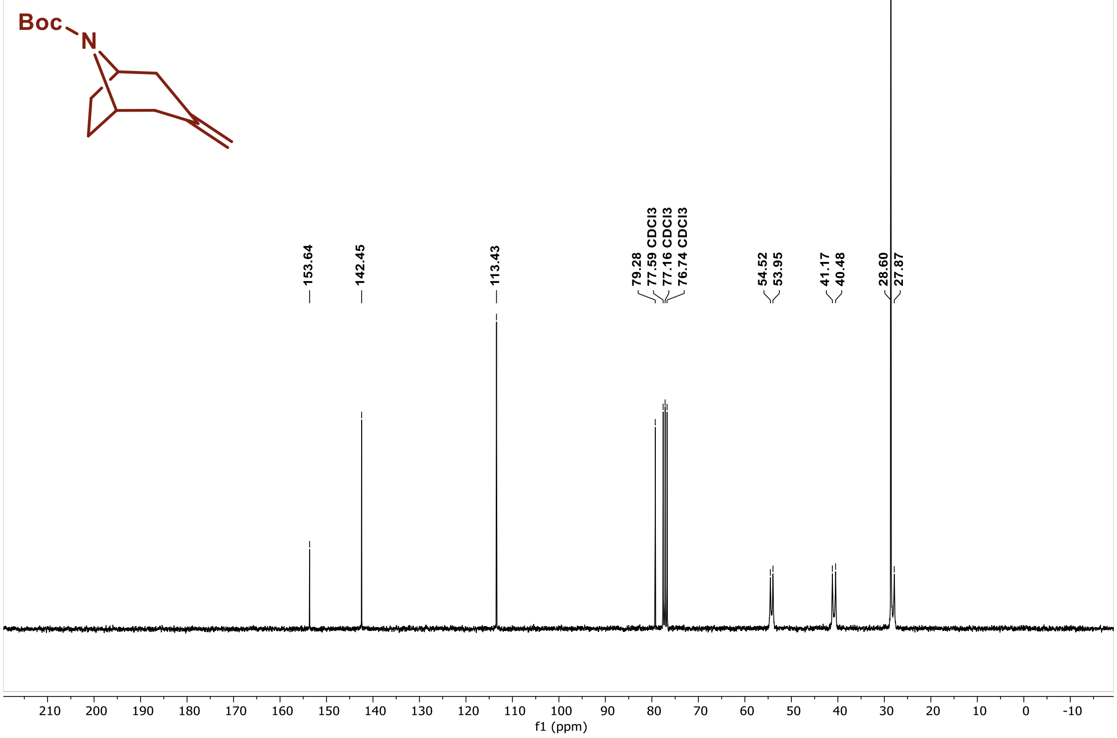

Compound **1aa**: ^{1}H NMR (300 MHz, $CDCl_3$)

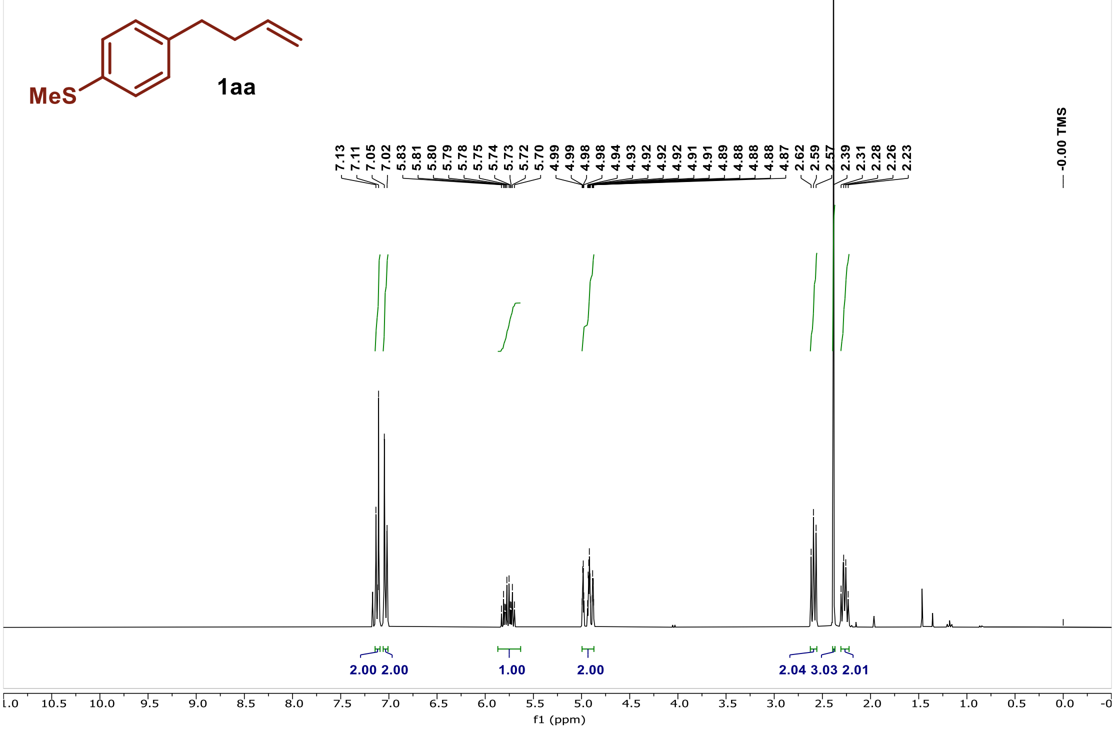

Compound **1aa**: ^{13}C NMR (75 MHz, $CDCl_3$)

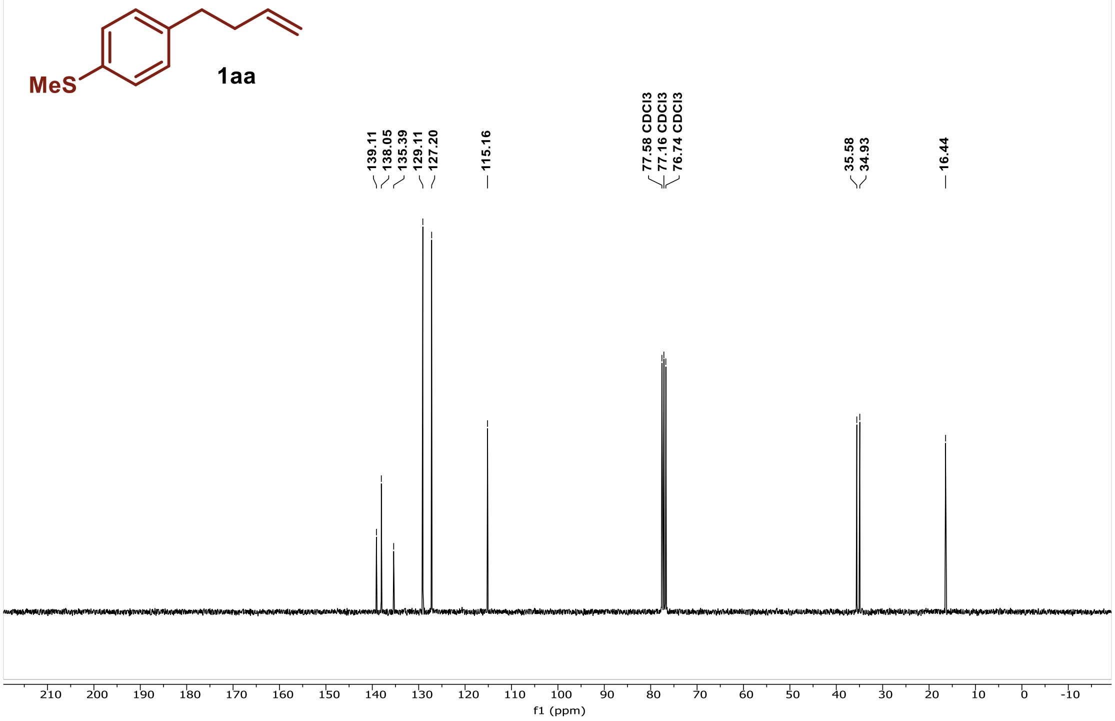

Compound **1ab**: ^{1}H NMR (300 MHz, $CDCl_3$)

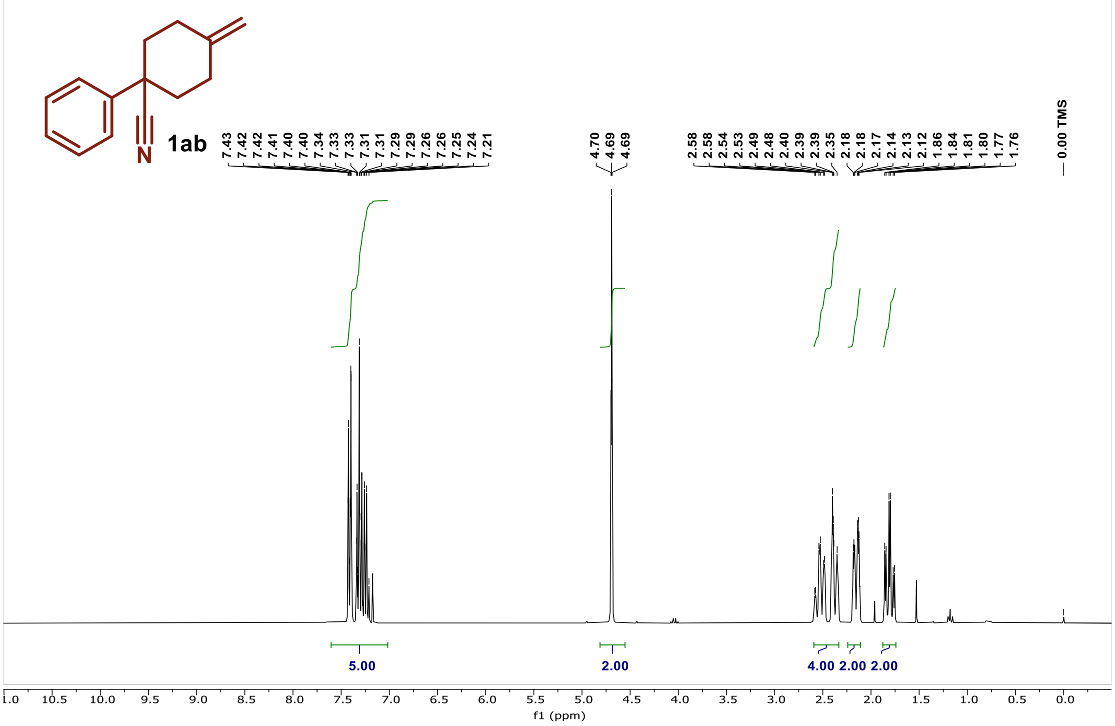

Compound **1ab**: ^{13}C NMR (75 MHz, $CDCl_3$)

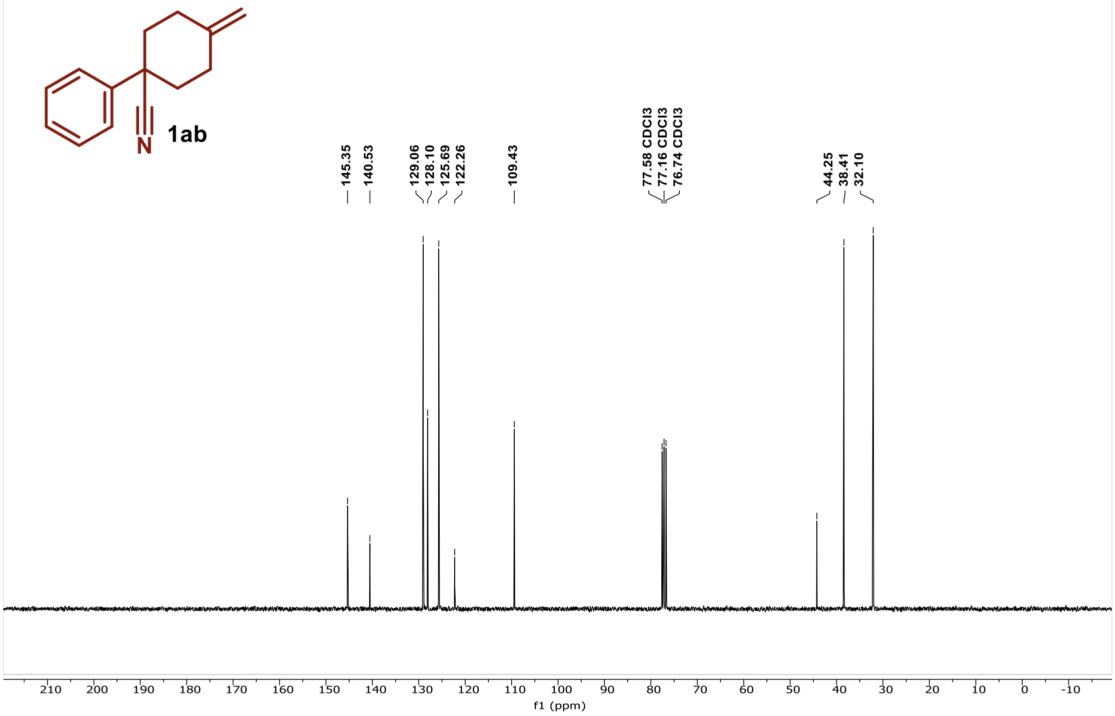

Aldehyde product

Compound **2a**: ^{1}H NMR (300 MHz, $CDCl_3$)

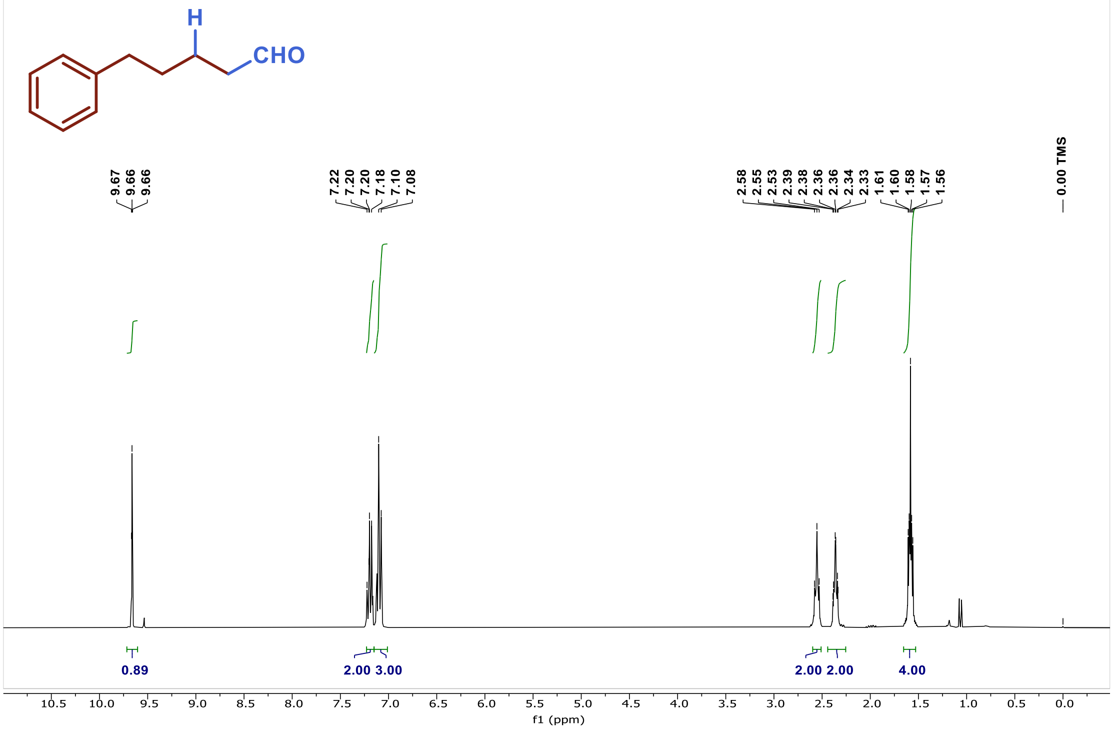

Compound **2a**: ^{13}C NMR (75 MHz, $CDCl_3$)

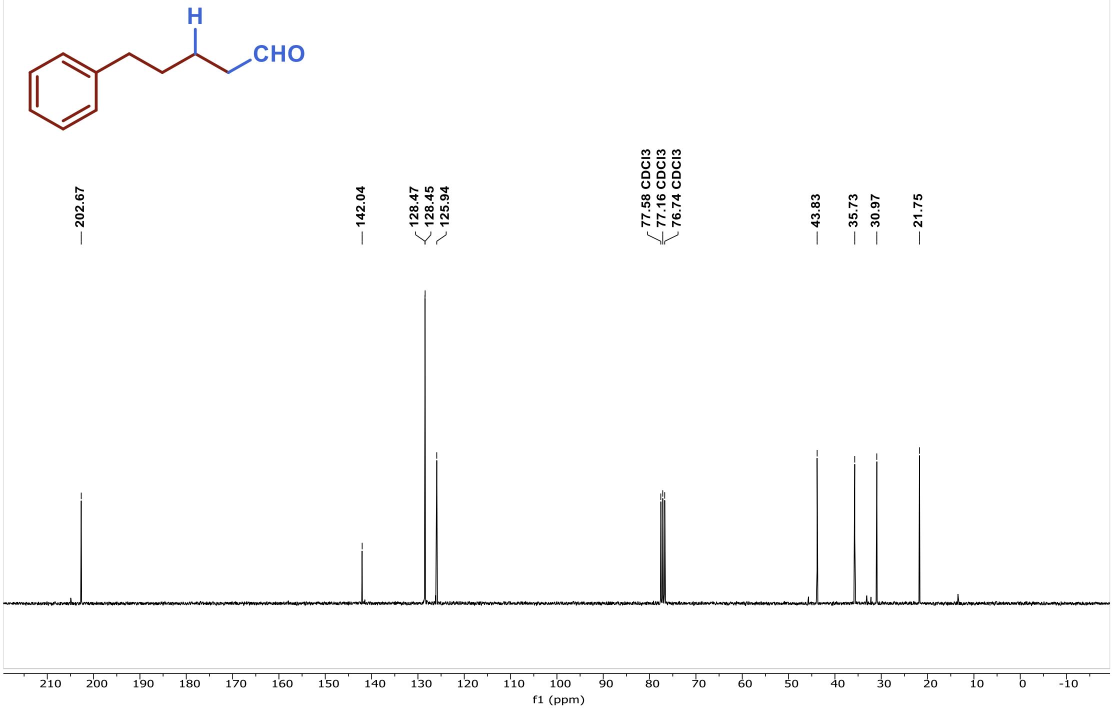

Compound **2b**: ^{1}H NMR (300 MHz, $CDCl_3$)

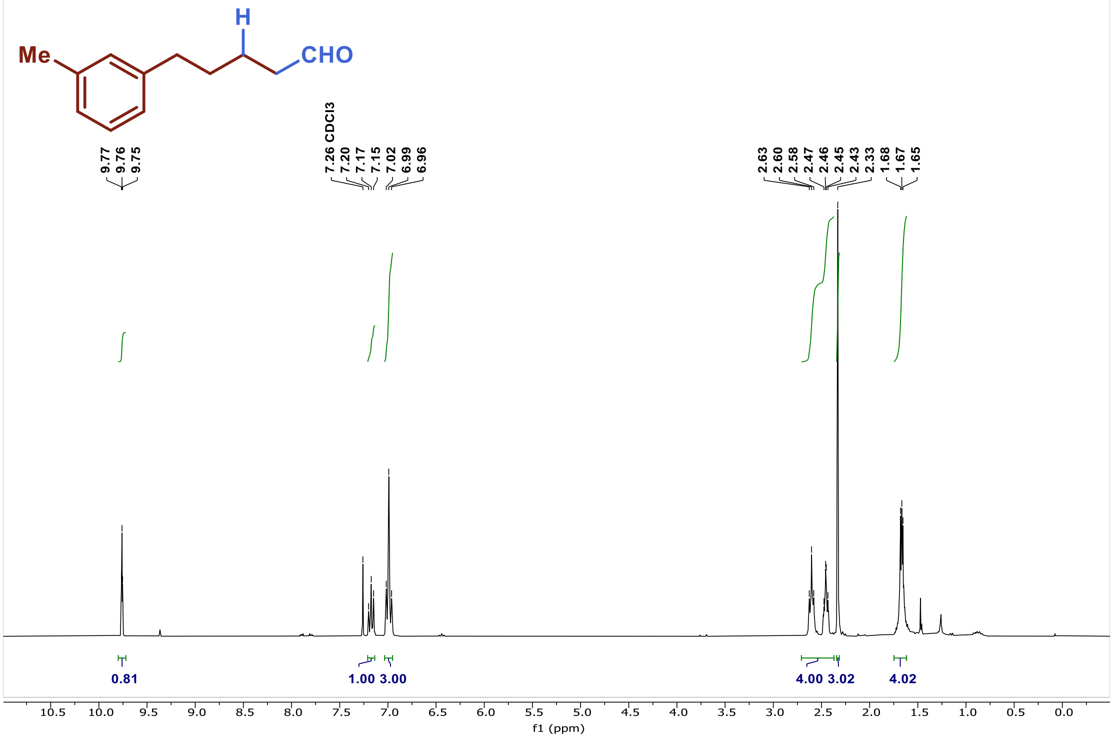

Compound **2b**: ^{13}C NMR (75 MHz, $CDCl_3$)

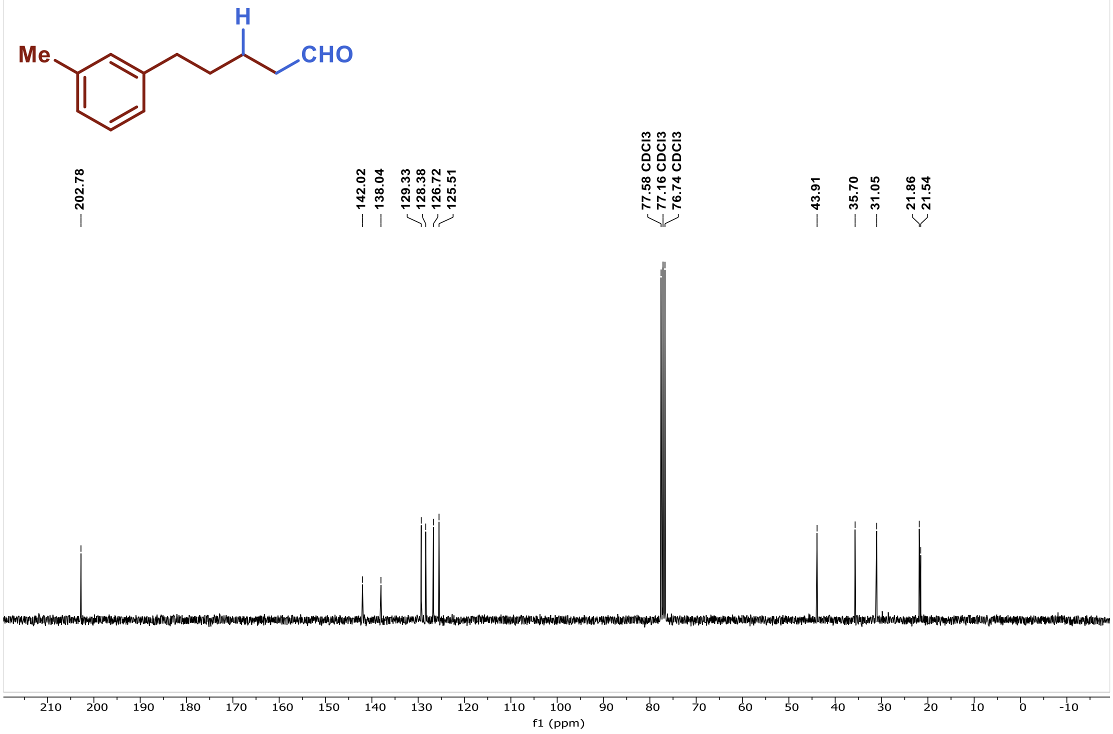

Compound **2c**: ^{1}H NMR (300 MHz, $CDCl_3$)

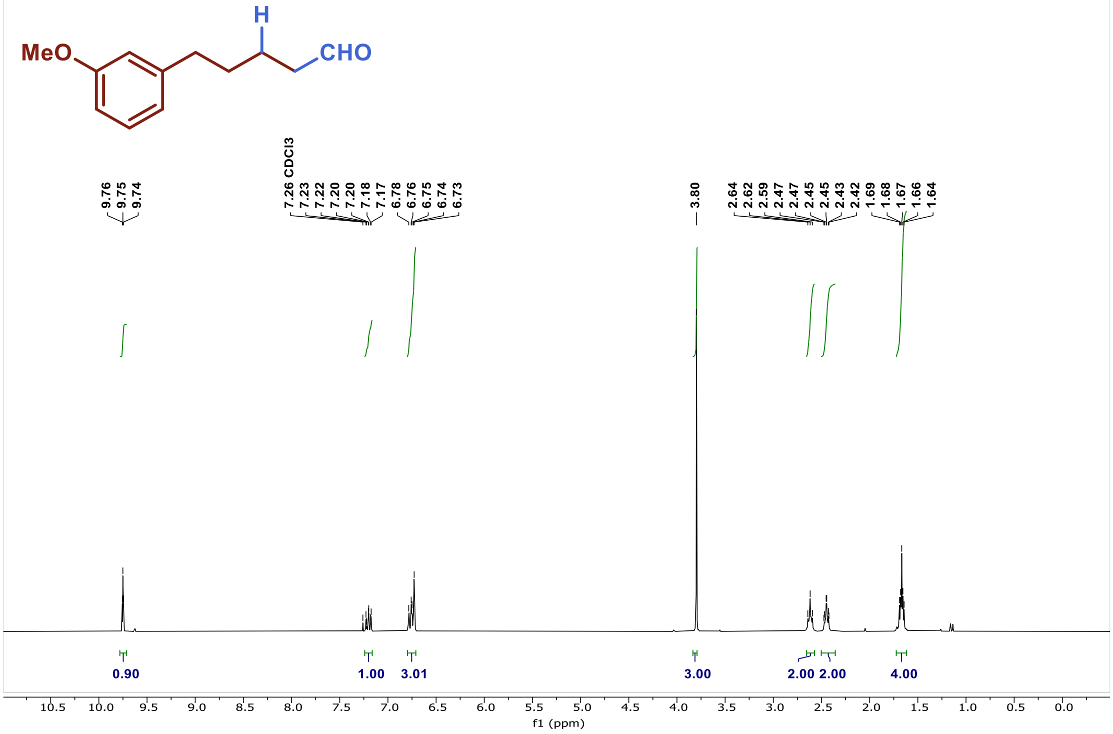

Compound **2c**: ^{13}C NMR (75 MHz, $CDCl_3$)

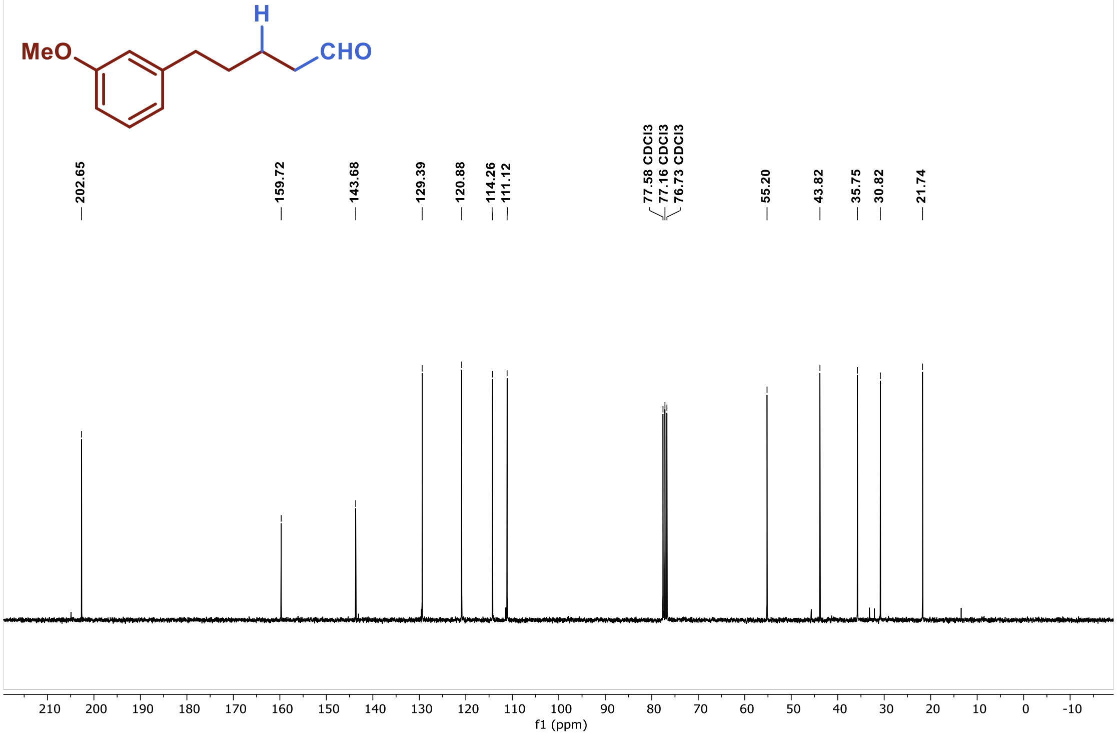

Compound **2d**: ^{1}H NMR (300 MHz, $CDCl_3$)

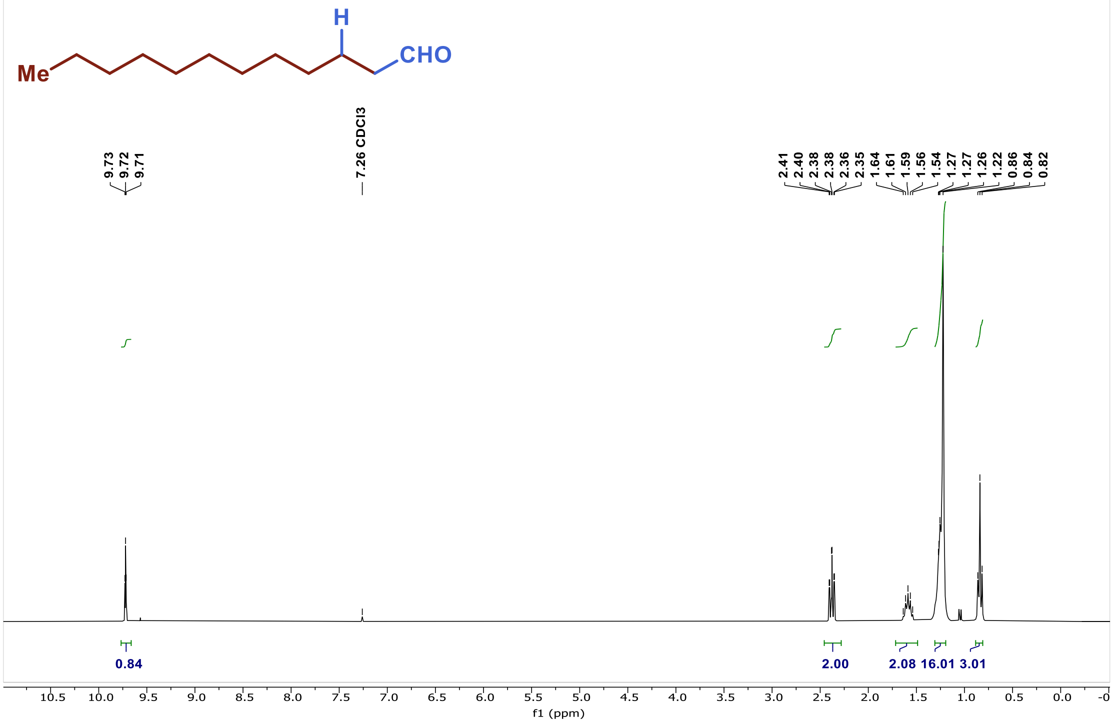

Compound **2d**: ^{13}C NMR (75 MHz, $CDCl_3$)

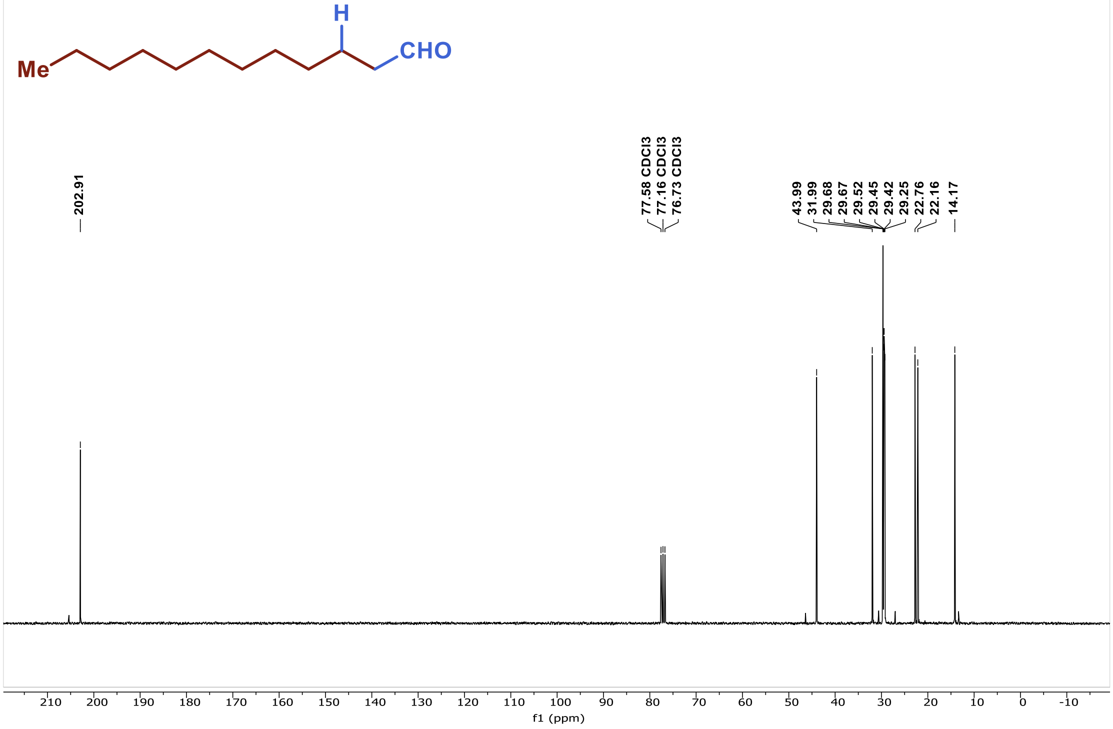

Compound **2e**: ^{1}H NMR (300 MHz, $CDCl_3$)

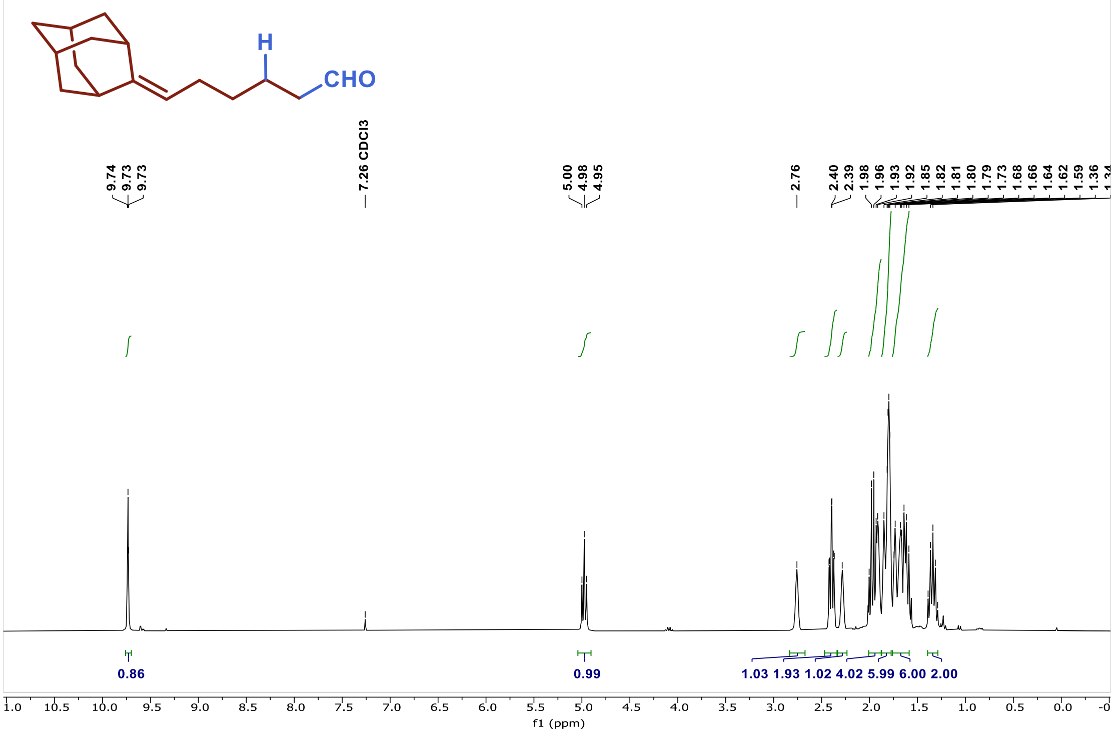

Compound **2e**: ^{13}C NMR (75 MHz, $CDCl_3$)

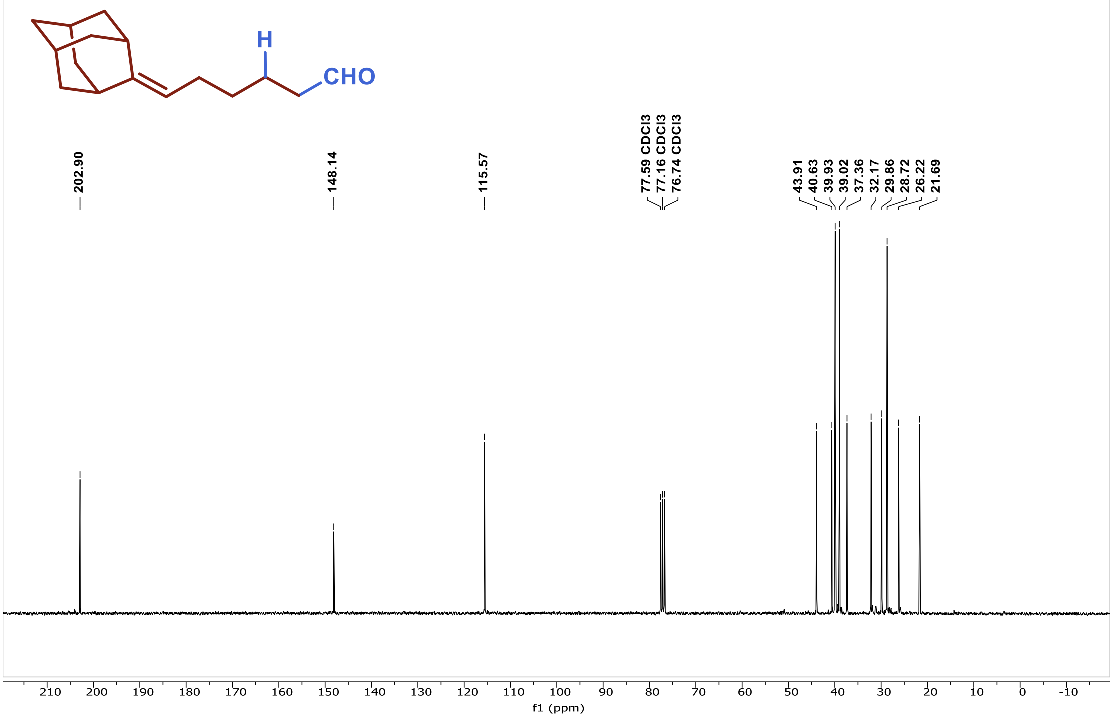

Compound **2f**: ^{1}H NMR (300 MHz, $CDCl_3$)

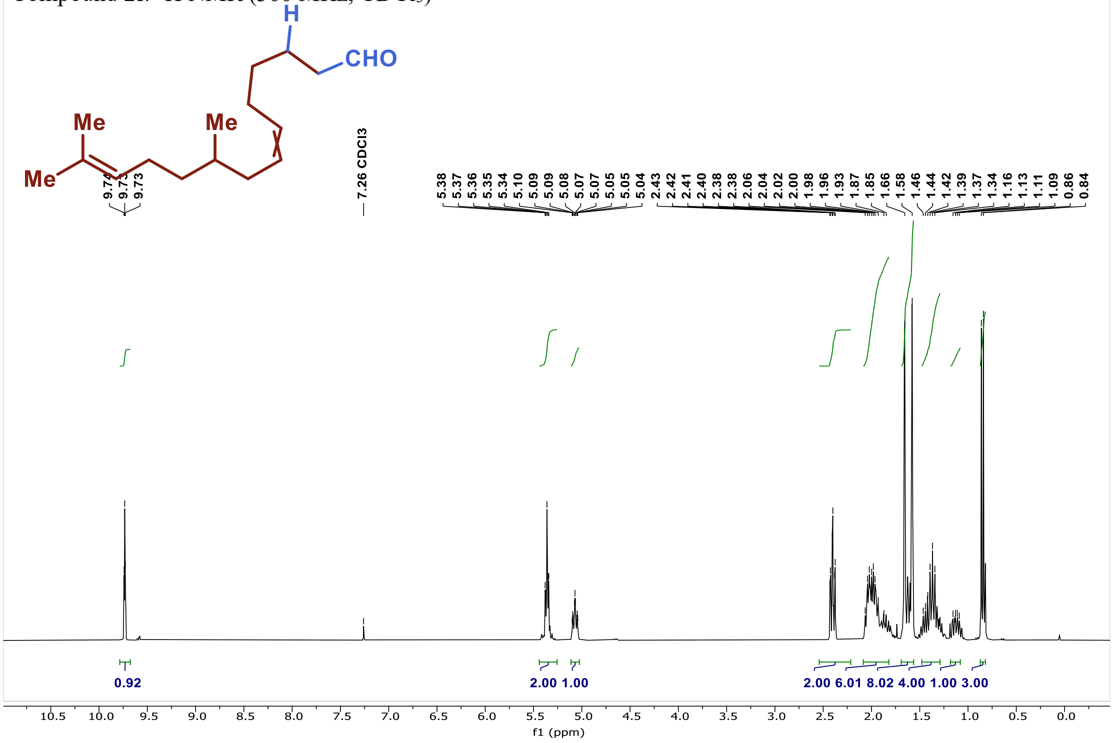

Compound **2f**: ^{13}C NMR (75 MHz, $CDCl_3$)

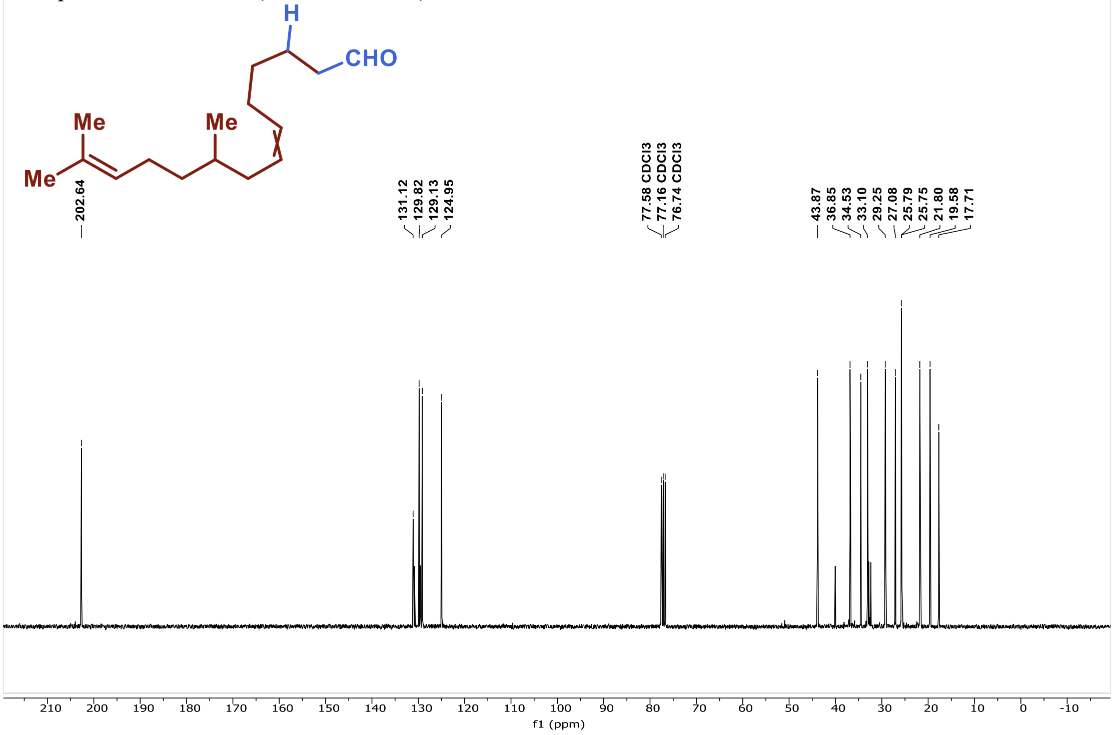

Compound **2g**: ¹H NMR (300 MHz, $CDCl_3$)

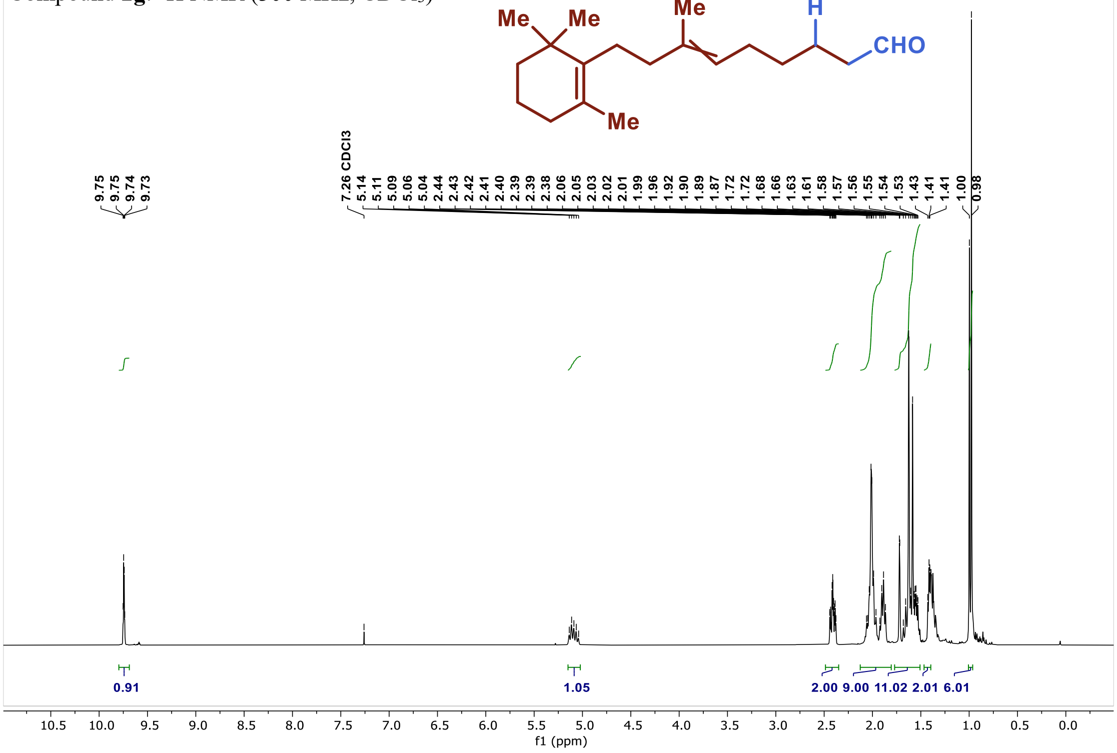

Compound **2g**: ¹³C NMR (75 MHz, $CDCl_3$)

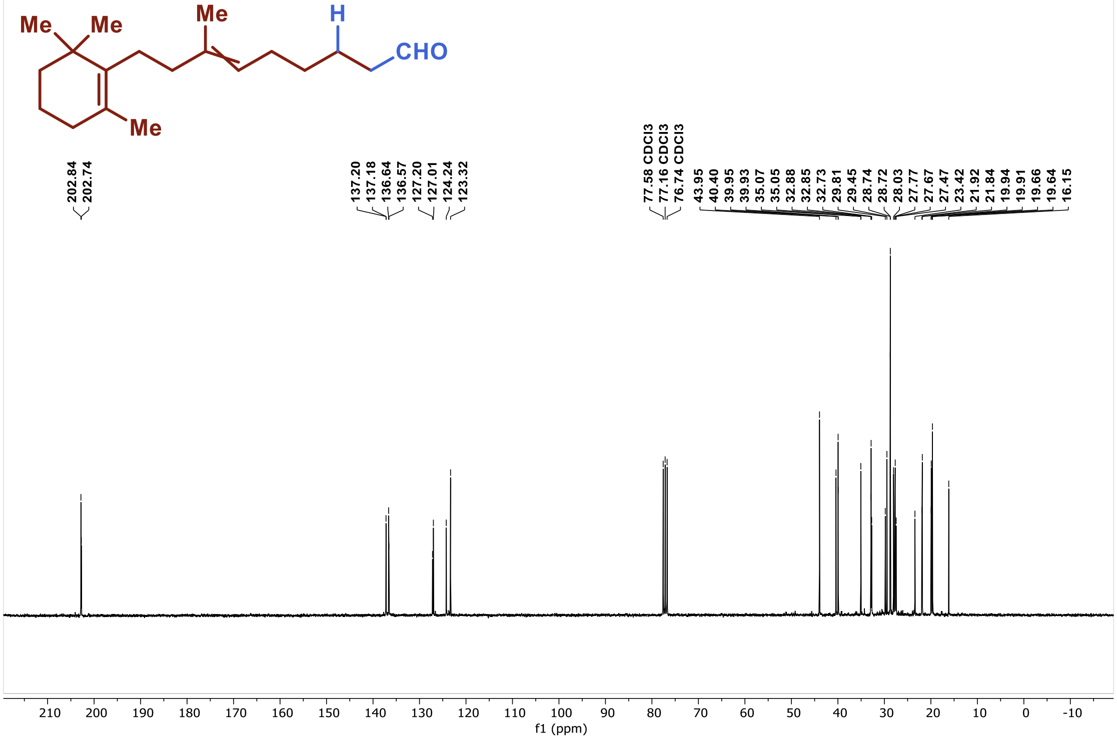

Compound **2h**: ^{1}H NMR (300 MHz, $CDCl_3$)

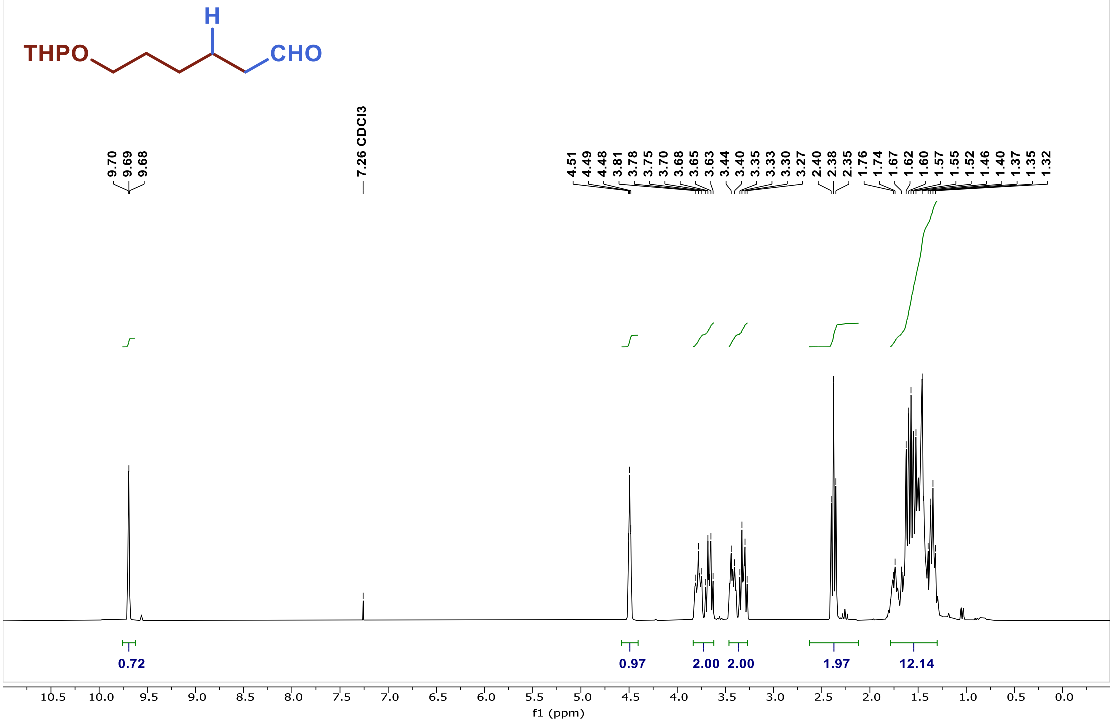

Compound **2h**: ^{13}C NMR (75 MHz, $CDCl_3$)

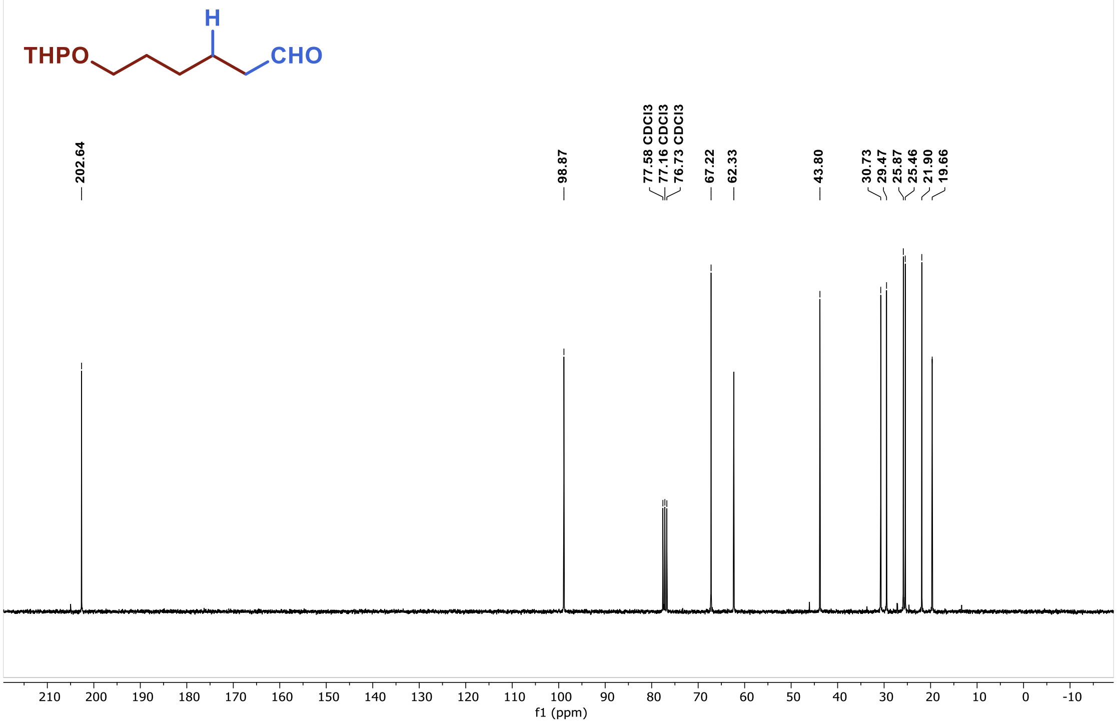

Compound **2i**: ^{1}H NMR (300 MHz, $CDCl_3$)

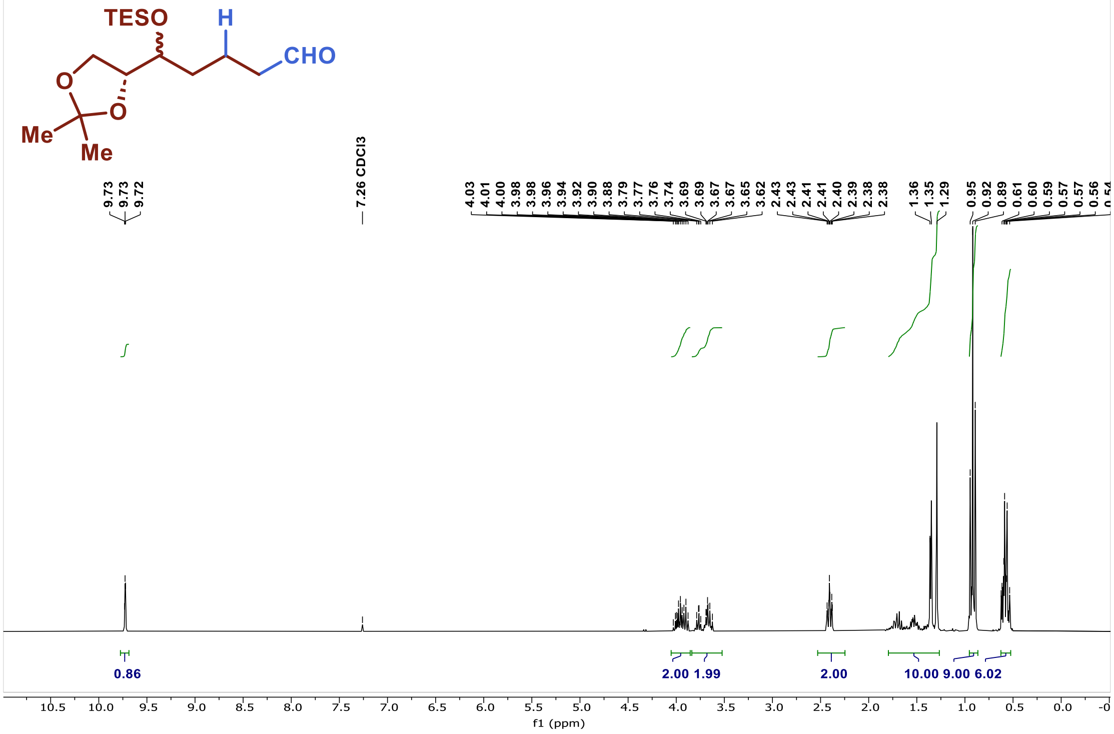

Compound **2i**: ^{13}C NMR (75 MHz, $CDCl_3$)

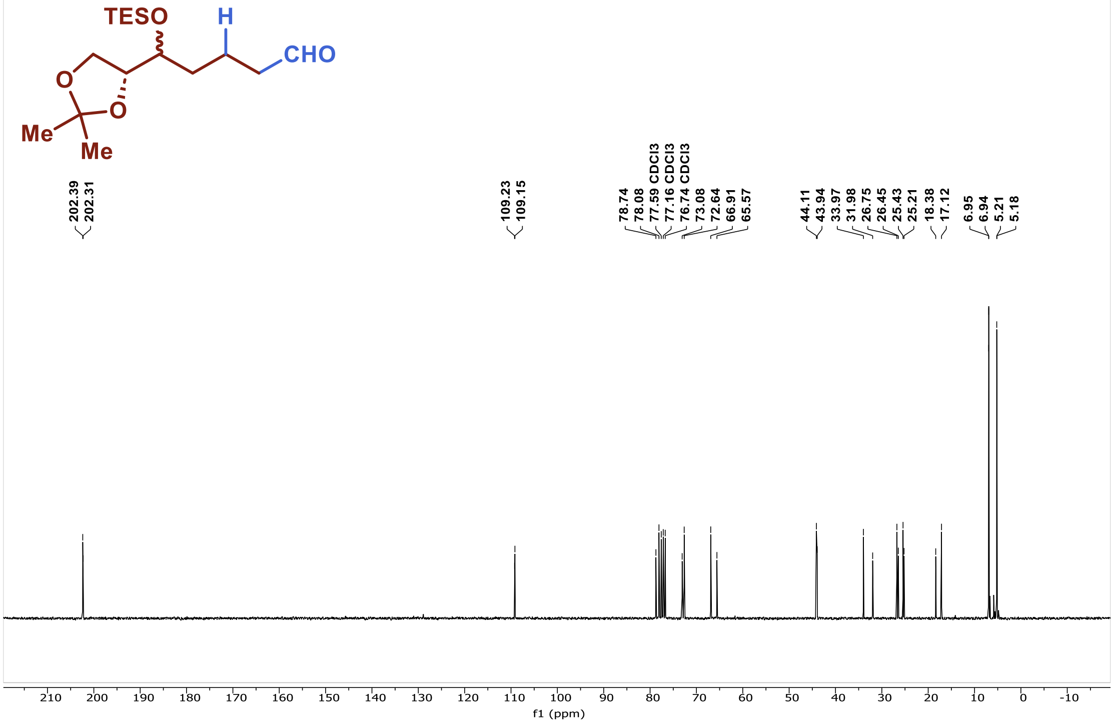

Compound **2j**: ^{1}H NMR (300 MHz, $CDCl_3$)

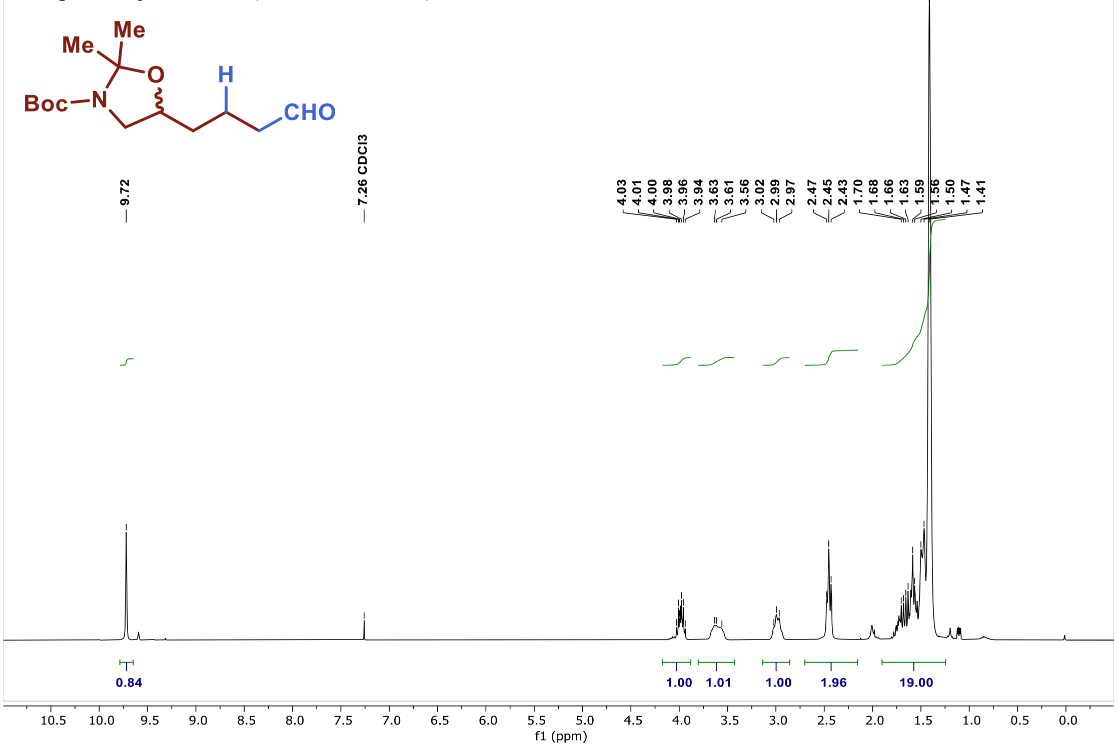

Compound **2j**: ^{13}C NMR (75 MHz, $CDCl_3$)

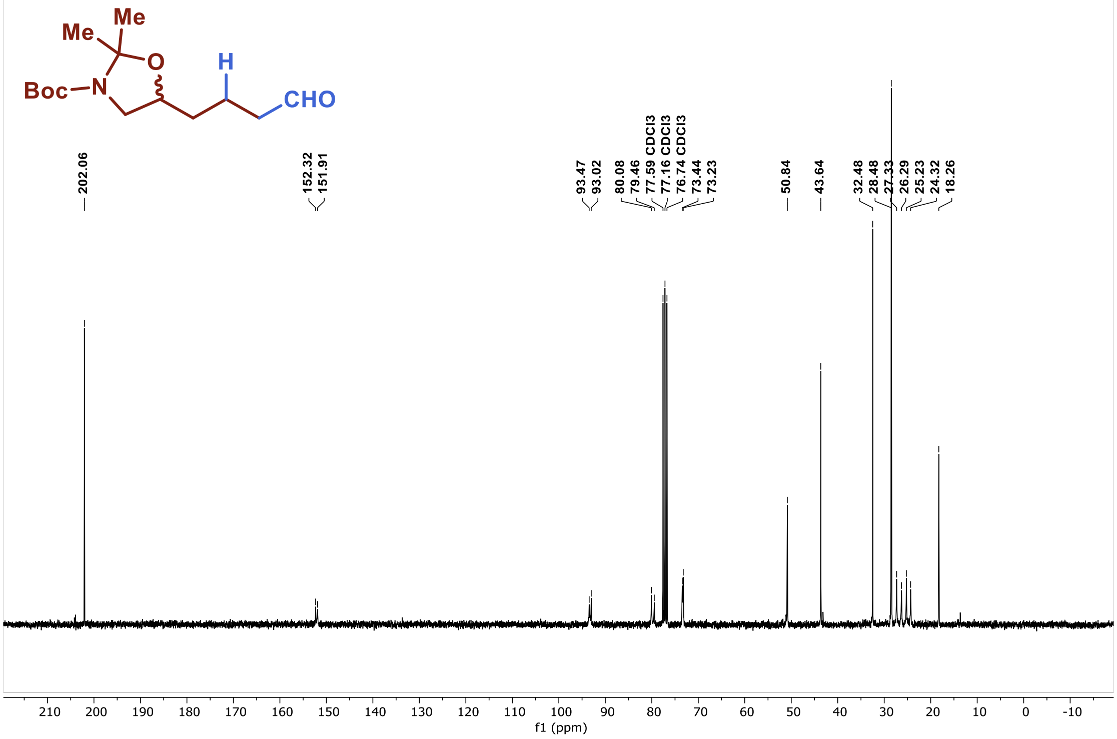

Compound **2k**: ^{1}H NMR (300 MHz, $CDCl_3$)

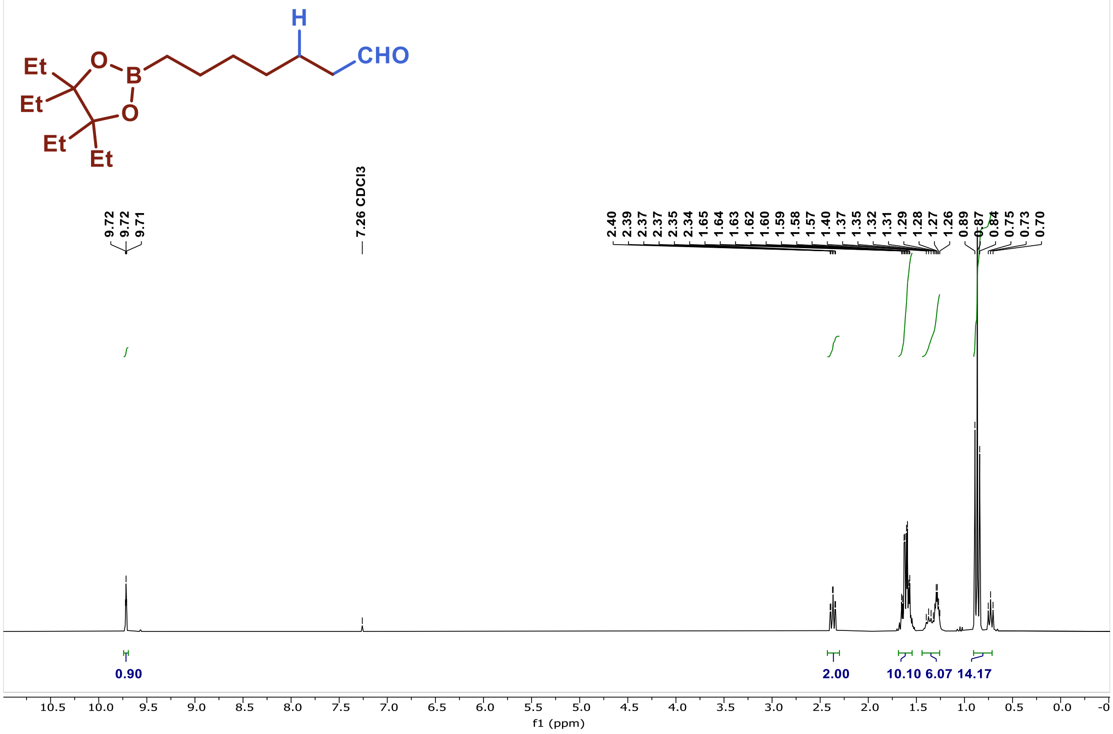

Compound **2k**: ^{13}C NMR (75 MHz, $CDCl_3$)

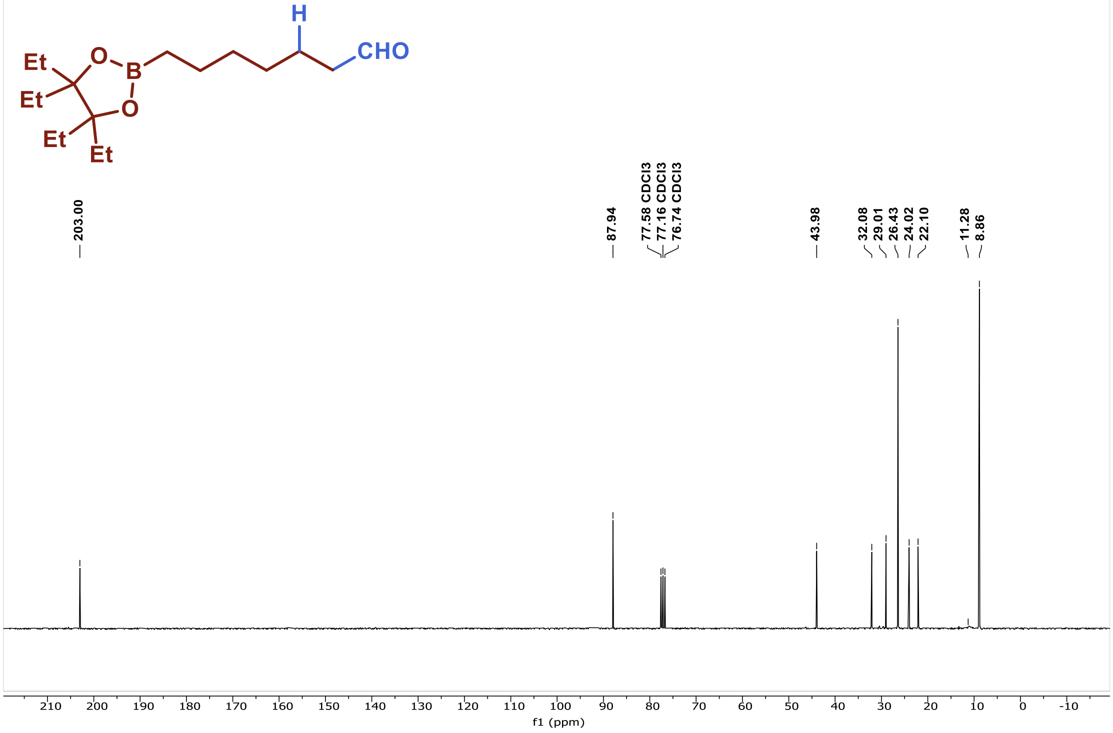

Compound **2l**: ^{1}H NMR (300 MHz, CDCl$_3$)

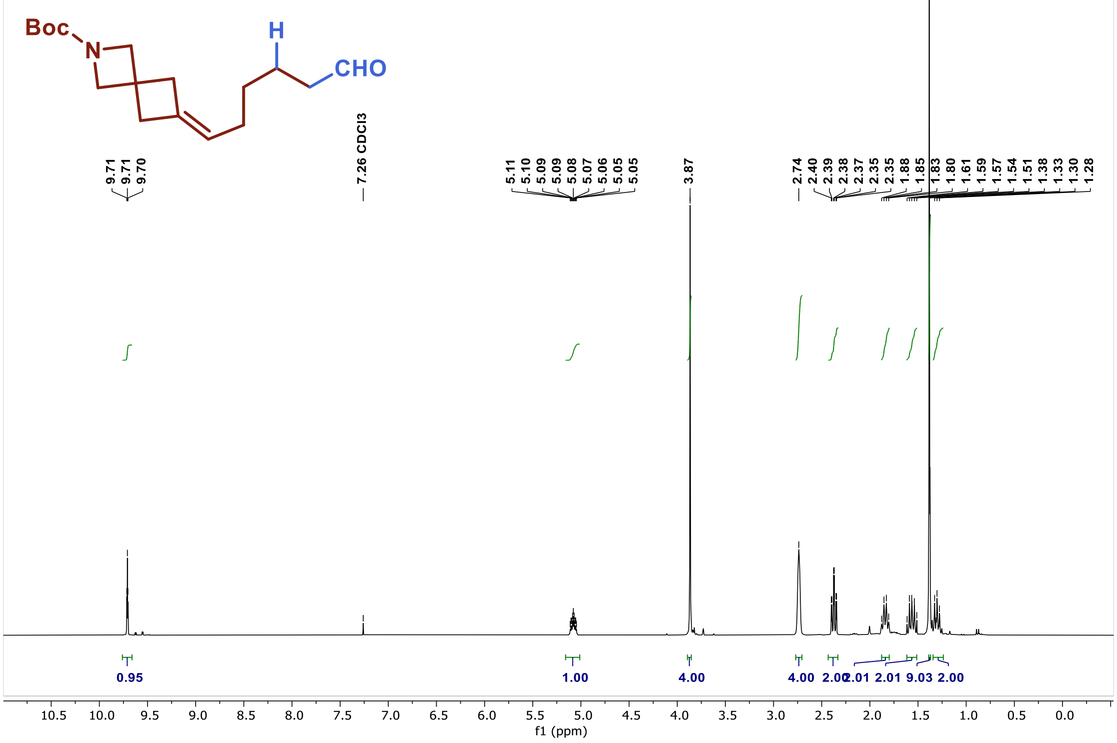

Compound **2l**: ^{13}C NMR (75 MHz, CDCl$_3$)

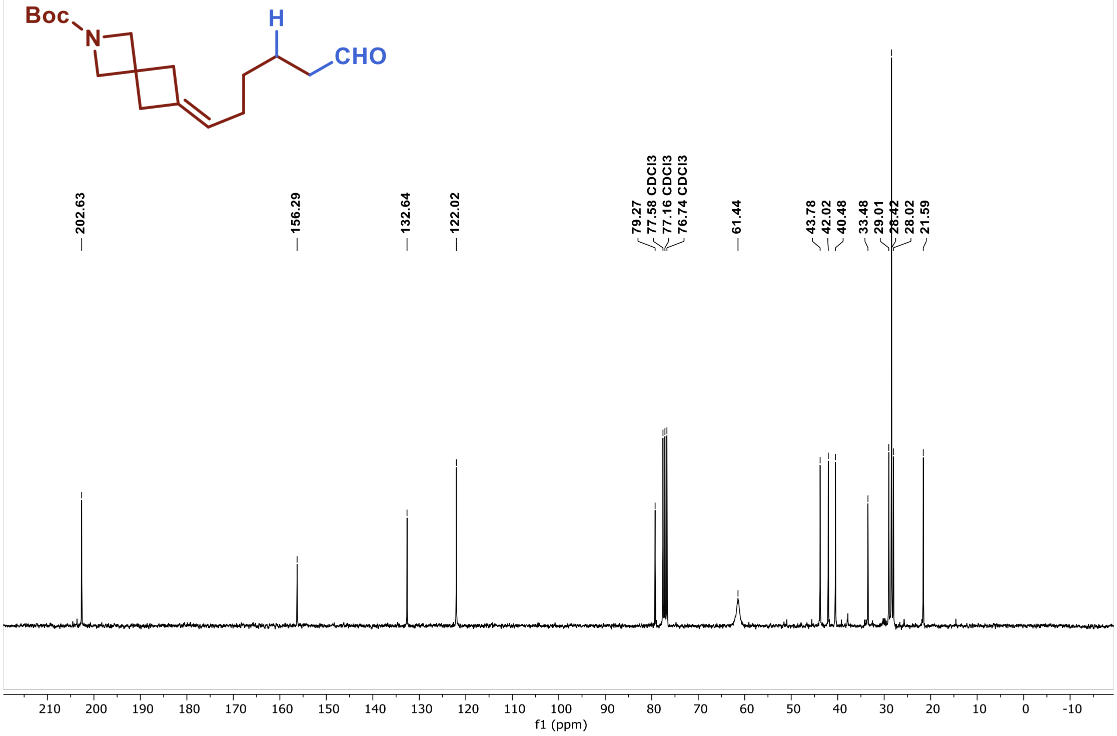

Compound **2m**: ^{1}H NMR (300 MHz, CDCl$_3$)

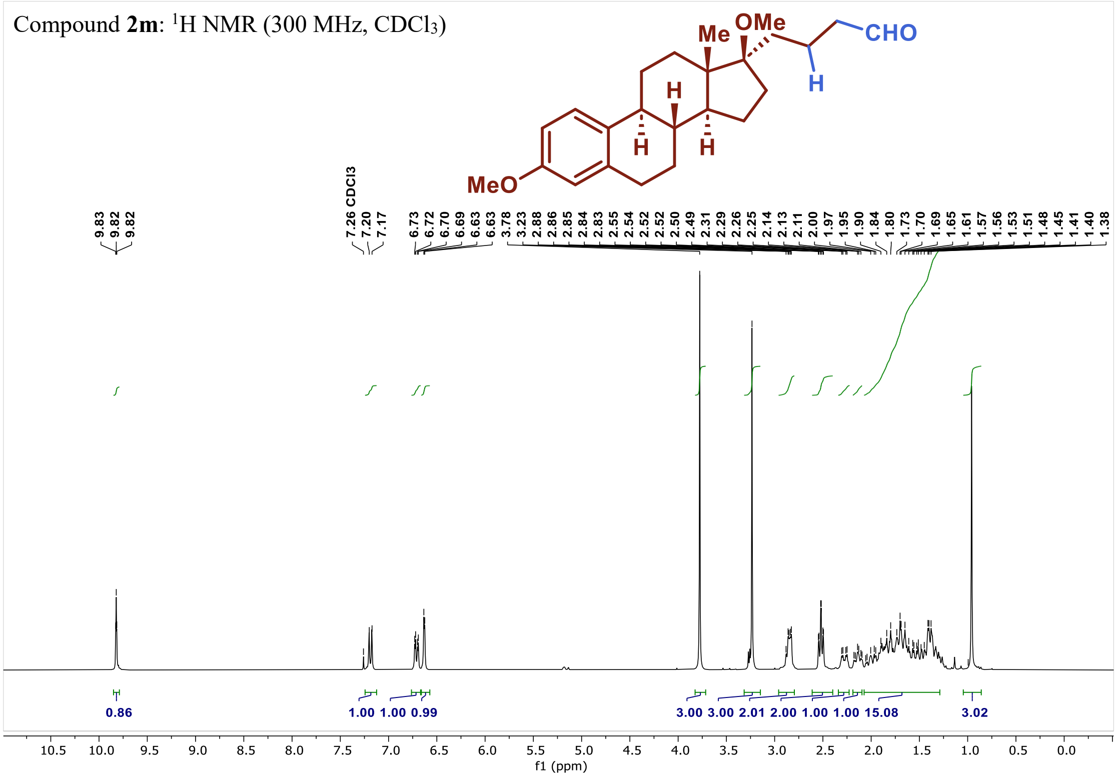

Compound **2m**: ^{13}C NMR (75 MHz, CDCl$_3$)

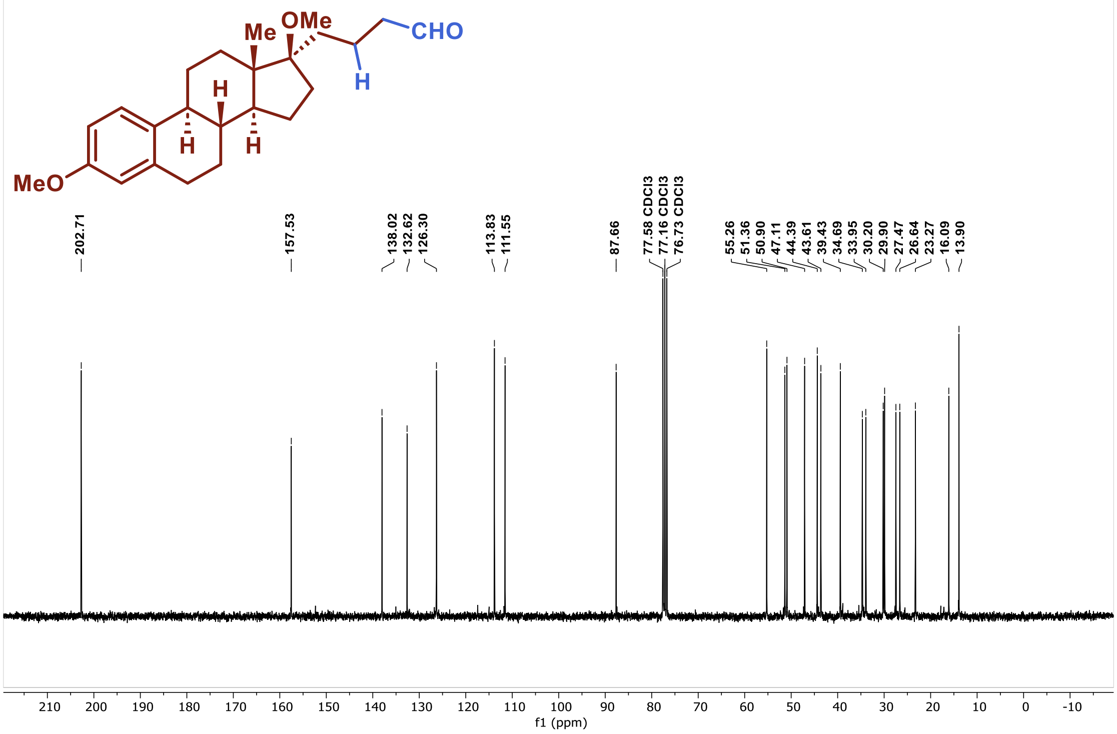

Compound **2m**: DEPT 135

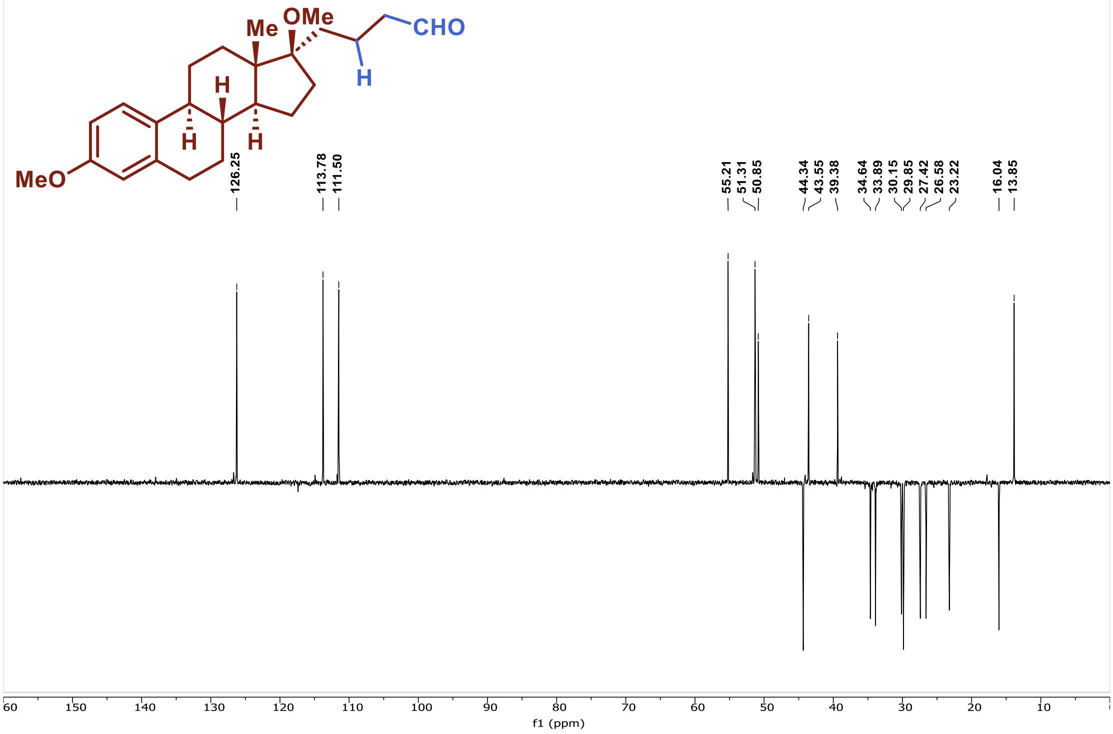

Compound **2m**: COSY

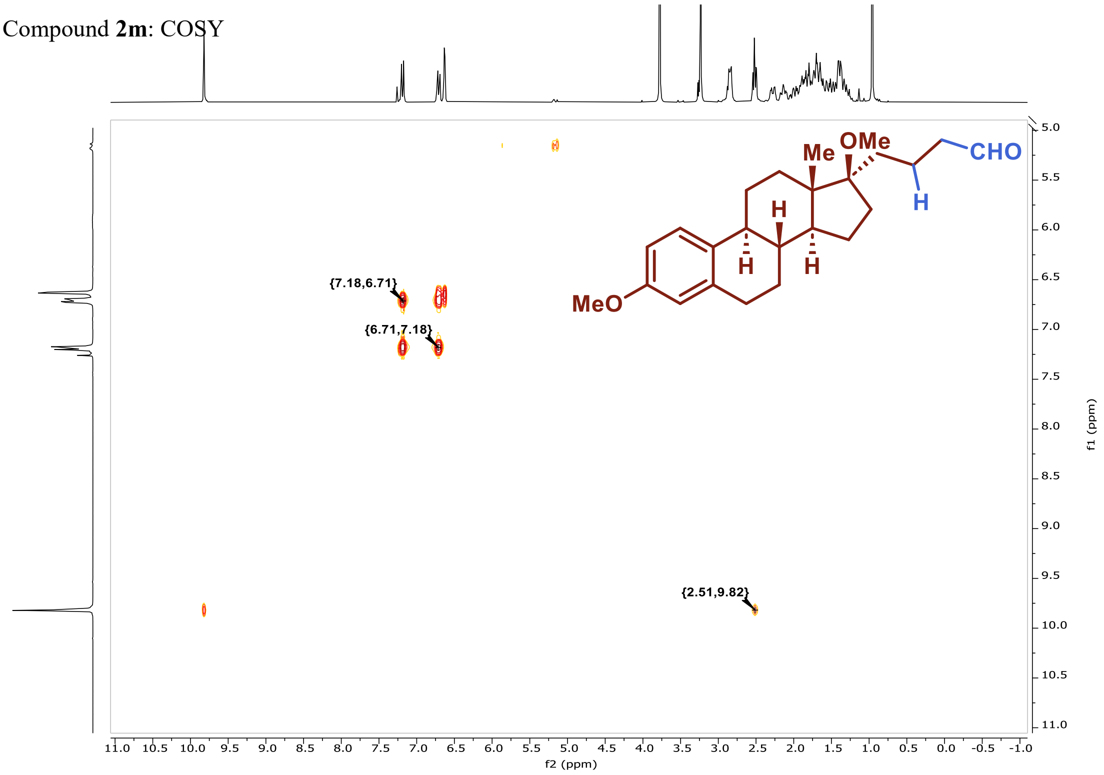

Compound **2m**: HSQC

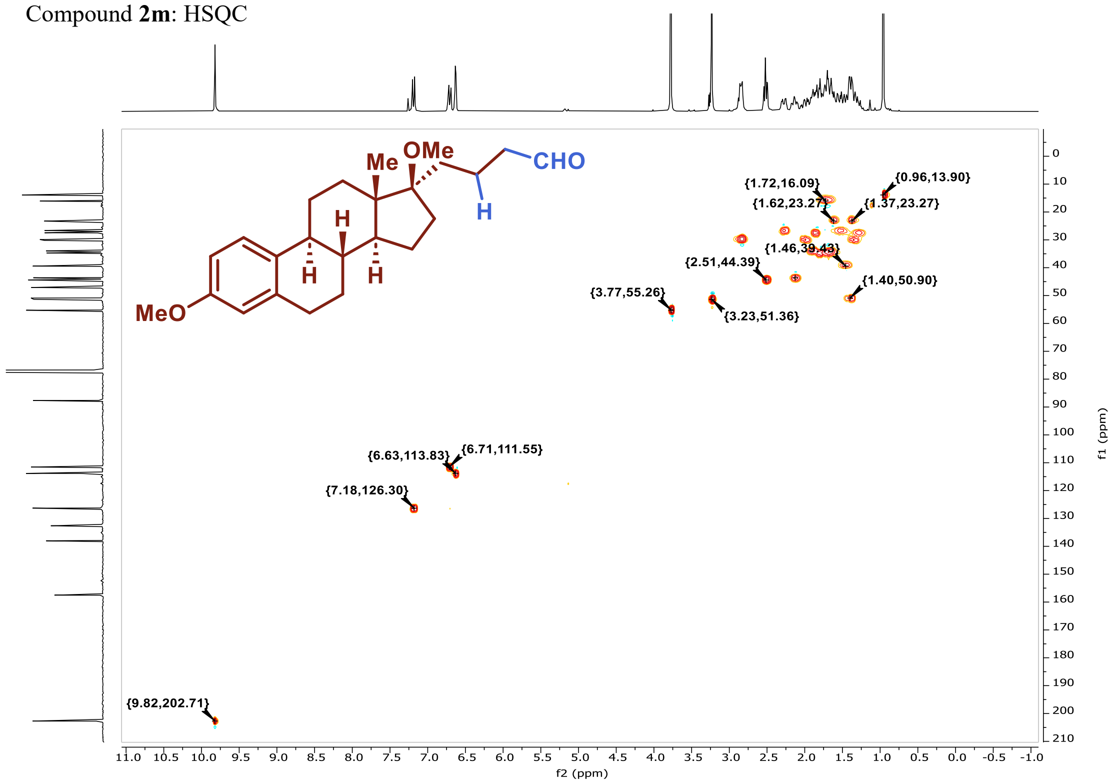

Compound **2m**: HMBC

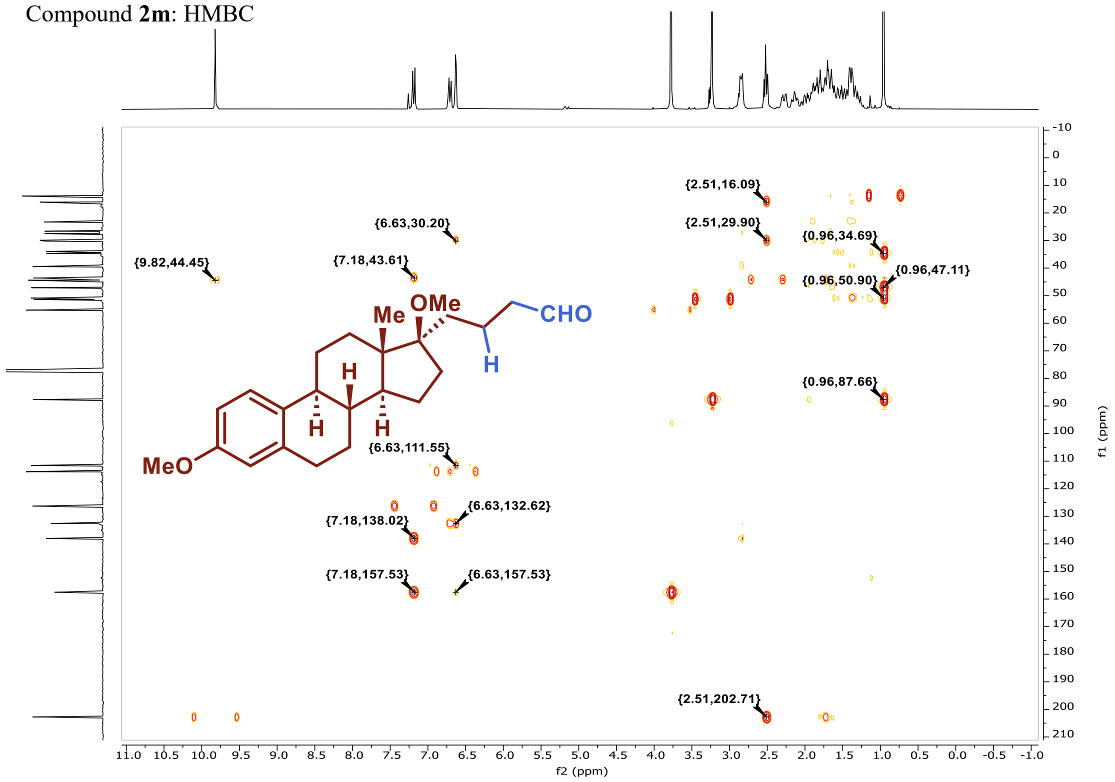

Compound **2m**: NOESY

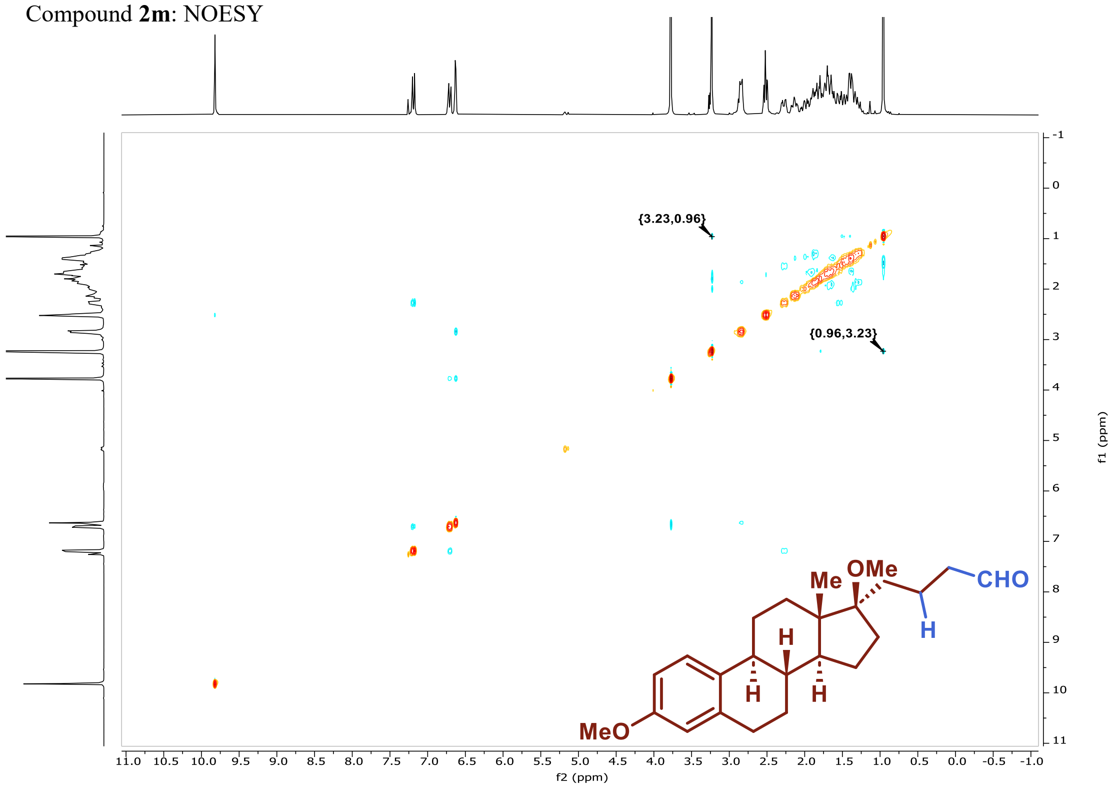

Compound **2n**: ^{1}H NMR (300 MHz, $CDCl_3$)

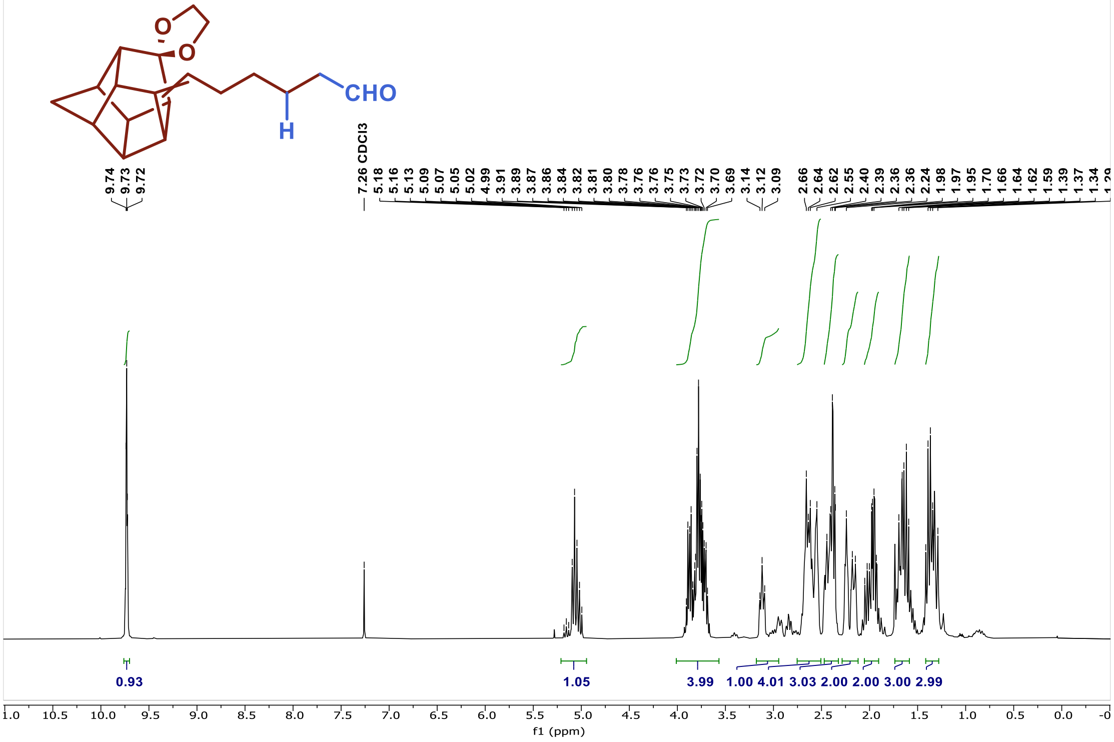

Compound **2n**: ^{13}C NMR (75 MHz, $CDCl_3$)

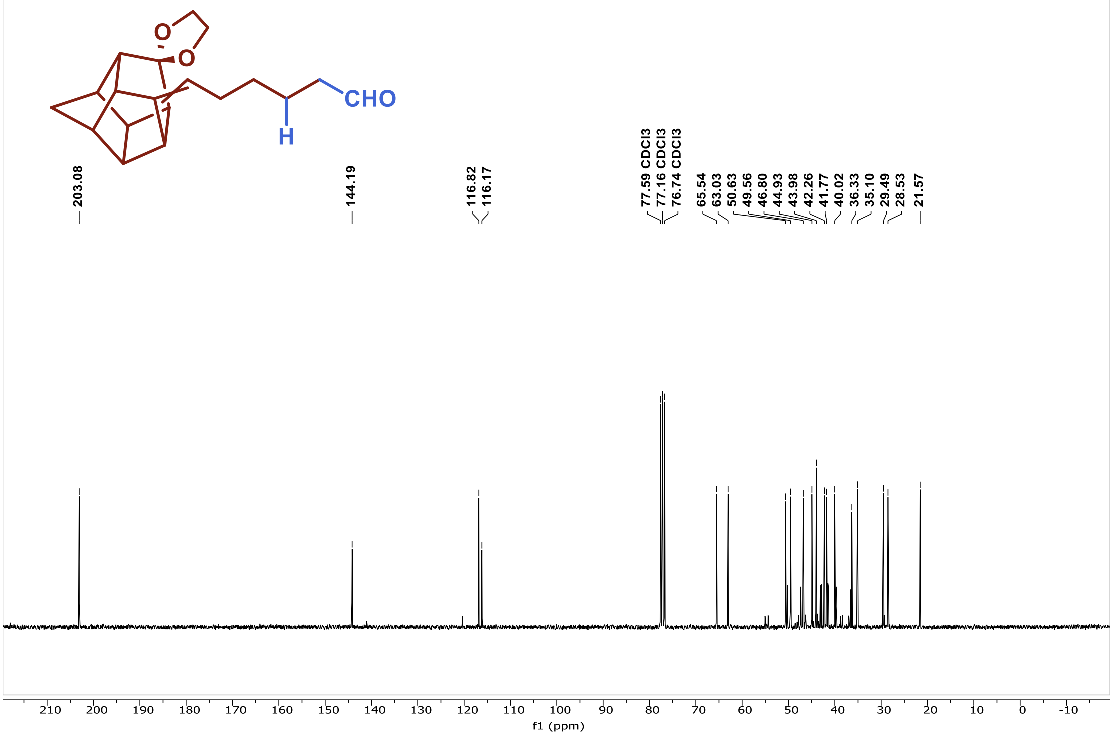

Compound **2o**: ^{1}H NMR (300 MHz, $CDCl_3$)

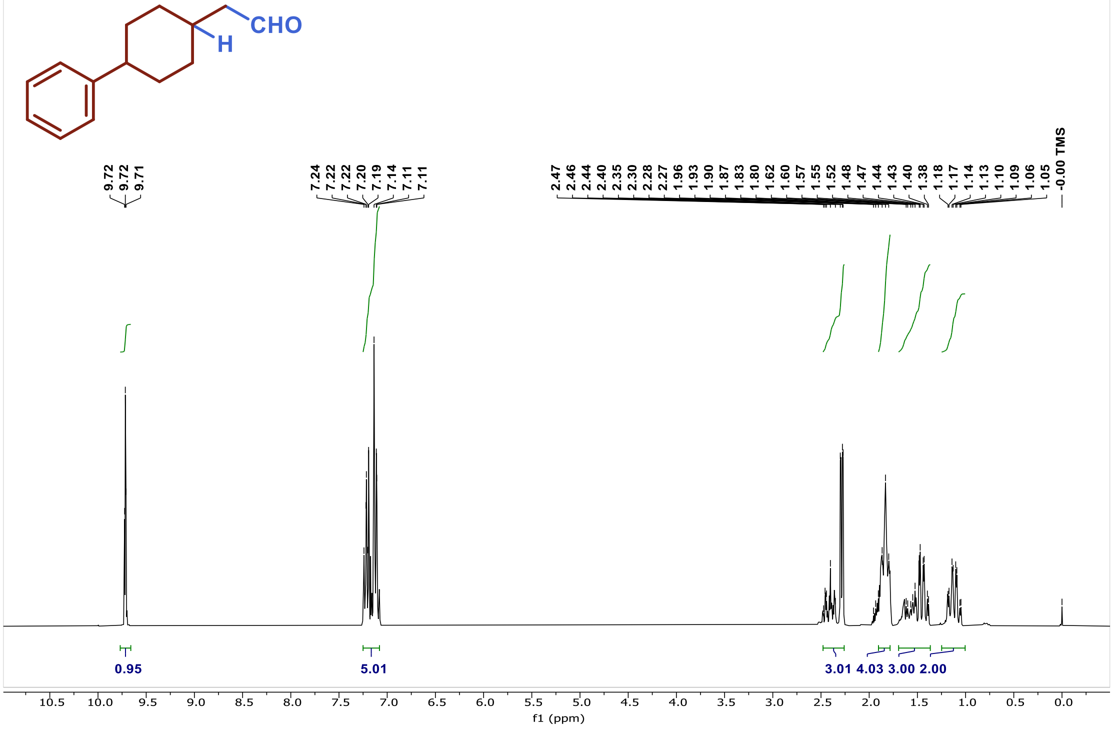

Compound **2o**: ^{13}C NMR (75 MHz, $CDCl_3$)

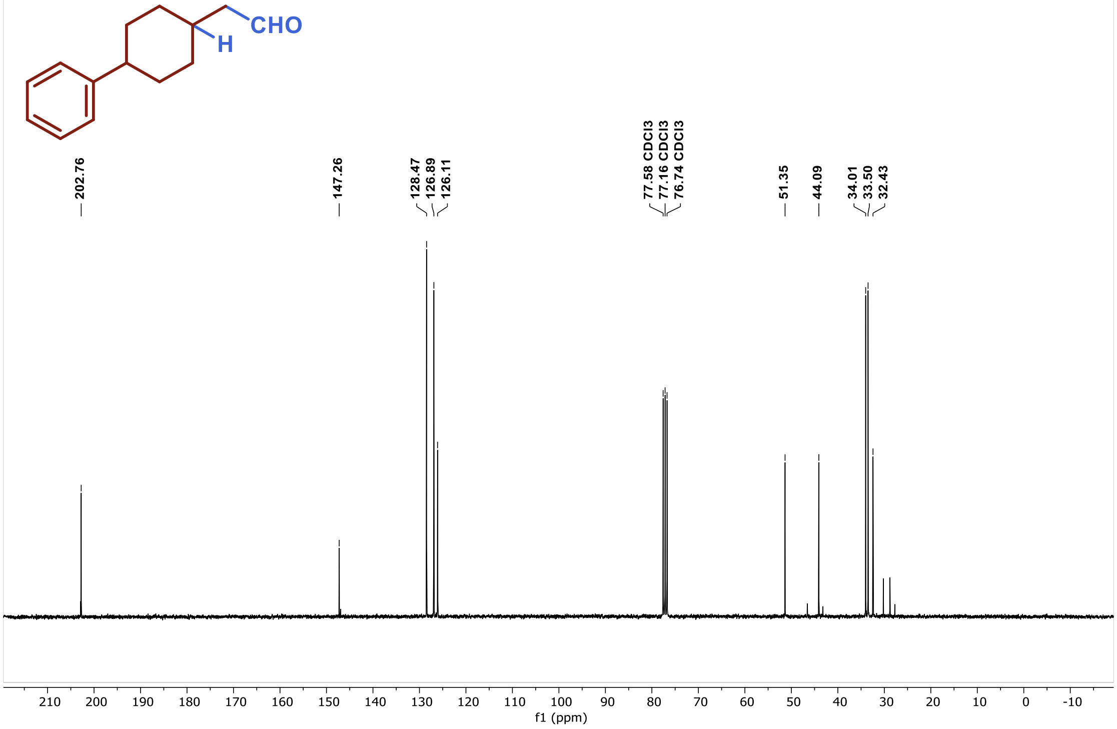

Compound **2p**: ^{1}H NMR (300 MHz, $CDCl_3$)

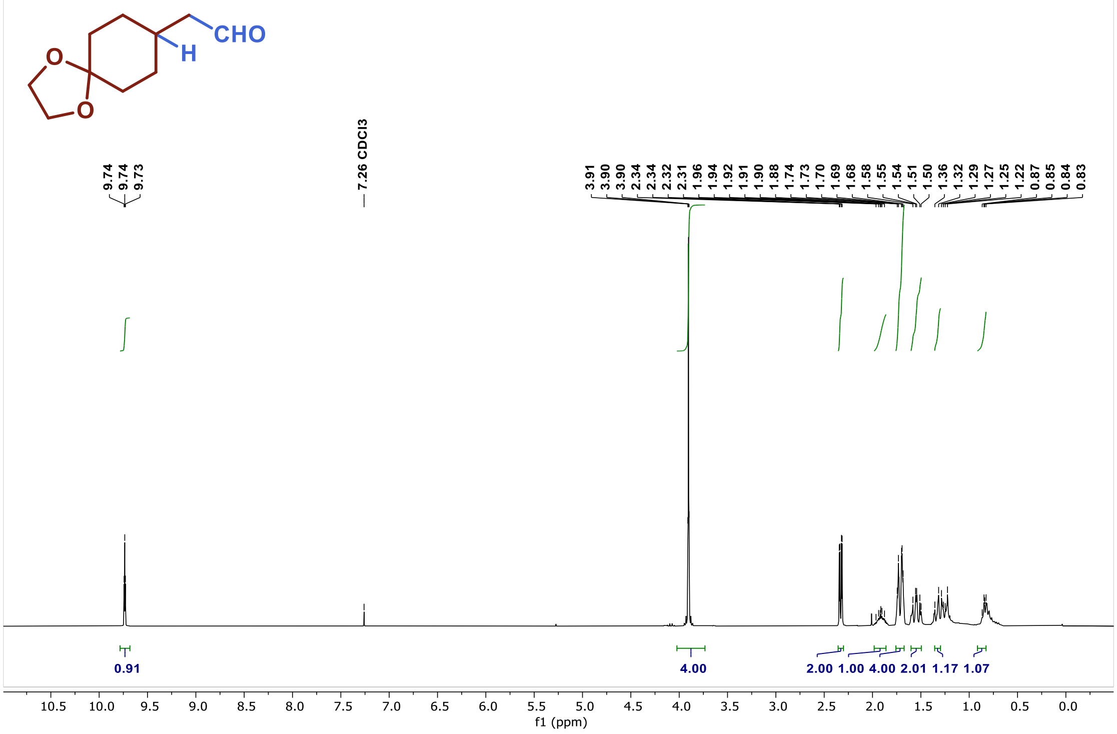

Compound **2p**: ^{13}C NMR (75 MHz, $CDCl_3$)

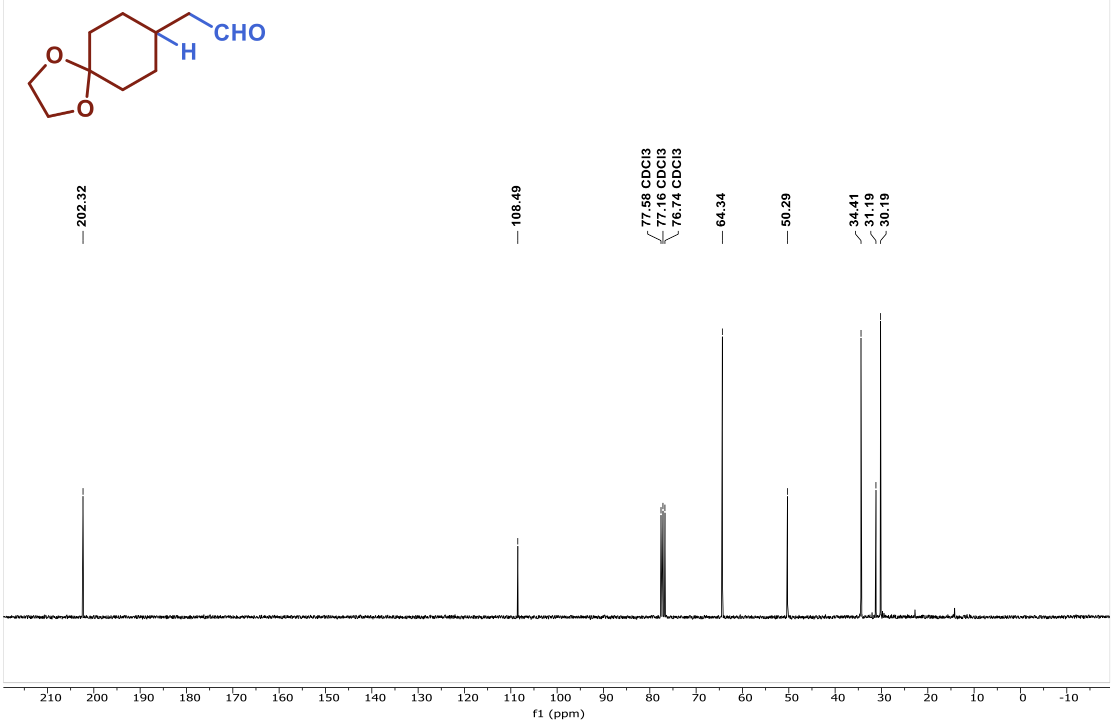

Compound **2q**: ^{1}H NMR (300 MHz, $CDCl_3$)

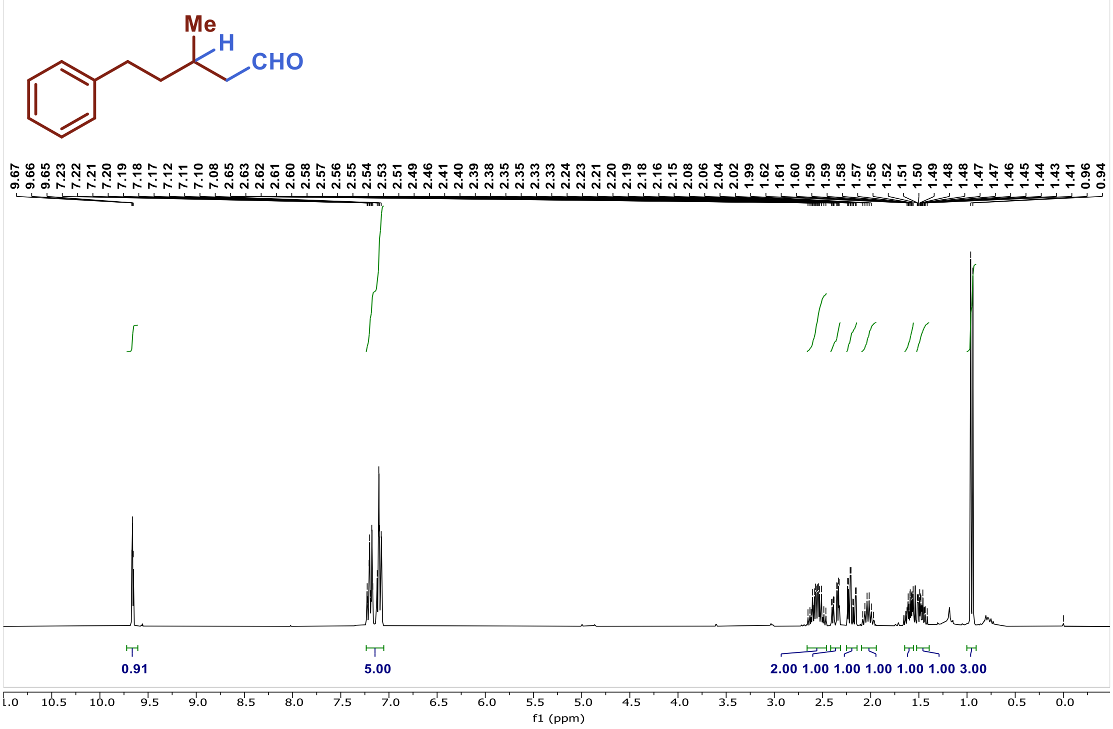

Compound **2q**: ^{13}C NMR (75 MHz, $CDCl_3$)

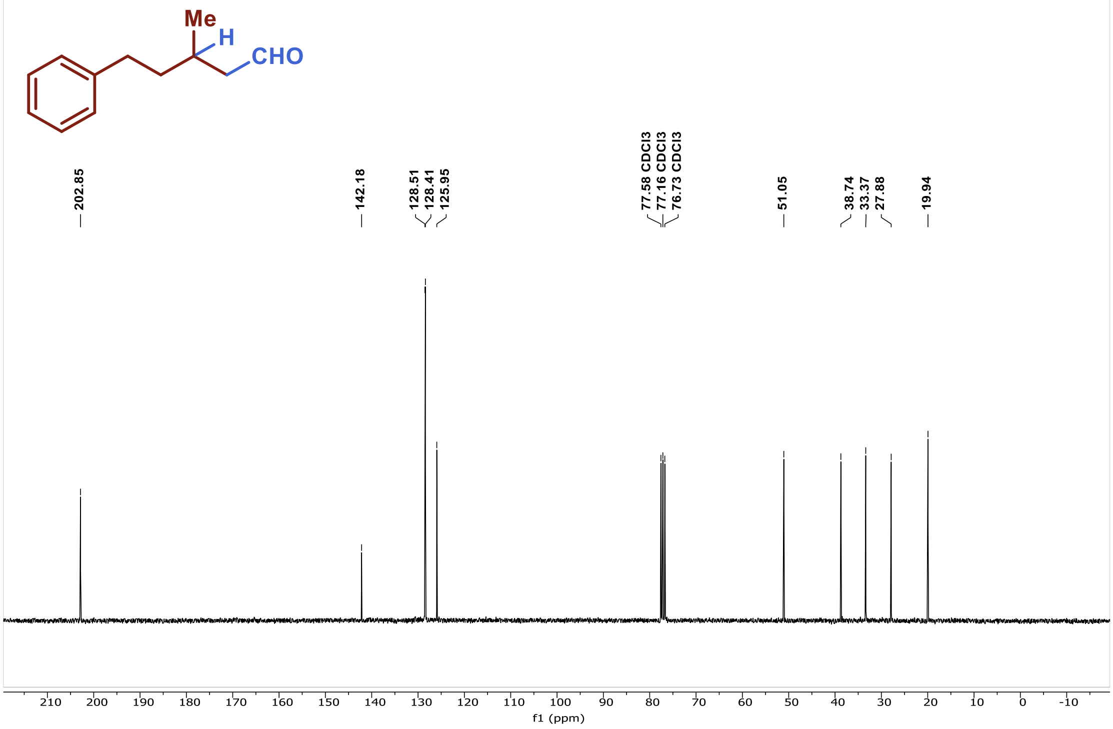

Compound **2r**: ^{1}H NMR (300 MHz, $CDCl_3$)

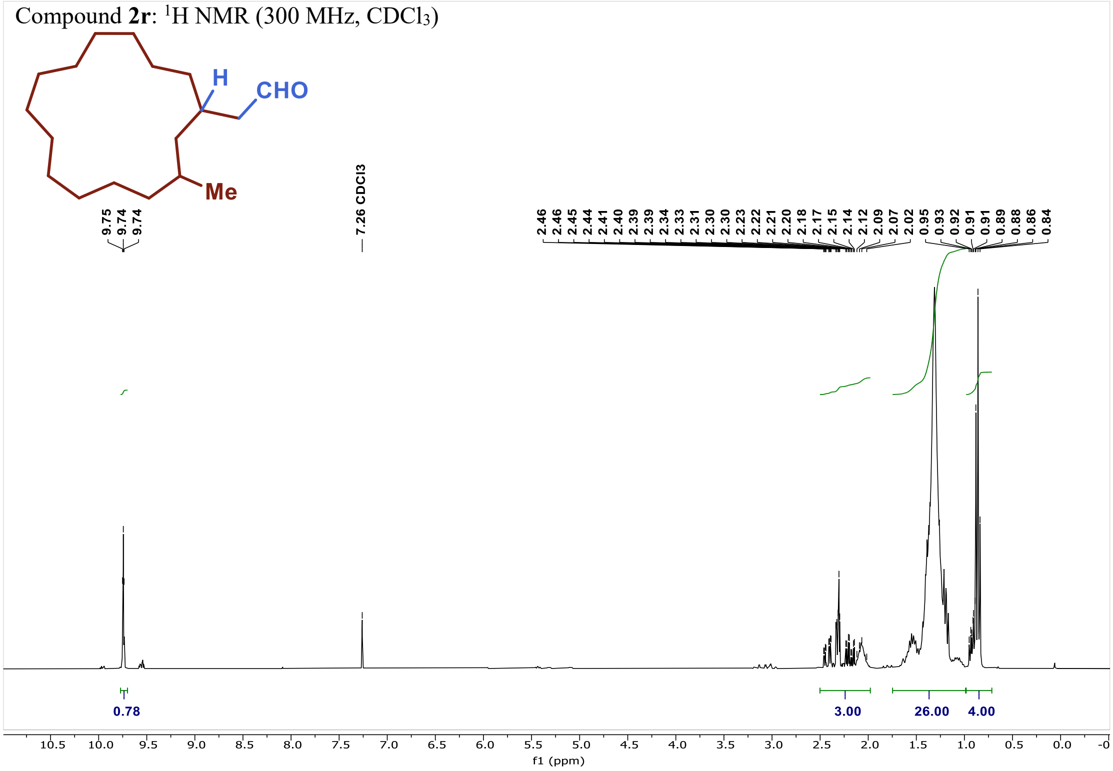

Compound **2r**: ^{13}C NMR (75 MHz, $CDCl_3$)

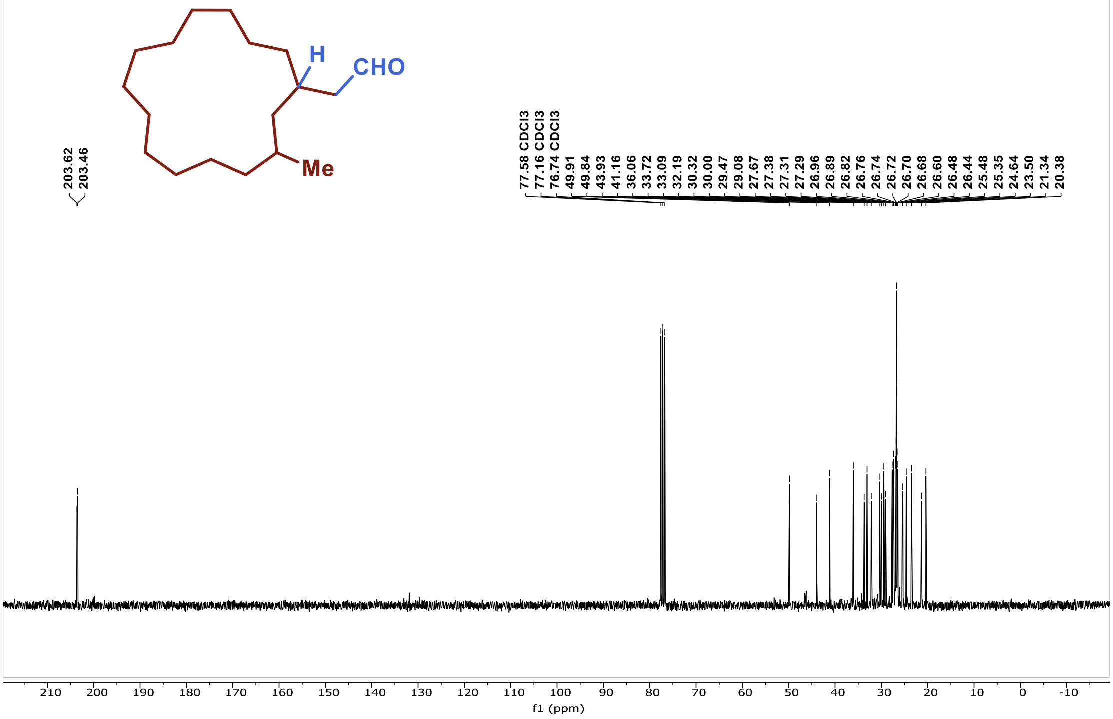

Compound **2s**: ^{1}H NMR (300 MHz, $CDCl_3$)

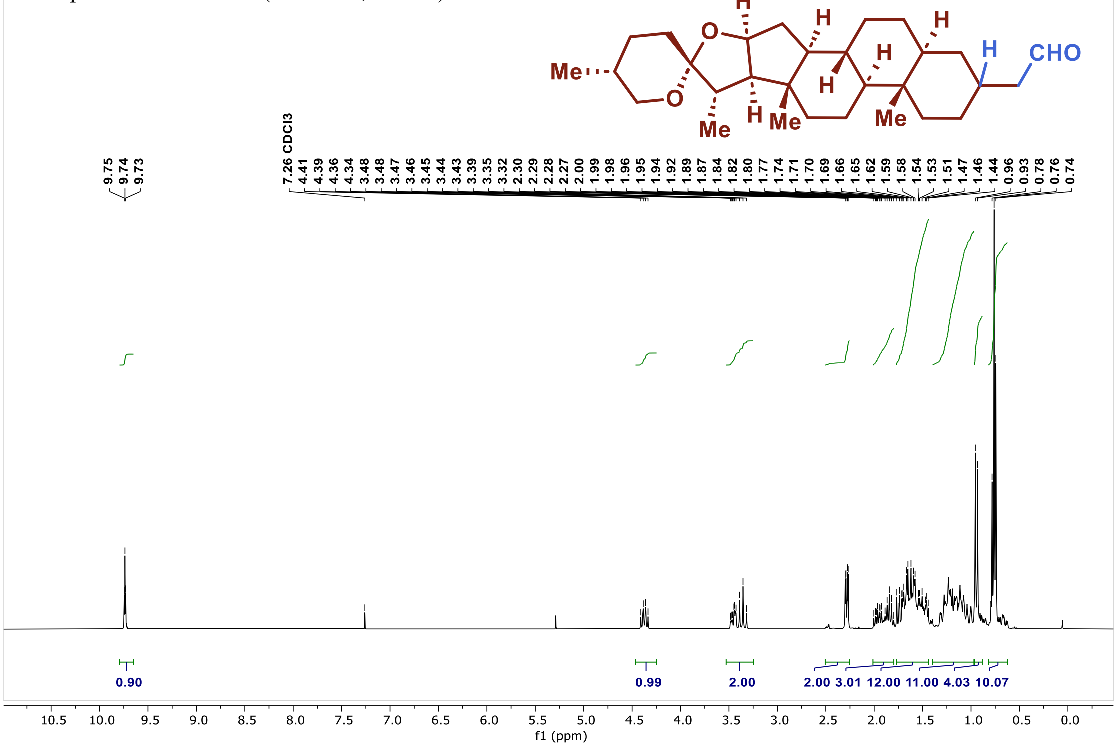

Compound **2s**: ^{13}C NMR (75 MHz, $CDCl_3$)

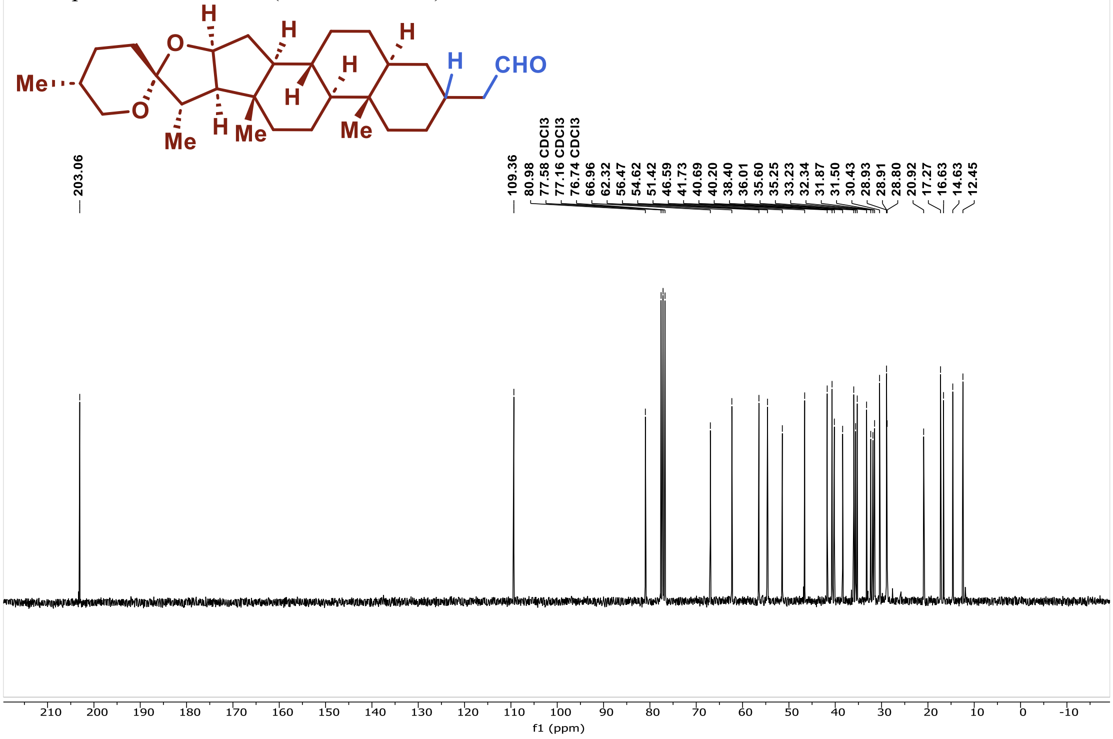

Compound **2s**: DEPT 135

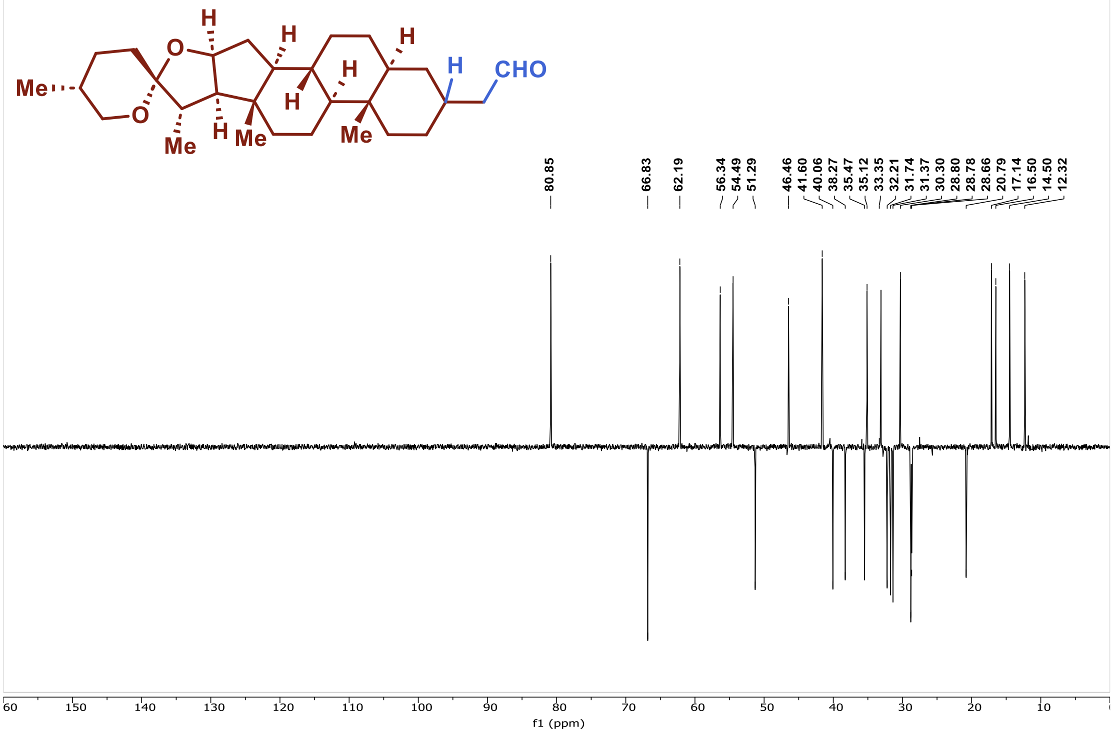

Compound **2s**: COSY

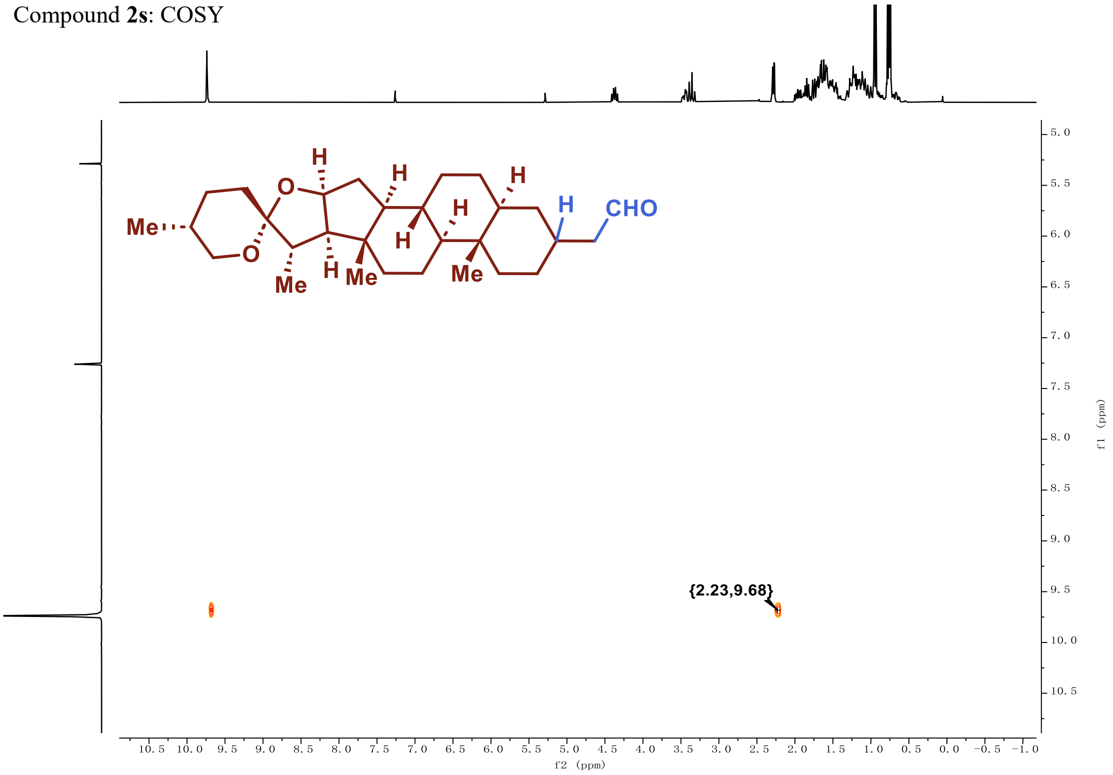

Compound **2s**: HSQC

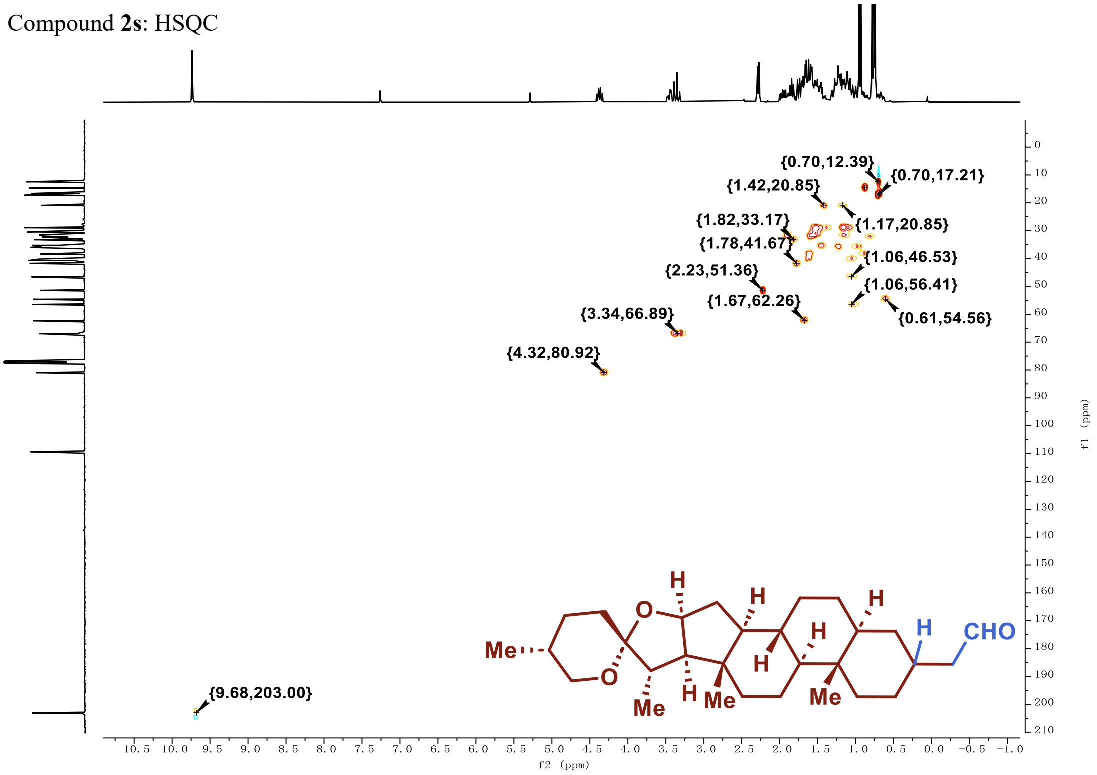

Compound **2s**: HMBC

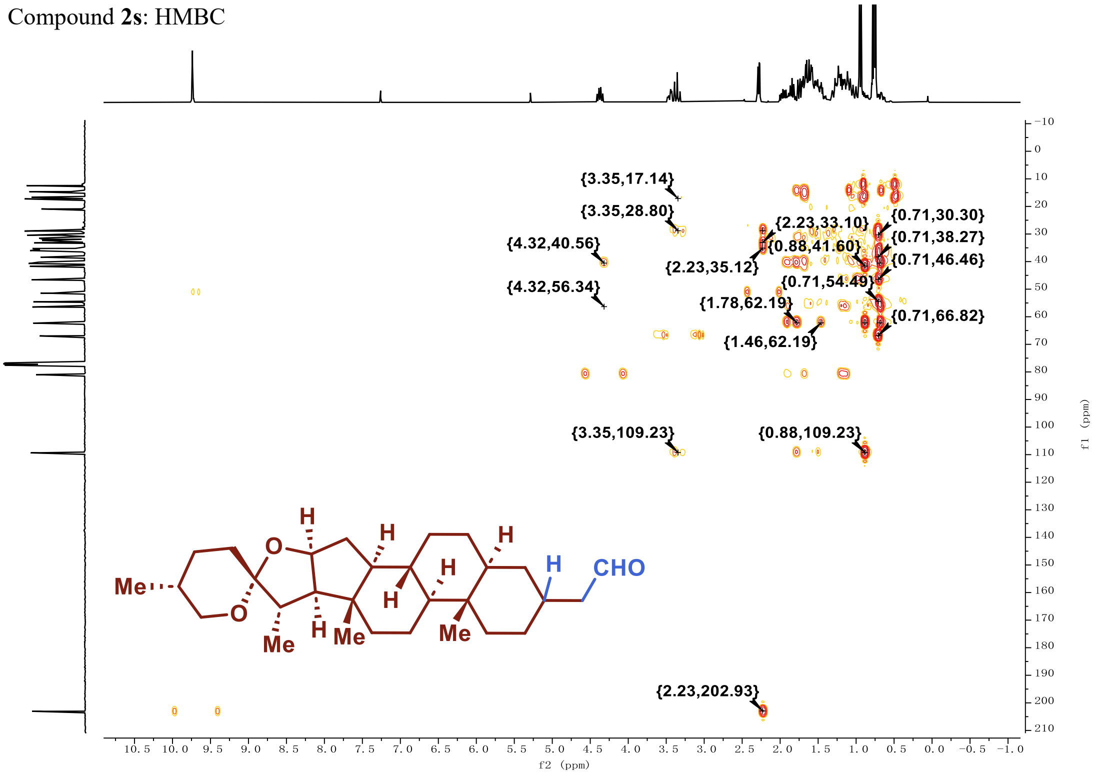

Compound **2s**: NOESY

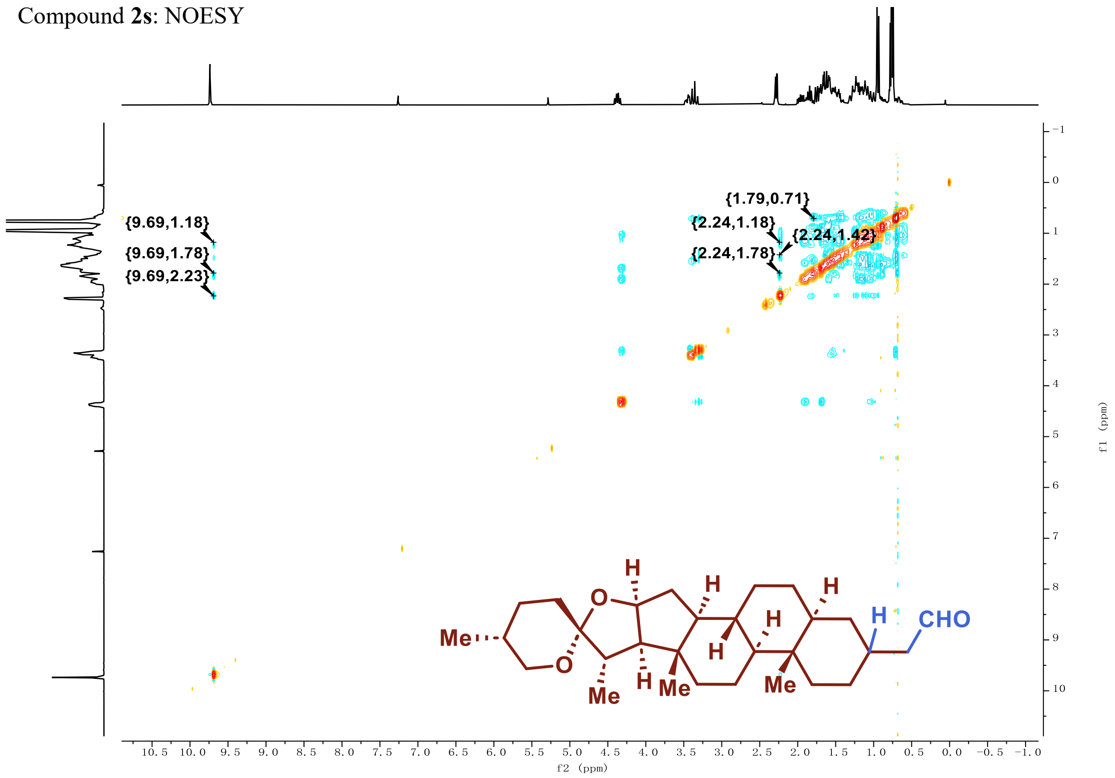

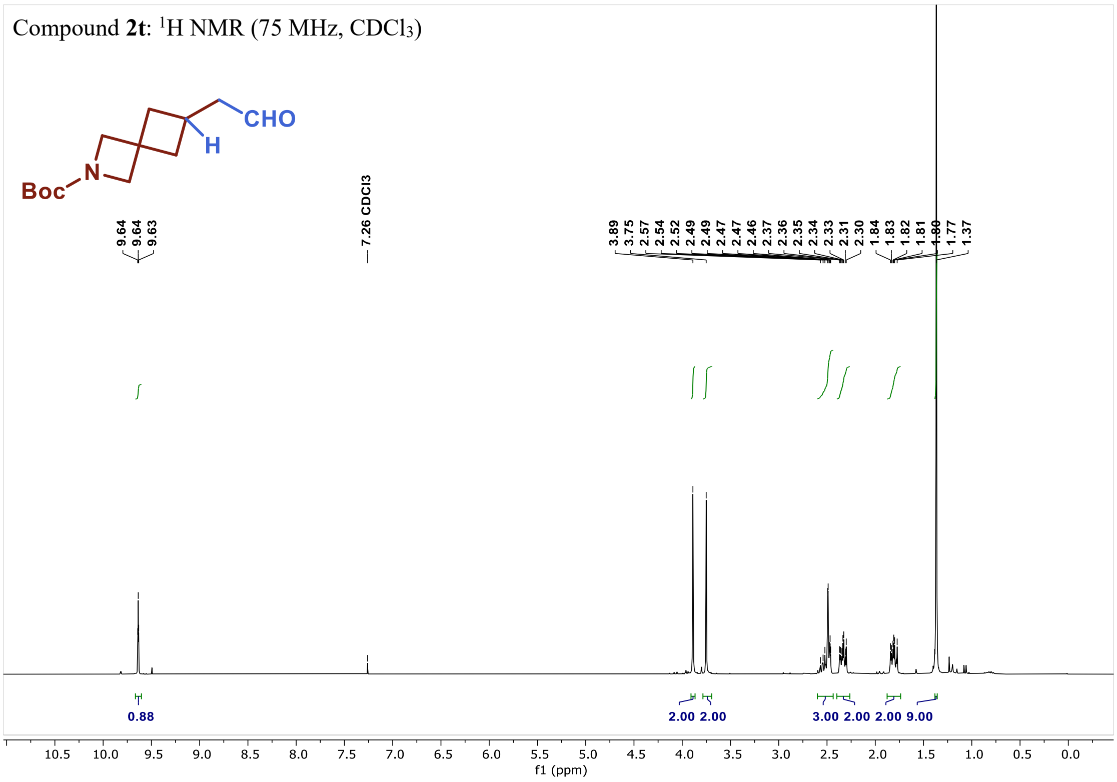

Compound **2t**: ^{1}H NMR (75 MHz, $CDCl_3$)

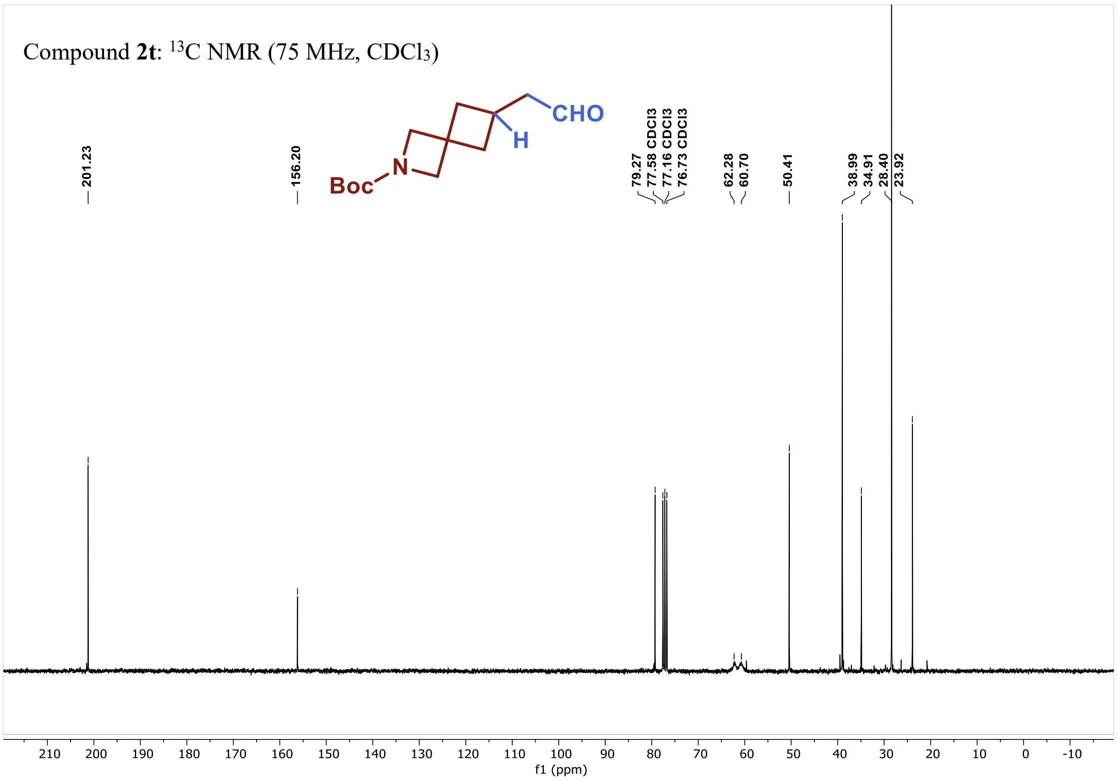

Compound **2t**: ^{13}C NMR (75 MHz, $CDCl_3$)

Compound **2u**: ^{1}H NMR (300 MHz, $CDCl_3$)

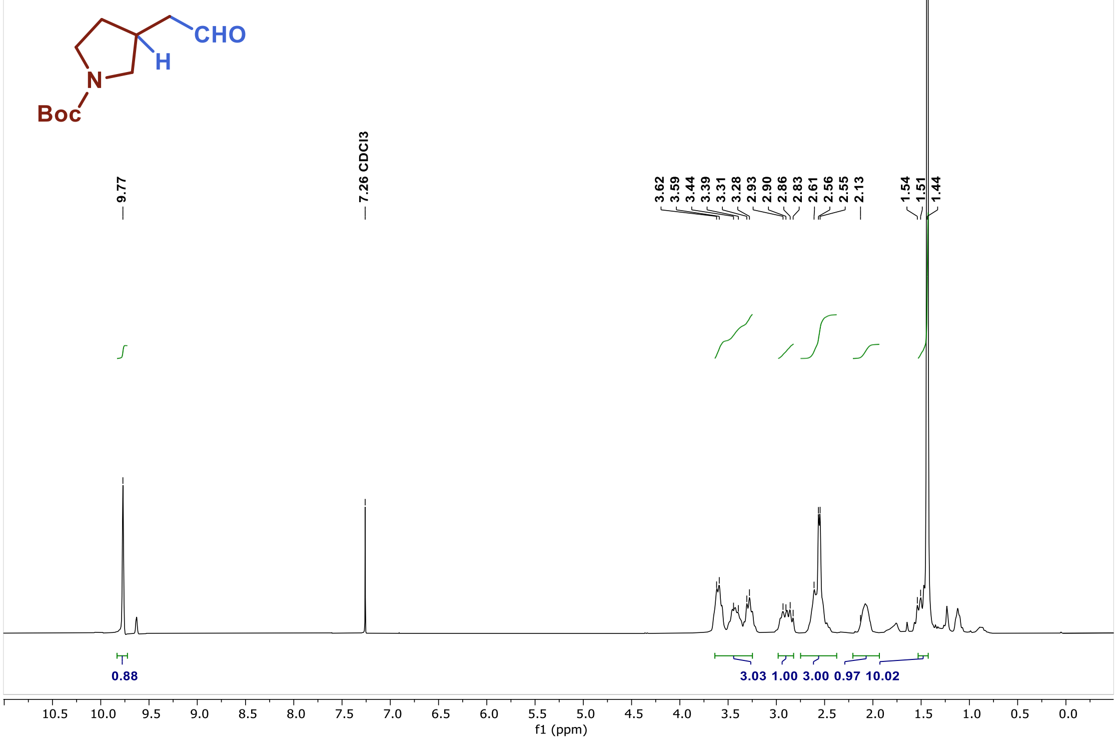

Compound **2u**: ^{13}C NMR (75 MHz, $CDCl_3$)

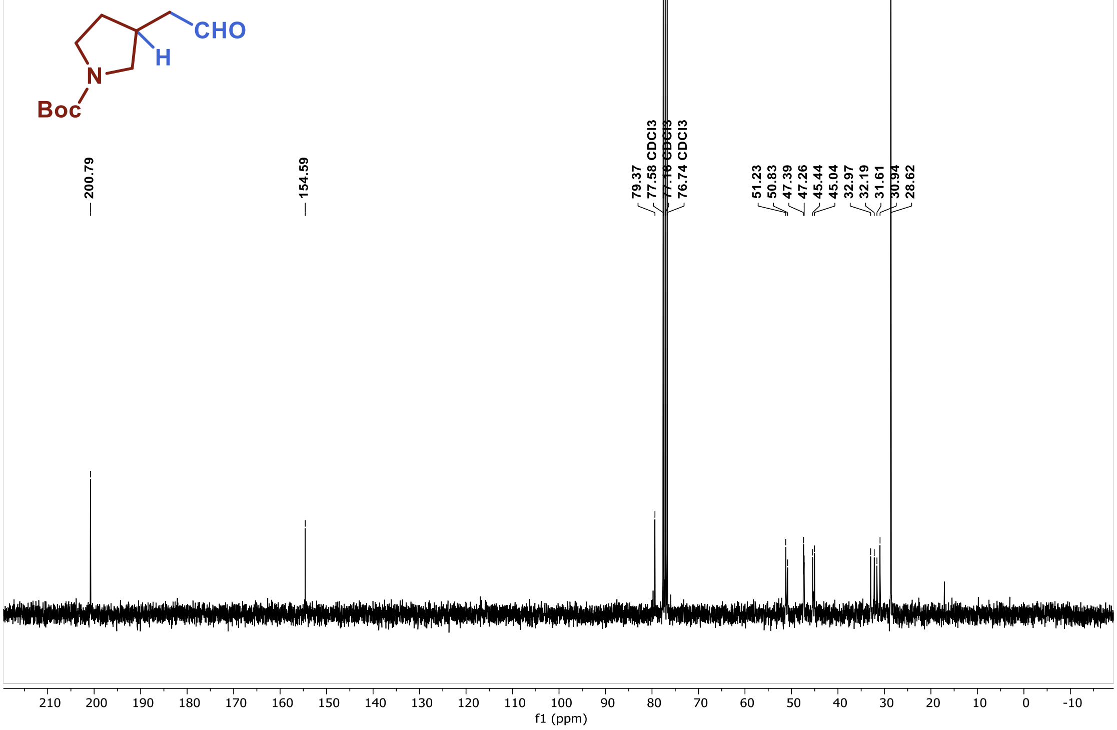

Compound **2v**: ^{1}H NMR (300 MHz, $CDCl_3$)

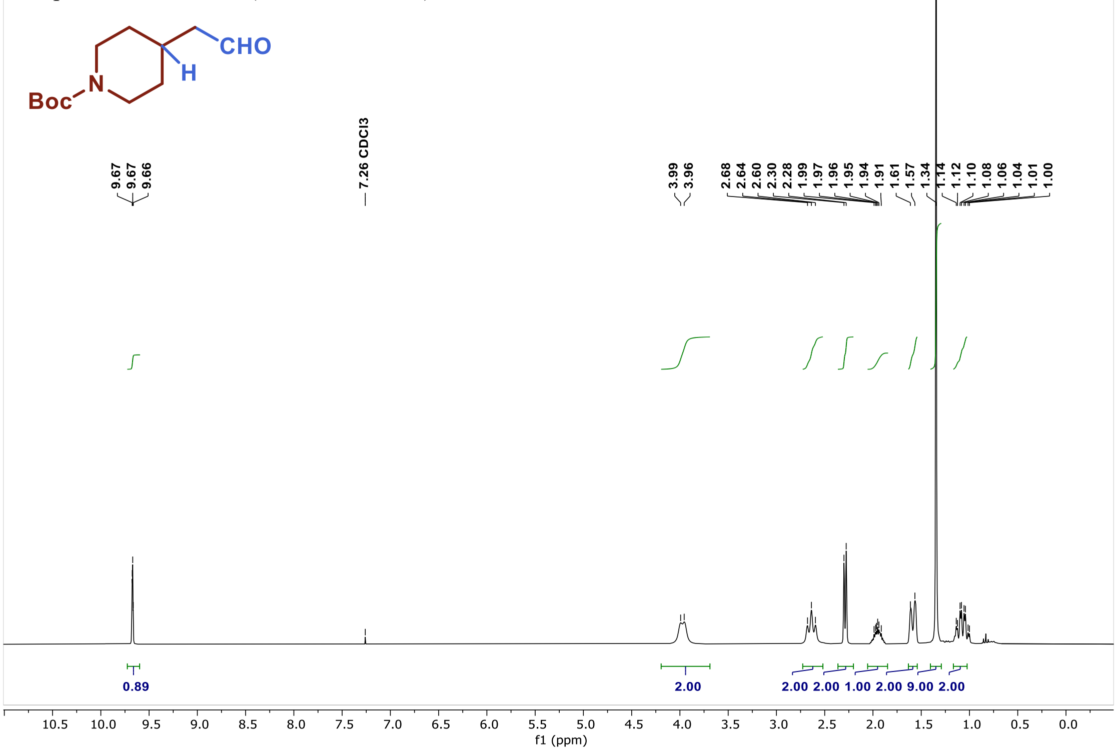

Compound **2v**: ^{13}C NMR (75 MHz, $CDCl_3$)

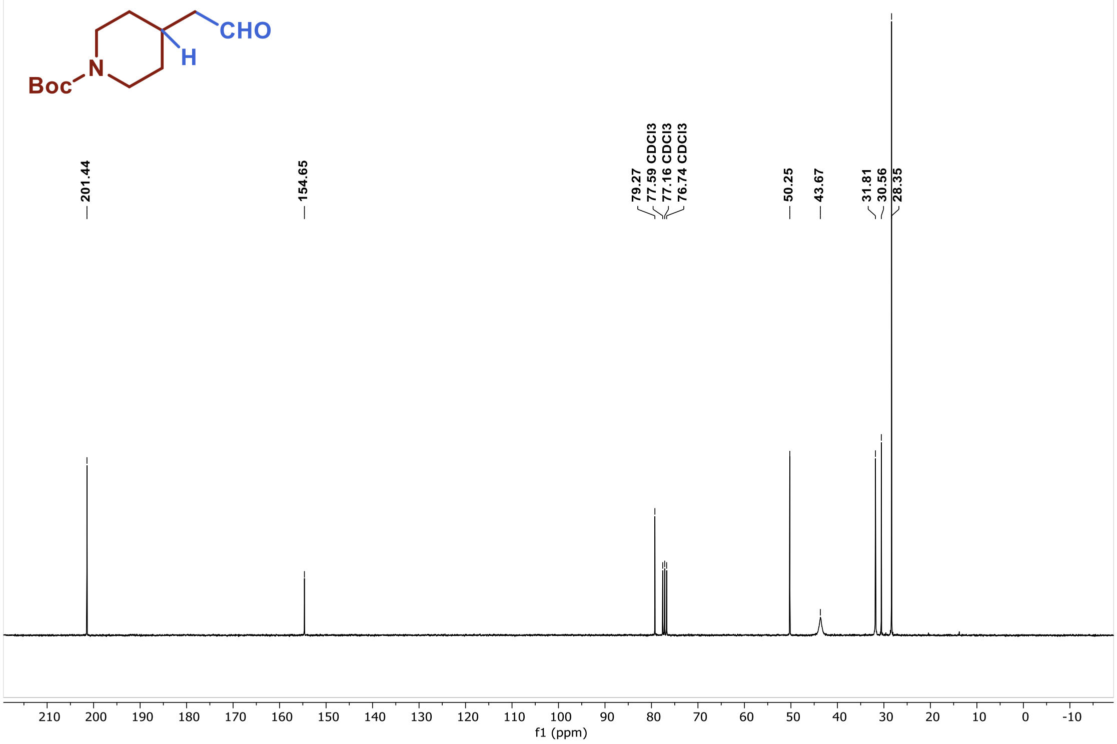

Compound **2w**: ^{1}H NMR (300 MHz, $CDCl_3$)

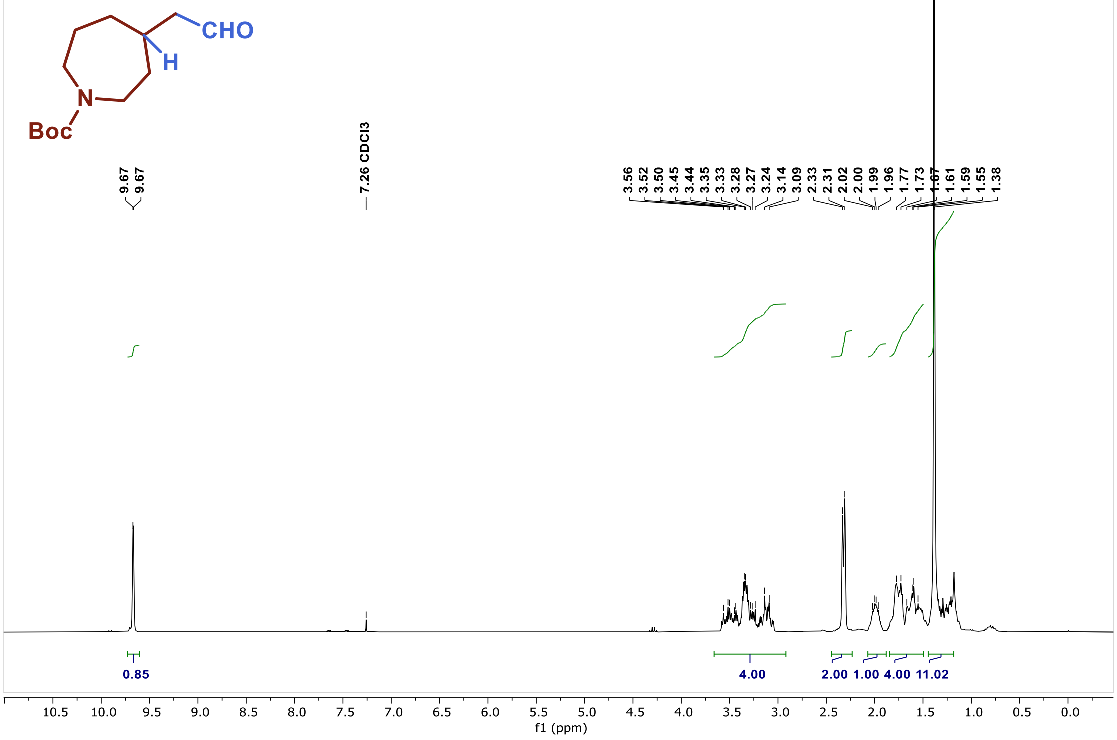

Compound **2w**: ^{13}C NMR (75 MHz, $CDCl_3$)

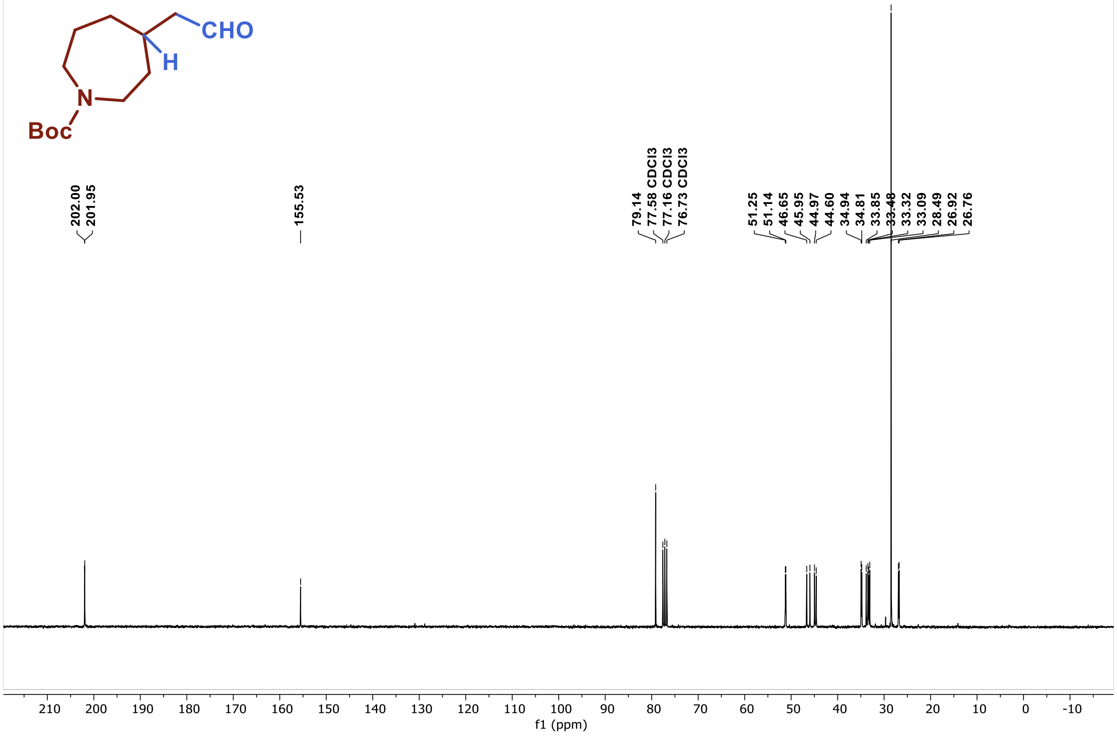

Compound **2x**: ^{1}H NMR (300 MHz, $CDCl_3$)

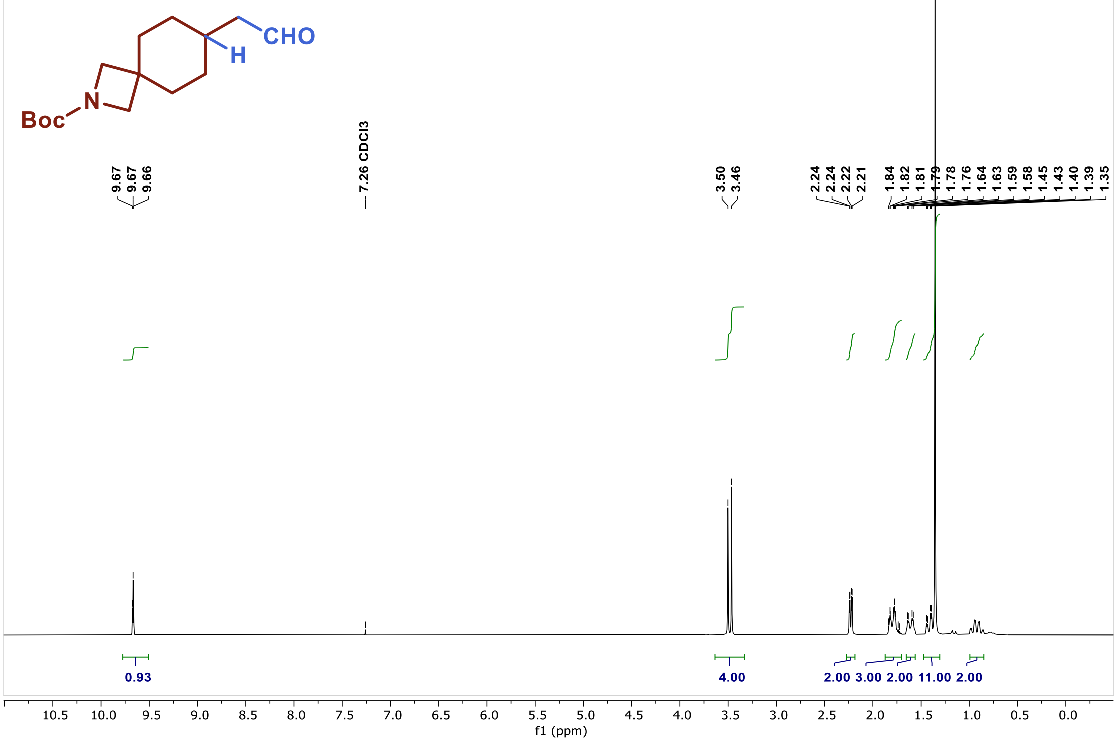

Compound **2x**: ^{13}C NMR (75 MHz, $CDCl_3$)

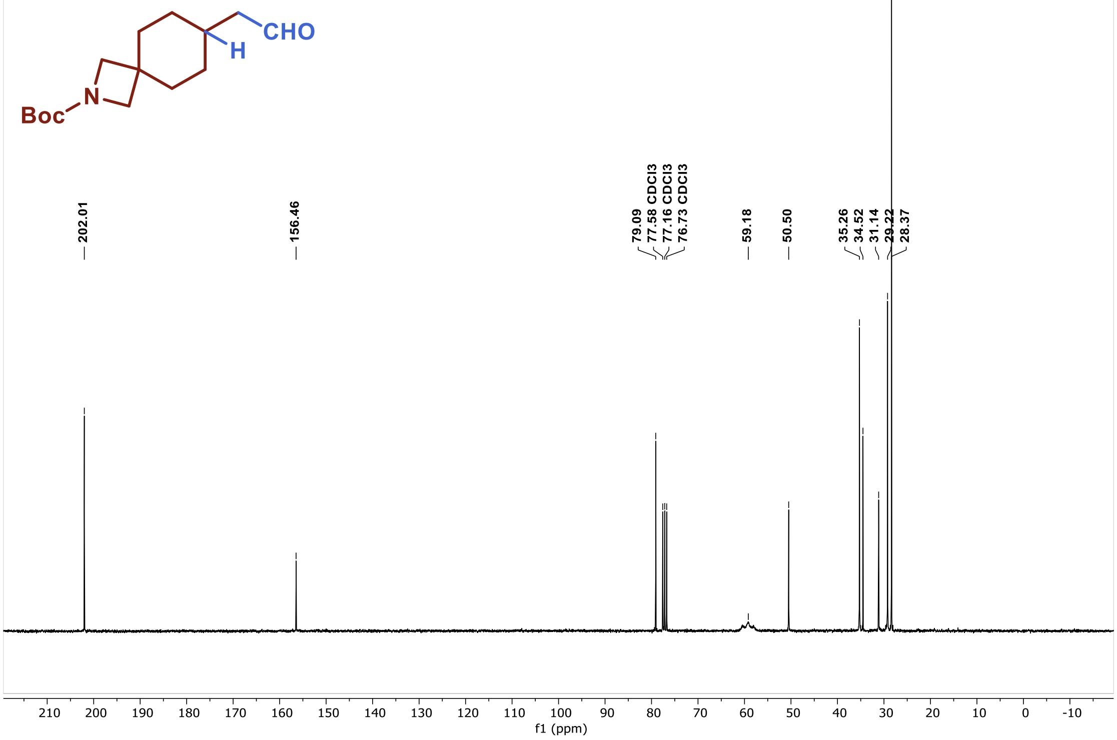

Compound **2y**: ^{1}H NMR (300 MHz, $CDCl_3$)

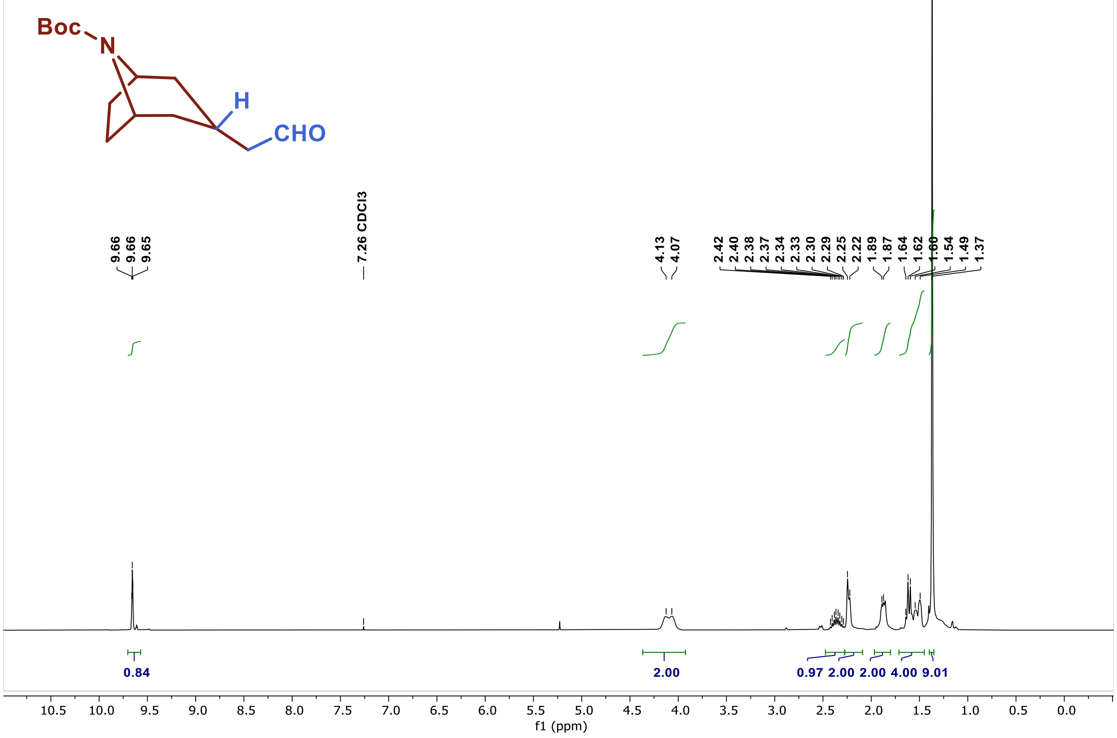

Compound **2y**: ^{13}C NMR (75 MHz, $CDCl_3$)

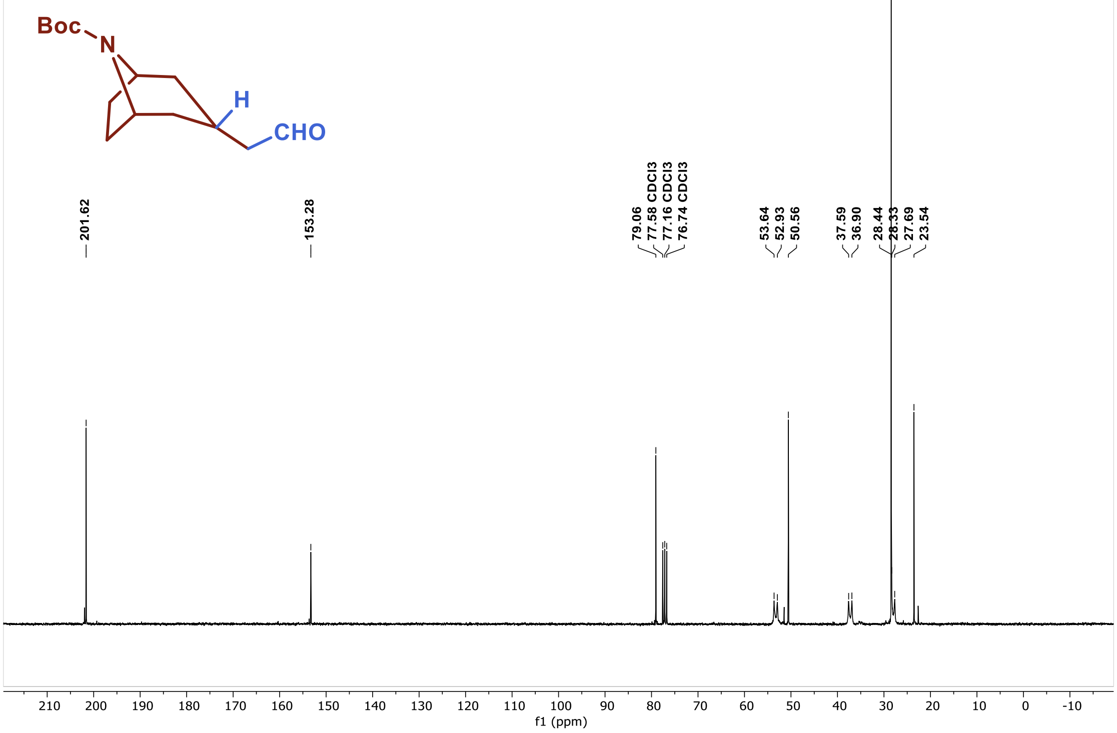

Amines

Compound **3a**: ^{1}H NMR (300 MHz, $CDCl_3$)

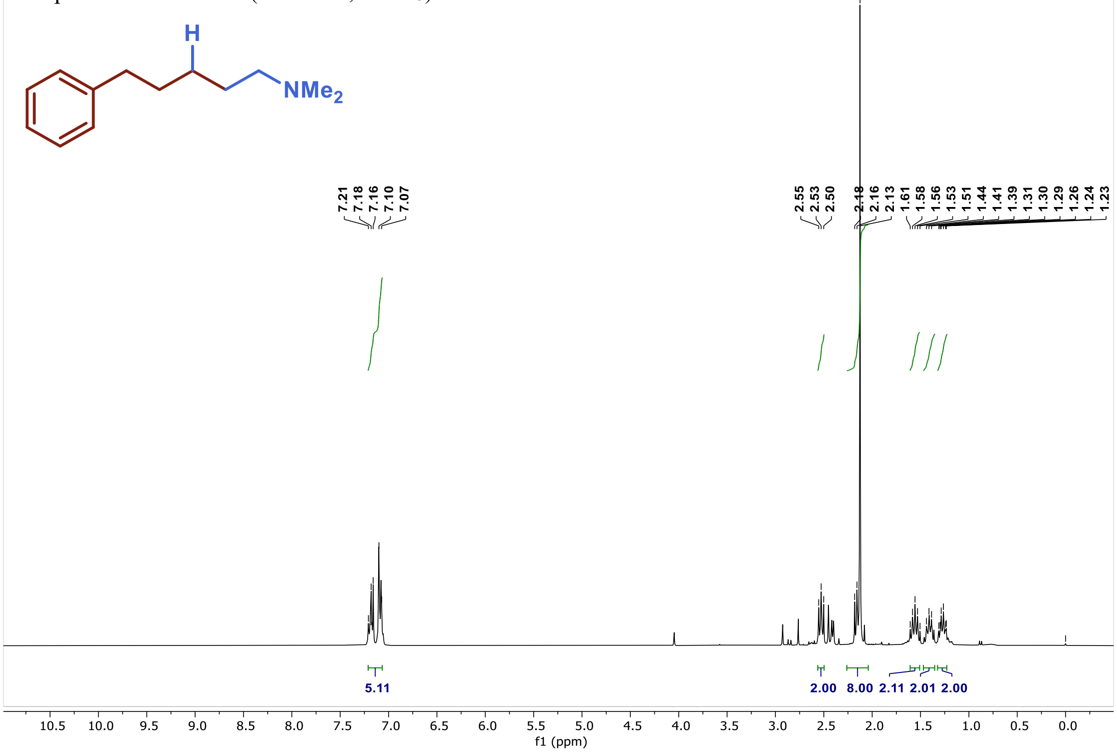

Compound **3a**: ^{13}C NMR (75 MHz, $CDCl_3$)

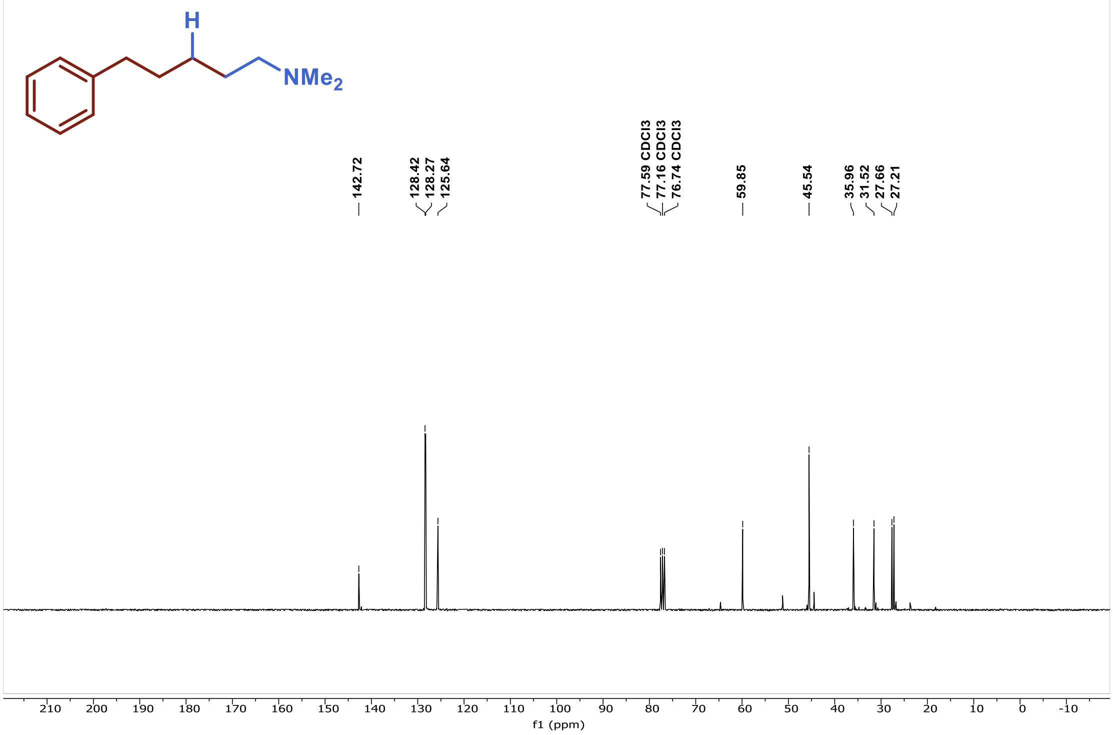

Compound **3c**: ^{1}H NMR (300 MHz, $CDCl_3$)

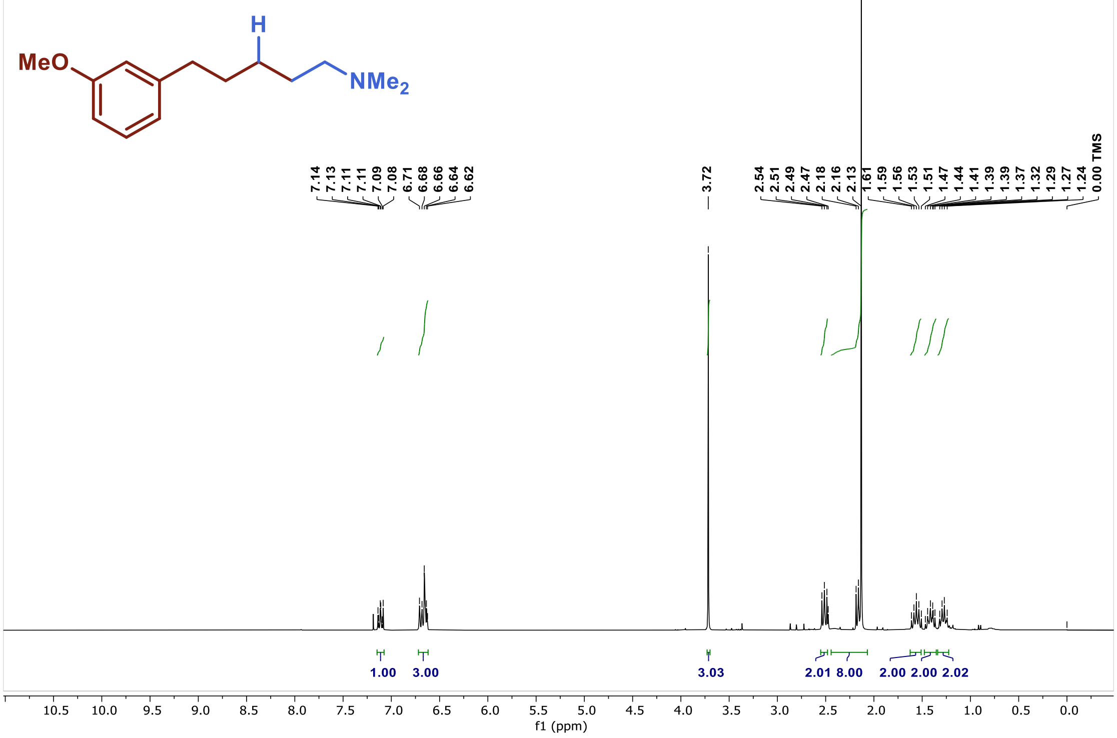

Compound **3c**: ^{13}C NMR (75 MHz, $CDCl_3$)

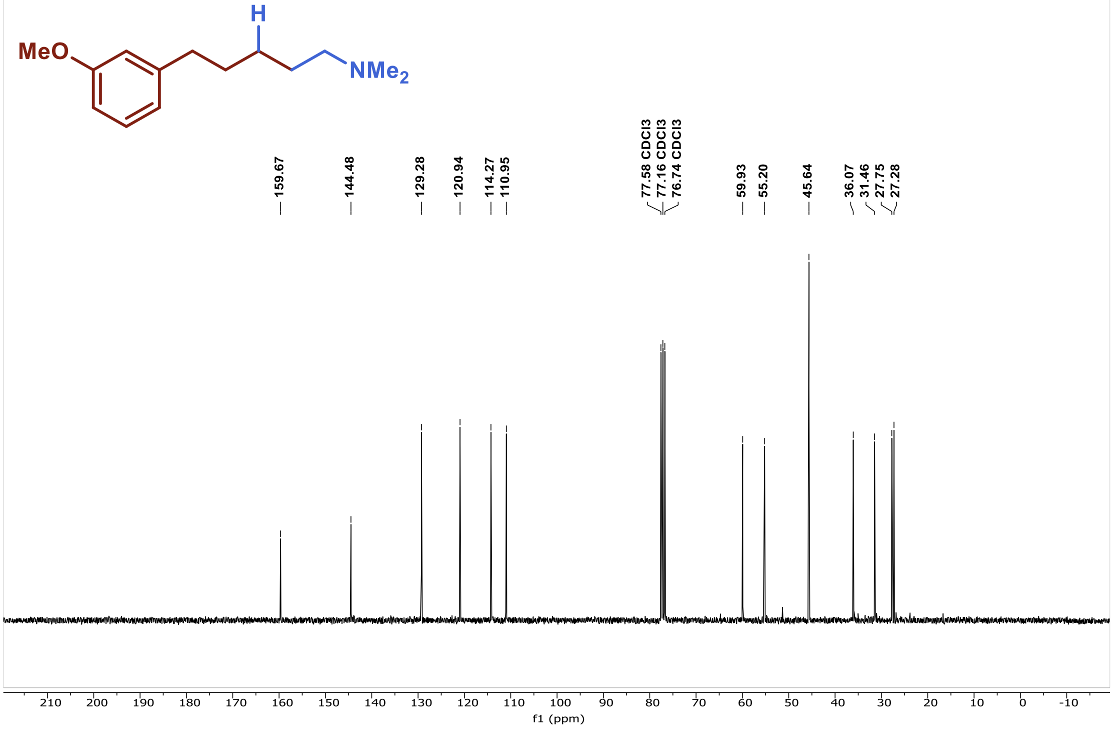

Compound **3d**: ^{1}H NMR (300 MHz, $CDCl_3$)

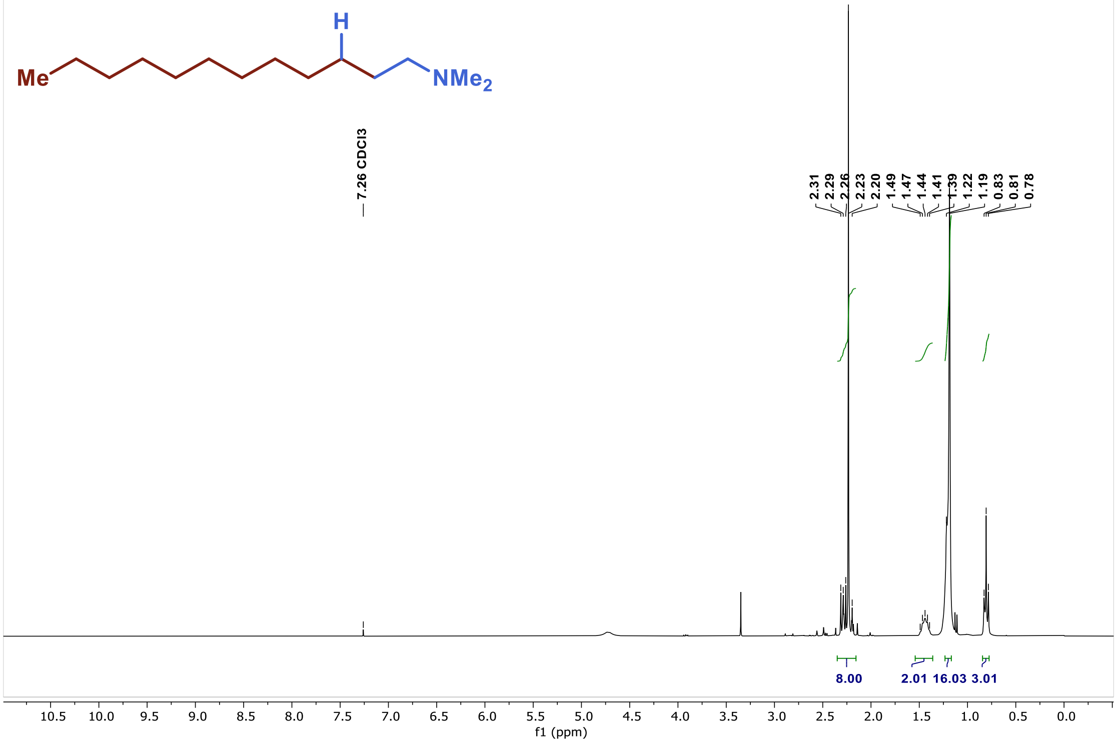

Compound **3d**: ^{13}C NMR (75 MHz, $CDCl_3$)

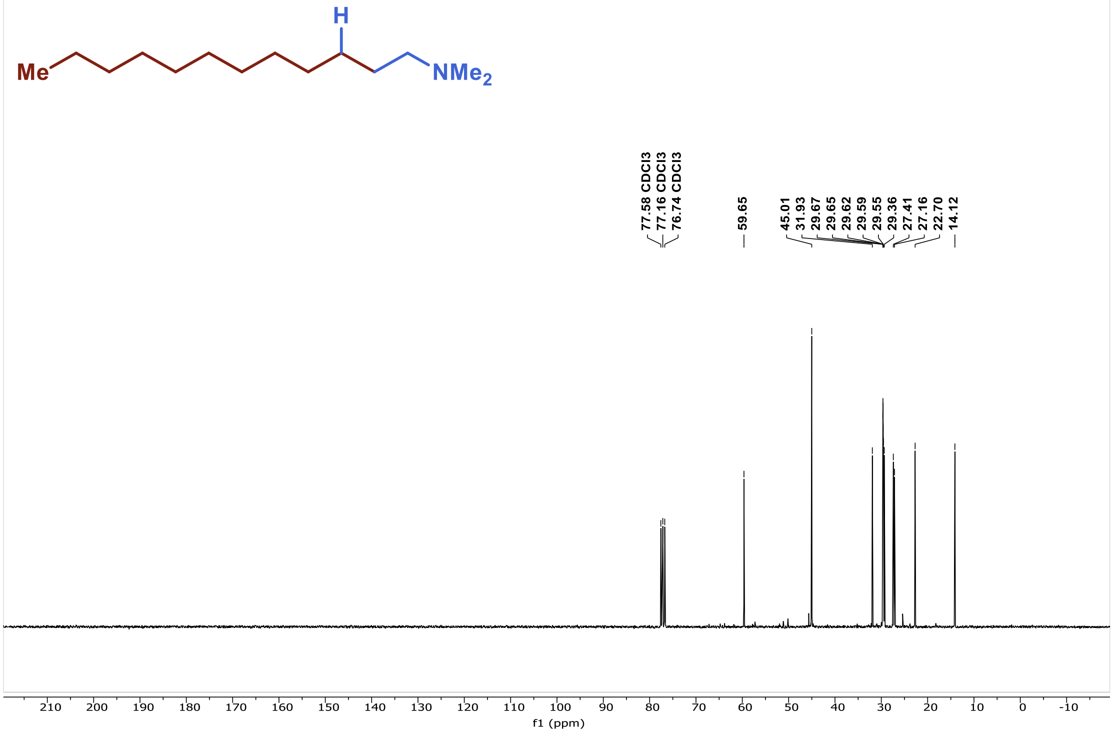

Compound **3e**: ^{1}H NMR (300 MHz, $CDCl_3$)

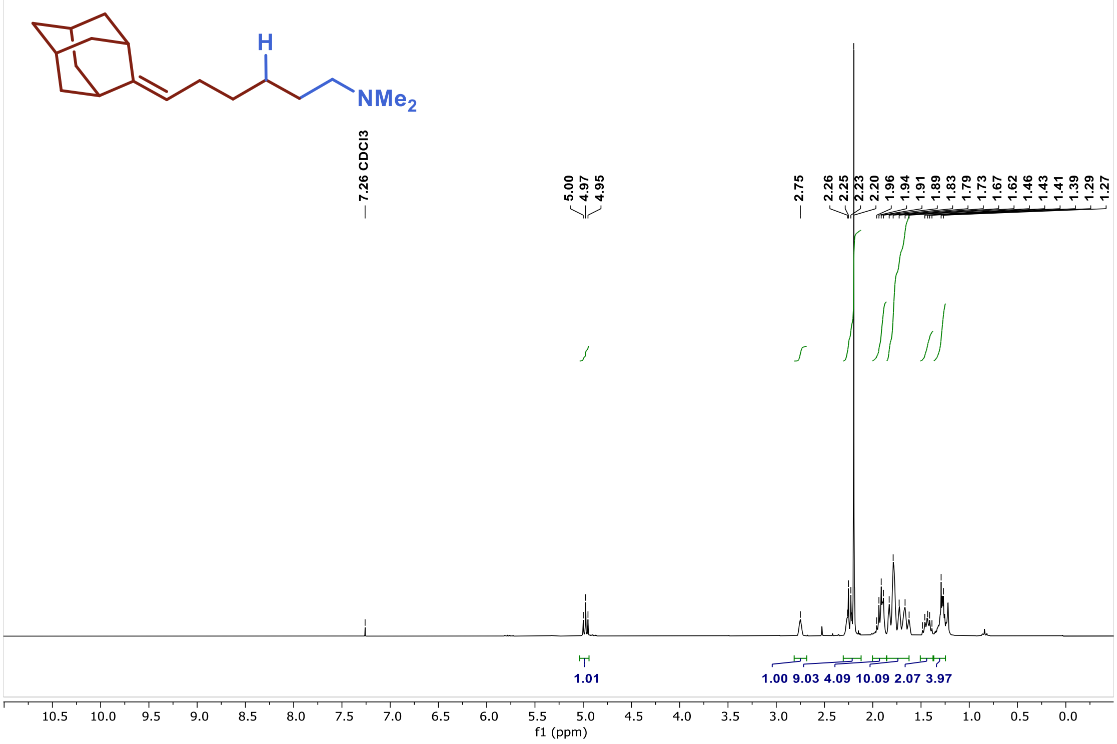

Compound **3e**: ^{13}C NMR (75 MHz, $CDCl_3$)

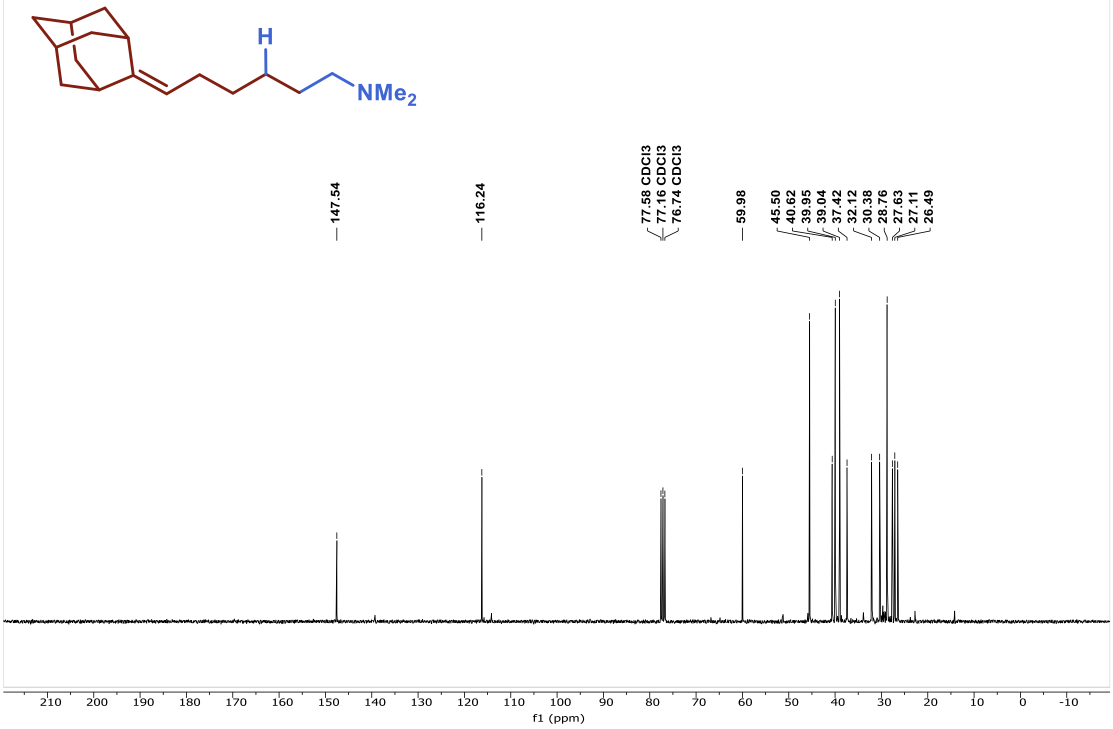

Compound **3h**: ^{1}H NMR (300 MHz, CDCl$_3$)

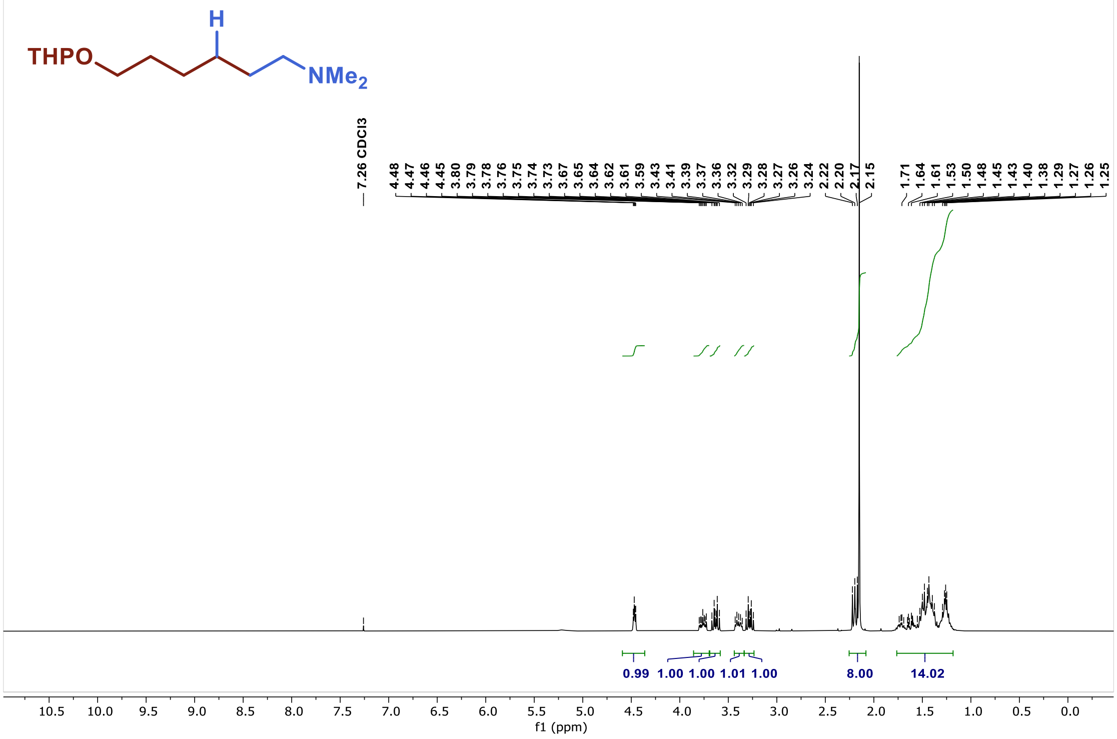

Compound **3h**: ^{13}C NMR (75 MHz, CDCl$_3$)

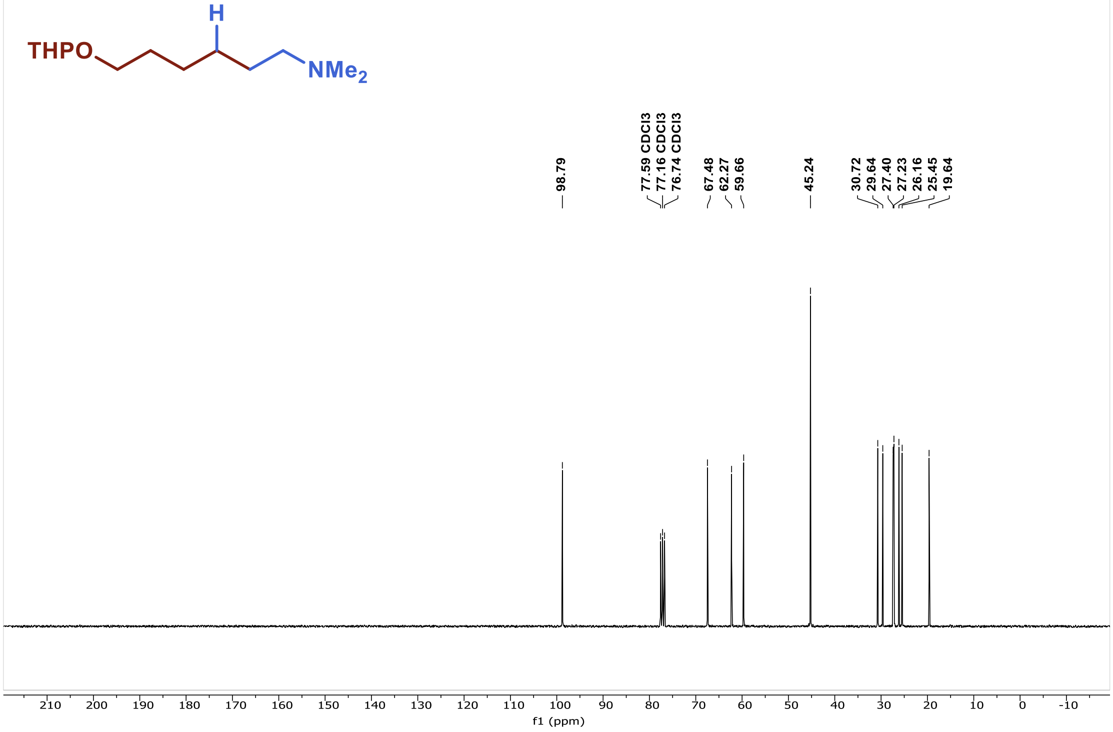

Compound **3o**: ^{1}H NMR (300 MHz, $CDCl_3$)

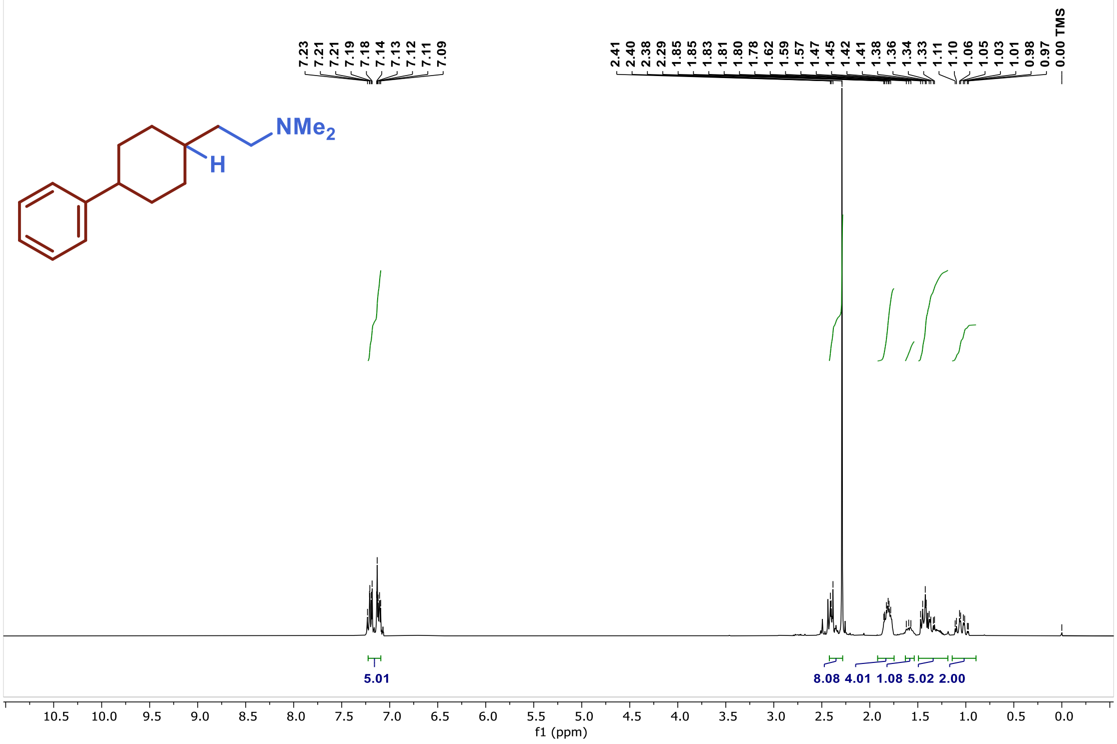

Compound **3o**: ^{13}C NMR (75 MHz, $CDCl_3$)

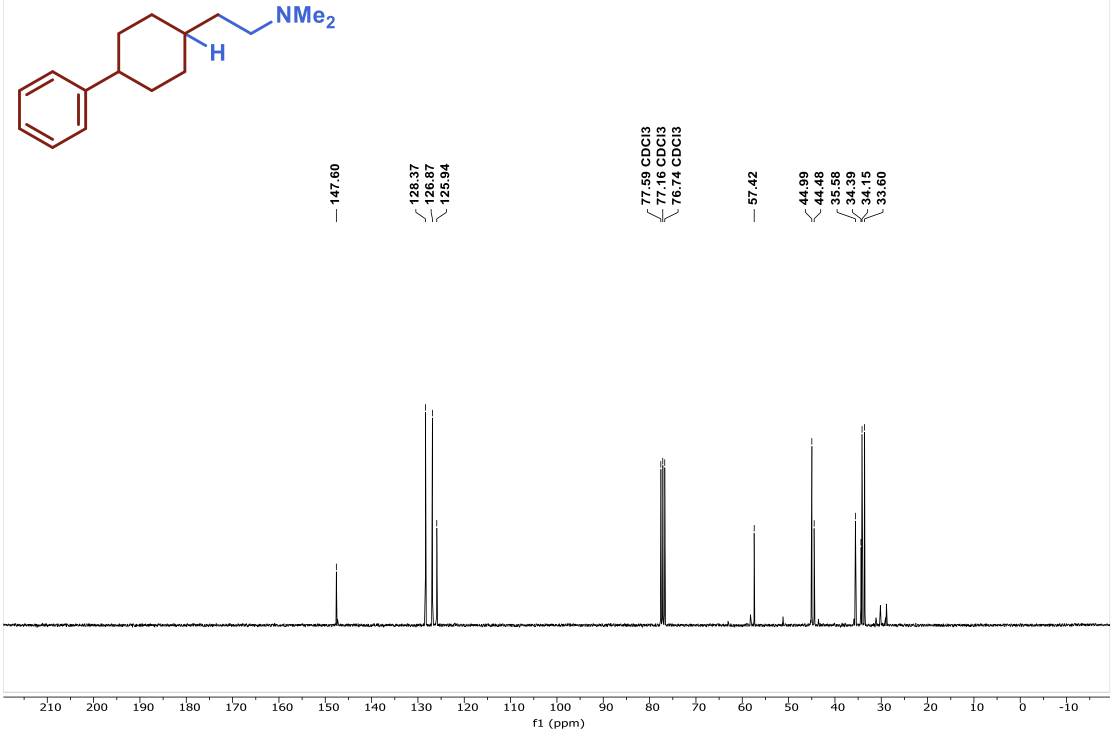

Compound **3p**: ^{1}H NMR (300 MHz, $CDCl_3$)

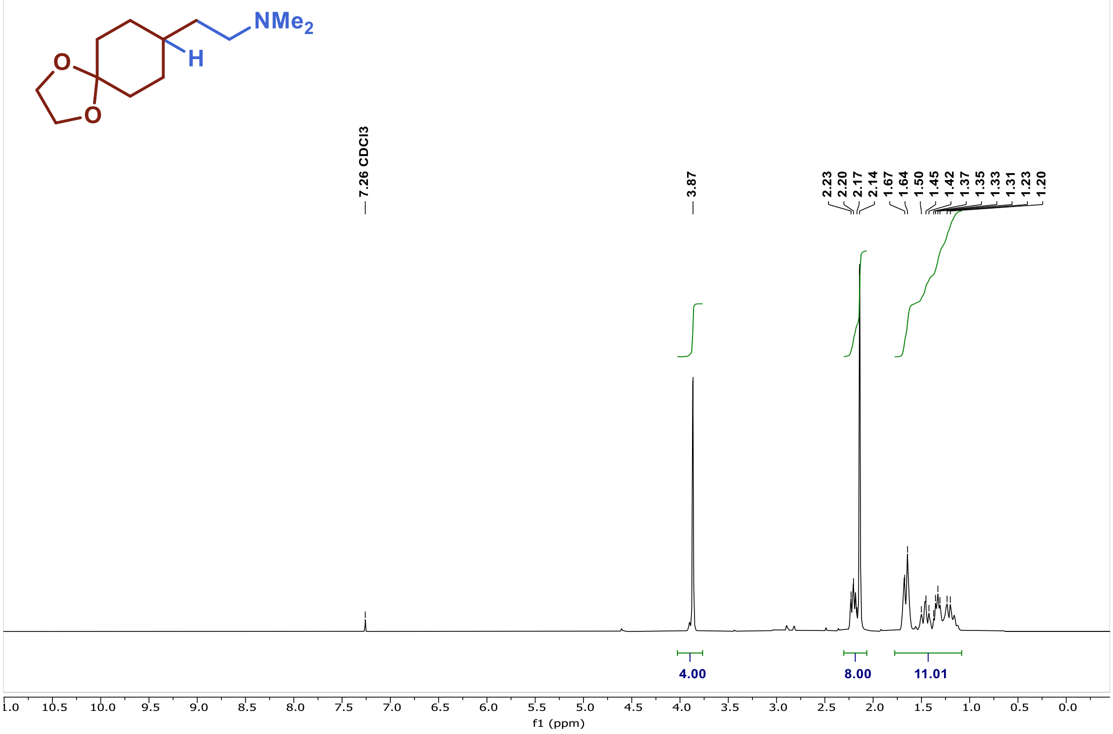

Compound **3p**: ^{13}C NMR (75 MHz, $CDCl_3$)

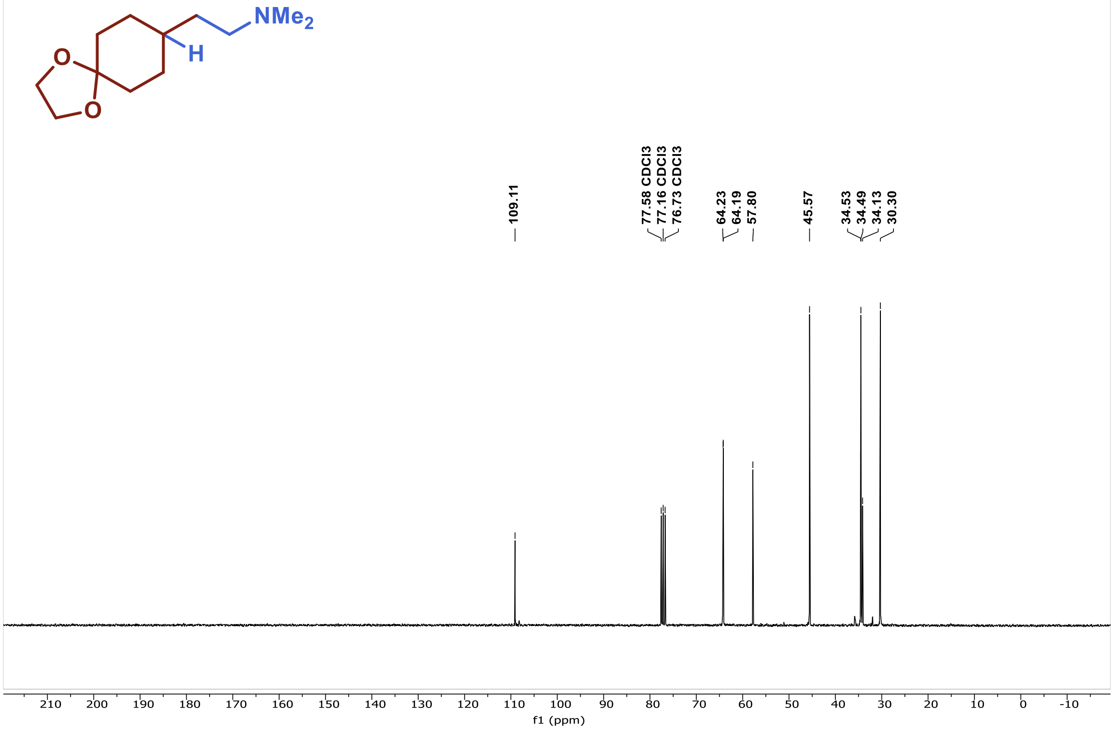

Compound **3t**: ^{1}H NMR (300 MHz, $CDCl_3$)

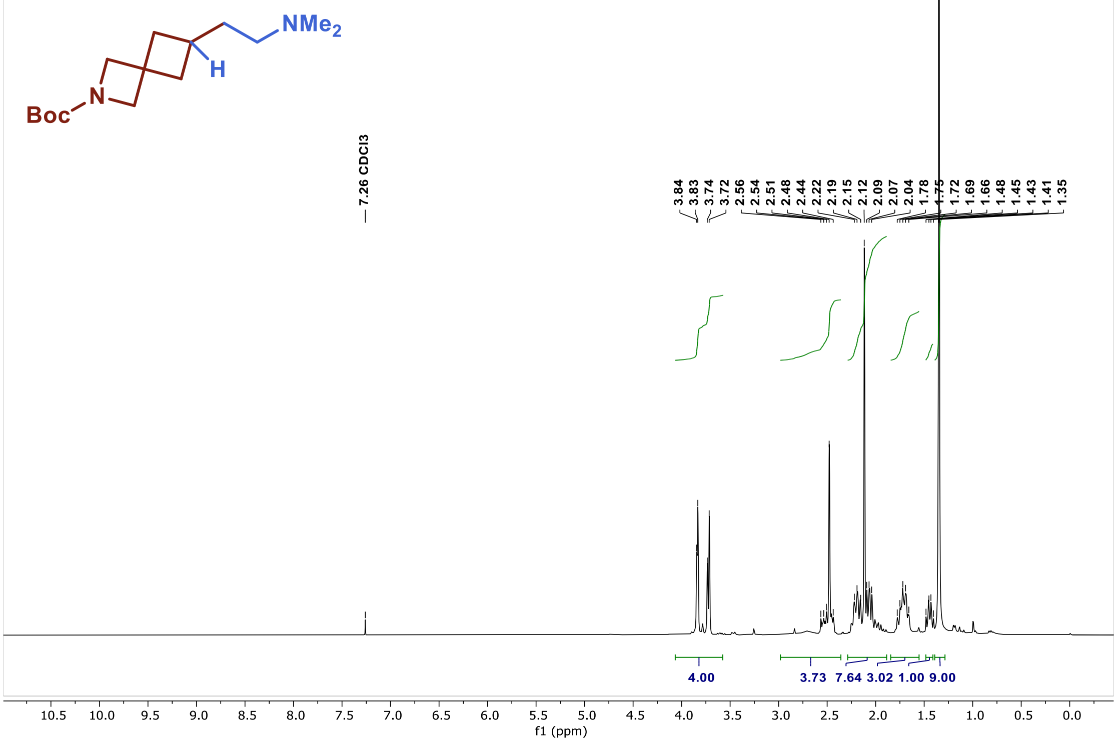

Compound **3t**: ^{13}C NMR (75 MHz, $CDCl_3$)

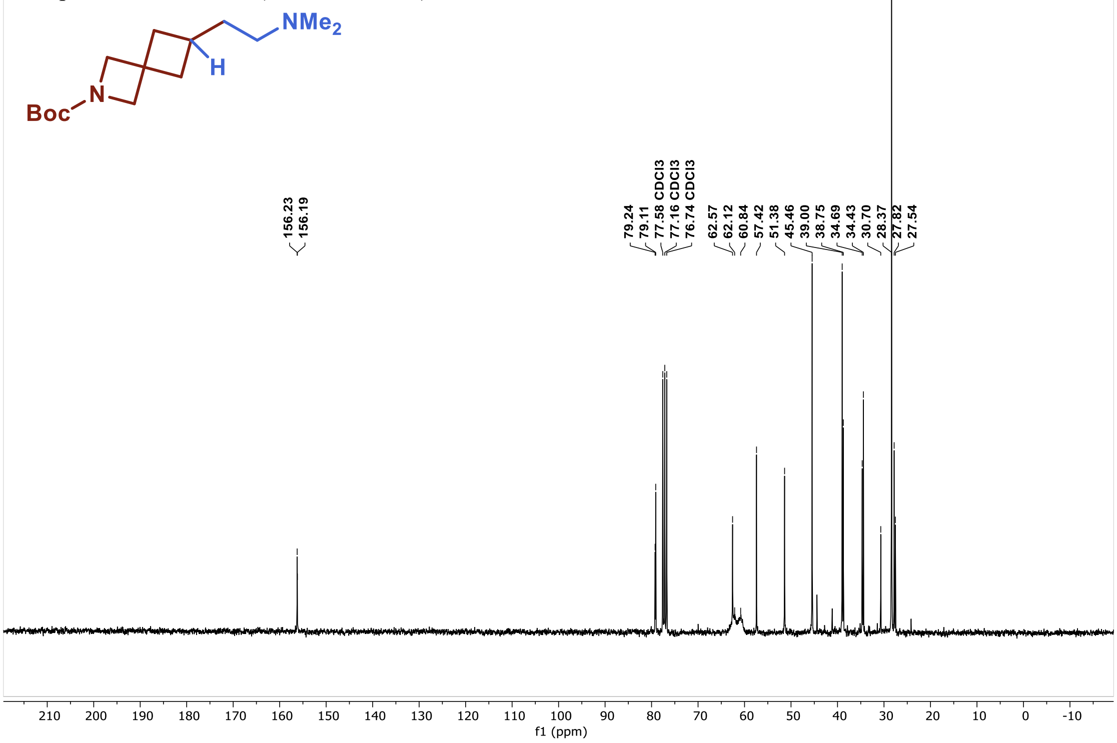

Compound **3u**: ^{1}H NMR (300 MHz, $CDCl_3$)

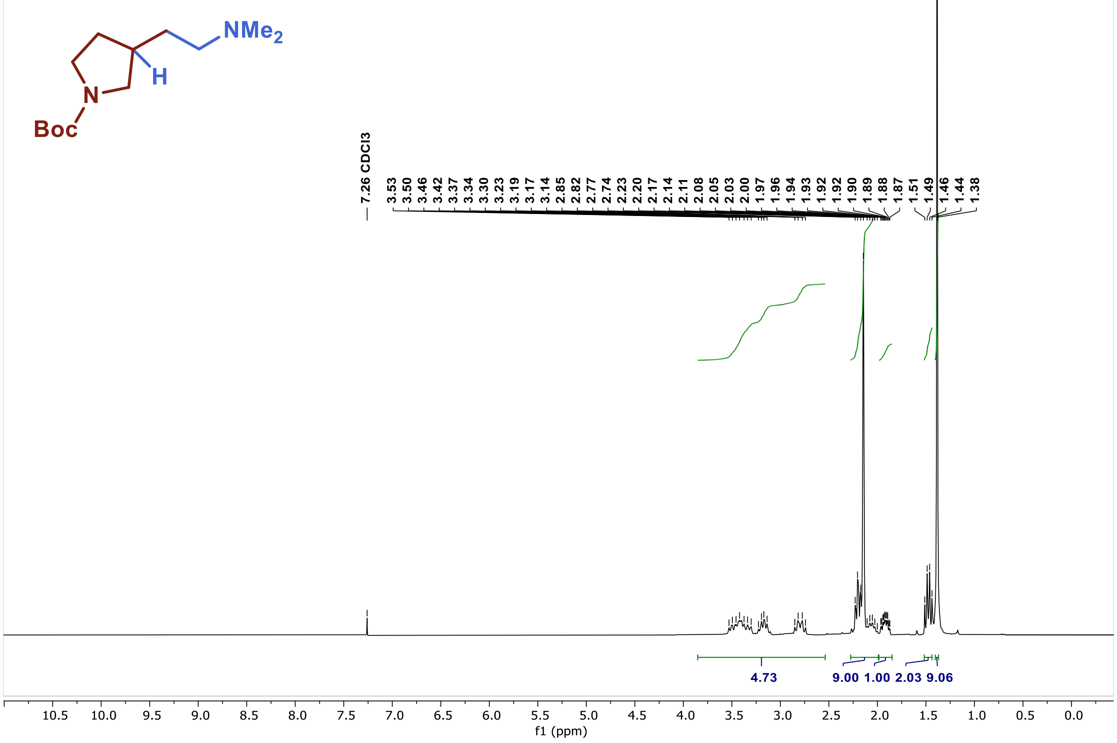

Compound **3u**: ^{13}C NMR (75 MHz, $CDCl_3$)

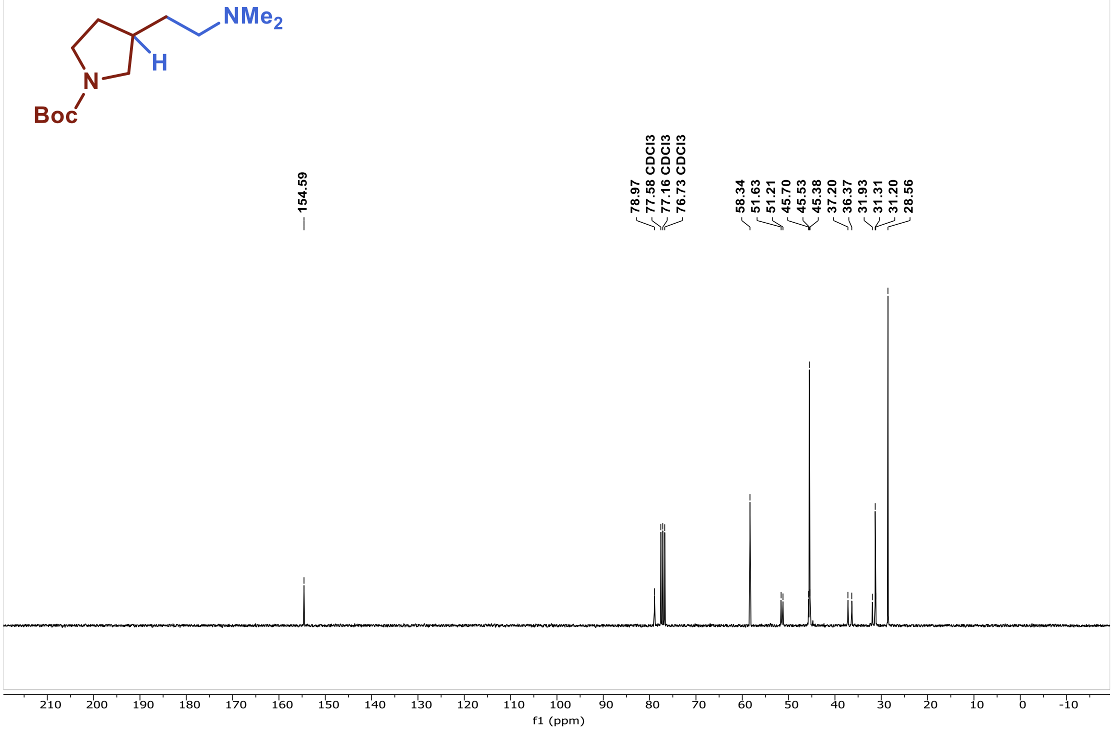

Compound **3v**: ^{1}H NMR (300 MHz, $CDCl_3$)

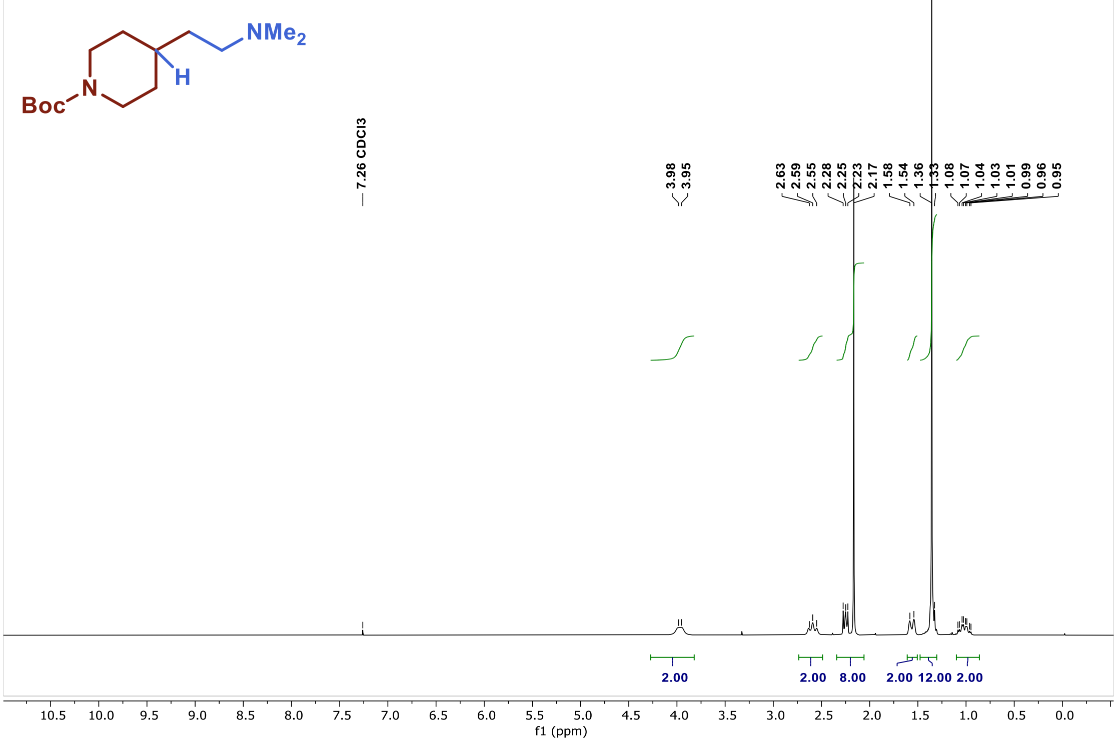

Compound **3v**: ^{13}C NMR (75 MHz, $CDCl_3$)

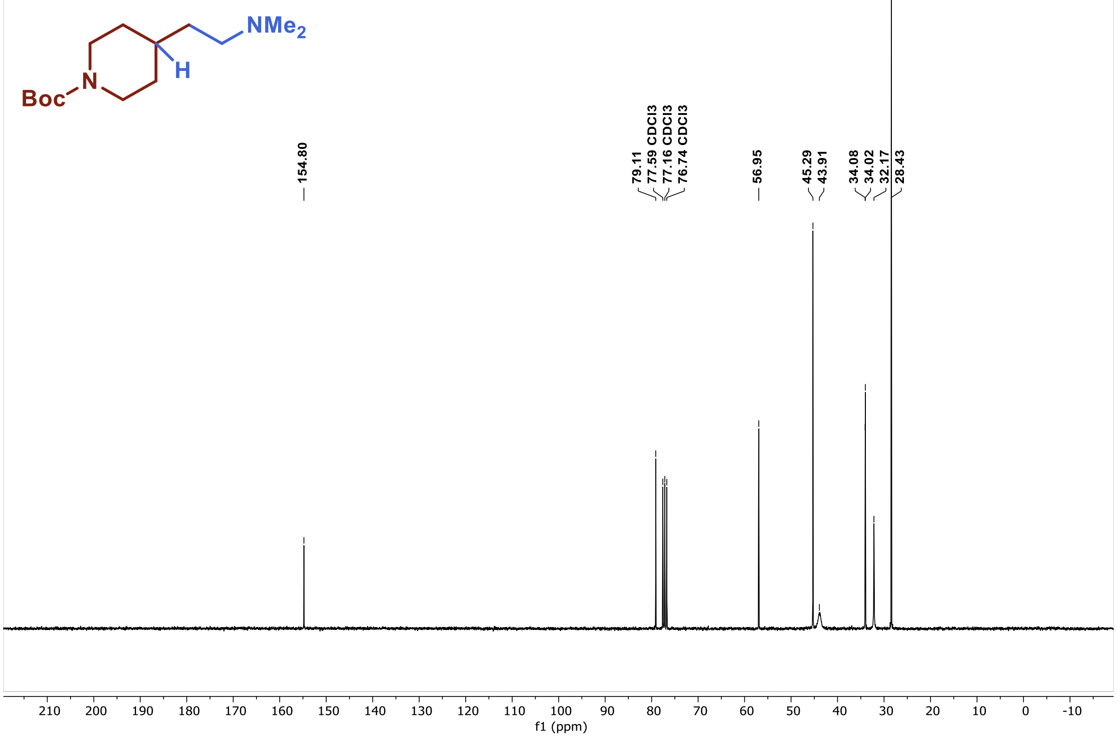

Compound **3w**: ^{1}H NMR (300 MHz, $CDCl_3$)

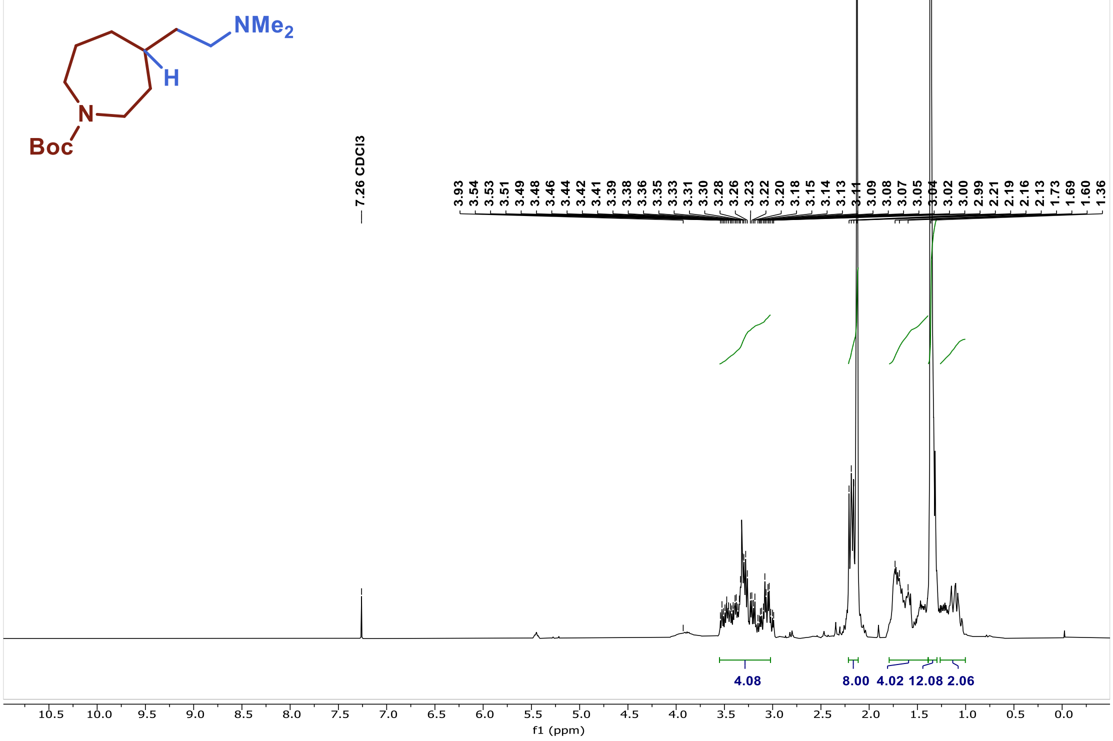

Compound **3w**: ^{13}C NMR (75 MHz, $CDCl_3$)

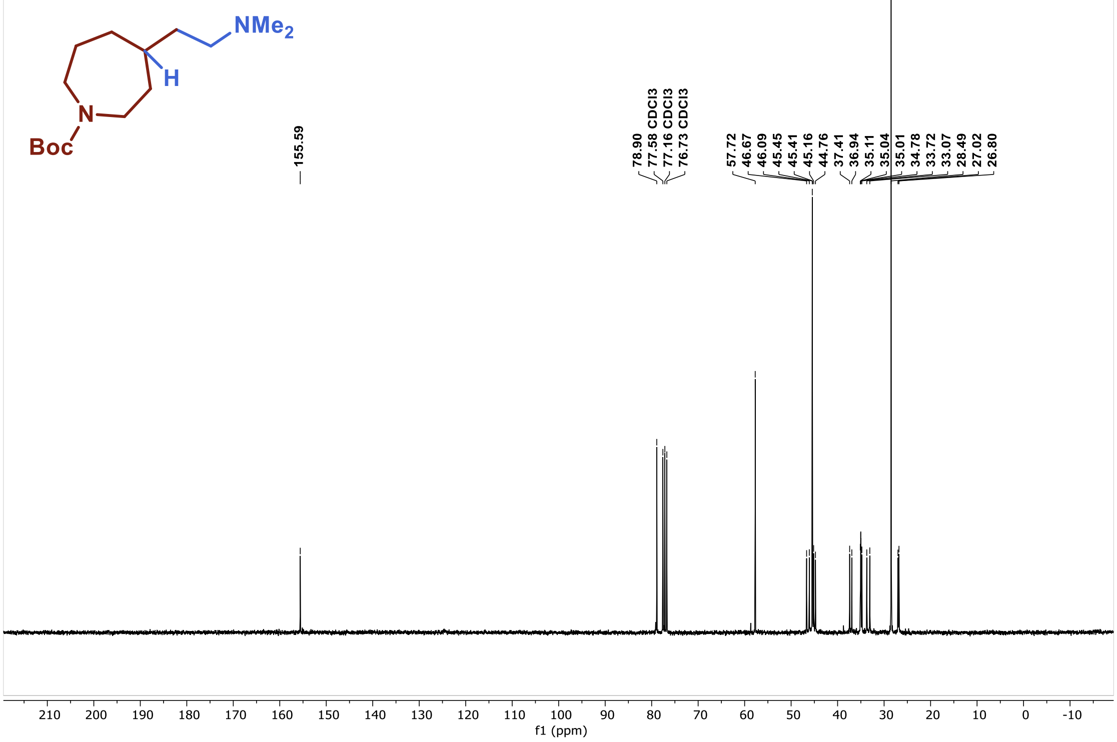

Compound **3x**: ^{1}H NMR (300 MHz, $CDCl_3$)

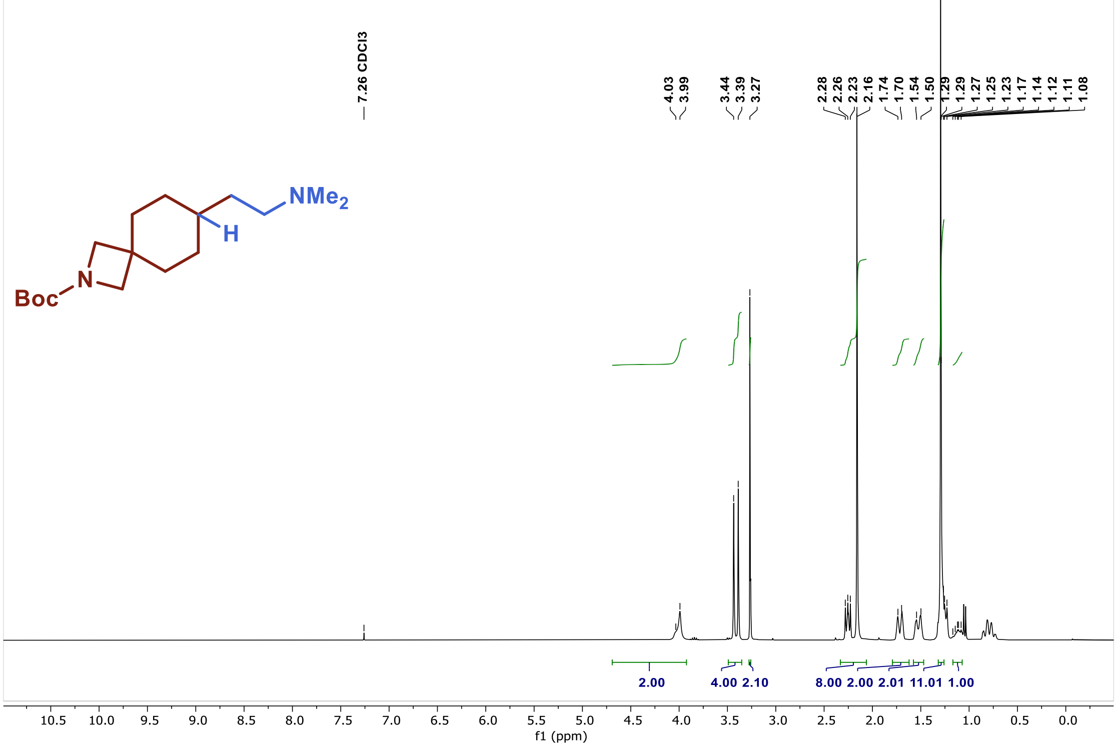

Compound **3x**: ^{13}C NMR (75 MHz, $CDCl_3$)

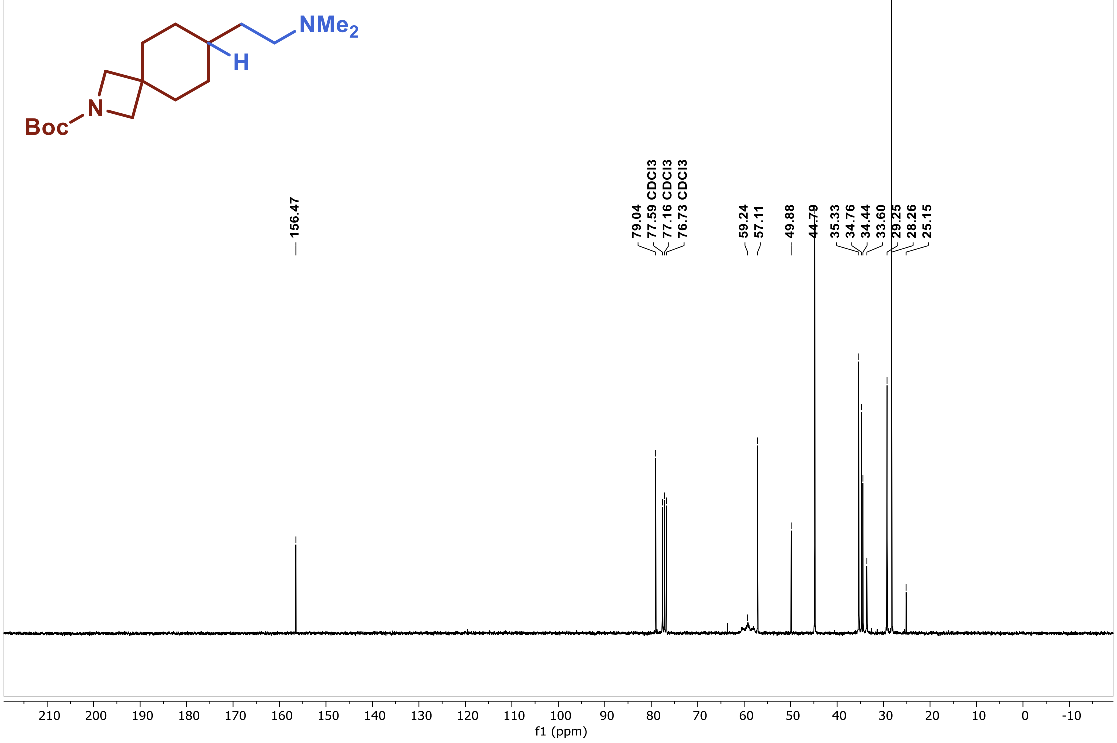

Using DMA as solvent

Compound **4a**: ^{1}H NMR (300 MHz, $CDCl_3$)

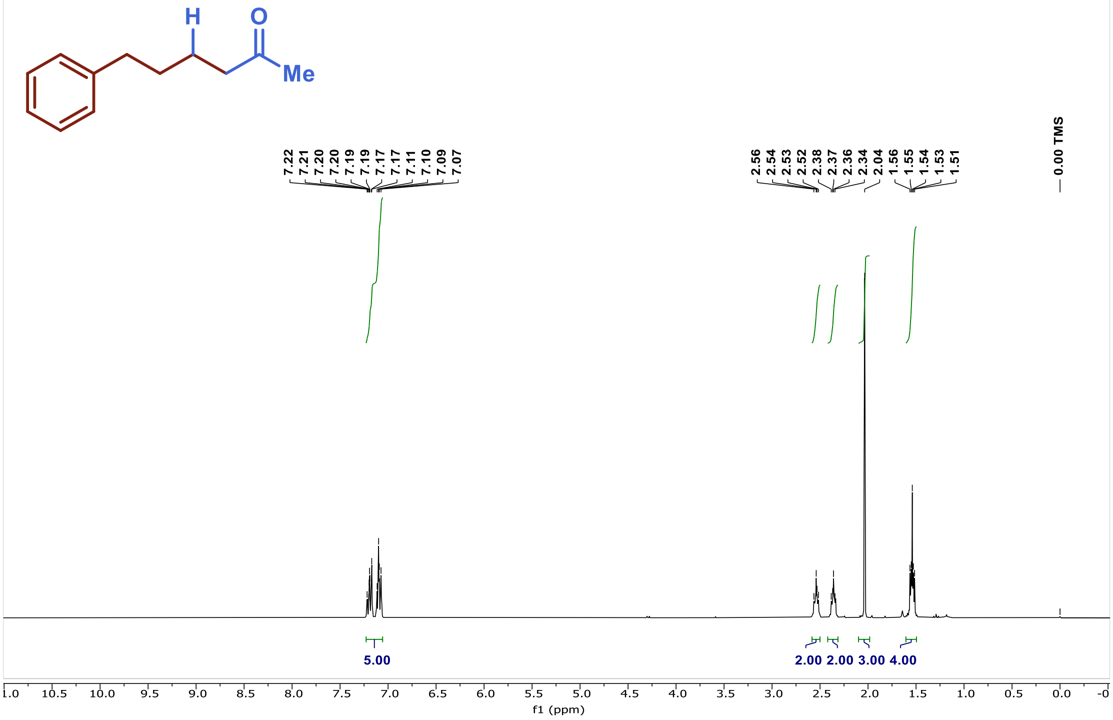

Compound **4a**: ^{13}C NMR (75 MHz, $CDCl_3$)

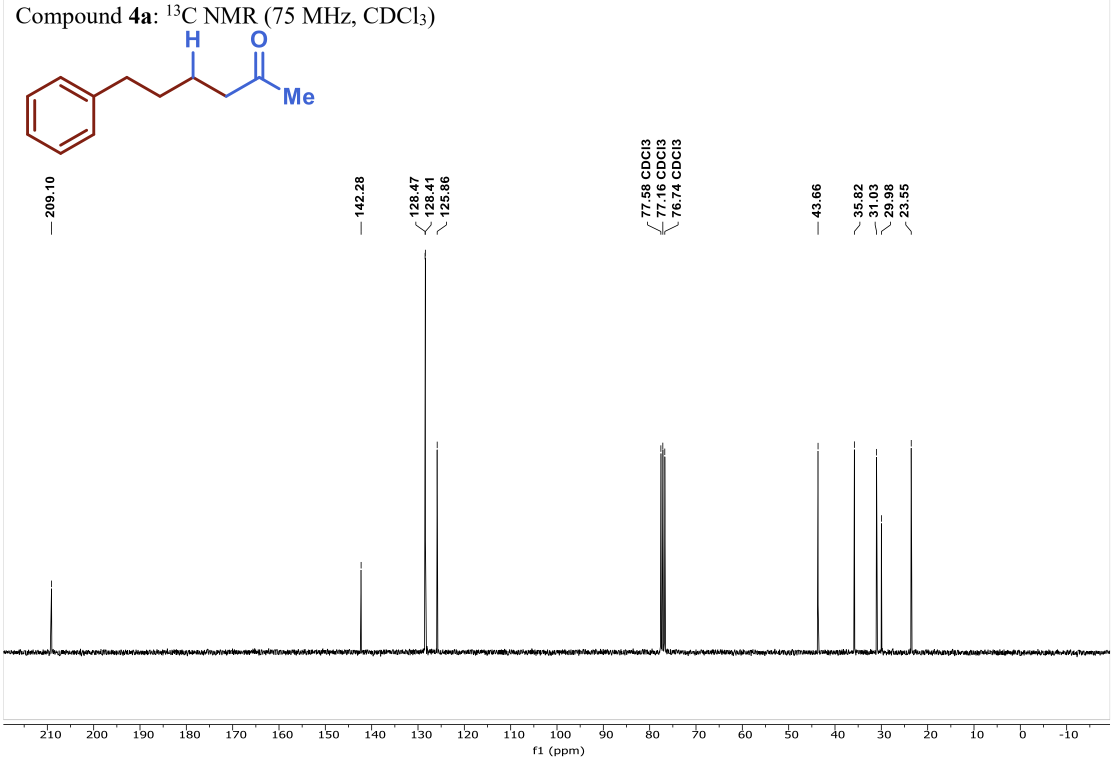

Compound **4d**: ¹H NMR (300 MHz, $CDCl_3$)

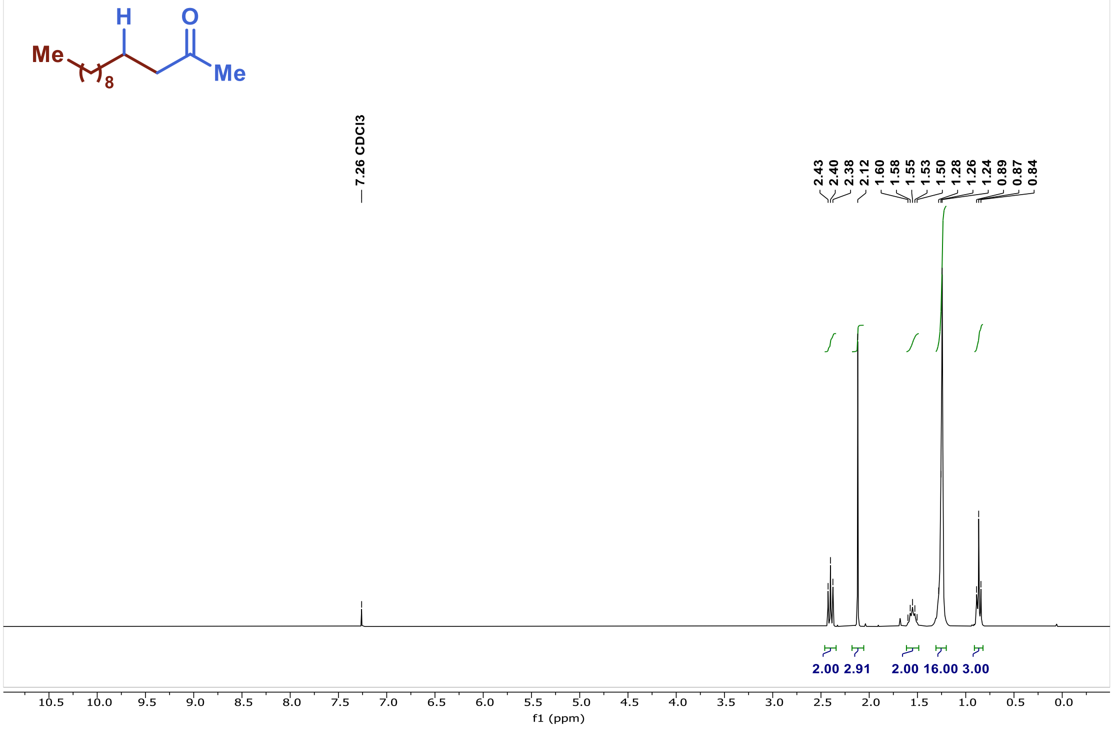

Compound **4d**: ¹³C NMR (75 MHz, $CDCl_3$)

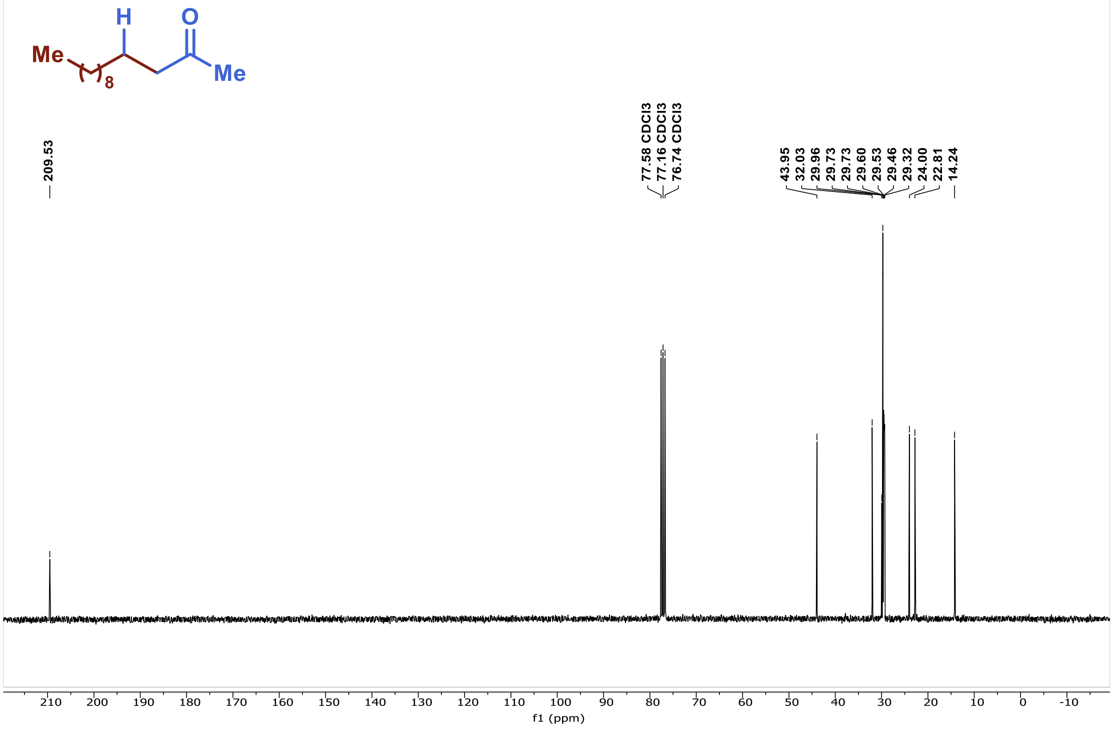

Homologation synthesis

Compound **5**: ^{1}H NMR (300 MHz, $CDCl_3$)

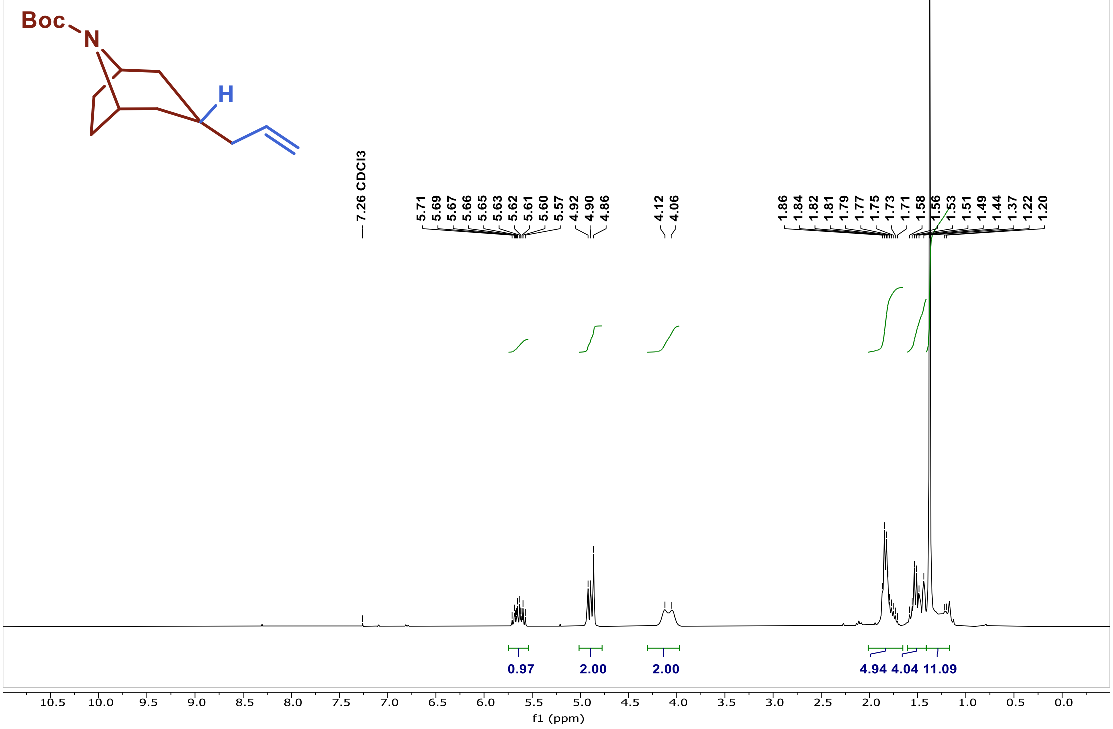

Compound **5**: ^{13}C NMR (75 MHz, $CDCl_3$)

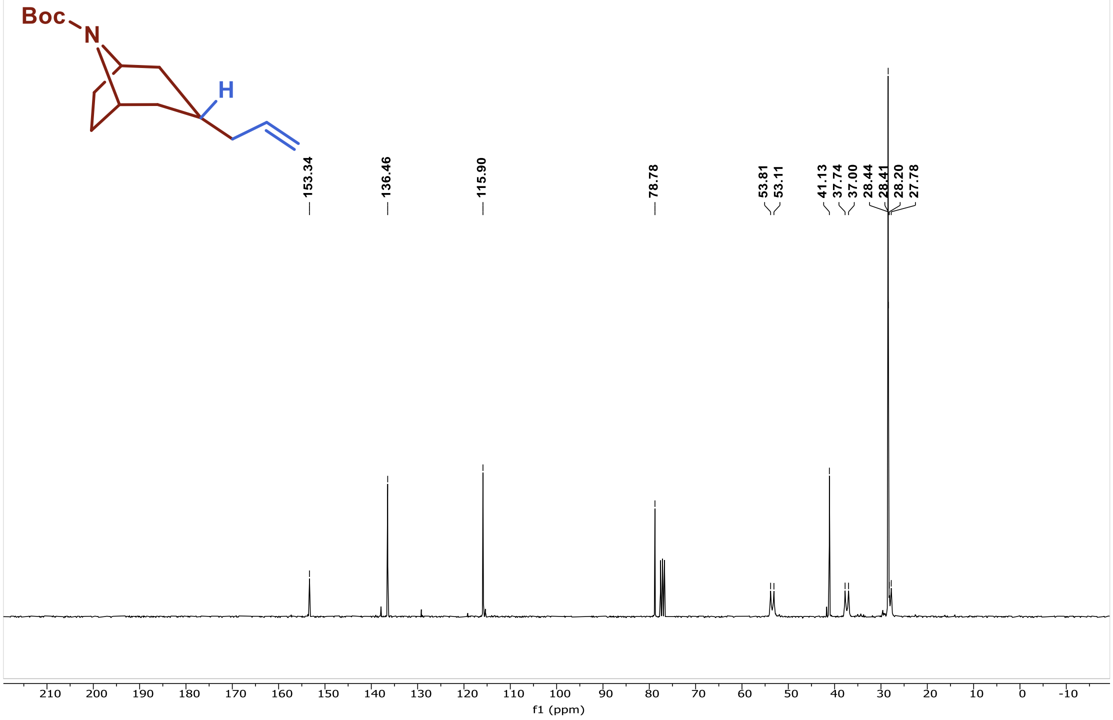

Compound **2z**: ^{1}H NMR (300 MHz, $CDCl_3$)

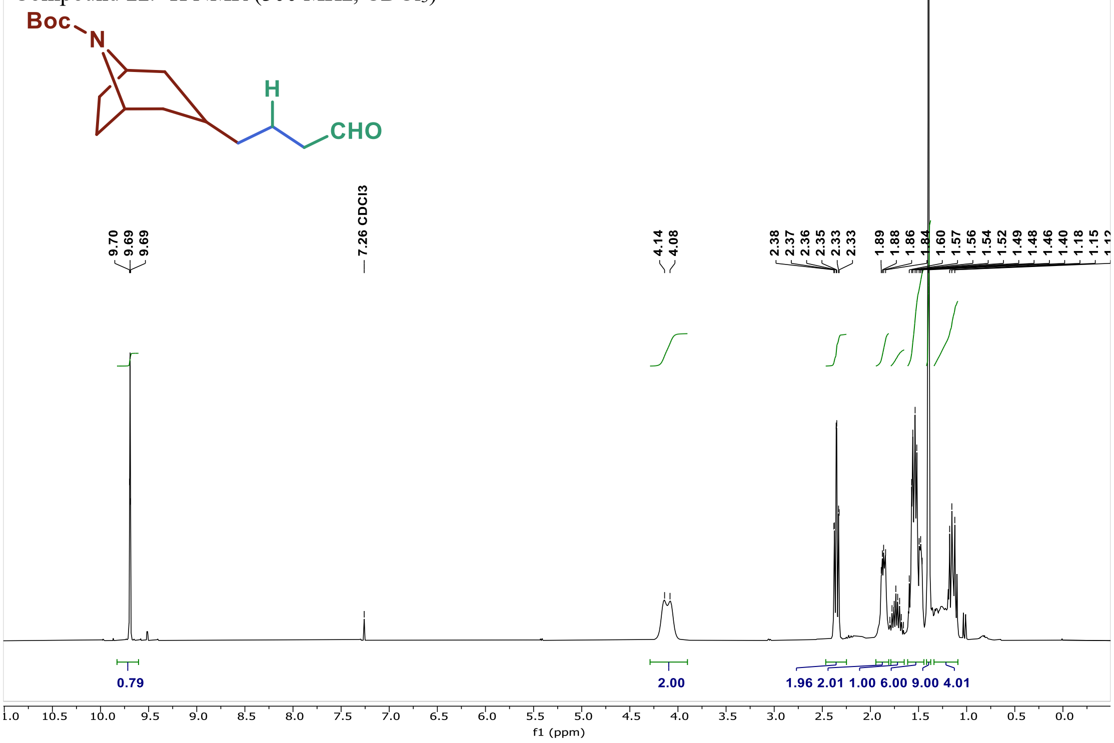

Compound **2z**: ^{13}C NMR (75 MHz, $CDCl_3$)

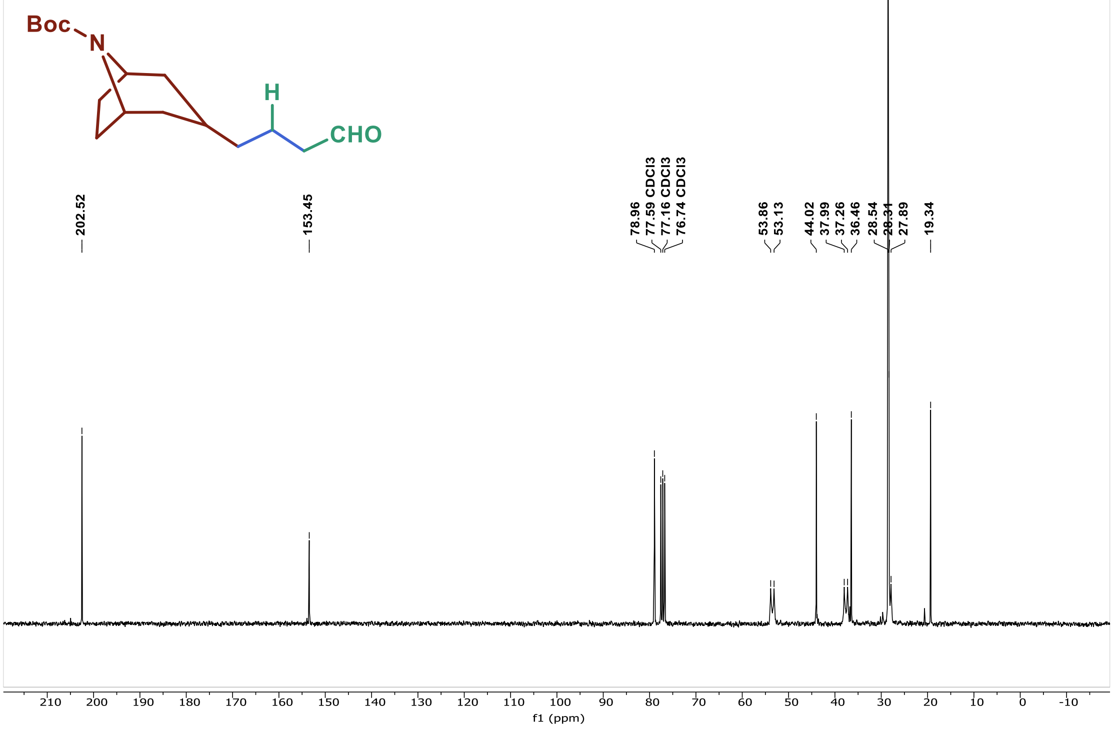

Compounds for mechanism

Compound **2ad'**: ^{1}H NMR (300 MHz, $CDCl_3$)

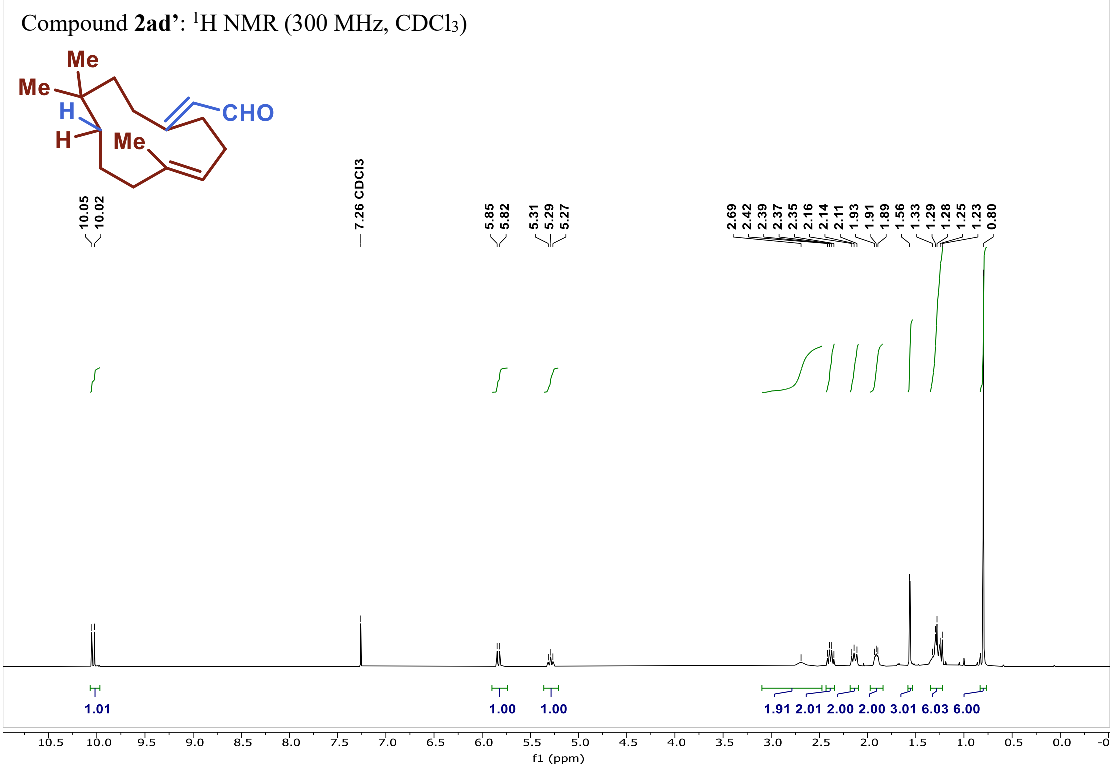

Compound **2ad'**: ^{13}C NMR (75 MHz, $CDCl_3$)

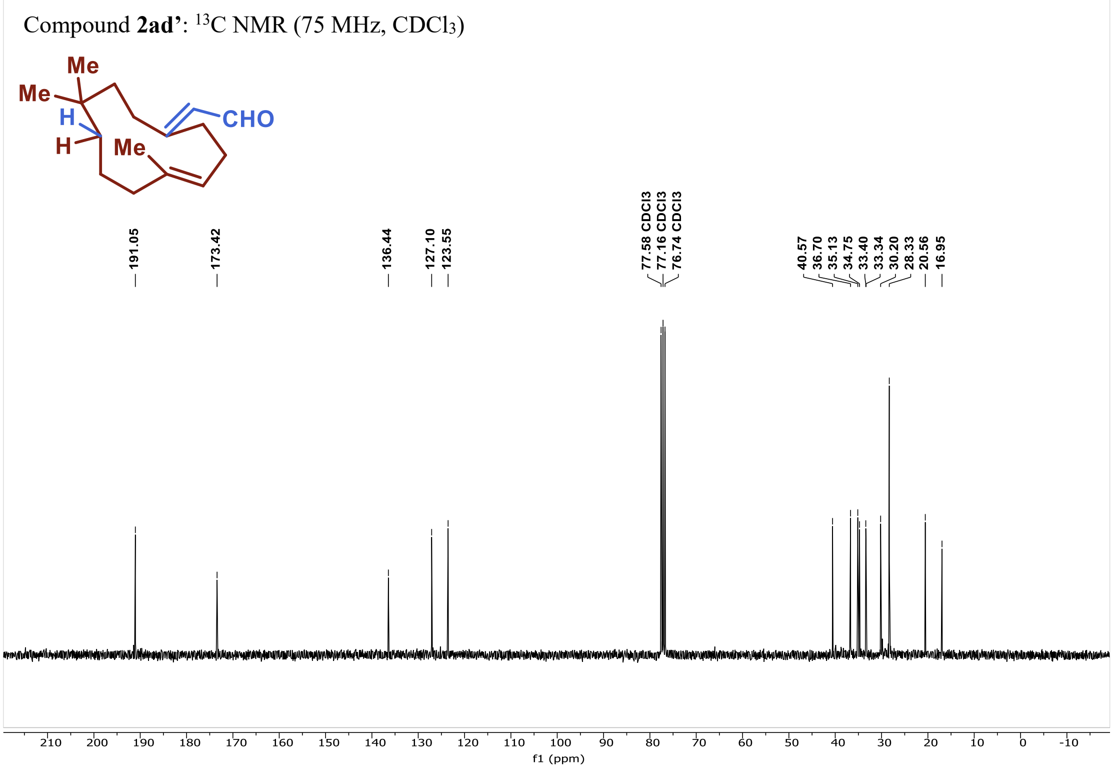

Compound **2ad'**: DEPT 135

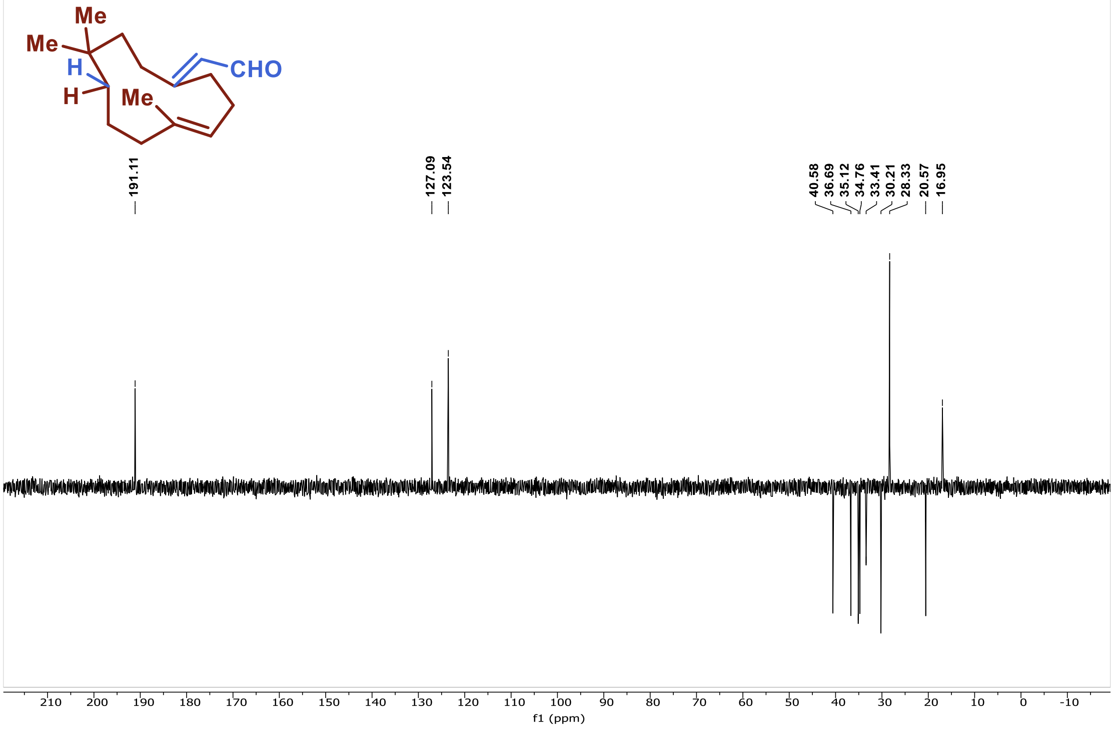

Compound **2ad'**: COSY

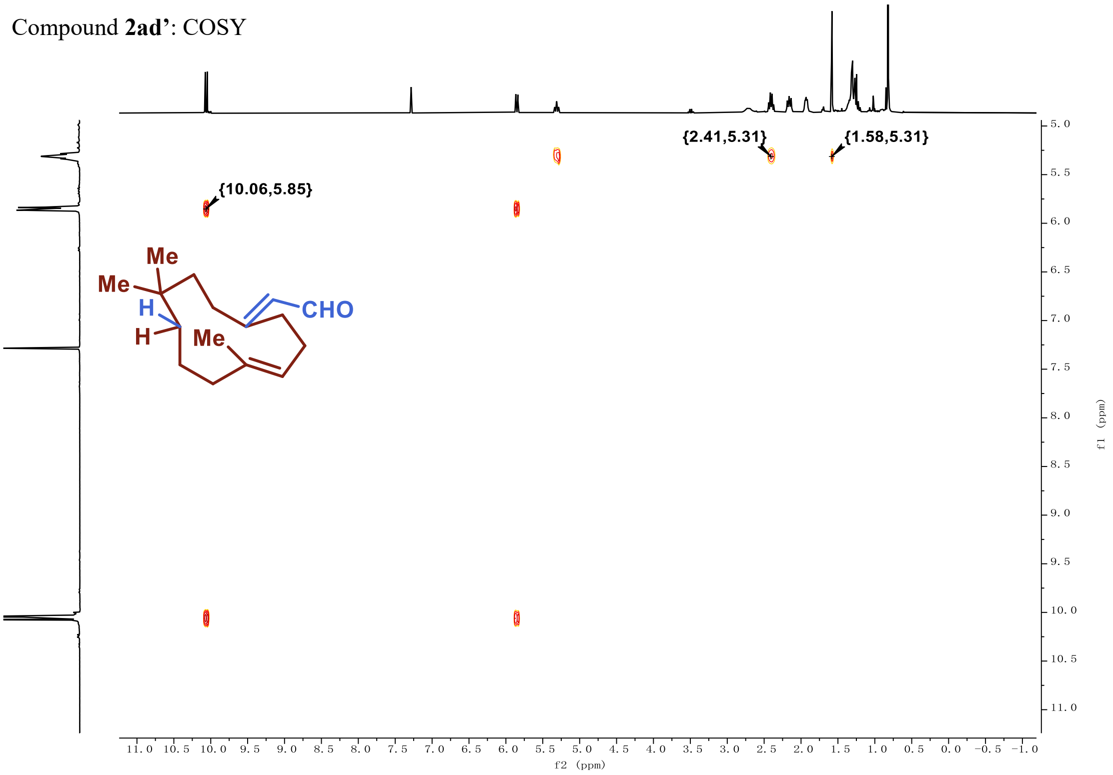

Compound **2ad'**: HSQC

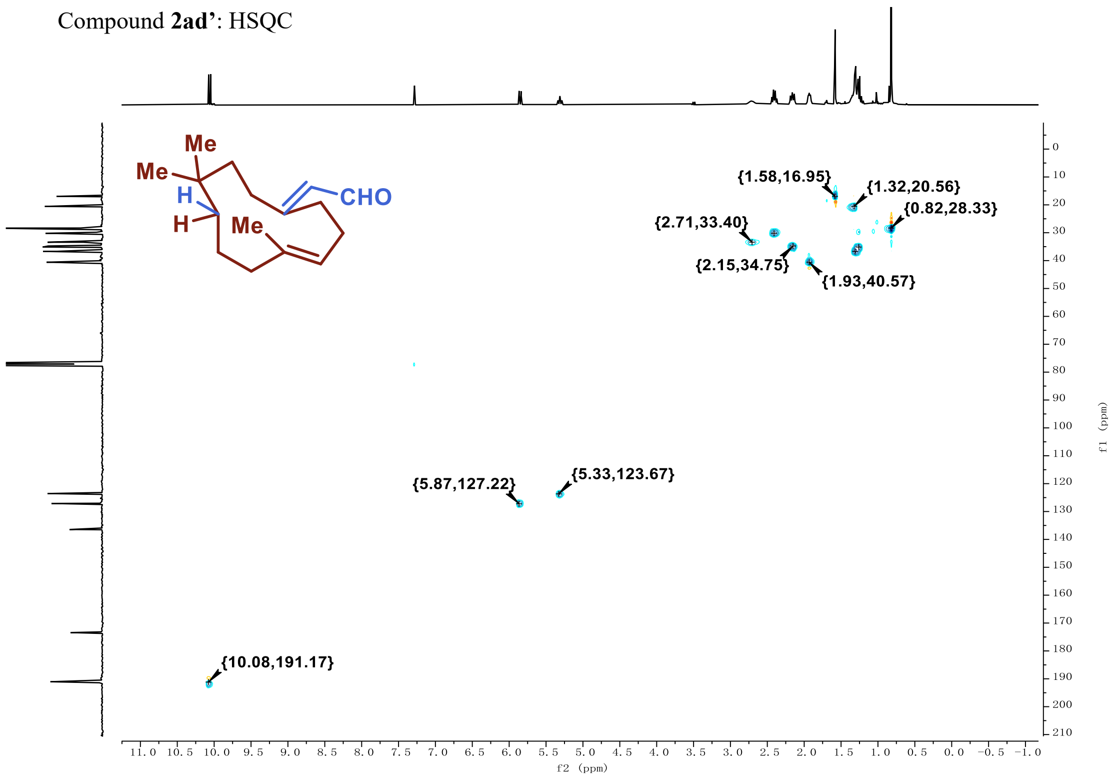

Compound **2ad'**: HMBC

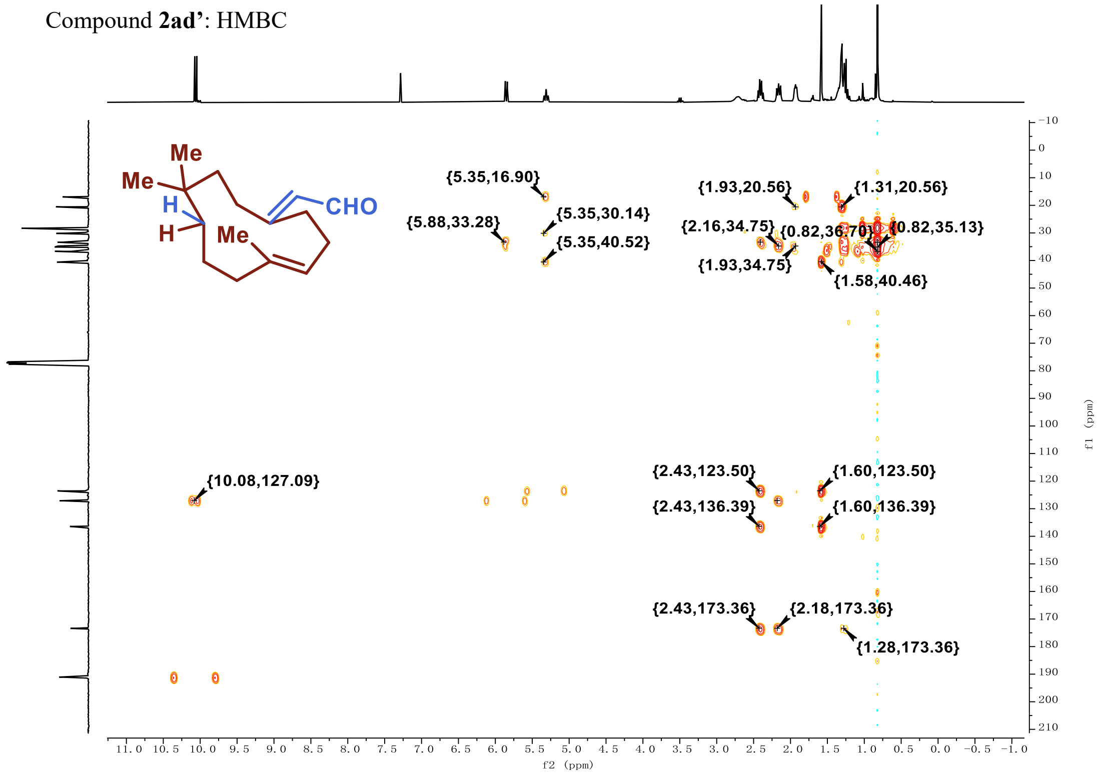

Compound **2ad'**: NOESY

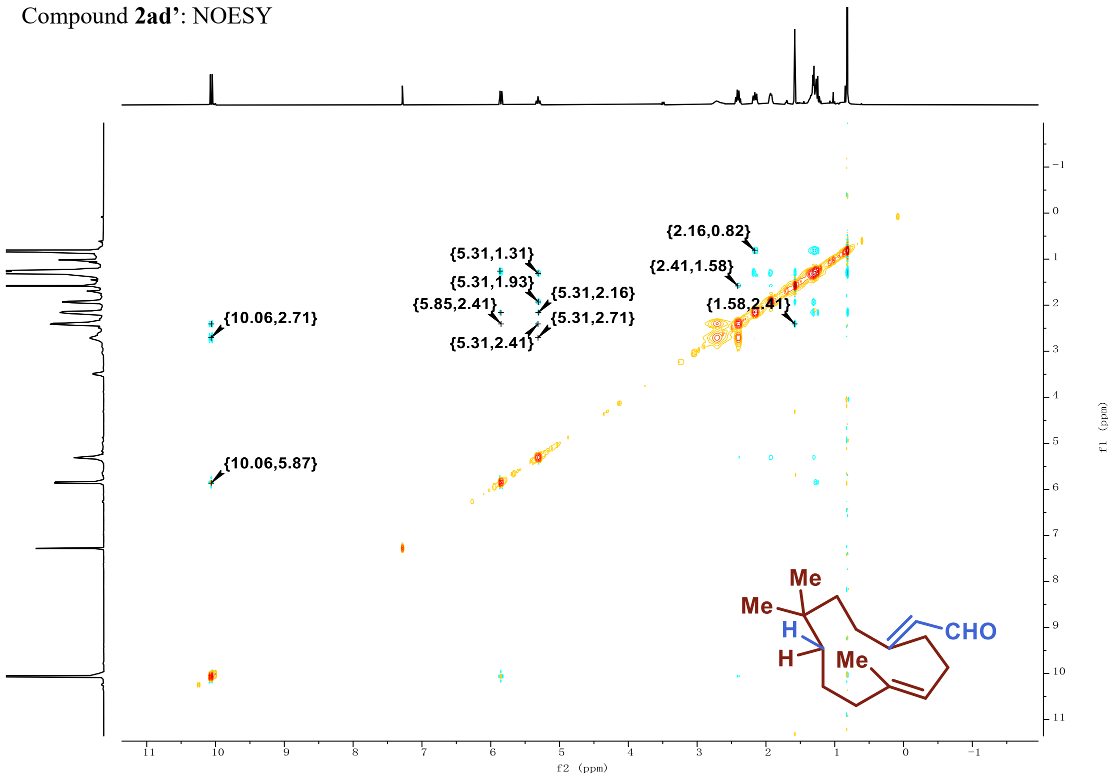

Compound **6a**: ^{1}H NMR (300 MHz, DMSO-d_6)

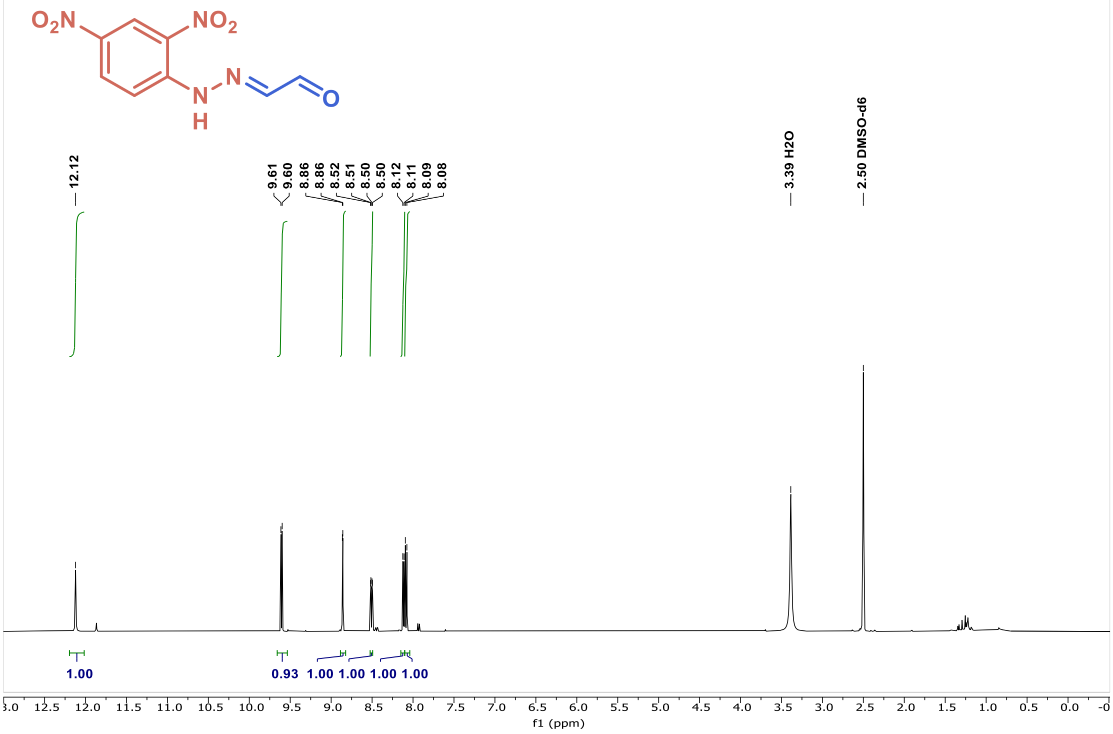

Compound **6a**: ^{13}C NMR (75 MHz, DMSO-d_6)

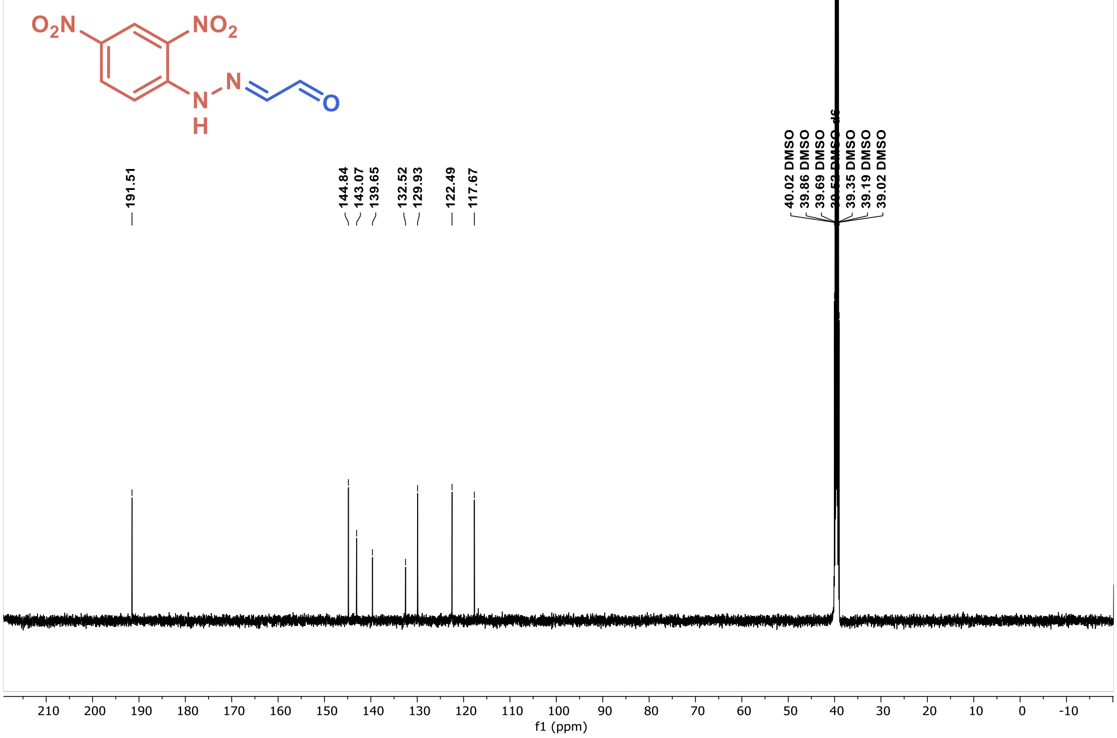

Compound **6b**: ^{1}H NMR (300 MHz, DMSO-d_6)

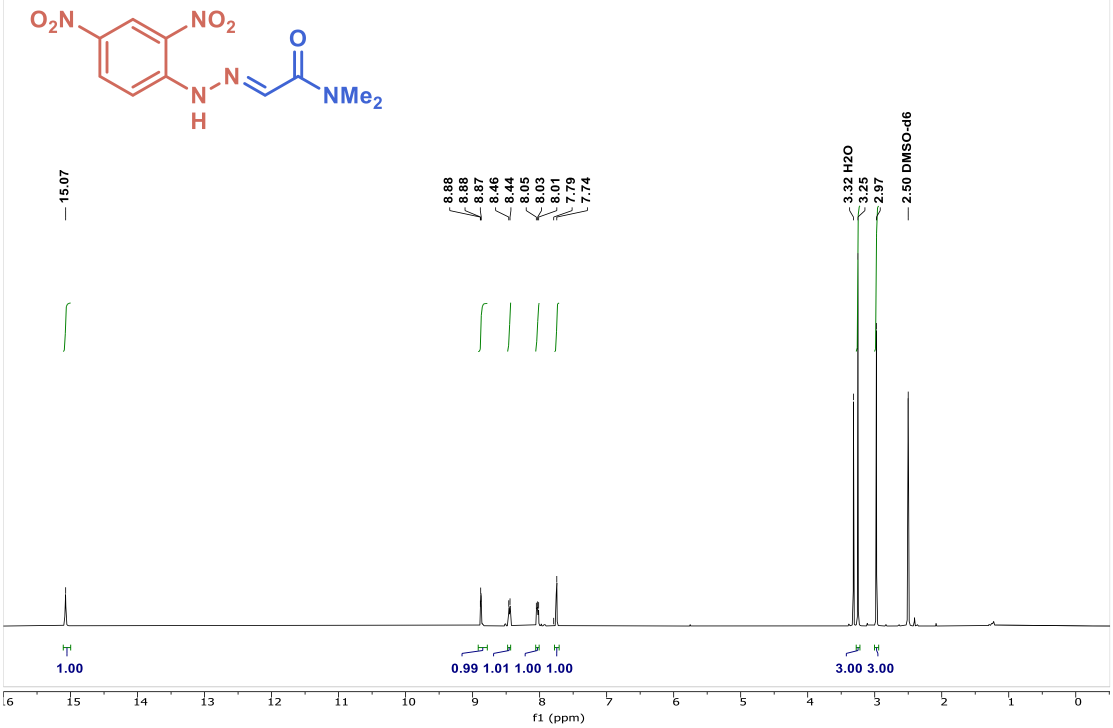

Compound **6b**: ^{13}C NMR (75 MHz, DMSO-d_6)

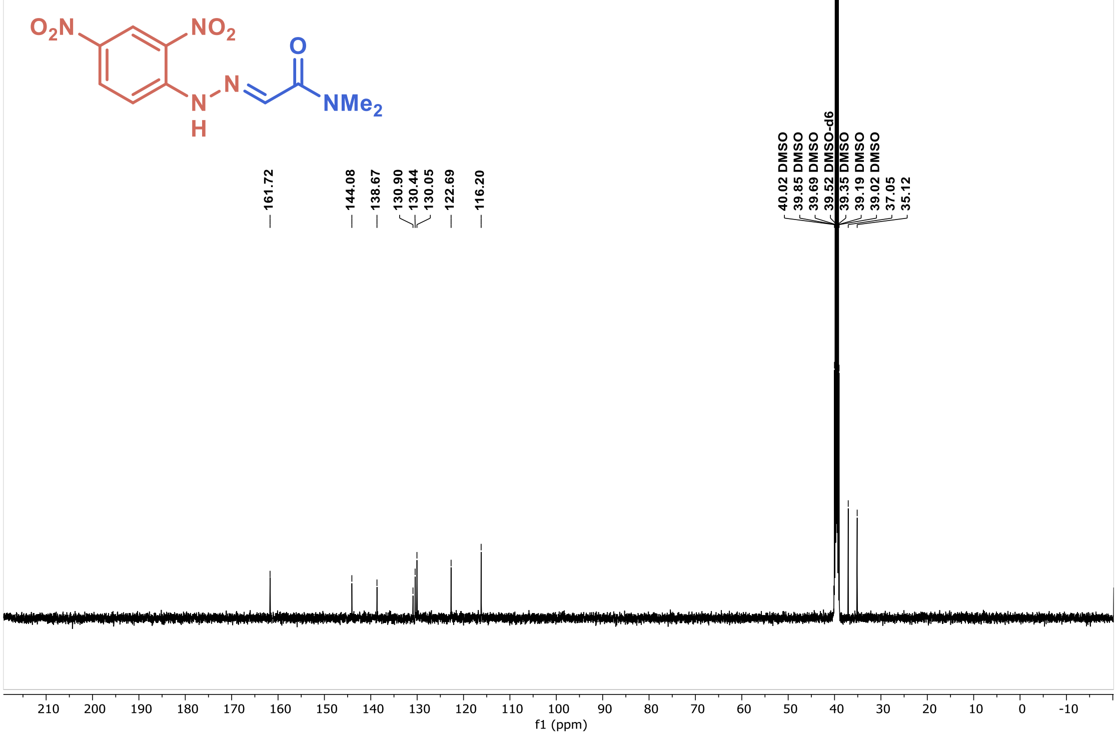

Compound **2a-*d*2**: ^{1}H NMR (300 MHz, CDCl$_3$)

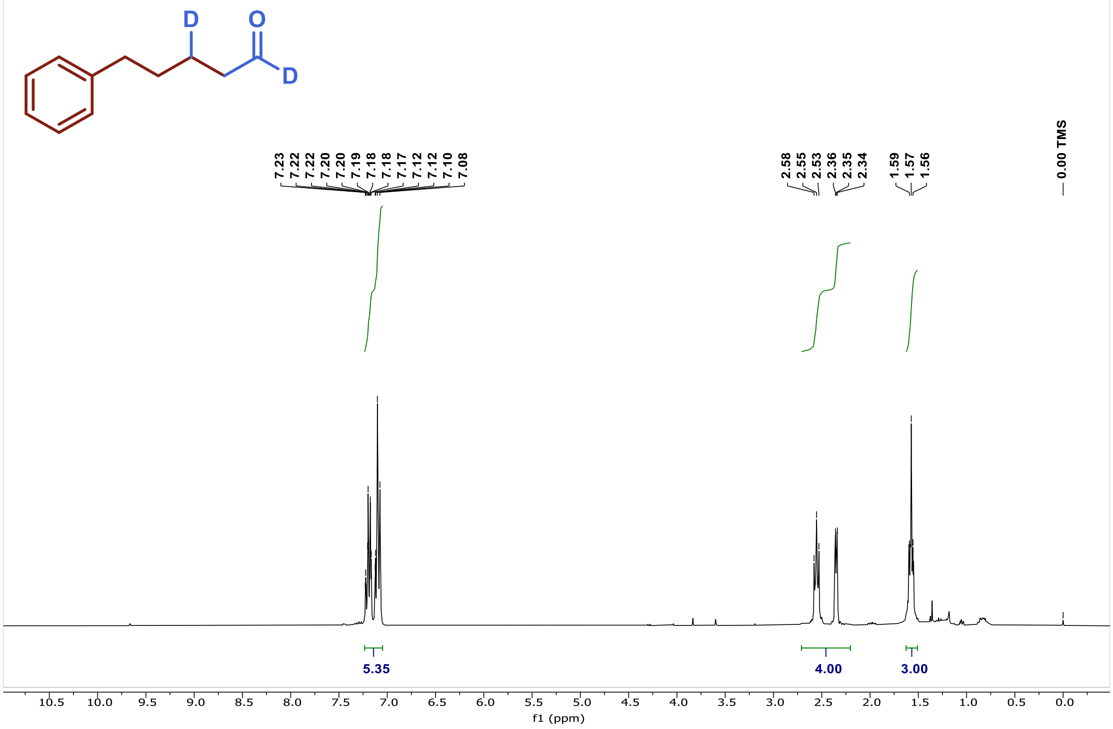

Compound **2a-*d*2**: ^{13}C NMR (75 MHz, CDCl$_3$)

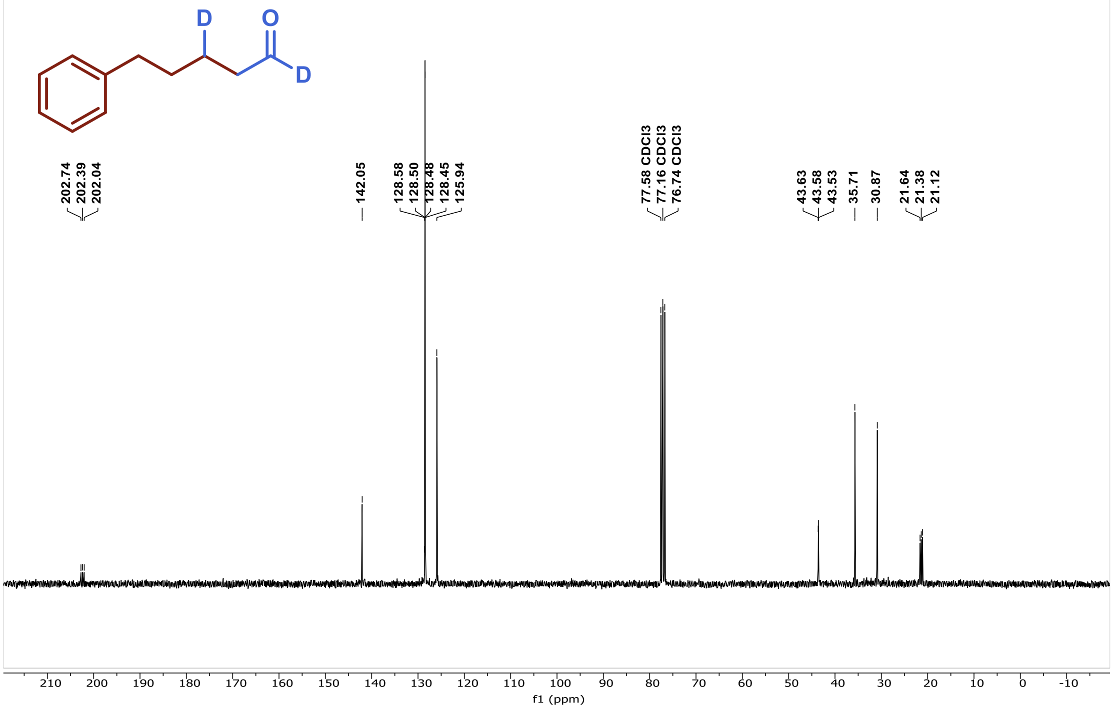

Compound **2a-*d*₂**: DEPT 135

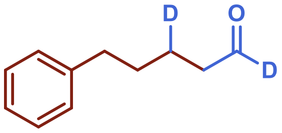

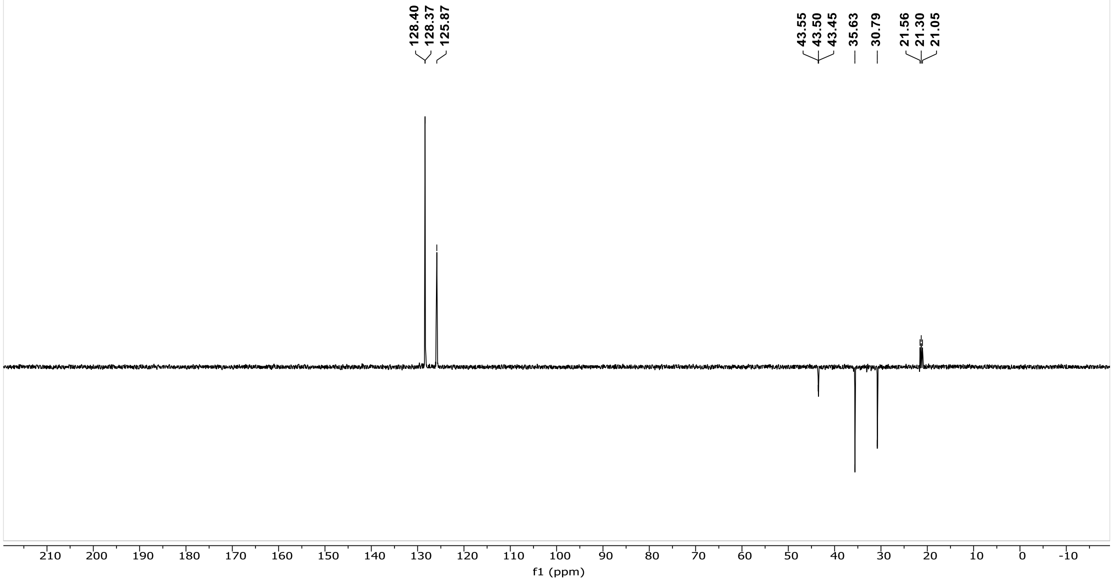

Unexpected products

Compound **1aa'**: ^{1}H NMR (300 MHz, $CDCl_3$)

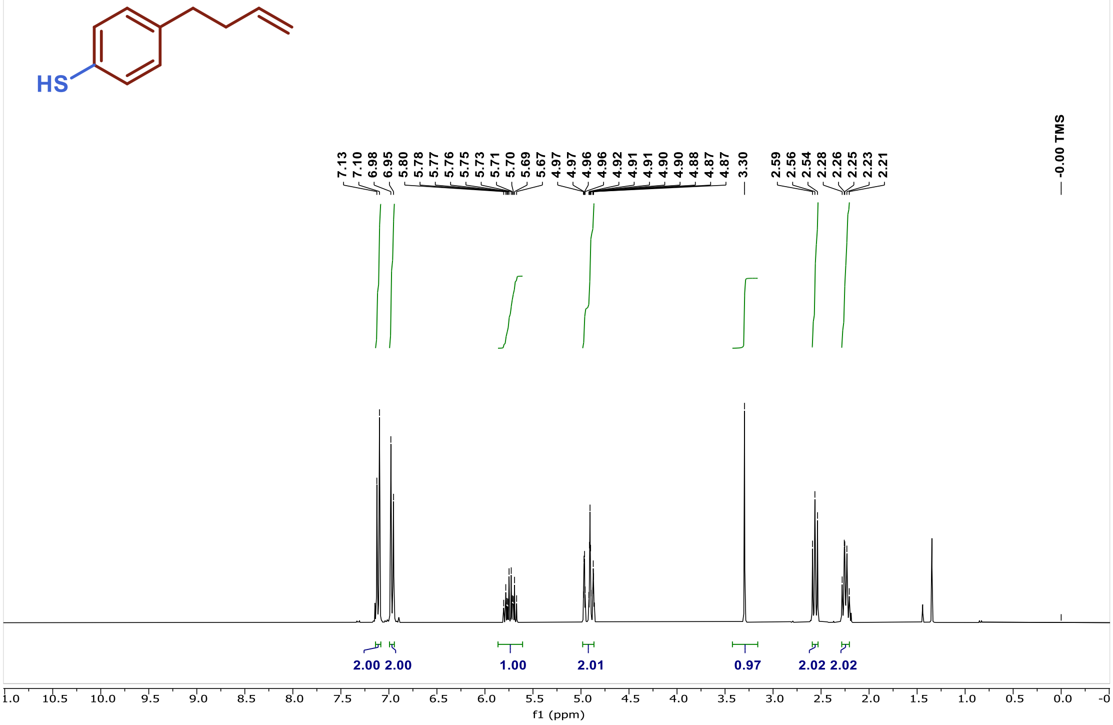

Compound **1aa'**: ^{13}C NMR (75 MHz, $CDCl_3$)

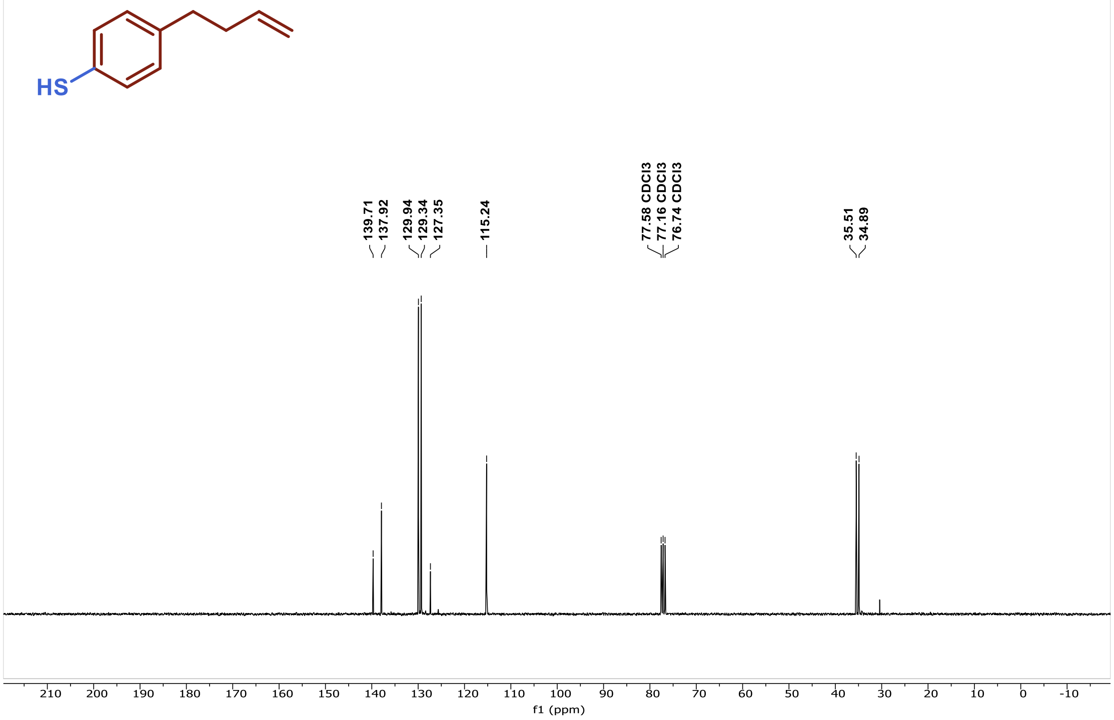

Compound **1ab'**: ^{1}H NMR (300 MHz, $CDCl_3$)

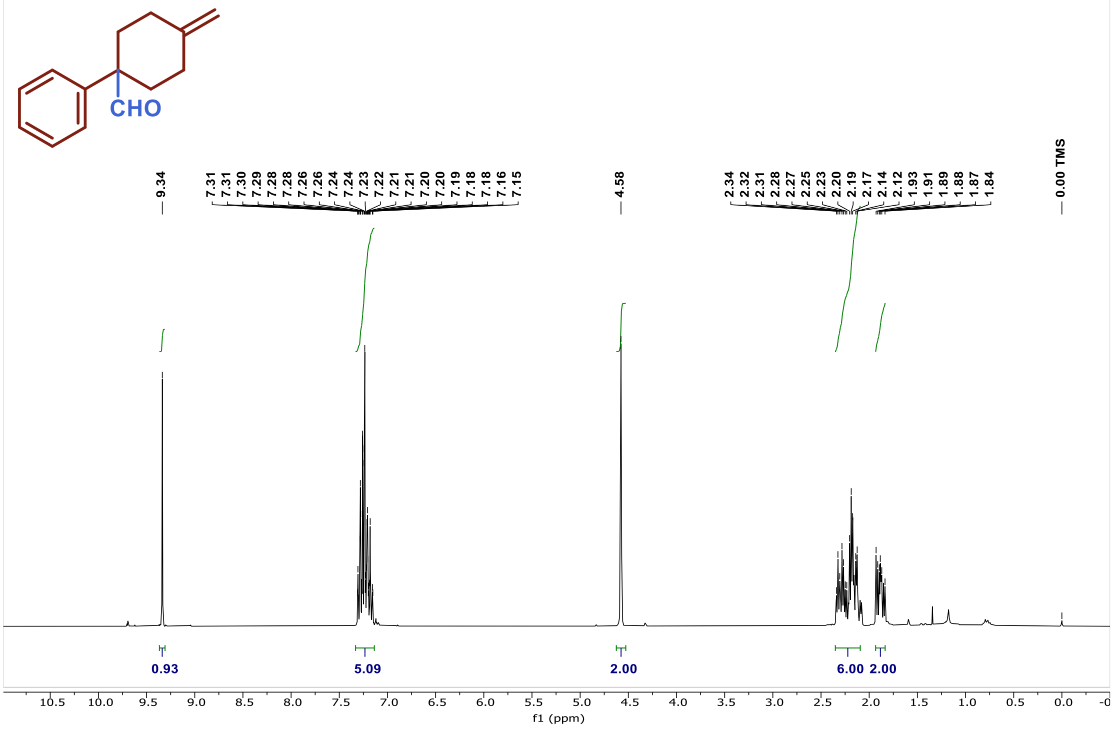

Compound **1ab'**: ^{13}C NMR (75 MHz, $CDCl_3$)

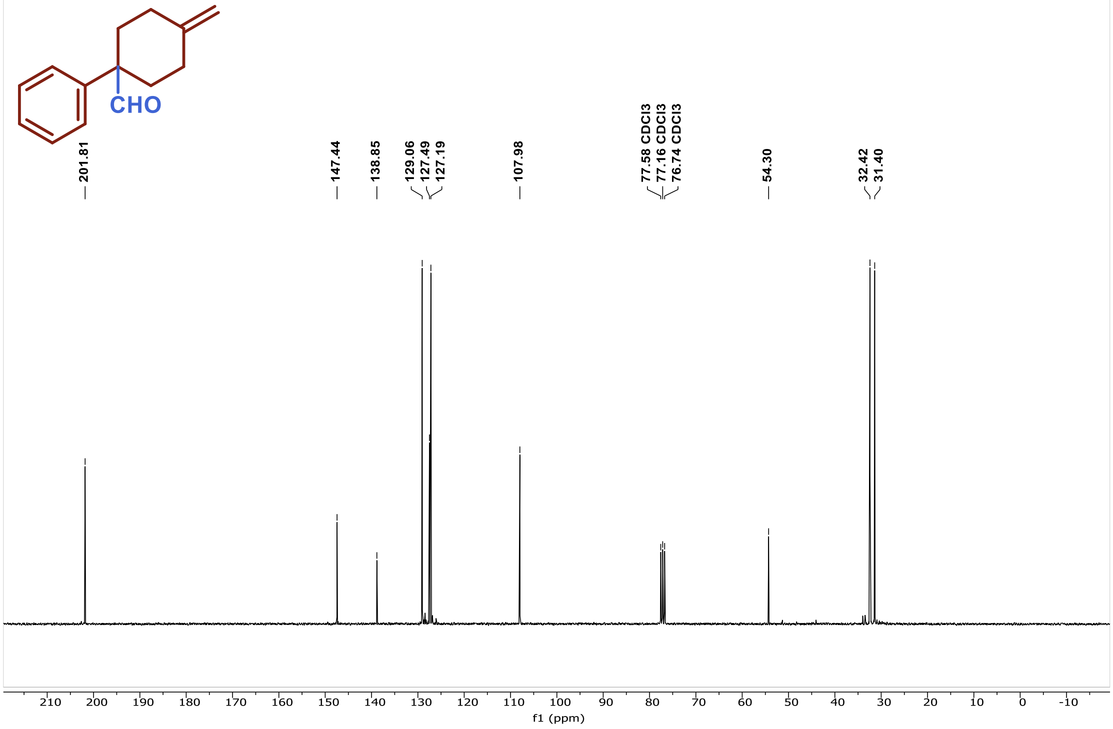

Supplement: Supplementary file 1 [file ja5c17824_si_001.pdf]
